# Problematic gaming, problematic social media use, and psychological well-being in adolescents: exploring networks of symptoms in 10 European countries

**DOI:** 10.1186/s13034-026-01123-3

**Published:** 2026-06-21

**Authors:** Andrea Stašek, Dimitri Löchner, Meyran Boniel-Nissim, Natale Canale, Regina van den Eijnden, Jana Furstova, Tommaso Galeotti, Sabina Hulbert, Helena Jeriček Klanšček, Claudia Marino, Lukas Blinka

**Affiliations:** 1 Psychology Research Institute, Faculty of Social Studies, Masaryk University, Brno, Czech Republic; 2 Institute of Psychology, University of Tartu, Tartu, Estonia; 3 Department of Educational Counselling, The Max Stern Academic College of Emek Yezreel, Afula, Israel; 4 Olomouc University Social Health Institute, Palacký University Olomouc, Olomouc, Czech Republic; 5 Department of Developmental and Social Psychology, University of Padova, Padova, Italy; 6 Interdisciplinary Social Science, Utrecht University, Utrecht, The Netherlands; 7 Centre for Health Services Studies, University of Kent, Canterbury, UK; 8 National Institute of Public Health, Ljubljana, Slovenia

**Keywords:** Adolescents, Problematic gaming, Problematic social media use, Escapism, Well-being, Loneliness, Psychosomatic symptoms, Network analysis, HBSC

## Abstract

**Background:**

Studies that jointly examine the specific symptoms of problematic gaming and problematic social media use with comparable conceptualizations and link them to adolescent psychological well-being remain scarce.

**Methods:**

This study addresses this gap with an effective representative sample of *n* = 33,586 adolescents aged 11–15 years across 10 European countries, collected in the 2021-22 Health Behaviour in School-aged Children (HBSC) survey. A network approach was applied to data about problematic media use (i.e., Internet Gaming Disorder Scale, Social Media Disorder Scale) and psychological well-being (i.e., WHO-5 Well-being Index, Multiple Health Complaints Scale, and Loneliness).

**Results:**

In gaming and social media, either separately or together, escapism was both the most common and the key bridge symptom: it was connected to loneliness in both genders and to overall well-being and nervousness in girls. Behavioral symptoms that reflect negative consequences of use (i.e., conflict, deception, and problems with others) were strongly related across gaming and social media, suggesting that they reflect a general condition rather than a behavior linked to a specific media use.

**Conclusions:**

The findings highlight the central role of escapism in the interplay between adolescent problematic media use and psychological well-being, with some gender-specific differences. Symptom-level focus can guide prevention and intervention strategies. It can also prompt further research to move beyond aggregated symptom scores to better capture the heterogeneity of problematic media use as well as how specifically relevant the symptoms are for the two problematic behaviors in relation to psychological well-being.

**Supplementary Information:**

The online version contains supplementary material available at https://doi.org/10.1186/s13034-026-01123-3.

## Introduction

Digital media are deeply embedded in the daily routines of adolescents, often serving as their central source of entertainment, communication, and information. Common activities include social media use, gaming, and online video viewing [1–3]. Motivations span from pedagogical to social interaction, with entertainment dominant [4]. The widespread adoption and the intensity of the use of digital media has raised important questions about its implications for adolescent health and well-being. Among European 15-year-olds, 96% use social media daily and 37% spend three or more hours on the platforms [5]. Moreover, one-third of 11-15-year-olds play games daily, and 22% dedicate at least four hours to gaming in one session [6]. This level of engagement raises concerns about potential overuse and its association with psychological well-being.

The association between digital media use and well-being is complex, influenced by individual characteristics, the nature of media engagement, and the content encountered online [4, 7, 8]. Moderate media use can mitigate feelings of loneliness and stress [9, 10], facilitate mental health support and treatment [11–13], and support subjective well-being and cognitive, social, and emotional outcomes [14]. On the other hand, problematic media use can also hinder psychological well-being. Therefore, in this study, we focused on the symptom level of the aforementioned constructs and applied the network approach to adolescent data from 10 European countries.

### Problematic gaming

Gaming is a prevalent activity among adolescents and it may fulfill key psychological needs, including autonomy, competence, and relatedness, as proposed by the Self-Determination Theory [15]. Adolescents who play in a harmonious, low-risk modality (i.e., who actively play but report no or few problematic gaming symptoms) report better health-related outcomes compared to their non-gaming peers [16, 17]. To this point, the DSM-5-TR refers to (Internet) Gaming Disorder (IGD) as a gaming pattern that results in significant impairment or distress, and that is characterized by meeting at least five of nine criteria adapted from both substance-based and behavioral addictions (i.e., preoccupation, tolerance, withdrawal, persistence, escape, problems, deception, displacement, and conflict) [18]. More recently, the World Health Organization officially recognized Gaming Disorder and Hazardous Gaming in the ICD-11, following decades of research on problematic gaming [19].

A recent international report [6] states that slightly more than 10% of adolescents are at risk for problematic gaming (PG), which is concerning given the repeatedly observed associations between a possible disorder and negative health-related outcomes, such as an increased internalization of symptoms [20], reduced peer support [21], elevated loneliness, and psychosomatic symptoms [16]. Longitudinal research shows increases in depressive feelings, anxiety, social anxiety, lower academic performance [22], loneliness, more aggressive behavior [23], lowered life satisfaction, and lowered perceived social competence [24]. In general, gaming and problematic gaming is much more prevalent among boys than girls – among European adolescents, the prevalence in boys is two-to-three times higher compared to girls. Also, the phenomenon seems stable irrespective of the boys’ age, while girls tend to play less and have less related issues as they grow older [6].

### Problematic social media use

In parallel with gaming, social media use has emerged as a central activity in adolescence, albeit with distinct behavioral dynamics and psychological correlates. Social media use is a complex set of online activities, including passive (i.e., viewing content, liking social media posts, stories, videos) and active social media use (i.e., generating content, messaging). Similar to PG, problematic social media use (PSMU) refers to a compulsive and excessive pattern of social media engagement that results in significant distress or functional impairment in daily life [25, 26]. Unlike PG, PSMU is not currently recognized as a formal disorder in the DSM-5-TR or the ICD-11. However, it has been considered as potentially falling within the ICD-11 broader classification of “Other specified disorders due to addictive behaviors” — a category that includes conditions that cause marked distress or impairment but that does not meet the full diagnostic criteria for recognized addictive disorders [27, 28].

Recent international surveys suggest that approximately one in 10 adolescents meet the criteria for PSMU with less pronounced gender differences: PSMU is more common in girls than in boys (14% vs. 8% in 15-year-olds) and the difference is mild compared to the differences in PG [6]. PSMU is still commonly characterized with a framework that originated in substance abuse [29], like using the DSM-5 framework to endorse five or six out of nine criteria. An increasing body of research highlights the addictive potential of PSMU and its associations with a range of psychological and somatic symptoms [30, 31]. Specifically, PSMU has been longitudinally linked to reduced mental health and life satisfaction [32, 24] and heightened attention problems, impulsivity [33], and loneliness [34]. Systematic reviews also highlight bidirectional associations between PSMU and elevated levels of anxiety, depression, low self-esteem, loneliness, and social isolation [35, 36]. Not only may problematic use contribute to emotional and social difficulties, but a minority of adolescents who experience psychological vulnerabilities — such as emotional distress, low psychological well-being, or limited social support — may also turn to social media as a maladaptive coping strategy, which reinforces problematic patterns and initiates a potential feedback loop of worsening mental health [37, 38]. However, the measurement can have an effect on observing the links. For example, Di Blasi et al. [39] and Marttila et al. [40] presented complex and mixed results in their longitudinal assessments of PSMU and psychological distress, loneliness, and life satisfaction.

### Comparing problematic gaming and problematic social media use

Although substantial research has addressed PSMU and PG separately, only a few studies have examined them concurrently. One research strand explored their association and found small to moderate correlations at both the disorder and symptom levels [41–43], but the symptom clustering within each condition supports their distinctiveness and aligns with the spectrum hypothesis of problematic internet use [44].

Another line of inquiry has compared PSMU and PG to other mental health difficulties — which, for instance, is needed to evaluate the pathological relevance of PSMU and which lacks formal diagnostic recognition. While some studies report comparable associations with depression, anxiety, stress, sleep issues, and conduct problems [45–47], some indicate PG to be riskier overall [48] while other studies highlight PSMU as more strongly related to anxiety [49, 50]. However, cross-condition comparisons are limited by the inconsistent operationalization of problematic behaviors, with only a few studies employing equivalent measures [42, 46, 47]. To date, no international comparative study has jointly examined PG, PSMU, and their correlates of well-being in adolescents.

In general, the negative consequences of PG that impact one’s life are repeatedly found to be the most important. Specifically, jeopardizing relationships and work or education [51], as well as the loss of interest in other activities [52], are consequences of PG that have the highest clinical significance. In the case of PSMU, deficient self-regulation, prioritization, and preoccupation were identified as the key symptoms [53]. On the other hand, tolerance in gaming [54], loss of control issues in social media use [53], and other cognitive and/or emotion-related symptoms are typically regarded as less clinically impairing and less predictive of adverse outcomes.

### Focusing on the symptom level

Despite a growing body of literature on PG and PSMU, the vast majority of existing studies have investigated them as unitary constructs without considering the potential heterogeneity and differential relevance of their specific symptoms. However, such approach poses limits to our understanding of problematic media use: by pooling diverse symptoms into one construct, we cannot investigate which specific symptoms are the most relevant for psychological well-being and how. Moreover, traditional latent models (as well as the summing/averaging approach) treat each construct as the cause of item responses (and symptom occurrence), which are otherwise independent (i.e., given a specific level of IGD, the symptoms of tolerance and escapism are uncorrelated) [55]. Network models, by contrast, conceptualize disorder constructs as systems of causally interacting symptoms, allowing for the identification of key symptoms. In line with previous suggestions to conceptualize PG as a formative construct (e.g. [56, 57]), there is increasing recognition that symptoms of problematic digital media use vary in severity, prevalence, and clinical importance [53, 58–62]. Emerging empirical studies further support the notion that symptoms can exert causal influences on each other, reinforcing the network perspective [63–65]. Finally, by applying latent models to data that originates from a system of phenomena that function as a network, and thus misspecifying the statistical model and creating an “inference gap” [66], leads to highly biased construct validity, model fit, and parameter estimates (e.g. [67]).

The present study leverages a large, unique, nationally representative data from the Health Behaviour in School-Aged Children (HBSC) study to investigate how individual symptoms of PG and PSMU are associated with adolescent subjective well-being, psychosomatic symptoms, and loneliness. We use the 2021-22 dataset from 10 European countries (Cyprus, Czech Republic, England, Estonia, Iceland, Malta, the Netherlands, North Macedonia, Scotland, and Slovenia) that included measures of both the forms of problematic media use and the above-mentioned psychological well-being indicators. Thanks to the availability of unprecedented size of the sample, we could apply the network approach to reliably estimate the structural associations among problematic media use symptoms and health indicators separately for boys and girls. This is due to the non-invariance and differential functioning for our used measures across the genders (e.g. [68, 69]), and reported gender differences between psychological well-being and problematic media use (e.g. [70]). The network approach allows us to (1) compare the relevance of each symptom across gender groups and types of problematic media use, and (2) examine which symptoms are most centrally or differentially related to the psychological well-being indicators (e.g., subjective well-being, psychosomatic symptoms, and loneliness).

In doing so, we aim to contribute to the ongoing debate about the role of individual symptoms of problematic media use and to inform the design of gender-sensitive and symptom-targeted interventions. Our goal is to answer the following research question: Are there differences in the way the symptoms of PG are related to psychological well-being compared to the connections between PSMU symptoms and the same factors? In our *preregistration*, we hypothesized the strength and valence of several links based on empirical evidence.

## Method

### Sample

Data for this study were drawn from the 2021-22 survey of the Health Behaviour in School-aged Children (HBSC) study (http://www.hbsc.org), a large-scale, international, repeated cross-sectional survey that investigated the health and health-related behaviors of adolescents. The study is conducted every four years in collaboration with the WHO, using a standardized protocol across the participating countries. Representative samples of 11-, 13-, and 15-year-old students are selected through cluster sampling, with schools serving as the primary sampling units. Participation in the survey is voluntary and anonymous. Each country obtained ethical approval in accordance with their national requirements [71].

The 2021-22 survey was carried out in more than 50 countries and regions across Europe and North America. It is important to note, that the data collection overlapped with the COVID-19 pandemic which included various lockdown and isolation periods in the countries. Due to this fact, adolescents were generally more exposed to media (due to partially online schooling) and likely spent less time with their peers offline than before the pandemic. We discuss the limits in the Discussion.

For the present analysis, we included data from 10 countries/regions that administered the questionnaires to assess both PG and PSMU, resulting in an initial sample of 59,039 adolescents. While the PSMU measure was included in the mandatory package of the HBSC protocol (and, thus, collected in all countries), the Internet Gaming Disorder Scale (IGDS) was included in the optional package and administered by a limited number of countries. Only countries that administered both measures were included in this study. Specifically for the present analysis, the inclusion criteria were as follows: (1) no missing values in gender, loneliness, IGDS, SMDS, and psychosomatic symptoms, and (2) no more than two missing out of five WHO-5 well-being items. This resulted in a final analytic sample of *n* = 33,586.

After applying the missing data procedures described below, the final analytic sample consisted of *n* = 33,586 (girls 45.4%), with participants from Cyprus (*n* = 3,722, girls 52.9%), Czech Republic (*n* = 8,276, girls 43.9%), England (*n* = 2,794, girls 47.0%), Estonia (*n* = 3,465, girls 41.4%), Iceland (*n* = 1,663, girls 43.4%), Malta (*n* = 2,691, girls 47.5%), the Netherlands (*n* = 3,325, girls 41.3%), North Macedonia (*n* = 1,531, girls 50.6%), Scotland (*n* = 2,026, girls 44.4%), and Slovenia (*n* = 4,093, girls 45.0%). The mean age of the final sample was 13.6 years (SD = 1.7).

### Measures

#### Internet gaming disorder scale

Problematic gaming behavior was assessed with the nine-item Internet Gaming Disorder Scale (IGDS) [72], which was developed in line with the DSM-5 diagnostic criteria. The scale includes nine dichotomous (yes/no) items to assess behaviors such as preoccupation with gaming, tolerance, withdrawal, loss of control, loss of interest in other activities, continued use despite problems, deception, escape, and conflict. The scale has demonstrated good psychometric properties, including strong internal consistency, structural validity, and criterion validity, with significant associations with gaming time, loneliness, and aggression, and lower well-being indicators, such as self-esteem and prosocial behavior. Longitudinal research further supports its utility in identifying distinct patterns of gaming behavior, particularly among boys [73].

#### Social media disorder scale

Problematic social media use was assessed with the nine-item Social Media Disorder Scale (SMDS) [74], which measures addiction-like behaviors related to social media over the preceding year. Each item is answered in a dichotomous format (yes/no) and reflects one of nine criteria adapted from the DSM-5 framework for IGD, including preoccupation, tolerance, withdrawal, persistence, displacement, problems with others, deception, escape, and conflict. The SMDS has demonstrated solid psychometric properties, including good internal consistency, structural validity, and measurement invariance across gender, age, and countries, supporting its suitability for international adolescent samples [75, 76].

#### World Health Organization’s five well-being index

Mental well-being was assessed using the WHO-5 Well-Being Index (WHO-5), a brief self-report instrument developed by the WHO Regional Office for Europe to measure positive aspects of psychological functioning [77, 78]. The scale consists of five positively worded items that reflect mood, vitality, and general interest over the preceding two weeks, rated on a 6-point Likert scale. Responses are summed to yield a raw score that ranges from 0 to 25, with higher scores indicating greater well-being. In this study, both the raw score and a latent factor score based on the five items were used in analyses.

The WHO-5 has demonstrated solid psychometric properties across diverse populations, including children and adolescents. It has been validated as a reliable and sensitive tool for detecting depression symptoms and it has shown adequate structural validity, internal consistency, and criterion validity in clinical and general youth samples [79, 80]. The scale is freely available in multiple languages and it has been widely used in both clinical practice and population-based research (www.who-5.org*).* We tested the scale for its unidimensionality with the WLSMV robust estimator and the fit was satisfactory (*χ*^*2*^ (*df*) = 476.251 (5); *CFI* = 0.982; *TLI* = 0.965; *SRMR* = 0.021; *RMSEA* [95% *CI*] = 0.093 [0.08;0.097]). A more detailed results overview is available in the Supplementary Material.

#### Multiple health complaints scale

Psychosomatic symptoms were assessed with the Multiple Health Complaints Scale (MHCS; or “HBSC Symptom Checklist”). It captures the frequency of common subjective health symptoms, which are often indicative of stress and psychological distress [81, 82]. The scale includes eight self-reported symptoms: four physical (e.g., headache, abdominal pain, backache, dizziness) and four psychological (e.g., feeling low, irritability or bad mood, nervousness, and sleeping difficulties). Because health complaints tend to cluster and because a universally accepted threshold for clinical significance is lacking, responses reflect subjective experiences rather than categorical diagnoses [83]. In this study, items were analyzed individually rather than as a composite score.

#### Loneliness

Loneliness was assessed with a single-item measure adapted from the Global School-based Student Health Survey (GSHS), which asked participants how often they felt lonely. This item has been widely used in international adolescent health research to estimate the prevalence of perceived loneliness (e.g. [84]). While multi-item scales like the UCLA Loneliness Scale offer more detailed assessments, the single-item format is efficient for large-scale studies and it has demonstrated comparable validity and reliability [85–87].

### Analytical procedure

We analyzed the data with R (version 4.5.1) [88] in RStudio (version 2025.09.0) [89] software. Before the analyses, we preregistered the procedure and hypotheses (https://doi.org/10.17605/OSF.IO/BHVXK). The analytical script is available in the Supplementary Material.

Based on our inclusion criteria, all respondents who did not respond to all of the IGDS, SMDS, MHCS, and loneliness items were deleted for the main analyses (removed *n* = 25,340) to ensure the comparability of the international data. Thus, all respondents with valid answers were used, including those who reported no present symptoms. Respondents who answered less than three items of the WHO-5 were also excluded (removed *n* = 104); if a respondent missed one or two items on this measure, their missing data were imputed with the Classification and Regression Trees [90] method for multiple imputation suited for non-normal and ordinal data [91]. Cases where imputation failed (*n* = 9) were excluded. The final analytic sample was *n* = 33,586. To address the possibility of non-random attrition, we compared the excluded and included participants on gender, age, country, and MHCS items; the results of these comparisons are in the Supplementary Materials. We also aggregated the WHO-5 into latent factor scores due to its one-dimensional nature. This variable was used in the network analyses.

Following the calculation of descriptive statistics from the full sample, and stratified by country and gender, we generated the bivariate correlation matrices of all variables (see Supplementary Material).

We conducted the main analysis with gender subsamples (n_Boys_ = 15,157; n_Girls_ = 12,378) separately due to the evidence for the non-invariance of the IGDS [69]. For all estimated network analyses, we applied the *mgm* estimator [92] due to the combination of binary and ordinal variables. The model had a tuning parameter set to 0.5 and a Haslbeck-Waldorp threshold. In the *mgm* models, the edges are average unstandardized logistic regression coefficients for each dyad of nodes (i.e., average of A → B and B → A). Therefore, the edge strength denotes the probability that both nodes have the same value (i.e., both absent or both present) after controlling for all other nodes.

First, we estimated four 19-node networks in a 2 × 2 (gender vs. media) manner: (1) IGDS, MHCS, WHO-5 items, and loneliness in boys; (2) IGDS, MHCS, WHO-5 items, and loneliness in girls; (3) SMDS, MHCS, WHO-5 items, and loneliness in boys; and (4) SMDS, MHCS, WHO-5 items, and loneliness in girls. Second, we estimated two 28-node networks (one for each gender) that included all of the variables (i.e., IGDS, SMDS, psychosomatic symptoms, well-being, and loneliness). We plotted average layouts for an easier comparison of the networks and included the threshold = 0.0999 to omit edges we considered too weak to interpret meaningfully. We also estimated the centrality indices (i.e., strength, closeness, betweenness, and expecte influence) for each node.

Finally, we tested the stability of the edges and centrality indices results using bootstrapping (*n* = 1000). Following recommendations [93], we considered the correlation stability coefficient (CS) (*r* = 0.7) ≥ 0.5 to indicate good stability. The stability tests are available in the Supplementary Material.

## Results

We present the descriptive statistics for girls, boys, and the overall sample in Table 1. Escapism was among the most prevalent symptoms for both the IGDS and SMDS (i.e., “played games so that you would not have to think about annoying things”/“used social media to escape from negative feelings”). Within the IGDS, boys reported escapism more often (48.3%) than girls (41.2%), although the effect size was small (d = 0.143). In contrast, within the SMDS, girls indicated the escape symptom more frequently (55.9%) than boys (37.6%), with a medium effect size (d = 0.375). Across the MHCS, girls consistently reported a higher prevalence of psychosomatic symptoms than boys. The largest differences were observed for Feeling low and Feeling nervous, both with medium effect sizes (d = 0.549 and d = 0.532, respectively). This pattern is consistent with boys scoring higher on Well-being and lower on Loneliness (both medium effects, d = 0.495 and d = 0.540, respectively). Overall, these results indicate that boys generally experienced more favorable psychological well-being outcomes than girls. A more detailed description of the main variables, as well as stratification by gender and country, is provided in the Supplementary Material.

**Table 1:** Means and standard deviations (in parentheses) of the main variables

	Boys	Girls	Overall	Gender comparison			
	(*n* = 18,348)	(*n* = 15,238)	(*n* = 33,586)	t	df	*p*	Cohen’s d
IGDS Preoccupation	0.426 (0.495)	0.226 (0.418)	0.335 (0.472)	40.110	33572.370	< 0.001	0.433
IGDS Tolerance	0.325 (0.468)	0.202 (0.401)	0.269 (0.443)	26.034	33552.295	< 0.001	0.281
IGDS Withdrawal	0.233 (0.423)	0.144 (0.351)	0.193 (0.394)	21.029	33583.990	< 0.001	0.227
IGDS Persistence	0.238 (0.426)	0.175 (0.380)	0.209 (0.407)	14.438	33417.059	< 0.001	0.157
IGDS Escape	0.483 (0.500)	0.412 (0.492)	0.451 (0.498)	13.085	32630.642	< 0.001	0.143
IGDS Problems	0.220 (0.415)	0.110 (0.312)	0.170 (0.376)	27.897	33281.078	< 0.001	0.298
IGDS Deception	0.154 (0.361)	0.105 (0.307)	0.132 (0.338)	13.261	33563.563	< 0.001	0.143
IGDS Displacement	0.124 (0.330)	0.086 (0.280)	0.107 (0.309)	11.538	33567.861	< 0.001	0.125
IGDS Conflict	0.128 (0.334)	0.0730 (0.260)	0.103 (0.304)	17.014	33444.927	< 0.001	0.182
SMDS Preoccupation	0.225 (0.418)	0.291 (0.454)	0.255 (0.436)	-13.747	31318.770	< 0.001	0.152
SMDS Tolerance	0.186 (0.389)	0.250 (0.433)	0.215 (0.411)	-14.015	30975.198	< 0.001	0.155
SMDS Withdrawal	0.192 (0.394)	0.281 (0.450)	0.233 (0.423)	-19.166	30544.631	< 0.001	0.213
SMDS Persistence	0.294 (0.456)	0.463 (0.499)	0.371 (0.483)	-32.159	31228.273	< 0.001	0.355
SMDS Escape	0.376 (0.484)	0.559 (0.496)	0.459 (0.498)	-34.115	32157.371	< 0.001	0.375
SMDS Problems	0.171 (0.376)	0.222 (0.415)	0.194 (0.395)	-11.616	31097.882	< 0.001	0.128
SMDS Deception	0.138 (0.345)	0.190 (0.392)	0.162 (0.368)	-12.726	30614.656	< 0.001	0.141
SMDS Displacement	0.157 (0.364)	0.218 (0.413)	0.185 (0.388)	-14.344	30624.319	< 0.001	0.159
SMDS Conflict	0.118 (0.322)	0.174 (0.379)	0.143 (0.350)	-14.447	30035.737	< 0.001	0.161
MHCS Headache	1.91 (1.130)	2.48 (1.36)	2.17 (1.27)	-41.097	29643.160	< 0.001	0.458
MHCS Stomach ache	1.62 (0.955)	2.13 (1.20)	1.85 (1.10)	-42.320	28927.059	< 0.001	0.473
MHCS Back pain	1.84 (1.20)	2.12 (1.38)	1.97 (1.29)	-19.531	30484.709	< 0.001	0.217
MHCS Feeling low	2.03 (1.28)	2.78 (1.46)	2.37 (1.41)	-49.509	30486.515	< 0.001	0.549
MHCS Irritability	2.52 (1.31)	3.10 (1.37)	2.78 (1.37)	-39.087	31818.434	< 0.001	0.430
MHCS Nervousness	2.51 (1.33)	3.24 (1.43)	2.84 (1.43)	-48.258	31576.474	< 0.001	0.532
MHCS Problems falling asleep	2.25 (1.46)	2.73 (1.58)	2.47 (1.53)	-28.679	31437.340	< 0.001	0.317
MHCS Dizziness	1.56 (1.04)	2.07 (1.39)	1.79 (1.24)	-37.219	27776.959	< 0.001	0.419
WHO-5 Overall well-being	3.14 (1.07)	2.59 (1.16)	2.89 (1.15)	44.812	31364.351	< 0.001	0.495
Loneliness	2.17 (1.04)	2.74 (1.08)	2.43 (1.10)	-49.045	31934.239	< 0.001	0.540

The table presents means for the groups. The parentheses show standard deviations. In the case of binary variables (IGDS and SMDS items), the mean is equal to the relative frequency. IGDS = Internet Gaming Disorder Scale; SMDS = Social Media Disorder Scale; MHCS = Multiple Health Complaints Scale; WHO-5 = World Health Organization’s Five Well-Being Index; df = degrees of freedom

**Fig. 1:**
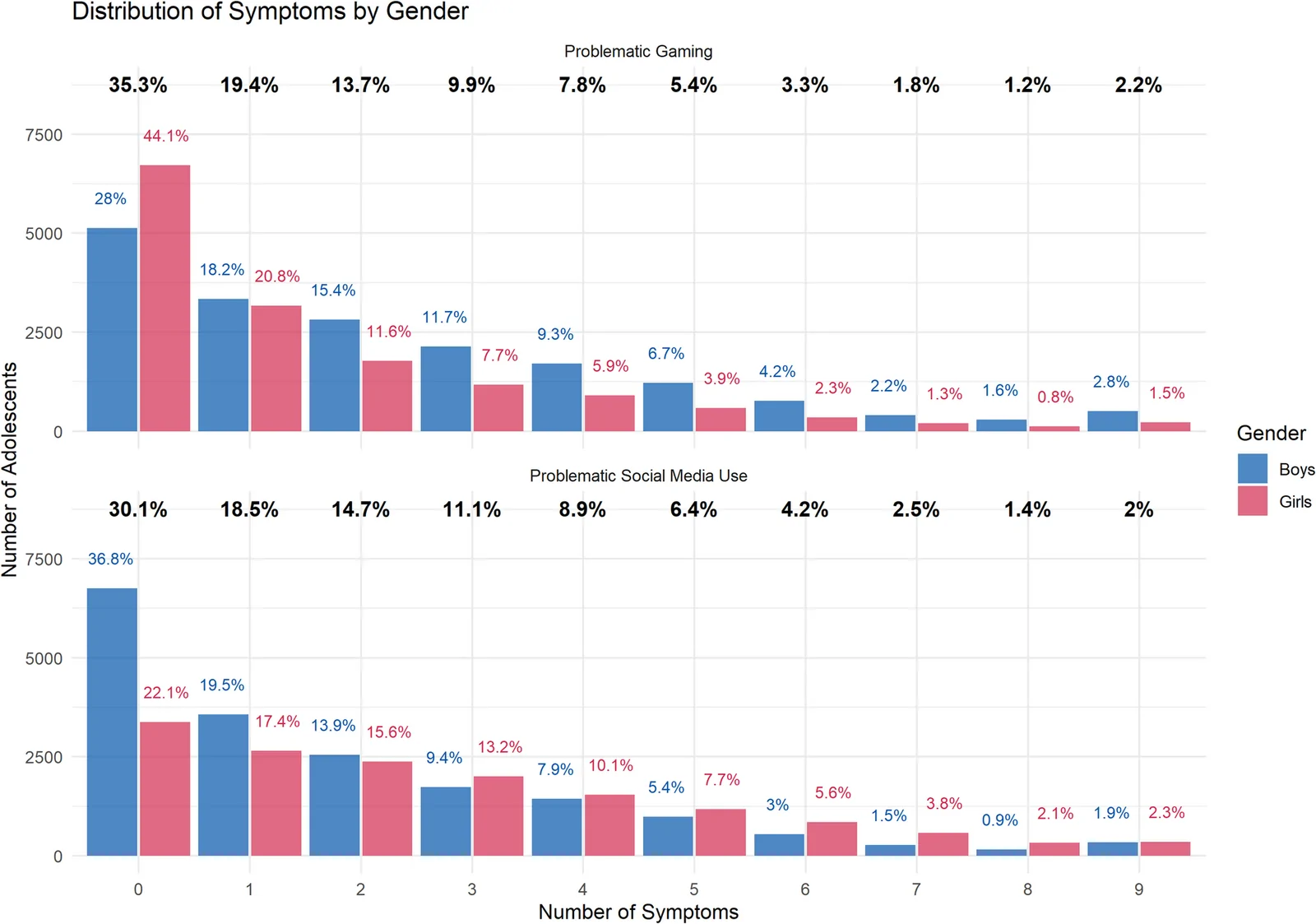
Distribution of symptom prevalence in boys and girls

In Fig. 1, we provide the prevalence of IGDS and SMDS symptoms in the whole population. There are large gender differences in the number of adolescents without any symptoms based on the media used: 28% of boys vs. 44.1% of girls reported no IGDS symptoms, while 36.8% of boys vs. 22.1% of girls reported no SMDS symptoms. Additionally, 48.4% of boys and 23.3% of girls reported gaming (almost) daily. Gaming in more than 4-hour sessions were reported by 23.7% of boys and 9.8% of girls. Regarding online contact with close friends, 47.9% of boys and 55.8% of girls are connected “almost all the time”. A more extensive description of these results and country-specific statistics are available in the Supplementary Material.

### Main results

**Fig. 2:**
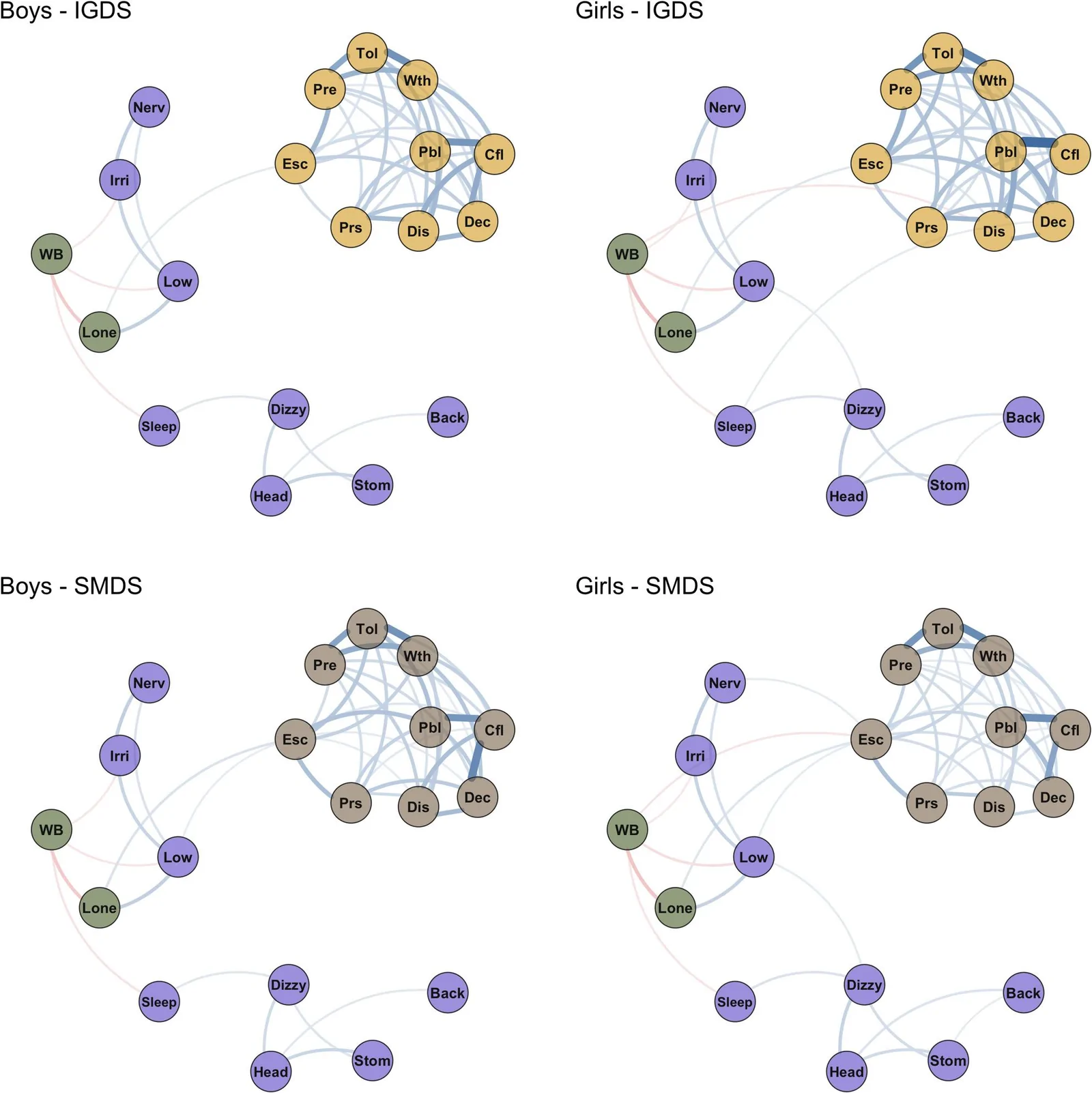
Separate network models for IGDS and SMDS symptoms and psychological well-being in boys and girls. Nodes represent symptoms. Edges represent associations between symptoms. Blue edges indicate positive correlations, whereas red edges indicate negative correlations. Line thickness reflects the expected magnitude of the association. *IGDS* Internet Gaming Disorder Scale, *SMDS* Social Media Disorder Scale, *Pre* Preoccupation, *Tol* Tolerance, *Wth* Withdrawal, *Prs* Persistence, *Ecs* Escape, *Pbl* Problems, *Dec* Deception, *Dis* Displacement, *Cfl* Conflict, *Head* Headache, *Stom* Stomach ache, *Back*  Backache, *Low*  Feeling low, *Irri* Irritability, *Nerv* Nervousness, *Sleep* Sleep Difficulties, *Dizzy* Feeling Dizzy, *WB* Well-being, *Lone* Loneliness.

When we compared our hypothesized and observed structures, we found that most of the assumptions were supported. First, we assumed two major clusters within health: a behavioral cluster, comprising of Deception, Problems, and Conflict, and a cognitive-emotional cluster, comprising Preoccupation, Withdrawal, Tolerance, and Escape. These hypothesized edges were mostly found in the networks (Fig. 2): in boys, we found especially strong edges between Conflict—Deception (IGDS: 0.483; SMDS: 0.681), Withdrawal—Tolerance (IGDS: 0.627; SMDS: 0.620), Conflict—Problems (IGDS: 0.599; SMDS: 0.597), and Preoccupation—Tolerance (IGDS: 0.500; SMDS: 0.510). In girls, several edges were even stronger: Conflict—Problems (IGDS: 0.878; SMDS: 0.650), Withdrawal—Tolerance (IGDS: 0.691; SMDS: 0.670), Preoccupation—Tolerance (IGDS: 0.607; SMDS: 0.604), and Preoccupation—Withdrawal (IGDS: 0.515; SMDS: 0.431).

Second, as expected, we found a moderate negative connection for Well-being—Loneliness in both boys (IGDS: -0.241; SMDS: -0.244) and girls (IGDS: -0.274; SMDS: -0.265), see Fig. 2. Third, we hypothesized that in gaming, Feeling low, Irritability, Nervousness, and Problems falling asleep would be linked to Escape, Conflict, Displacement, and Tolerance, respectively. Our data showed that, in boys, only Loneliness was connected to Escape when all other connections were taken into account. Girls’ data presented connections for Loneliness—Escape, Well-being—Displacement, and Problems falling asleep—Deception. In the context of SMDS, Escape was connected to both Loneliness and Feeling low in boys and Loneliness, Feeling low, Well-being, and Nervousness in girls. Escapism had an important associative role between mental health, psychosomatic symptoms, and problematic media use, similar to the role of a bridge. The centrality of the nodes, the stability of the results, and the country-specific results are available in the Supplementary Material.

**Fig. 3:**
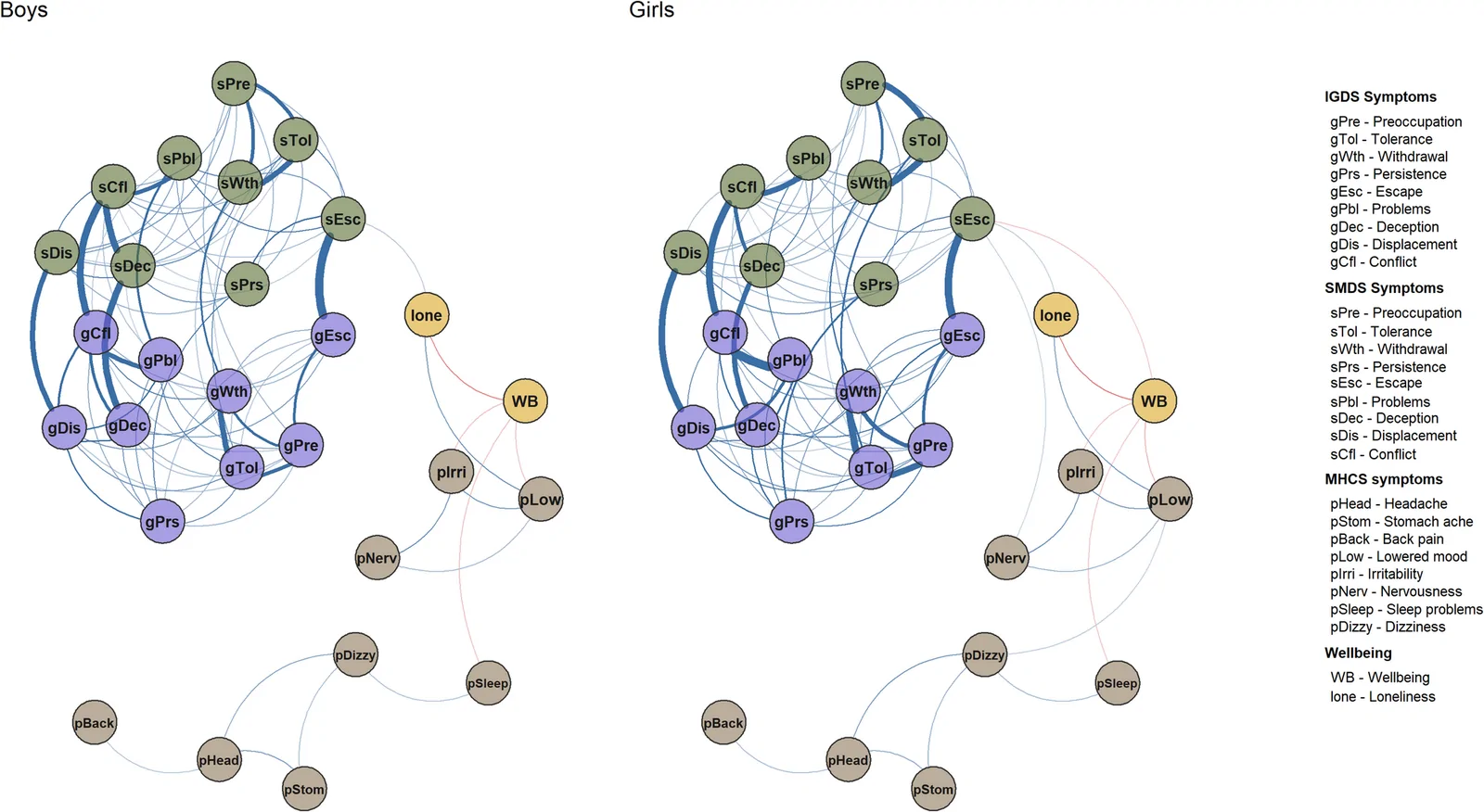
Network models of problematic media use symptoms (IGDS, SMDS) and psychological well-being (MHCS, WHO-5, and Loneliness) in boys and girls. Nodes represent symptoms, and edges represent associations between symptoms. Blue edges indicate positive correlations, whereas red edges indicate negative correlations. Line thickness reflects the expected magnitude of the association. *IGDS *Internet Gaming Disorder Scale, *SMDS* Social Media Disorder Scale, *MHCS* Multiple Health Complaints Scale

When taking into account all of the IGDS and SMDS symptoms (Fig. 3), we found that in both boys and girls, SMDS Escape—Loneliness (boys: 0.121; girls: 0.111) was the relationship that linked problematic media use symptoms and psychological well-being. In girls, two additional edges were connecting psychological well-being and problematic media use: SMDS Escape—Well-being (-0.113) and SMDS Escape—Nervousness (0.104).

The large network (Fig. 3) also allowed us to test how specific IGDS and SMDS symptoms connect to each other: the strongest cross-media edges were within Escape (boys: 0.699; girls: 0.663), Conflict (boys: 0.648; girls: 0.634), Deception (boys: 0.605; girls: 0.461), Displacement (boys: 0.535; girls: 0.612), and Problems (boys: 0.390; girls: 0.326). Thus, across the two media, the behavioral symptoms were more mirrored than the cognitive-emotional ones. The centrality of the nodes, stability of the results, and country-specific results are provided in the Supplementary Material.

## Discussion

Our goal was to explore the interconnectedness of the individual symptoms of problematic gaming (PG), problematic social media use (PSMU), well-being, psychosomatic symptoms, and loneliness in adolescent boys and girls. Rather than relying on traditional sum or averaged symptom scores, this study applied a novel statistical approach (i.e., network analysis) to identify the most impactful symptom-level associations. Most research in PG and PSMU applies sums or averages which can lead to severe bias due to strict assumptions behind these operations [55, 66, 67], therefore our study use the network approach that aligns with theoretical functioning of PG and PSMU.

The cluster of anxiety- and depression-related symptoms (e.g., irritability, nervousness, feeling low) was meaningfully associated with lower subjective well-being and stronger feelings of loneliness in both boys and girls. Loneliness itself was the link to problematic media use (both to PG and PSMU) through escapist motivation to engage in media use across (or vice versa, escapism was the link to feeling lonely and to other psychosocial issues, as our analyses were applied to cross-sectional data). The dyad of escapism and loneliness seems, therefore, crucial for both research and potential interventions in this area. Lastly, certain symptoms (mainly the negative consequences of problematic media use) were linked across media, suggesting that symptoms are partially independent of specific media and reflect a more general pattern.

First, we identified Loneliness and the Escape symptom as repeatedly connected constructs across genders and media types when we assessed the problematic media use separately. Therefore, we support the evidence that lonely adolescents tend to use media to cope with or escape from negative feelings more often and/or that adolescents who use media to cope with negative feelings tend to be lonelier (e.g. [94–96]). Moreover, social media escapism was also positively linked to well-being, indicating its more complex role. While escapism has previously been found to be associated with distress [97], it was also criticized for its diagnostic utility within PG [98] and difficult operationalization in the context of both problematic gaming and streaming [99]. Certain levels of escapism might be related to general media mood modification properties, but in more extreme forms, the sole coping strategy may hint at potential problematic behavior [100, 101]. Simply put, escapism might not be a clinically meaningful symptom, per se, but rather a contextually important motive for why the digital medium is reached in the first place, and it should be considered as a symptom only in its extreme form. Indeed, recent studies suggest that escapism is very common (e.g., present in half of adolescent male gamers; [69]).

In girls, nervousness and feeling low were also connected to social media escapism. It is possible that these partially correlated relationships were significant in girls and not boys due to the higher prevalence of these problems in girls, with a moderate effect size. Existing literature has generally not found gender differences in escapism mechanisms [99], except for pornography use [102]. Nonetheless, there is evidence that girls and boys tend to regulate emotions differently: girls are more likely than boys to seek social and emotional support [103, 104], which may explain their use of social media for this purpose, given its broad opportunities to communicate. This may enable girls, more than boys, to cope with a wider range of negative emotions (i.e., loneliness, low well-being, nervousness, and feeling low). On the other hand, if the use of social media to escape negative feelings does not provide real gratification but, on the contrary, generates further negative emotions (such as feeling low due to social media use generating negative social comparison), that, in line with the compensatory internet use theory [37], may reinforce the problematic nature of the behavior and the motivation may become dysfunctional.

Second, the network models that included both media highlighted the most prominent bridge symptoms connecting media use and psychological well-being. While escaping to social media, both girls and boys can feel lonelier, but girls can also have lower well-being and feel nervous or anxious; however, the associations can very well be bidirectional, meaning that social media escapism can be the result of feeling lonely, unwell, and nervous, and simultaneously the cause, too. Previous research has yielded mixed findings on gender differences in usage types and their causes and outcomes. While some studies and reviews have found no gender differences [105, 106], Fardouly et al. [107] highlight differences in users’ experiences. Booker et al. [108] suggested that with higher age, the effect of social media interactions on well-being in girls might be mediated more by social comparison processes (i.e., the older adolescent girls are, the more they are affected by social comparison). In contrast, boys’ level of social interaction in early adolescence is not linked to worse well-being. Frison and Eggermont [109] found that Facebook has negative impacts when used actively by boys and passively by girls. Girls are also more preoccupied with their social media appearance than boys [110] and report higher levels of appearance anxiety [111, 112] when using social media. In general, girls tend to compare themselves to others online more often and use social media to seek information, while boys search other profiles to seek friends [113]. Therefore, the types of motivation to escape negative feelings may be more social for boys (i.e., focused on loneliness) and broader (i.e., encompassing social and emotional aspects) for girls. Similarly, escapism can increase loneliness and anxiety and decrease overall well-being.

Third, we found that individual PG and PSMU symptoms have various roles and some form stronger dyads and clusters than others, as expected. Within both media, two groups of more interconnected symptoms emerged: the first comprised of symptoms that captured problematic behavior and negative consequences (i.e., the symptoms labelled Conflict, Problems, Deception), while the second consisted of cognitive-emotional symptoms (i.e., Preoccupation, Withdrawal, Tolerance, and Escape). The groups thus suggest the existence of a two-cluster structure of problematic media use. Even though some previous studies that used a latent factor approach specified a one-factor solution in both the IGDS [72, 114, 115] and the SMDS [74, 76, 116], these papers usually applied the controversial Hu and Bentler cut-offs for model fit evaluation [117–119]. Our findings align with multiple studies that employ the network approach [120–122], latent factors [123, 124], and their combination [125]. This study thus supports the notion that research should consider the various roles of symptoms, rather than treating them as interchangeable, for example, by summing or averaging items without strong clinical support for the scoring process [97]. It also hints at a potential problem with using a cut-off as a simple sum score (i.e., 5 + symptoms, as is suggested by DSM-5 in the case of the PG, and often adopted for other potential behavioral addictions) and the model fit evaluation cut-offs.

Finally, thanks to our unique design, we could examine whether similar symptoms occur across media. We found that the negative consequences of problematic media use tend to co-occur across the two media (e.g., an adolescent who lies about social media use is also likely to lie about gaming). A similar pattern was observed for conflicts with family regarding usage, the tendency to use media to escape negative feelings, losing interest in other activities, and increased usage time. Current media typically encompass a variety of activities and are widely embraced by adolescents; thus, the existence of isolated gaming or social media use is relatively rare. For example, online games often incorporate chat and gambling features, while social media networks frequently include games. Cross-media multitasking is also frequent [126]. In the context of disordered use, Thege et al. [127] found that 12.3–37.2% of their problematic gamers also reported aspects of problematic work, sex, eating, and gambling. Moreno et al. [50] found a significant overlap of problematic internet use, social media use, and gaming. Our findings similarly point to a need for various levels of analysis – on one hand, it is necessary to examine isolated media activities in detail; on the other hand, for many adolescents, these activities are mixed and interconnected in practice. Thus, while the field was recently more focused on theories and models of specific problematic media uses, we suggest more attention to a broader theory of problematic media use, as has been attempted by Davis and Kim [128, 129], Caplan [59], Dong and Potenza [130], and Brand et al. [131]. Indeed, it is plausible, especially for a vulnerable subset of adolescents, that different maladaptive behaviors may stem from similar underlying psychological mechanisms, individual characteristics and vulnerabilities, and levels of psychosocial well-being [132, 133, 134]. Therefore, interventions aimed at addressing the main shared features of problematic media use (e.g., escapism motivations, reward cycles, and social expectations) may help promote more adaptive media use, as well as adolescents’ well-being and socio-emotional skills during this developmental stage.

### Limitations

Our study has several limitations that affect its generalizability. First, our findings are bound to the measurement tools used in the HBSC survey. It has been well-argued before that the cross-study comparison of behavioral addiction results are challenging due to the varying wording of the individual symptoms. Our study is strong in its sampling, despite using measures that have been previously criticized for inflating the prevalence of risk (e.g. [135]). Second, in a previous paper, we found that the IGDS is only partially invariant across genders and countries [69]; however, this should not seriously affect the relationships across the variables in our study. However, using and comparing (latent) means could be problematic due to the application of latent models to network-based constructs [67] and due to insufficient latent factor invariance. Third, some analyses presented in the Supplementary Material (especially those involving the 28-node networks modeled with country-level subsamples) may lack sufficient power due to the smaller sample size and should be interpreted with caution. Our goal was to capitalize on the unique sample at hand and conduct a detailed examination of the phenomenon both internationally and at the national and gender levels. Network analyses are sample-demanding; therefore, estimating large networks with data from < 1,000 samples can be easily underpowered. Fourth, our data are cross-sectional and, despite the network approach assuming causal mutual relationships, our results present mere directionless associations. Fifth, the specifics of gender differences in usage remain unknown, and more in-depth, qualitative, and content-oriented research is necessary. The differences likely lie in the contextual aspects of the media (i.e., types of games played, nature of social interactions), which were not detectable in our measurement. The role of escapism itself, then, can vary in different contexts. Sixth, the problematic media use, psychosomatic symptoms, and loneliness were measured with only one item, posing a threat to the reliability of each symptomatic construct. Seventh, the sample included only school-attending adolescents, limiting the generalizability to the broader youth population, particularly those not enrolled in school or those experiencing social withdrawal. Finally, the data collection overlapped with the COVID-19 pandemic which was characterized by increased screen time [136] and game and social media use [137]; however, according to available findings, the trend did not disappear after the lockdowns were lifted [138]. Taken together with the fact that the data were collected outside of the lockdown period, we believe our findings are relevant even for post-pandemic times.

## Conclusion

Our results leverage a unique internationally synchronized representative sampling of adolescents to show the problematic gaming and problematic social media use symptoms that are linked to specific psychological well-being issues. Moreover, the symptom-level network analysis allowed us to examine the inter-symptom relations within the behaviors as well as cross-media associations. The assessment of gender differences showed that, (1) in general, problematic media use is associated with worse well-being, higher loneliness, and several psychosomatic symptoms, (2) when both problematic gaming and social media use are taken into account, social media escapism connects to loneliness in boys and loneliness, nervousness, and well-being in girls, highlighting the slightly different roles of the usage, and (3) primarily the behavioral components of problematic media use are strongly related across media (i.e., deceiving, prioritizing, having conflicts, or increasing usage due to both gaming and social media use). These results clearly indicate the need for further investigation of escapism motivation and its role in disorder development. Furthermore, future research should focus on differentiating between the symptoms of problematic media use, rather than using aggregated indices of problematic use (e.g., means/sums), which can lead to biased results. Overall, the results of the present study may inform prevention efforts and interventions that can address the relevant symptoms of the two problematic behaviors in relation to psychological well-being.

## Supplementary Information

Supplementary Material 1.

Supplementary Material 2.

## Data Availability

Data are available upon request at hbsc.org. The analytical script is available at [osf.ie](https:/osf.io/bhvxk/overview? view_only=026d4b673f7d40fdabc94a803b6c6a35).

## Abbreviations

DSM-5-TR: Diagnostic and Statistical Manual of Mental Disorders Fifth Edition – Text Revision
HBSC: Health Behaviour in School-aged Children
ICD-11: International Classification of Diseases Eleventh Revision
IGD: Internet Gaming Disorder
IGDS: Internet Gaming Disorder Scale
MHCS: Multiple Health Complaints Scale
PG: Problematic Gaming
PSMU: Problematic Social Media Use
SMDS: Social Media Disorder Scale
WHO: World Health Organization
