# Supplementary material for: Problematic gaming, problematic social media use, and psychological well-being in adolescents: exploring networks of symptoms in 10 European countries
Source: Child Adolesc Psychiatry Ment Health. 2026 Jun 21;20:125. doi: 10.1186/s13034-026-01123-3 (PMC13576324; doi:10.1186/s13034-026-01123-3)
Supplement: Supplementary file 1 [file 13034_2026_1123_MOESM1_ESM.html]

version https://git-lfs.github.com/spec/v1
oid sha256:010c0bf1f12e80a370595a72d86a3d88dba15bd4dbda4423874557313bb965ef
size 927159
