# Supplementary material for: Problematic gaming, problematic social media use, and psychological well-being in adolescents: exploring networks of symptoms in 10 European countries
Source: Child Adolesc Psychiatry Ment Health. 2026 Jun 21;20:125. doi: 10.1186/s13034-026-01123-3 (PMC13576324; doi:10.1186/s13034-026-01123-3)
Supplement: Supplementary file 2 [file 13034_2026_1123_MOESM2_ESM.html]

Supplementary Materials


Code 

- Show All Code
- Hide All Code

# Supplementary Materials

#### December 2025

# Descriptives

## Table 1. Social media use frequency by region and gender.

|  | CY | | CZ | | EE | | GB-ENG | | GB-SCT | | IS | | MK | | MT | | NL | | SI | | Overall | |
| --- | --- | --- | --- | --- | --- | --- | --- | --- | --- | --- | --- | --- | --- | --- | --- | --- | --- | --- | --- | --- | --- | --- |
|  | Boys (N=1754) | Girls (N=1968) | Boys (N=4646) | Girls (N=3630) | Boys (N=2030) | Girls (N=1435) | Boys (N=1481) | Girls (N=1313) | Boys (N=1126) | Girls (N=900) | Boys (N=941) | Girls (N=722) | Boys (N=756) | Girls (N=775) | Boys (N=1412) | Girls (N=1279) | Boys (N=1953) | Girls (N=1372) | Boys (N=2249) | Girls (N=1844) | Boys (N=18348) | Girls (N=15238) |
| Days gaming /week |  |  |  |  |  |  |  |  |  |  |  |  |  |  |  |  |  |  |  |  |  |  |
| (Almost) never | 133 (7.6%) | 542 (27.5%) | 0 (0%) | 0 (0%) | 0 (0%) | 0 (0%) | 0 (0%) | 0 (0%) | 0 (0%) | 0 (0%) | 26 (2.8%) | 202 (28.0%) | 0 (0%) | 0 (0%) | 57 (4.0%) | 153 (12.0%) | 7 (0.4%) | 26 (1.9%) | 0 (0%) | 0 (0%) | 223 (1.2%) | 923 (6.1%) |
| < 1 day | 111 (6.3%) | 300 (15.2%) | 316 (6.8%) | 1047 (28.8%) | 96 (4.7%) | 299 (20.8%) | 68 (4.6%) | 271 (20.6%) | 32 (2.8%) | 151 (16.8%) | 44 (4.7%) | 132 (18.3%) | 0 (0%) | 0 (0%) | 62 (4.4%) | 145 (11.3%) | 47 (2.4%) | 185 (13.5%) | 145 (6.4%) | 507 (27.5%) | 921 (5.0%) | 3037 (19.9%) |
| 1 day | 102 (5.8%) | 164 (8.3%) | 261 (5.6%) | 501 (13.8%) | 78 (3.8%) | 194 (13.5%) | 61 (4.1%) | 161 (12.3%) | 37 (3.3%) | 87 (9.7%) | 29 (3.1%) | 59 (8.2%) | 0 (0%) | 0 (0%) | 74 (5.2%) | 133 (10.4%) | 84 (4.3%) | 169 (12.3%) | 176 (7.8%) | 281 (15.2%) | 902 (4.9%) | 1749 (11.5%) |
| 2-3 days | 429 (24.5%) | 333 (16.9%) | 836 (18.0%) | 873 (24.0%) | 291 (14.3%) | 365 (25.4%) | 318 (21.5%) | 327 (24.9%) | 179 (15.9%) | 241 (26.8%) | 178 (18.9%) | 162 (22.4%) | 0 (0%) | 0 (0%) | 260 (18.4%) | 272 (21.3%) | 331 (16.9%) | 355 (25.9%) | 622 (27.7%) | 497 (27.0%) | 3444 (18.8%) | 3425 (22.5%) |
| 4-5 days | 271 (15.5%) | 182 (9.2%) | 876 (18.9%) | 436 (12.0%) | 358 (17.6%) | 203 (14.1%) | 249 (16.8%) | 181 (13.8%) | 206 (18.3%) | 120 (13.3%) | 185 (19.7%) | 73 (10.1%) | 0 (0%) | 0 (0%) | 234 (16.6%) | 134 (10.5%) | 368 (18.8%) | 203 (14.8%) | 433 (19.3%) | 205 (11.1%) | 3180 (17.3%) | 1737 (11.4%) |
| (Almost) every day | 700 (39.9%) | 442 (22.5%) | 2357 (50.7%) | 773 (21.3%) | 1207 (59.5%) | 374 (26.1%) | 777 (52.5%) | 369 (28.1%) | 668 (59.3%) | 297 (33.0%) | 478 (50.8%) | 94 (13.0%) | 0 (0%) | 0 (0%) | 720 (51.0%) | 420 (32.8%) | 1113 (57.0%) | 431 (31.4%) | 868 (38.6%) | 350 (19.0%) | 8888 (48.4%) | 3550 (23.3%) |
| Missing | 8 (0.5%) | 5 (0.3%) | 0 (0%) | 0 (0%) | 0 (0%) | 0 (0%) | 8 (0.5%) | 4 (0.3%) | 4 (0.4%) | 4 (0.4%) | 1 (0.1%) | 0 (0%) | 756 (100%) | 775 (100%) | 5 (0.4%) | 22 (1.7%) | 3 (0.2%) | 3 (0.2%) | 5 (0.2%) | 4 (0.2%) | 790 (4.3%) | 817 (5.4%) |
| Hours gaming /day |  |  |  |  |  |  |  |  |  |  |  |  |  |  |  |  |  |  |  |  |  |  |
| 1-2 hours | 734 (41.8%) | 1352 (68.7%) | 1781 (38.3%) | 2536 (69.9%) | 0 (0%) | 0 (0%) | 410 (27.7%) | 688 (52.4%) | 224 (19.9%) | 501 (55.7%) | 332 (35.3%) | 463 (64.1%) | 0 (0%) | 0 (0%) | 380 (26.9%) | 664 (51.9%) | 391 (20.0%) | 678 (49.4%) | 722 (32.1%) | 1154 (62.6%) | 4974 (27.1%) | 8036 (52.7%) |
| 2-4 hours | 588 (33.5%) | 345 (17.5%) | 1606 (34.6%) | 683 (18.8%) | 0 (0%) | 0 (0%) | 504 (34.0%) | 392 (29.9%) | 425 (37.7%) | 247 (27.4%) | 365 (38.8%) | 109 (15.1%) | 0 (0%) | 0 (0%) | 498 (35.3%) | 282 (22.0%) | 1030 (52.7%) | 495 (36.1%) | 1077 (47.9%) | 519 (28.1%) | 6093 (33.2%) | 3072 (20.2%) |
| 4-6 hours | 229 (13.1%) | 117 (5.9%) | 733 (15.8%) | 225 (6.2%) | 0 (0%) | 0 (0%) | 297 (20.1%) | 124 (9.4%) | 254 (22.6%) | 88 (9.8%) | 140 (14.9%) | 39 (5.4%) | 0 (0%) | 0 (0%) | 260 (18.4%) | 117 (9.1%) | 363 (18.6%) | 129 (9.4%) | 303 (13.5%) | 108 (5.9%) | 2579 (14.1%) | 947 (6.2%) |
| 6-8 hours | 91 (5.2%) | 43 (2.2%) | 264 (5.7%) | 75 (2.1%) | 0 (0%) | 0 (0%) | 121 (8.2%) | 42 (3.2%) | 116 (10.3%) | 20 (2.2%) | 57 (6.1%) | 2 (0.3%) | 0 (0%) | 0 (0%) | 125 (8.9%) | 45 (3.5%) | 92 (4.7%) | 38 (2.8%) | 78 (3.5%) | 30 (1.6%) | 944 (5.1%) | 295 (1.9%) |
| 8+ hours | 85 (4.8%) | 26 (1.3%) | 223 (4.8%) | 49 (1.3%) | 0 (0%) | 0 (0%) | 137 (9.3%) | 42 (3.2%) | 105 (9.3%) | 28 (3.1%) | 41 (4.4%) | 6 (0.8%) | 0 (0%) | 0 (0%) | 103 (7.3%) | 47 (3.7%) | 71 (3.6%) | 28 (2.0%) | 66 (2.9%) | 28 (1.5%) | 831 (4.5%) | 254 (1.7%) |
| Missing | 27 (1.5%) | 85 (4.3%) | 39 (0.8%) | 62 (1.7%) | 2030 (100%) | 1435 (100%) | 12 (0.8%) | 25 (1.9%) | 2 (0.2%) | 16 (1.8%) | 6 (0.6%) | 103 (14.3%) | 756 (100%) | 775 (100%) | 46 (3.3%) | 124 (9.7%) | 6 (0.3%) | 4 (0.3%) | 3 (0.1%) | 5 (0.3%) | 2927 (16.0%) | 2634 (17.3%) |
| eContact: Close friends |  |  |  |  |  |  |  |  |  |  |  |  |  |  |  |  |  |  |  |  |  |  |
| (Almost) never | 101 (5.8%) | 89 (4.5%) | 364 (7.8%) | 226 (6.2%) | 91 (4.5%) | 53 (3.7%) | 54 (3.6%) | 37 (2.8%) | 39 (3.5%) | 22 (2.4%) | 55 (5.8%) | 35 (4.8%) | 40 (5.3%) | 20 (2.6%) | 86 (6.1%) | 67 (5.2%) | 140 (7.2%) | 89 (6.5%) | 177 (7.9%) | 130 (7.0%) | 1147 (6.3%) | 768 (5.0%) |
| Almost all the time | 542 (30.9%) | 756 (38.4%) | 815 (17.5%) | 729 (20.1%) | 393 (19.4%) | 328 (22.9%) | 383 (25.9%) | 463 (35.3%) | 318 (28.2%) | 438 (48.7%) | 229 (24.3%) | 236 (32.7%) | 273 (36.1%) | 376 (48.5%) | 418 (29.6%) | 497 (38.9%) | 324 (16.6%) | 307 (22.4%) | 483 (21.5%) | 505 (27.4%) | 4178 (22.8%) | 4635 (30.4%) |
| At least weekly | 230 (13.1%) | 232 (11.8%) | 947 (20.4%) | 719 (19.8%) | 368 (18.1%) | 215 (15.0%) | 212 (14.3%) | 138 (10.5%) | 110 (9.8%) | 57 (6.3%) | 151 (16.0%) | 94 (13.0%) | 94 (12.4%) | 72 (9.3%) | 165 (11.7%) | 121 (9.5%) | 303 (15.5%) | 211 (15.4%) | 357 (15.9%) | 234 (12.7%) | 2937 (16.0%) | 2093 (13.7%) |
| Daily | 359 (20.5%) | 316 (16.1%) | 1321 (28.4%) | 1006 (27.7%) | 581 (28.6%) | 371 (25.9%) | 407 (27.5%) | 300 (22.8%) | 284 (25.2%) | 138 (15.3%) | 216 (23.0%) | 134 (18.6%) | 131 (17.3%) | 116 (15.0%) | 304 (21.5%) | 267 (20.9%) | 603 (30.9%) | 390 (28.4%) | 504 (22.4%) | 357 (19.4%) | 4710 (25.7%) | 3395 (22.3%) |
| Don't know | 70 (4.0%) | 49 (2.5%) | 223 (4.8%) | 152 (4.2%) | 80 (3.9%) | 37 (2.6%) | 42 (2.8%) | 25 (1.9%) | 13 (1.2%) | 10 (1.1%) | 54 (5.7%) | 29 (4.0%) | 37 (4.9%) | 21 (2.7%) | 58 (4.1%) | 25 (2.0%) | 46 (2.4%) | 19 (1.4%) | 58 (2.6%) | 49 (2.7%) | 681 (3.7%) | 416 (2.7%) |
| Several times daily | 442 (25.2%) | 520 (26.4%) | 954 (20.5%) | 777 (21.4%) | 512 (25.2%) | 431 (30.0%) | 376 (25.4%) | 344 (26.2%) | 356 (31.6%) | 232 (25.8%) | 230 (24.4%) | 190 (26.3%) | 180 (23.8%) | 170 (21.9%) | 373 (26.4%) | 293 (22.9%) | 525 (26.9%) | 352 (25.7%) | 655 (29.1%) | 560 (30.4%) | 4603 (25.1%) | 3869 (25.4%) |
| Missing | 10 (0.6%) | 6 (0.3%) | 22 (0.5%) | 21 (0.6%) | 5 (0.2%) | 0 (0%) | 7 (0.5%) | 6 (0.5%) | 6 (0.5%) | 3 (0.3%) | 6 (0.6%) | 4 (0.6%) | 1 (0.1%) | 0 (0%) | 8 (0.6%) | 9 (0.7%) | 12 (0.6%) | 4 (0.3%) | 15 (0.7%) | 9 (0.5%) | 92 (0.5%) | 62 (0.4%) |
| eContact: Larger friend group |  |  |  |  |  |  |  |  |  |  |  |  |  |  |  |  |  |  |  |  |  |  |
| (Almost) never | 224 (12.8%) | 281 (14.3%) | 988 (21.3%) | 862 (23.7%) | 243 (12.0%) | 215 (15.0%) | 188 (12.7%) | 223 (17.0%) | 143 (12.7%) | 124 (13.8%) | 146 (15.5%) | 122 (16.9%) | 93 (12.3%) | 114 (14.7%) | 230 (16.3%) | 221 (17.3%) | 365 (18.7%) | 288 (21.0%) | 400 (17.8%) | 366 (19.8%) | 3020 (16.5%) | 2816 (18.5%) |
| Almost all the time | 275 (15.7%) | 270 (13.7%) | 369 (7.9%) | 219 (6.0%) | 257 (12.7%) | 151 (10.5%) | 202 (13.6%) | 165 (12.6%) | 155 (13.8%) | 144 (16.0%) | 148 (15.7%) | 90 (12.5%) | 120 (15.9%) | 106 (13.7%) | 184 (13.0%) | 144 (11.3%) | 161 (8.2%) | 82 (6.0%) | 252 (11.2%) | 186 (10.1%) | 2123 (11.6%) | 1557 (10.2%) |
| At least weekly | 403 (23.0%) | 460 (23.4%) | 1419 (30.5%) | 1199 (33.0%) | 555 (27.3%) | 396 (27.6%) | 389 (26.3%) | 301 (22.9%) | 257 (22.8%) | 192 (21.3%) | 198 (21.0%) | 157 (21.7%) | 199 (26.3%) | 212 (27.4%) | 292 (20.7%) | 288 (22.5%) | 484 (24.8%) | 351 (25.6%) | 603 (26.8%) | 493 (26.7%) | 4799 (26.2%) | 4049 (26.6%) |
| Daily | 341 (19.4%) | 365 (18.5%) | 782 (16.8%) | 538 (14.8%) | 474 (23.3%) | 331 (23.1%) | 343 (23.2%) | 310 (23.6%) | 297 (26.4%) | 215 (23.9%) | 166 (17.6%) | 129 (17.9%) | 157 (20.8%) | 143 (18.5%) | 330 (23.4%) | 256 (20.0%) | 445 (22.8%) | 261 (19.0%) | 414 (18.4%) | 320 (17.4%) | 3749 (20.4%) | 2868 (18.8%) |
| Don't know | 117 (6.7%) | 162 (8.2%) | 580 (12.5%) | 432 (11.9%) | 144 (7.1%) | 112 (7.8%) | 94 (6.3%) | 96 (7.3%) | 51 (4.5%) | 41 (4.6%) | 106 (11.3%) | 82 (11.4%) | 28 (3.7%) | 54 (7.0%) | 108 (7.6%) | 122 (9.5%) | 180 (9.2%) | 192 (14.0%) | 145 (6.4%) | 124 (6.7%) | 1553 (8.5%) | 1417 (9.3%) |
| Several times daily | 369 (21.0%) | 407 (20.7%) | 424 (9.1%) | 307 (8.5%) | 347 (17.1%) | 218 (15.2%) | 249 (16.8%) | 196 (14.9%) | 217 (19.3%) | 179 (19.9%) | 161 (17.1%) | 131 (18.1%) | 159 (21.0%) | 145 (18.7%) | 259 (18.3%) | 233 (18.2%) | 293 (15.0%) | 189 (13.8%) | 404 (18.0%) | 332 (18.0%) | 2882 (15.7%) | 2337 (15.3%) |
| Missing | 25 (1.4%) | 23 (1.2%) | 84 (1.8%) | 73 (2.0%) | 10 (0.5%) | 12 (0.8%) | 16 (1.1%) | 22 (1.7%) | 6 (0.5%) | 5 (0.6%) | 16 (1.7%) | 11 (1.5%) | 0 (0%) | 1 (0.1%) | 9 (0.6%) | 15 (1.2%) | 25 (1.3%) | 9 (0.7%) | 31 (1.4%) | 23 (1.2%) | 222 (1.2%) | 194 (1.3%) |
| eContact: Online friends |  |  |  |  |  |  |  |  |  |  |  |  |  |  |  |  |  |  |  |  |  |  |
| (Almost) never | 405 (23.1%) | 397 (20.2%) | 1430 (30.8%) | 1121 (30.9%) | 459 (22.6%) | 307 (21.4%) | 390 (26.3%) | 298 (22.7%) | 336 (29.8%) | 244 (27.1%) | 232 (24.7%) | 131 (18.1%) | 222 (29.4%) | 232 (29.9%) | 384 (27.2%) | 359 (28.1%) | 532 (27.2%) | 319 (23.3%) | 574 (25.5%) | 467 (25.3%) | 4964 (27.1%) | 3875 (25.4%) |
| Almost all the time | 149 (8.5%) | 135 (6.9%) | 345 (7.4%) | 251 (6.9%) | 198 (9.8%) | 121 (8.4%) | 113 (7.6%) | 114 (8.7%) | 86 (7.6%) | 94 (10.4%) | 91 (9.7%) | 51 (7.1%) | 60 (7.9%) | 60 (7.7%) | 96 (6.8%) | 101 (7.9%) | 111 (5.7%) | 73 (5.3%) | 197 (8.8%) | 172 (9.3%) | 1446 (7.9%) | 1172 (7.7%) |
| At least weekly | 221 (12.6%) | 247 (12.6%) | 754 (16.2%) | 447 (12.3%) | 422 (20.8%) | 258 (18.0%) | 272 (18.4%) | 178 (13.6%) | 185 (16.4%) | 123 (13.7%) | 122 (13.0%) | 103 (14.3%) | 192 (25.4%) | 132 (17.0%) | 205 (14.5%) | 147 (11.5%) | 241 (12.3%) | 122 (8.9%) | 377 (16.8%) | 228 (12.4%) | 2991 (16.3%) | 1985 (13.0%) |
| Daily | 153 (8.7%) | 123 (6.3%) | 442 (9.5%) | 236 (6.5%) | 257 (12.7%) | 149 (10.4%) | 146 (9.9%) | 117 (8.9%) | 125 (11.1%) | 111 (12.3%) | 80 (8.5%) | 61 (8.4%) | 92 (12.2%) | 68 (8.8%) | 150 (10.6%) | 102 (8.0%) | 191 (9.8%) | 99 (7.2%) | 233 (10.4%) | 163 (8.8%) | 1869 (10.2%) | 1229 (8.1%) |
| Don't know | 690 (39.3%) | 935 (47.5%) | 1343 (28.9%) | 1361 (37.5%) | 487 (24.0%) | 467 (32.5%) | 458 (30.9%) | 509 (38.8%) | 298 (26.5%) | 250 (27.8%) | 334 (35.5%) | 316 (43.8%) | 114 (15.1%) | 230 (29.7%) | 452 (32.0%) | 469 (36.7%) | 723 (37.0%) | 679 (49.5%) | 617 (27.4%) | 613 (33.2%) | 5516 (30.1%) | 5829 (38.3%) |
| Several times daily | 126 (7.2%) | 116 (5.9%) | 311 (6.7%) | 197 (5.4%) | 193 (9.5%) | 128 (8.9%) | 99 (6.7%) | 88 (6.7%) | 91 (8.1%) | 73 (8.1%) | 72 (7.7%) | 55 (7.6%) | 76 (10.1%) | 53 (6.8%) | 118 (8.4%) | 92 (7.2%) | 134 (6.9%) | 73 (5.3%) | 216 (9.6%) | 170 (9.2%) | 1436 (7.8%) | 1045 (6.9%) |
| Missing | 10 (0.6%) | 15 (0.8%) | 21 (0.5%) | 17 (0.5%) | 14 (0.7%) | 5 (0.3%) | 3 (0.2%) | 9 (0.7%) | 5 (0.4%) | 5 (0.6%) | 10 (1.1%) | 5 (0.7%) | 0 (0%) | 0 (0%) | 7 (0.5%) | 9 (0.7%) | 21 (1.1%) | 7 (0.5%) | 35 (1.6%) | 31 (1.7%) | 126 (0.7%) | 103 (0.7%) |
| eContact: Other |  |  |  |  |  |  |  |  |  |  |  |  |  |  |  |  |  |  |  |  |  |  |
| (Almost) never | 293 (16.7%) | 317 (16.1%) | 1026 (22.1%) | 632 (17.4%) | 283 (13.9%) | 140 (9.8%) | 221 (14.9%) | 174 (13.3%) | 159 (14.1%) | 108 (12.0%) | 144 (15.3%) | 103 (14.3%) | 134 (17.7%) | 108 (13.9%) | 243 (17.2%) | 175 (13.7%) | 327 (16.7%) | 222 (16.2%) | 416 (18.5%) | 313 (17.0%) | 3246 (17.7%) | 2292 (15.0%) |
| Almost all the time | 351 (20.0%) | 377 (19.2%) | 395 (8.5%) | 322 (8.9%) | 230 (11.3%) | 177 (12.3%) | 227 (15.3%) | 208 (15.8%) | 147 (13.1%) | 175 (19.4%) | 129 (13.7%) | 98 (13.6%) | 146 (19.3%) | 164 (21.2%) | 269 (19.1%) | 270 (21.1%) | 247 (12.6%) | 159 (11.6%) | 393 (17.5%) | 302 (16.4%) | 2534 (13.8%) | 2252 (14.8%) |
| At least weekly | 364 (20.8%) | 426 (21.6%) | 1181 (25.4%) | 955 (26.3%) | 586 (28.9%) | 386 (26.9%) | 365 (24.6%) | 309 (23.5%) | 299 (26.6%) | 182 (20.2%) | 218 (23.2%) | 181 (25.1%) | 168 (22.2%) | 171 (22.1%) | 231 (16.4%) | 229 (17.9%) | 430 (22.0%) | 330 (24.1%) | 437 (19.4%) | 402 (21.8%) | 4279 (23.3%) | 3571 (23.4%) |
| Daily | 274 (15.6%) | 329 (16.7%) | 891 (19.2%) | 842 (23.2%) | 472 (23.3%) | 412 (28.7%) | 334 (22.6%) | 336 (25.6%) | 240 (21.3%) | 233 (25.9%) | 171 (18.2%) | 154 (21.3%) | 116 (15.3%) | 140 (18.1%) | 256 (18.1%) | 257 (20.1%) | 434 (22.2%) | 326 (23.8%) | 378 (16.8%) | 359 (19.5%) | 3566 (19.4%) | 3388 (22.2%) |
| Don't know | 172 (9.8%) | 124 (6.3%) | 636 (13.7%) | 363 (10.0%) | 180 (8.9%) | 53 (3.7%) | 128 (8.6%) | 65 (5.0%) | 90 (8.0%) | 39 (4.3%) | 115 (12.2%) | 61 (8.4%) | 73 (9.7%) | 65 (8.4%) | 168 (11.9%) | 121 (9.5%) | 179 (9.2%) | 91 (6.6%) | 208 (9.2%) | 102 (5.5%) | 1949 (10.6%) | 1084 (7.1%) |
| Several times daily | 286 (16.3%) | 381 (19.4%) | 485 (10.4%) | 501 (13.8%) | 270 (13.3%) | 262 (18.3%) | 201 (13.6%) | 216 (16.5%) | 188 (16.7%) | 161 (17.9%) | 152 (16.2%) | 122 (16.9%) | 118 (15.6%) | 127 (16.4%) | 237 (16.8%) | 217 (17.0%) | 321 (16.4%) | 240 (17.5%) | 371 (16.5%) | 345 (18.7%) | 2629 (14.3%) | 2572 (16.9%) |
| Missing | 14 (0.8%) | 14 (0.7%) | 32 (0.7%) | 15 (0.4%) | 9 (0.4%) | 5 (0.3%) | 5 (0.3%) | 5 (0.4%) | 3 (0.3%) | 2 (0.2%) | 12 (1.3%) | 3 (0.4%) | 1 (0.1%) | 0 (0%) | 8 (0.6%) | 10 (0.8%) | 15 (0.8%) | 4 (0.3%) | 46 (2.0%) | 21 (1.1%) | 145 (0.8%) | 79 (0.5%) |

## Figure 1. Social Media Use Frequency by Contact Type.

### Overall

### CY

### CZ

### EE

### GB-ENG

### IS

### MK

### MT

### NL

### GB-SCT

### SI

## Figure 2. Distribution of Symptoms Prevalence in Boys and Girls

### Overall

### CY

### CZ

### EE

### GB-ENG

### IS

### MK

### MT

### NL

### GB-SCT

### SI

## Table 2. Descriptive Statistics for Main Variables.

### Overall

Descriptive statistics and gender differences in total sample

|  | Boys (N=18348) | Girls (N=15238) | Overall (N=33586) | t | df | p | d |
| --- | --- | --- | --- | --- | --- | --- | --- |
| IGDS Preoccupation |  |  |  | 40.110 | 33572.370 | <0.001 | 0.433 |
| Mean (SD) | 0.426 (0.495) | 0.226 (0.418) | 0.335 (0.472) |  |  |  |  |
| Median [Min, Max] | 0 [0, 1.00] | 0 [0, 1.00] | 0 [0, 1.00] |  |  |  |  |
| IGDS Tolerance |  |  |  | 26.034 | 33552.295 | <0.001 | 0.281 |
| Mean (SD) | 0.325 (0.468) | 0.202 (0.401) | 0.269 (0.443) |  |  |  |  |
| Median [Min, Max] | 0 [0, 1.00] | 0 [0, 1.00] | 0 [0, 1.00] |  |  |  |  |
| IGDS Withdrawal |  |  |  | 21.029 | 33583.990 | <0.001 | 0.227 |
| Mean (SD) | 0.233 (0.423) | 0.144 (0.351) | 0.193 (0.394) |  |  |  |  |
| Median [Min, Max] | 0 [0, 1.00] | 0 [0, 1.00] | 0 [0, 1.00] |  |  |  |  |
| IGDS Persistence |  |  |  | 14.438 | 33417.059 | <0.001 | 0.157 |
| Mean (SD) | 0.238 (0.426) | 0.175 (0.380) | 0.209 (0.407) |  |  |  |  |
| Median [Min, Max] | 0 [0, 1.00] | 0 [0, 1.00] | 0 [0, 1.00] |  |  |  |  |
| IGDS Escape |  |  |  | 13.085 | 32630.642 | <0.001 | 0.143 |
| Mean (SD) | 0.483 (0.500) | 0.412 (0.492) | 0.451 (0.498) |  |  |  |  |
| Median [Min, Max] | 0 [0, 1.00] | 0 [0, 1.00] | 0 [0, 1.00] |  |  |  |  |
| IGDS Problems |  |  |  | 27.897 | 33281.078 | <0.001 | 0.298 |
| Mean (SD) | 0.220 (0.415) | 0.110 (0.312) | 0.170 (0.376) |  |  |  |  |
| Median [Min, Max] | 0 [0, 1.00] | 0 [0, 1.00] | 0 [0, 1.00] |  |  |  |  |
| IGDS Deception |  |  |  | 13.261 | 33563.563 | <0.001 | 0.143 |
| Mean (SD) | 0.154 (0.361) | 0.105 (0.307) | 0.132 (0.338) |  |  |  |  |
| Median [Min, Max] | 0 [0, 1.00] | 0 [0, 1.00] | 0 [0, 1.00] |  |  |  |  |
| IGDS Displacement |  |  |  | 11.538 | 33567.861 | <0.001 | 0.125 |
| Mean (SD) | 0.124 (0.330) | 0.0856 (0.280) | 0.107 (0.309) |  |  |  |  |
| Median [Min, Max] | 0 [0, 1.00] | 0 [0, 1.00] | 0 [0, 1.00] |  |  |  |  |
| IGDS Conflict |  |  |  | 17.014 | 33444.927 | <0.001 | 0.182 |
| Mean (SD) | 0.128 (0.334) | 0.0730 (0.260) | 0.103 (0.304) |  |  |  |  |
| Median [Min, Max] | 0 [0, 1.00] | 0 [0, 1.00] | 0 [0, 1.00] |  |  |  |  |
| SMDS Preoccupation |  |  |  | -13.747 | 31318.770 | <0.001 | 0.152 |
| Mean (SD) | 0.225 (0.418) | 0.291 (0.454) | 0.255 (0.436) |  |  |  |  |
| Median [Min, Max] | 0 [0, 1.00] | 0 [0, 1.00] | 0 [0, 1.00] |  |  |  |  |
| SMDS Tolerance |  |  |  | -14.015 | 30975.198 | <0.001 | 0.155 |
| Mean (SD) | 0.186 (0.389) | 0.250 (0.433) | 0.215 (0.411) |  |  |  |  |
| Median [Min, Max] | 0 [0, 1.00] | 0 [0, 1.00] | 0 [0, 1.00] |  |  |  |  |
| SMDS Withdrawal |  |  |  | -19.166 | 30544.631 | <0.001 | 0.213 |
| Mean (SD) | 0.192 (0.394) | 0.281 (0.450) | 0.233 (0.423) |  |  |  |  |
| Median [Min, Max] | 0 [0, 1.00] | 0 [0, 1.00] | 0 [0, 1.00] |  |  |  |  |
| SMDS Persistence |  |  |  | -32.159 | 31228.273 | <0.001 | 0.355 |
| Mean (SD) | 0.294 (0.456) | 0.463 (0.499) | 0.371 (0.483) |  |  |  |  |
| Median [Min, Max] | 0 [0, 1.00] | 0 [0, 1.00] | 0 [0, 1.00] |  |  |  |  |
| SMDS Escape |  |  |  | -34.115 | 32157.371 | <0.001 | 0.375 |
| Mean (SD) | 0.376 (0.484) | 0.559 (0.496) | 0.459 (0.498) |  |  |  |  |
| Median [Min, Max] | 0 [0, 1.00] | 1.00 [0, 1.00] | 0 [0, 1.00] |  |  |  |  |
| SMDS Problems |  |  |  | -11.616 | 31097.882 | <0.001 | 0.128 |
| Mean (SD) | 0.171 (0.376) | 0.222 (0.415) | 0.194 (0.395) |  |  |  |  |
| Median [Min, Max] | 0 [0, 1.00] | 0 [0, 1.00] | 0 [0, 1.00] |  |  |  |  |
| SMDS Deception |  |  |  | -12.726 | 30614.656 | <0.001 | 0.141 |
| Mean (SD) | 0.138 (0.345) | 0.190 (0.392) | 0.162 (0.368) |  |  |  |  |
| Median [Min, Max] | 0 [0, 1.00] | 0 [0, 1.00] | 0 [0, 1.00] |  |  |  |  |
| SMDS Displacement |  |  |  | -14.344 | 30624.319 | <0.001 | 0.159 |
| Mean (SD) | 0.157 (0.364) | 0.218 (0.413) | 0.185 (0.388) |  |  |  |  |
| Median [Min, Max] | 0 [0, 1.00] | 0 [0, 1.00] | 0 [0, 1.00] |  |  |  |  |
| SMDS Conflict |  |  |  | -14.447 | 30035.737 | <0.001 | 0.161 |
| Mean (SD) | 0.118 (0.322) | 0.174 (0.379) | 0.143 (0.350) |  |  |  |  |
| Median [Min, Max] | 0 [0, 1.00] | 0 [0, 1.00] | 0 [0, 1.00] |  |  |  |  |
| MHCS Headache |  |  |  | -41.097 | 29643.160 | <0.001 | 0.458 |
| Mean (SD) | 1.91 (1.13) | 2.48 (1.36) | 2.17 (1.27) |  |  |  |  |
| Median [Min, Max] | 2.00 [1.00, 5.00] | 2.00 [1.00, 5.00] | 2.00 [1.00, 5.00] |  |  |  |  |
| MHCS Stomach ache |  |  |  | -42.320 | 28927.059 | <0.001 | 0.473 |
| Mean (SD) | 1.62 (0.955) | 2.13 (1.20) | 1.85 (1.10) |  |  |  |  |
| Median [Min, Max] | 1.00 [1.00, 5.00] | 2.00 [1.00, 5.00] | 1.00 [1.00, 5.00] |  |  |  |  |
| MHCS Back pain |  |  |  | -19.531 | 30484.709 | <0.001 | 0.217 |
| Mean (SD) | 1.84 (1.20) | 2.12 (1.38) | 1.97 (1.29) |  |  |  |  |
| Median [Min, Max] | 1.00 [1.00, 5.00] | 2.00 [1.00, 5.00] | 1.00 [1.00, 5.00] |  |  |  |  |
| MHCS Feeling low |  |  |  | -49.509 | 30486.515 | <0.001 | 0.549 |
| Mean (SD) | 2.03 (1.28) | 2.78 (1.46) | 2.37 (1.41) |  |  |  |  |
| Median [Min, Max] | 2.00 [1.00, 5.00] | 3.00 [1.00, 5.00] | 2.00 [1.00, 5.00] |  |  |  |  |
| MHCS Irritability |  |  |  | -39.087 | 31818.434 | <0.001 | 0.430 |
| Mean (SD) | 2.52 (1.31) | 3.10 (1.37) | 2.78 (1.37) |  |  |  |  |
| Median [Min, Max] | 2.00 [1.00, 5.00] | 3.00 [1.00, 5.00] | 3.00 [1.00, 5.00] |  |  |  |  |
| MHCS Nervousness |  |  |  | -48.258 | 31576.474 | <0.001 | 0.532 |
| Mean (SD) | 2.51 (1.33) | 3.24 (1.43) | 2.84 (1.43) |  |  |  |  |
| Median [Min, Max] | 2.00 [1.00, 5.00] | 3.00 [1.00, 5.00] | 3.00 [1.00, 5.00] |  |  |  |  |
| MHCS Problems falling asleep |  |  |  | -28.679 | 31437.340 | <0.001 | 0.317 |
| Mean (SD) | 2.25 (1.46) | 2.73 (1.58) | 2.47 (1.53) |  |  |  |  |
| Median [Min, Max] | 2.00 [1.00, 5.00] | 2.00 [1.00, 5.00] | 2.00 [1.00, 5.00] |  |  |  |  |
| MHCS Dizziness |  |  |  | -37.219 | 27776.959 | <0.001 | 0.419 |
| Mean (SD) | 1.56 (1.04) | 2.07 (1.39) | 1.79 (1.24) |  |  |  |  |
| Median [Min, Max] | 1.00 [1.00, 5.00] | 1.00 [1.00, 5.00] | 1.00 [1.00, 5.00] |  |  |  |  |
| WHO-5 Wellbeing 1 |  |  |  | 31.597 | 31422.143 | <0.001 | 0.349 |
| Mean (SD) | 3.45 (1.19) | 3.02 (1.28) | 3.26 (1.25) |  |  |  |  |
| Median [Min, Max] | 4.00 [0, 5.00] | 3.00 [0, 5.00] | 4.00 [0, 5.00] |  |  |  |  |
| WHO-5 Wellbeing 2 |  |  |  | 42.270 | 31704.795 | <0.001 | 0.466 |
| Mean (SD) | 3.14 (1.32) | 2.51 (1.39) | 2.85 (1.39) |  |  |  |  |
| Median [Min, Max] | 3.00 [0, 5.00] | 3.00 [0, 5.00] | 3.00 [0, 5.00] |  |  |  |  |
| WHO-5 Wellbeing 3 |  |  |  | 39.741 | 31304.882 | <0.001 | 0.439 |
| Mean (SD) | 3.32 (1.35) | 2.70 (1.47) | 3.04 (1.44) |  |  |  |  |
| Median [Min, Max] | 4.00 [0, 5.00] | 3.00 [0, 5.00] | 3.00 [0, 5.00] |  |  |  |  |
| WHO-5 Wellbeing 4 |  |  |  | 33.205 | 32575.451 | <0.001 | 0.364 |
| Mean (SD) | 2.60 (1.60) | 2.02 (1.58) | 2.34 (1.62) |  |  |  |  |
| Median [Min, Max] | 3.00 [0, 5.00] | 2.00 [0, 5.00] | 2.00 [0, 5.00] |  |  |  |  |
| WHO-5 Wellbeing 5 |  |  |  | 31.163 | 31636.193 | <0.001 | 0.344 |
| Mean (SD) | 3.18 (1.42) | 2.68 (1.51) | 2.95 (1.48) |  |  |  |  |
| Median [Min, Max] | 3.00 [0, 5.00] | 3.00 [0, 5.00] | 3.00 [0, 5.00] |  |  |  |  |
| WHO-5 Overall Wellbeing (Averaged) |  |  |  | 44.802 | 31364.572 | <0.001 | 0.495 |
| Mean (SD) | 3.14 (1.07) | 2.59 (1.16) | 2.89 (1.15) |  |  |  |  |
| Median [Min, Max] | 3.20 [0, 5.00] | 2.60 [0, 5.00] | 3.00 [0, 5.00] |  |  |  |  |
| Loneliness |  |  |  | -49.045 | 31934.239 | <0.001 | 0.540 |
| Mean (SD) | 2.17 (1.04) | 2.74 (1.08) | 2.43 (1.10) |  |  |  |  |
| Median [Min, Max] | 2.00 [1.00, 5.00] | 3.00 [1.00, 5.00] | 2.00 [1.00, 5.00] |  |  |  |  |

### CY

Descriptive statistics and gender differences in CY sample

|  | Boys (N=1754) | Girls (N=1968) | Overall (N=3722) | t | df | p | d |
| --- | --- | --- | --- | --- | --- | --- | --- |
| IGDS Preoccupation |  |  |  | 13.748 | 3431.479 | <0.001 | 0.456 |
| Mean (SD) | 0.426 (0.495) | 0.219 (0.414) | 0.316 (0.465) |  |  |  |  |
| Median [Min, Max] | 0 [0, 1.00] | 0 [0, 1.00] | 0 [0, 1.00] |  |  |  |  |
| IGDS Tolerance |  |  |  | 8.422 | 3501.375 | <0.001 | 0.279 |
| Mean (SD) | 0.344 (0.475) | 0.220 (0.414) | 0.278 (0.448) |  |  |  |  |
| Median [Min, Max] | 0 [0, 1.00] | 0 [0, 1.00] | 0 [0, 1.00] |  |  |  |  |
| IGDS Withdrawal |  |  |  | 7.583 | 3420.880 | <0.001 | 0.252 |
| Mean (SD) | 0.257 (0.437) | 0.157 (0.363) | 0.204 (0.403) |  |  |  |  |
| Median [Min, Max] | 0 [0, 1.00] | 0 [0, 1.00] | 0 [0, 1.00] |  |  |  |  |
| IGDS Persistence |  |  |  | 5.805 | 3485.942 | <0.001 | 0.192 |
| Mean (SD) | 0.237 (0.425) | 0.161 (0.367) | 0.196 (0.397) |  |  |  |  |
| Median [Min, Max] | 0 [0, 1.00] | 0 [0, 1.00] | 0 [0, 1.00] |  |  |  |  |
| IGDS Escape |  |  |  | 6.414 | 3627.660 | <0.001 | 0.211 |
| Mean (SD) | 0.449 (0.498) | 0.346 (0.476) | 0.394 (0.489) |  |  |  |  |
| Median [Min, Max] | 0 [0, 1.00] | 0 [0, 1.00] | 0 [0, 1.00] |  |  |  |  |
| IGDS Problems |  |  |  | 8.801 | 3295.477 | <0.001 | 0.293 |
| Mean (SD) | 0.233 (0.423) | 0.123 (0.328) | 0.175 (0.380) |  |  |  |  |
| Median [Min, Max] | 0 [0, 1.00] | 0 [0, 1.00] | 0 [0, 1.00] |  |  |  |  |
| IGDS Deception |  |  |  | 5.996 | 3343.853 | <0.001 | 0.199 |
| Mean (SD) | 0.160 (0.366) | 0.0940 (0.292) | 0.125 (0.331) |  |  |  |  |
| Median [Min, Max] | 0 [0, 1.00] | 0 [0, 1.00] | 0 [0, 1.00] |  |  |  |  |
| IGDS Displacement |  |  |  | 3.747 | 3463.343 | <0.001 | 0.124 |
| Mean (SD) | 0.125 (0.331) | 0.0869 (0.282) | 0.105 (0.306) |  |  |  |  |
| Median [Min, Max] | 0 [0, 1.00] | 0 [0, 1.00] | 0 [0, 1.00] |  |  |  |  |
| IGDS Conflict |  |  |  | 6.721 | 3226.873 | <0.001 | 0.224 |
| Mean (SD) | 0.144 (0.351) | 0.0747 (0.263) | 0.107 (0.309) |  |  |  |  |
| Median [Min, Max] | 0 [0, 1.00] | 0 [0, 1.00] | 0 [0, 1.00] |  |  |  |  |
| SMDS Preoccupation |  |  |  | -5.252 | 3717.692 | <0.001 | 0.172 |
| Mean (SD) | 0.231 (0.422) | 0.307 (0.461) | 0.271 (0.445) |  |  |  |  |
| Median [Min, Max] | 0 [0, 1.00] | 0 [0, 1.00] | 0 [0, 1.00] |  |  |  |  |
| SMDS Tolerance |  |  |  | -3.786 | 3709.298 | <0.001 | 0.124 |
| Mean (SD) | 0.251 (0.434) | 0.306 (0.461) | 0.280 (0.449) |  |  |  |  |
| Median [Min, Max] | 0 [0, 1.00] | 0 [0, 1.00] | 0 [0, 1.00] |  |  |  |  |
| SMDS Withdrawal |  |  |  | -6.546 | 3717.928 | <0.001 | 0.214 |
| Mean (SD) | 0.256 (0.437) | 0.354 (0.478) | 0.308 (0.462) |  |  |  |  |
| Median [Min, Max] | 0 [0, 1.00] | 0 [0, 1.00] | 0 [0, 1.00] |  |  |  |  |
| SMDS Persistence |  |  |  | -7.204 | 3711.990 | <0.001 | 0.236 |
| Mean (SD) | 0.305 (0.461) | 0.418 (0.493) | 0.365 (0.481) |  |  |  |  |
| Median [Min, Max] | 0 [0, 1.00] | 0 [0, 1.00] | 0 [0, 1.00] |  |  |  |  |
| SMDS Escape |  |  |  | -8.313 | 3666.244 | <0.001 | 0.273 |
| Mean (SD) | 0.443 (0.497) | 0.578 (0.494) | 0.515 (0.500) |  |  |  |  |
| Median [Min, Max] | 0 [0, 1.00] | 1.00 [0, 1.00] | 1.00 [0, 1.00] |  |  |  |  |
| SMDS Problems |  |  |  | -4.009 | 3715.343 | <0.001 | 0.131 |
| Mean (SD) | 0.213 (0.410) | 0.269 (0.444) | 0.243 (0.429) |  |  |  |  |
| Median [Min, Max] | 0 [0, 1.00] | 0 [0, 1.00] | 0 [0, 1.00] |  |  |  |  |
| SMDS Deception |  |  |  | -3.925 | 3719.275 | <0.001 | 0.128 |
| Mean (SD) | 0.166 (0.373) | 0.217 (0.412) | 0.193 (0.395) |  |  |  |  |
| Median [Min, Max] | 0 [0, 1.00] | 0 [0, 1.00] | 0 [0, 1.00] |  |  |  |  |
| SMDS Displacement |  |  |  | -4.569 | 3719.583 | <0.001 | 0.149 |
| Mean (SD) | 0.184 (0.388) | 0.245 (0.430) | 0.217 (0.412) |  |  |  |  |
| Median [Min, Max] | 0 [0, 1.00] | 0 [0, 1.00] | 0 [0, 1.00] |  |  |  |  |
| SMDS Conflict |  |  |  | -5.916 | 3713.608 | <0.001 | 0.193 |
| Mean (SD) | 0.150 (0.357) | 0.225 (0.418) | 0.190 (0.392) |  |  |  |  |
| Median [Min, Max] | 0 [0, 1.00] | 0 [0, 1.00] | 0 [0, 1.00] |  |  |  |  |
| MHCS Headache |  |  |  | -14.217 | 3668.354 | <0.001 | 0.461 |
| Mean (SD) | 1.75 (1.06) | 2.30 (1.34) | 2.04 (1.24) |  |  |  |  |
| Median [Min, Max] | 1.00 [1.00, 5.00] | 2.00 [1.00, 5.00] | 2.00 [1.00, 5.00] |  |  |  |  |
| MHCS Stomach ache |  |  |  | -14.606 | 3659.078 | <0.001 | 0.473 |
| Mean (SD) | 1.55 (0.890) | 2.04 (1.14) | 1.81 (1.06) |  |  |  |  |
| Median [Min, Max] | 1.00 [1.00, 5.00] | 2.00 [1.00, 5.00] | 1.00 [1.00, 5.00] |  |  |  |  |
| MHCS Back pain |  |  |  | -7.803 | 3715.893 | <0.001 | 0.254 |
| Mean (SD) | 1.77 (1.17) | 2.09 (1.36) | 1.94 (1.28) |  |  |  |  |
| Median [Min, Max] | 1.00 [1.00, 5.00] | 2.00 [1.00, 5.00] | 1.00 [1.00, 5.00] |  |  |  |  |
| MHCS Feeling low |  |  |  | -10.298 | 3719.978 | <0.001 | 0.336 |
| Mean (SD) | 2.15 (1.30) | 2.61 (1.46) | 2.39 (1.40) |  |  |  |  |
| Median [Min, Max] | 2.00 [1.00, 5.00] | 2.00 [1.00, 5.00] | 2.00 [1.00, 5.00] |  |  |  |  |
| MHCS Irritability |  |  |  | -10.716 | 3695.056 | <0.001 | 0.351 |
| Mean (SD) | 2.69 (1.39) | 3.19 (1.44) | 2.96 (1.44) |  |  |  |  |
| Median [Min, Max] | 2.00 [1.00, 5.00] | 3.00 [1.00, 5.00] | 3.00 [1.00, 5.00] |  |  |  |  |
| MHCS Nervousness |  |  |  | -11.529 | 3699.163 | <0.001 | 0.378 |
| Mean (SD) | 2.61 (1.42) | 3.16 (1.48) | 2.90 (1.48) |  |  |  |  |
| Median [Min, Max] | 2.00 [1.00, 5.00] | 3.00 [1.00, 5.00] | 3.00 [1.00, 5.00] |  |  |  |  |
| MHCS Problems falling asleep |  |  |  | -7.956 | 3719.957 | <0.001 | 0.259 |
| Mean (SD) | 1.90 (1.33) | 2.26 (1.50) | 2.09 (1.44) |  |  |  |  |
| Median [Min, Max] | 1.00 [1.00, 5.00] | 2.00 [1.00, 5.00] | 1.00 [1.00, 5.00] |  |  |  |  |
| MHCS Dizziness |  |  |  | -12.901 | 3568.816 | <0.001 | 0.416 |
| Mean (SD) | 1.51 (0.944) | 1.99 (1.31) | 1.76 (1.18) |  |  |  |  |
| Median [Min, Max] | 1.00 [1.00, 5.00] | 1.00 [1.00, 5.00] | 1.00 [1.00, 5.00] |  |  |  |  |
| WHO-5 Wellbeing 1 |  |  |  | 11.237 | 3719.502 | <0.001 | 0.366 |
| Mean (SD) | 3.65 (1.18) | 3.18 (1.34) | 3.40 (1.29) |  |  |  |  |
| Median [Min, Max] | 4.00 [0, 5.00] | 4.00 [0, 5.00] | 4.00 [0, 5.00] |  |  |  |  |
| WHO-5 Wellbeing 2 |  |  |  | 16.145 | 3716.830 | <0.001 | 0.526 |
| Mean (SD) | 3.38 (1.30) | 2.64 (1.50) | 2.99 (1.45) |  |  |  |  |
| Median [Min, Max] | 4.00 [0, 5.00] | 3.00 [0, 5.00] | 3.00 [0, 5.00] |  |  |  |  |
| WHO-5 Wellbeing 3 |  |  |  | 12.281 | 3719.728 | <0.001 | 0.400 |
| Mean (SD) | 3.57 (1.35) | 2.99 (1.53) | 3.26 (1.47) |  |  |  |  |
| Median [Min, Max] | 4.00 [0, 5.00] | 3.00 [0, 5.00] | 4.00 [0, 5.00] |  |  |  |  |
| WHO-5 Wellbeing 4 |  |  |  | 9.791 | 3696.999 | <0.001 | 0.321 |
| Mean (SD) | 2.83 (1.63) | 2.29 (1.69) | 2.55 (1.68) |  |  |  |  |
| Median [Min, Max] | 3.00 [0, 5.00] | 2.00 [0, 5.00] | 3.00 [0, 5.00] |  |  |  |  |
| WHO-5 Wellbeing 5 |  |  |  | 7.861 | 3719.504 | <0.001 | 0.257 |
| Mean (SD) | 3.37 (1.41) | 2.99 (1.56) | 3.17 (1.50) |  |  |  |  |
| Median [Min, Max] | 4.00 [0, 5.00] | 3.00 [0, 5.00] | 3.00 [0, 5.00] |  |  |  |  |
| WHO-5 Overall Wellbeing (Averaged) |  |  |  | 14.535 | 3711.629 | <0.001 | 0.473 |
| Mean (SD) | 3.36 (1.04) | 2.82 (1.22) | 3.07 (1.17) |  |  |  |  |
| Median [Min, Max] | 3.40 [0, 5.00] | 2.80 [0, 5.00] | 3.20 [0, 5.00] |  |  |  |  |
| Loneliness |  |  |  | -14.007 | 3719.509 | <0.001 | 0.457 |
| Mean (SD) | 2.10 (1.03) | 2.60 (1.15) | 2.37 (1.12) |  |  |  |  |
| Median [Min, Max] | 2.00 [1.00, 5.00] | 3.00 [1.00, 5.00] | 2.00 [1.00, 5.00] |  |  |  |  |

### CZ

Descriptive statistics and gender differences in CZ sample

|  | Boys (N=4646) | Girls (N=3630) | Overall (N=8276) | t | df | p | d |
| --- | --- | --- | --- | --- | --- | --- | --- |
| IGDS Preoccupation |  |  |  | 20.188 | 8266.062 | <0.001 | 0.436 |
| Mean (SD) | 0.385 (0.487) | 0.190 (0.392) | 0.299 (0.458) |  |  |  |  |
| Median [Min, Max] | 0 [0, 1.00] | 0 [0, 1.00] | 0 [0, 1.00] |  |  |  |  |
| IGDS Tolerance |  |  |  | 13.725 | 8273.785 | <0.001 | 0.295 |
| Mean (SD) | 0.241 (0.428) | 0.127 (0.333) | 0.191 (0.393) |  |  |  |  |
| Median [Min, Max] | 0 [0, 1.00] | 0 [0, 1.00] | 0 [0, 1.00] |  |  |  |  |
| IGDS Withdrawal |  |  |  | 12.302 | 8256.787 | <0.001 | 0.263 |
| Mean (SD) | 0.183 (0.386) | 0.0915 (0.288) | 0.143 (0.350) |  |  |  |  |
| Median [Min, Max] | 0 [0, 1.00] | 0 [0, 1.00] | 0 [0, 1.00] |  |  |  |  |
| IGDS Persistence |  |  |  | 4.733 | 8057.285 | <0.001 | 0.104 |
| Mean (SD) | 0.209 (0.406) | 0.168 (0.374) | 0.191 (0.393) |  |  |  |  |
| Median [Min, Max] | 0 [0, 1.00] | 0 [0, 1.00] | 0 [0, 1.00] |  |  |  |  |
| IGDS Escape |  |  |  | 3.785 | 7808.532 | <0.001 | 0.084 |
| Mean (SD) | 0.501 (0.500) | 0.459 (0.498) | 0.483 (0.500) |  |  |  |  |
| Median [Min, Max] | 1.00 [0, 1.00] | 0 [0, 1.00] | 0 [0, 1.00] |  |  |  |  |
| IGDS Problems |  |  |  | 15.905 | 8180.757 | <0.001 | 0.338 |
| Mean (SD) | 0.211 (0.408) | 0.0898 (0.286) | 0.158 (0.365) |  |  |  |  |
| Median [Min, Max] | 0 [0, 1.00] | 0 [0, 1.00] | 0 [0, 1.00] |  |  |  |  |
| IGDS Deception |  |  |  | 7.066 | 8246.712 | <0.001 | 0.153 |
| Mean (SD) | 0.140 (0.347) | 0.0906 (0.287) | 0.118 (0.323) |  |  |  |  |
| Median [Min, Max] | 0 [0, 1.00] | 0 [0, 1.00] | 0 [0, 1.00] |  |  |  |  |
| IGDS Displacement |  |  |  | 6.721 | 8264.399 | <0.001 | 0.145 |
| Mean (SD) | 0.112 (0.316) | 0.0700 (0.255) | 0.0936 (0.291) |  |  |  |  |
| Median [Min, Max] | 0 [0, 1.00] | 0 [0, 1.00] | 0 [0, 1.00] |  |  |  |  |
| IGDS Conflict |  |  |  | 8.730 | 8271.556 | <0.001 | 0.187 |
| Mean (SD) | 0.126 (0.332) | 0.0700 (0.255) | 0.102 (0.302) |  |  |  |  |
| Median [Min, Max] | 0 [0, 1.00] | 0 [0, 1.00] | 0 [0, 1.00] |  |  |  |  |
| SMDS Preoccupation |  |  |  | -5.992 | 7451.364 | <0.001 | 0.134 |
| Mean (SD) | 0.201 (0.401) | 0.257 (0.437) | 0.225 (0.418) |  |  |  |  |
| Median [Min, Max] | 0 [0, 1.00] | 0 [0, 1.00] | 0 [0, 1.00] |  |  |  |  |
| SMDS Tolerance |  |  |  | -3.670 | 7522.786 | <0.001 | 0.082 |
| Mean (SD) | 0.160 (0.367) | 0.191 (0.393) | 0.174 (0.379) |  |  |  |  |
| Median [Min, Max] | 0 [0, 1.00] | 0 [0, 1.00] | 0 [0, 1.00] |  |  |  |  |
| SMDS Withdrawal |  |  |  | -6.404 | 7334.229 | <0.001 | 0.144 |
| Mean (SD) | 0.163 (0.369) | 0.218 (0.413) | 0.187 (0.390) |  |  |  |  |
| Median [Min, Max] | 0 [0, 1.00] | 0 [0, 1.00] | 0 [0, 1.00] |  |  |  |  |
| SMDS Persistence |  |  |  | -14.255 | 7395.760 | <0.001 | 0.320 |
| Mean (SD) | 0.278 (0.448) | 0.428 (0.495) | 0.344 (0.475) |  |  |  |  |
| Median [Min, Max] | 0 [0, 1.00] | 0 [0, 1.00] | 0 [0, 1.00] |  |  |  |  |
| SMDS Escape |  |  |  | -17.573 | 7646.032 | <0.001 | 0.391 |
| Mean (SD) | 0.355 (0.479) | 0.546 (0.498) | 0.439 (0.496) |  |  |  |  |
| Median [Min, Max] | 0 [0, 1.00] | 1.00 [0, 1.00] | 0 [0, 1.00] |  |  |  |  |
| SMDS Problems |  |  |  | -3.606 | 7516.577 | <0.001 | 0.081 |
| Mean (SD) | 0.153 (0.360) | 0.183 (0.387) | 0.167 (0.373) |  |  |  |  |
| Median [Min, Max] | 0 [0, 1.00] | 0 [0, 1.00] | 0 [0, 1.00] |  |  |  |  |
| SMDS Deception |  |  |  | -5.020 | 7331.171 | <0.001 | 0.113 |
| Mean (SD) | 0.125 (0.330) | 0.164 (0.370) | 0.142 (0.349) |  |  |  |  |
| Median [Min, Max] | 0 [0, 1.00] | 0 [0, 1.00] | 0 [0, 1.00] |  |  |  |  |
| SMDS Displacement |  |  |  | -4.552 | 7453.538 | <0.001 | 0.102 |
| Mean (SD) | 0.158 (0.365) | 0.196 (0.397) | 0.175 (0.380) |  |  |  |  |
| Median [Min, Max] | 0 [0, 1.00] | 0 [0, 1.00] | 0 [0, 1.00] |  |  |  |  |
| SMDS Conflict |  |  |  | -7.213 | 7102.609 | <0.001 | 0.163 |
| Mean (SD) | 0.117 (0.321) | 0.174 (0.379) | 0.142 (0.349) |  |  |  |  |
| Median [Min, Max] | 0 [0, 1.00] | 0 [0, 1.00] | 0 [0, 1.00] |  |  |  |  |
| MHCS Headache |  |  |  | -20.051 | 6922.358 | <0.001 | 0.455 |
| Mean (SD) | 1.85 (1.05) | 2.38 (1.28) | 2.08 (1.18) |  |  |  |  |
| Median [Min, Max] | 2.00 [1.00, 5.00] | 2.00 [1.00, 5.00] | 2.00 [1.00, 5.00] |  |  |  |  |
| MHCS Stomach ache |  |  |  | -16.375 | 6833.310 | <0.001 | 0.373 |
| Mean (SD) | 1.51 (0.872) | 1.87 (1.09) | 1.67 (0.990) |  |  |  |  |
| Median [Min, Max] | 1.00 [1.00, 5.00] | 2.00 [1.00, 5.00] | 1.00 [1.00, 5.00] |  |  |  |  |
| MHCS Back pain |  |  |  | -7.610 | 7352.169 | <0.001 | 0.171 |
| Mean (SD) | 1.92 (1.22) | 2.14 (1.37) | 2.02 (1.29) |  |  |  |  |
| Median [Min, Max] | 1.00 [1.00, 5.00] | 2.00 [1.00, 5.00] | 1.00 [1.00, 5.00] |  |  |  |  |
| MHCS Feeling low |  |  |  | -24.014 | 7082.170 | <0.001 | 0.543 |
| Mean (SD) | 1.95 (1.23) | 2.67 (1.45) | 2.26 (1.38) |  |  |  |  |
| Median [Min, Max] | 1.00 [1.00, 5.00] | 2.00 [1.00, 5.00] | 2.00 [1.00, 5.00] |  |  |  |  |
| MHCS Irritability |  |  |  | -19.580 | 7746.970 | <0.001 | 0.434 |
| Mean (SD) | 2.72 (1.26) | 3.27 (1.28) | 2.96 (1.30) |  |  |  |  |
| Median [Min, Max] | 3.00 [1.00, 5.00] | 3.00 [1.00, 5.00] | 3.00 [1.00, 5.00] |  |  |  |  |
| MHCS Nervousness |  |  |  | -21.244 | 7670.958 | <0.001 | 0.473 |
| Mean (SD) | 2.70 (1.32) | 3.34 (1.37) | 2.98 (1.38) |  |  |  |  |
| Median [Min, Max] | 3.00 [1.00, 5.00] | 4.00 [1.00, 5.00] | 3.00 [1.00, 5.00] |  |  |  |  |
| MHCS Problems falling asleep |  |  |  | -15.239 | 7479.537 | <0.001 | 0.341 |
| Mean (SD) | 2.12 (1.43) | 2.63 (1.55) | 2.34 (1.50) |  |  |  |  |
| Median [Min, Max] | 1.00 [1.00, 5.00] | 2.00 [1.00, 5.00] | 2.00 [1.00, 5.00] |  |  |  |  |
| MHCS Dizziness |  |  |  | -15.469 | 6129.317 | <0.001 | 0.358 |
| Mean (SD) | 1.30 (0.776) | 1.64 (1.13) | 1.45 (0.965) |  |  |  |  |
| Median [Min, Max] | 1.00 [1.00, 5.00] | 1.00 [1.00, 5.00] | 1.00 [1.00, 5.00] |  |  |  |  |
| WHO-5 Wellbeing 1 |  |  |  | 15.125 | 7542.260 | <0.001 | 0.338 |
| Mean (SD) | 3.41 (1.13) | 3.02 (1.20) | 3.24 (1.18) |  |  |  |  |
| Median [Min, Max] | 4.00 [0, 5.00] | 3.00 [0, 5.00] | 4.00 [0, 5.00] |  |  |  |  |
| WHO-5 Wellbeing 2 |  |  |  | 20.616 | 7644.309 | <0.001 | 0.459 |
| Mean (SD) | 3.02 (1.29) | 2.41 (1.34) | 2.75 (1.34) |  |  |  |  |
| Median [Min, Max] | 3.00 [0, 5.00] | 2.00 [0, 5.00] | 3.00 [0, 5.00] |  |  |  |  |
| WHO-5 Wellbeing 3 |  |  |  | 20.696 | 7558.513 | <0.001 | 0.462 |
| Mean (SD) | 3.25 (1.36) | 2.60 (1.44) | 2.96 (1.43) |  |  |  |  |
| Median [Min, Max] | 3.00 [0, 5.00] | 3.00 [0, 5.00] | 3.00 [0, 5.00] |  |  |  |  |
| WHO-5 Wellbeing 4 |  |  |  | 17.725 | 7978.894 | <0.001 | 0.390 |
| Mean (SD) | 2.38 (1.57) | 1.78 (1.48) | 2.12 (1.56) |  |  |  |  |
| Median [Min, Max] | 2.00 [0, 5.00] | 1.00 [0, 5.00] | 2.00 [0, 5.00] |  |  |  |  |
| WHO-5 Wellbeing 5 |  |  |  | 15.587 | 7676.466 | <0.001 | 0.347 |
| Mean (SD) | 3.03 (1.43) | 2.53 (1.47) | 2.81 (1.47) |  |  |  |  |
| Median [Min, Max] | 3.00 [0, 5.00] | 3.00 [0, 5.00] | 3.00 [0, 5.00] |  |  |  |  |
| WHO-5 Overall Wellbeing (Averaged) |  |  |  | 23.399 | 7611.566 | <0.001 | 0.521 |
| Mean (SD) | 3.02 (1.03) | 2.47 (1.08) | 2.78 (1.08) |  |  |  |  |
| Median [Min, Max] | 3.20 [0, 5.00] | 2.40 [0, 5.00] | 2.80 [0, 5.00] |  |  |  |  |
| Loneliness |  |  |  | -27.751 | 7790.157 | <0.001 | 0.615 |
| Mean (SD) | 2.20 (1.05) | 2.84 (1.05) | 2.48 (1.09) |  |  |  |  |
| Median [Min, Max] | 2.00 [1.00, 5.00] | 3.00 [1.00, 5.00] | 2.00 [1.00, 5.00] |  |  |  |  |

### EE

Descriptive statistics and gender differences in EE sample

|  | Boys (N=2030) | Girls (N=1435) | Overall (N=3465) | t | df | p | d |
| --- | --- | --- | --- | --- | --- | --- | --- |
| IGDS Preoccupation |  |  |  | 9.729 | 3274.221 | <0.001 | 0.329 |
| Mean (SD) | 0.425 (0.494) | 0.269 (0.444) | 0.360 (0.480) |  |  |  |  |
| Median [Min, Max] | 0 [0, 1.00] | 0 [0, 1.00] | 0 [0, 1.00] |  |  |  |  |
| IGDS Tolerance |  |  |  | 7.974 | 3258.740 | <0.001 | 0.270 |
| Mean (SD) | 0.396 (0.489) | 0.268 (0.443) | 0.343 (0.475) |  |  |  |  |
| Median [Min, Max] | 0 [0, 1.00] | 0 [0, 1.00] | 0 [0, 1.00] |  |  |  |  |
| IGDS Withdrawal |  |  |  | 3.229 | 3204.240 | 0.001 | 0.110 |
| Mean (SD) | 0.271 (0.445) | 0.224 (0.417) | 0.252 (0.434) |  |  |  |  |
| Median [Min, Max] | 0 [0, 1.00] | 0 [0, 1.00] | 0 [0, 1.00] |  |  |  |  |
| IGDS Persistence |  |  |  | 4.579 | 3268.642 | <0.001 | 0.155 |
| Mean (SD) | 0.255 (0.436) | 0.190 (0.393) | 0.228 (0.420) |  |  |  |  |
| Median [Min, Max] | 0 [0, 1.00] | 0 [0, 1.00] | 0 [0, 1.00] |  |  |  |  |
| IGDS Escape |  |  |  | 4.738 | 3121.033 | <0.001 | 0.163 |
| Mean (SD) | 0.486 (0.500) | 0.405 (0.491) | 0.452 (0.498) |  |  |  |  |
| Median [Min, Max] | 0 [0, 1.00] | 0 [0, 1.00] | 0 [0, 1.00] |  |  |  |  |
| IGDS Problems |  |  |  | 8.097 | 3419.162 | <0.001 | 0.268 |
| Mean (SD) | 0.233 (0.423) | 0.128 (0.334) | 0.189 (0.392) |  |  |  |  |
| Median [Min, Max] | 0 [0, 1.00] | 0 [0, 1.00] | 0 [0, 1.00] |  |  |  |  |
| IGDS Deception |  |  |  | 3.849 | 3294.162 | <0.001 | 0.130 |
| Mean (SD) | 0.185 (0.388) | 0.137 (0.344) | 0.165 (0.371) |  |  |  |  |
| Median [Min, Max] | 0 [0, 1.00] | 0 [0, 1.00] | 0 [0, 1.00] |  |  |  |  |
| IGDS Displacement |  |  |  | 4.854 | 3404.775 | <0.001 | 0.161 |
| Mean (SD) | 0.130 (0.336) | 0.0794 (0.271) | 0.109 (0.311) |  |  |  |  |
| Median [Min, Max] | 0 [0, 1.00] | 0 [0, 1.00] | 0 [0, 1.00] |  |  |  |  |
| IGDS Conflict |  |  |  | 4.871 | 3377.712 | <0.001 | 0.163 |
| Mean (SD) | 0.154 (0.361) | 0.0990 (0.299) | 0.131 (0.337) |  |  |  |  |
| Median [Min, Max] | 0 [0, 1.00] | 0 [0, 1.00] | 0 [0, 1.00] |  |  |  |  |
| SMDS Preoccupation |  |  |  | -4.353 | 2908.865 | <0.001 | 0.152 |
| Mean (SD) | 0.212 (0.409) | 0.277 (0.448) | 0.239 (0.426) |  |  |  |  |
| Median [Min, Max] | 0 [0, 1.00] | 0 [0, 1.00] | 0 [0, 1.00] |  |  |  |  |
| SMDS Tolerance |  |  |  | -4.294 | 2912.425 | <0.001 | 0.150 |
| Mean (SD) | 0.213 (0.410) | 0.277 (0.448) | 0.240 (0.427) |  |  |  |  |
| Median [Min, Max] | 0 [0, 1.00] | 0 [0, 1.00] | 0 [0, 1.00] |  |  |  |  |
| SMDS Withdrawal |  |  |  | -5.504 | 2808.043 | <0.001 | 0.194 |
| Mean (SD) | 0.168 (0.374) | 0.246 (0.431) | 0.201 (0.400) |  |  |  |  |
| Median [Min, Max] | 0 [0, 1.00] | 0 [0, 1.00] | 0 [0, 1.00] |  |  |  |  |
| SMDS Persistence |  |  |  | -11.041 | 2841.838 | <0.001 | 0.389 |
| Mean (SD) | 0.260 (0.439) | 0.440 (0.497) | 0.335 (0.472) |  |  |  |  |
| Median [Min, Max] | 0 [0, 1.00] | 0 [0, 1.00] | 0 [0, 1.00] |  |  |  |  |
| SMDS Escape |  |  |  | -9.229 | 2985.603 | <0.001 | 0.321 |
| Mean (SD) | 0.342 (0.475) | 0.498 (0.500) | 0.407 (0.491) |  |  |  |  |
| Median [Min, Max] | 0 [0, 1.00] | 0 [0, 1.00] | 0 [0, 1.00] |  |  |  |  |
| SMDS Problems |  |  |  | -2.155 | 2950.100 | 0.031 | 0.075 |
| Mean (SD) | 0.145 (0.352) | 0.172 (0.378) | 0.156 (0.363) |  |  |  |  |
| Median [Min, Max] | 0 [0, 1.00] | 0 [0, 1.00] | 0 [0, 1.00] |  |  |  |  |
| SMDS Deception |  |  |  | -3.011 | 2875.951 | 0.003 | 0.106 |
| Mean (SD) | 0.126 (0.331) | 0.162 (0.369) | 0.141 (0.348) |  |  |  |  |
| Median [Min, Max] | 0 [0, 1.00] | 0 [0, 1.00] | 0 [0, 1.00] |  |  |  |  |
| SMDS Displacement |  |  |  | -0.461 | 3055.550 | 0.645 | 0.016 |
| Mean (SD) | 0.136 (0.343) | 0.141 (0.349) | 0.138 (0.345) |  |  |  |  |
| Median [Min, Max] | 0 [0, 1.00] | 0 [0, 1.00] | 0 [0, 1.00] |  |  |  |  |
| SMDS Conflict |  |  |  | -3.983 | 2797.798 | <0.001 | 0.141 |
| Mean (SD) | 0.116 (0.321) | 0.164 (0.371) | 0.136 (0.343) |  |  |  |  |
| Median [Min, Max] | 0 [0, 1.00] | 0 [0, 1.00] | 0 [0, 1.00] |  |  |  |  |
| MHCS Headache |  |  |  | -10.910 | 2819.305 | <0.001 | 0.385 |
| Mean (SD) | 2.00 (1.23) | 2.51 (1.41) | 2.21 (1.33) |  |  |  |  |
| Median [Min, Max] | 2.00 [1.00, 5.00] | 2.00 [1.00, 5.00] | 2.00 [1.00, 5.00] |  |  |  |  |
| MHCS Stomach ache |  |  |  | -13.280 | 2689.787 | <0.001 | 0.474 |
| Mean (SD) | 1.72 (0.995) | 2.23 (1.21) | 1.93 (1.12) |  |  |  |  |
| Median [Min, Max] | 1.00 [1.00, 5.00] | 2.00 [1.00, 5.00] | 2.00 [1.00, 5.00] |  |  |  |  |
| MHCS Back pain |  |  |  | -4.118 | 2889.678 | <0.001 | 0.144 |
| Mean (SD) | 1.90 (1.26) | 2.09 (1.39) | 1.98 (1.32) |  |  |  |  |
| Median [Min, Max] | 1.00 [1.00, 5.00] | 1.00 [1.00, 5.00] | 1.00 [1.00, 5.00] |  |  |  |  |
| MHCS Feeling low |  |  |  | -16.623 | 2836.131 | <0.001 | 0.586 |
| Mean (SD) | 2.05 (1.33) | 2.88 (1.51) | 2.40 (1.47) |  |  |  |  |
| Median [Min, Max] | 1.00 [1.00, 5.00] | 3.00 [1.00, 5.00] | 2.00 [1.00, 5.00] |  |  |  |  |
| MHCS Irritability |  |  |  | -13.983 | 2964.748 | <0.001 | 0.487 |
| Mean (SD) | 2.51 (1.31) | 3.16 (1.40) | 2.78 (1.39) |  |  |  |  |
| Median [Min, Max] | 2.00 [1.00, 5.00] | 3.00 [1.00, 5.00] | 3.00 [1.00, 5.00] |  |  |  |  |
| MHCS Nervousness |  |  |  | -12.900 | 2868.450 | <0.001 | 0.453 |
| Mean (SD) | 2.46 (1.37) | 3.11 (1.53) | 2.73 (1.47) |  |  |  |  |
| Median [Min, Max] | 2.00 [1.00, 5.00] | 3.00 [1.00, 5.00] | 3.00 [1.00, 5.00] |  |  |  |  |
| MHCS Problems falling asleep |  |  |  | -7.780 | 2917.255 | <0.001 | 0.272 |
| Mean (SD) | 2.31 (1.50) | 2.74 (1.64) | 2.49 (1.57) |  |  |  |  |
| Median [Min, Max] | 2.00 [1.00, 5.00] | 2.00 [1.00, 5.00] | 2.00 [1.00, 5.00] |  |  |  |  |
| MHCS Dizziness |  |  |  | -10.075 | 2716.362 | <0.001 | 0.359 |
| Mean (SD) | 1.87 (1.28) | 2.37 (1.55) | 2.08 (1.42) |  |  |  |  |
| Median [Min, Max] | 1.00 [1.00, 5.00] | 2.00 [1.00, 5.00] | 1.00 [1.00, 5.00] |  |  |  |  |
| WHO-5 Wellbeing 1 |  |  |  | 8.377 | 2990.212 | <0.001 | 0.291 |
| Mean (SD) | 3.45 (1.22) | 3.09 (1.28) | 3.30 (1.26) |  |  |  |  |
| Median [Min, Max] | 4.00 [0, 5.00] | 3.00 [0, 5.00] | 4.00 [0, 5.00] |  |  |  |  |
| WHO-5 Wellbeing 2 |  |  |  | 12.120 | 2979.295 | <0.001 | 0.422 |
| Mean (SD) | 3.06 (1.37) | 2.47 (1.45) | 2.82 (1.43) |  |  |  |  |
| Median [Min, Max] | 3.00 [0, 5.00] | 2.00 [0, 5.00] | 3.00 [0, 5.00] |  |  |  |  |
| WHO-5 Wellbeing 3 |  |  |  | 10.529 | 2904.170 | <0.001 | 0.369 |
| Mean (SD) | 3.25 (1.36) | 2.72 (1.50) | 3.03 (1.44) |  |  |  |  |
| Median [Min, Max] | 3.00 [0, 5.00] | 3.00 [0, 5.00] | 3.00 [0, 5.00] |  |  |  |  |
| WHO-5 Wellbeing 4 |  |  |  | 9.422 | 3093.835 | <0.001 | 0.325 |
| Mean (SD) | 2.50 (1.57) | 1.99 (1.57) | 2.29 (1.59) |  |  |  |  |
| Median [Min, Max] | 3.00 [0, 5.00] | 2.00 [0, 5.00] | 2.00 [0, 5.00] |  |  |  |  |
| WHO-5 Wellbeing 5 |  |  |  | 8.258 | 2931.270 | <0.001 | 0.289 |
| Mean (SD) | 3.19 (1.41) | 2.77 (1.52) | 3.02 (1.47) |  |  |  |  |
| Median [Min, Max] | 4.00 [0, 5.00] | 3.00 [0, 5.00] | 3.00 [0, 5.00] |  |  |  |  |
| WHO-5 Overall Wellbeing (Averaged) |  |  |  | 11.938 | 2925.721 | <0.001 | 0.418 |
| Mean (SD) | 3.09 (1.11) | 2.61 (1.21) | 2.89 (1.18) |  |  |  |  |
| Median [Min, Max] | 3.20 [0, 5.00] | 2.60 [0, 5.00] | 3.00 [0, 5.00] |  |  |  |  |
| Loneliness |  |  |  | -14.466 | 3008.529 | <0.001 | 0.502 |
| Mean (SD) | 2.20 (1.05) | 2.74 (1.09) | 2.42 (1.10) |  |  |  |  |
| Median [Min, Max] | 2.00 [1.00, 5.00] | 3.00 [1.00, 5.00] | 2.00 [1.00, 5.00] |  |  |  |  |

### GB-ENG

Descriptive statistics and gender differences in GB-ENG sample

|  | Boys (N=1481) | Girls (N=1313) | Overall (N=2794) | t | df | p | d |
| --- | --- | --- | --- | --- | --- | --- | --- |
| IGDS Preoccupation |  |  |  | 13.243 | 2782.721 | <0.001 | 0.500 |
| Mean (SD) | 0.560 (0.497) | 0.319 (0.466) | 0.447 (0.497) |  |  |  |  |
| Median [Min, Max] | 1.00 [0, 1.00] | 0 [0, 1.00] | 0 [0, 1.00] |  |  |  |  |
| IGDS Tolerance |  |  |  | 7.705 | 2776.908 | <0.001 | 0.291 |
| Mean (SD) | 0.493 (0.500) | 0.350 (0.477) | 0.426 (0.495) |  |  |  |  |
| Median [Min, Max] | 0 [0, 1.00] | 0 [0, 1.00] | 0 [0, 1.00] |  |  |  |  |
| IGDS Withdrawal |  |  |  | 5.550 | 2791.845 | <0.001 | 0.209 |
| Mean (SD) | 0.311 (0.463) | 0.219 (0.413) | 0.267 (0.443) |  |  |  |  |
| Median [Min, Max] | 0 [0, 1.00] | 0 [0, 1.00] | 0 [0, 1.00] |  |  |  |  |
| IGDS Persistence |  |  |  | 1.082 | 2764.652 | 0.279 | 0.041 |
| Mean (SD) | 0.280 (0.449) | 0.262 (0.440) | 0.272 (0.445) |  |  |  |  |
| Median [Min, Max] | 0 [0, 1.00] | 0 [0, 1.00] | 0 [0, 1.00] |  |  |  |  |
| IGDS Escape |  |  |  | 0.546 | 2750.763 | 0.585 | 0.021 |
| Mean (SD) | 0.550 (0.498) | 0.540 (0.499) | 0.545 (0.498) |  |  |  |  |
| Median [Min, Max] | 1.00 [0, 1.00] | 1.00 [0, 1.00] | 1.00 [0, 1.00] |  |  |  |  |
| IGDS Problems |  |  |  | 8.312 | 2741.026 | <0.001 | 0.310 |
| Mean (SD) | 0.248 (0.432) | 0.127 (0.333) | 0.191 (0.393) |  |  |  |  |
| Median [Min, Max] | 0 [0, 1.00] | 0 [0, 1.00] | 0 [0, 1.00] |  |  |  |  |
| IGDS Deception |  |  |  | 1.491 | 2778.559 | 0.136 | 0.056 |
| Mean (SD) | 0.176 (0.381) | 0.155 (0.362) | 0.166 (0.372) |  |  |  |  |
| Median [Min, Max] | 0 [0, 1.00] | 0 [0, 1.00] | 0 [0, 1.00] |  |  |  |  |
| IGDS Displacement |  |  |  | 2.370 | 2789.640 | 0.018 | 0.089 |
| Mean (SD) | 0.159 (0.366) | 0.128 (0.334) | 0.145 (0.352) |  |  |  |  |
| Median [Min, Max] | 0 [0, 1.00] | 0 [0, 1.00] | 0 [0, 1.00] |  |  |  |  |
| IGDS Conflict |  |  |  | 3.695 | 2786.372 | <0.001 | 0.139 |
| Mean (SD) | 0.142 (0.349) | 0.0967 (0.296) | 0.121 (0.326) |  |  |  |  |
| Median [Min, Max] | 0 [0, 1.00] | 0 [0, 1.00] | 0 [0, 1.00] |  |  |  |  |
| SMDS Preoccupation |  |  |  | -5.988 | 2646.592 | <0.001 | 0.229 |
| Mean (SD) | 0.226 (0.418) | 0.327 (0.469) | 0.273 (0.446) |  |  |  |  |
| Median [Min, Max] | 0 [0, 1.00] | 0 [0, 1.00] | 0 [0, 1.00] |  |  |  |  |
| SMDS Tolerance |  |  |  | -6.678 | 2621.322 | <0.001 | 0.255 |
| Mean (SD) | 0.210 (0.407) | 0.321 (0.467) | 0.262 (0.440) |  |  |  |  |
| Median [Min, Max] | 0 [0, 1.00] | 0 [0, 1.00] | 0 [0, 1.00] |  |  |  |  |
| SMDS Withdrawal |  |  |  | -7.144 | 2621.705 | <0.001 | 0.273 |
| Mean (SD) | 0.218 (0.413) | 0.339 (0.474) | 0.275 (0.447) |  |  |  |  |
| Median [Min, Max] | 0 [0, 1.00] | 0 [0, 1.00] | 0 [0, 1.00] |  |  |  |  |
| SMDS Persistence |  |  |  | -14.265 | 2706.174 | <0.001 | 0.543 |
| Mean (SD) | 0.319 (0.466) | 0.579 (0.494) | 0.441 (0.497) |  |  |  |  |
| Median [Min, Max] | 0 [0, 1.00] | 1.00 [0, 1.00] | 0 [0, 1.00] |  |  |  |  |
| SMDS Escape |  |  |  | -14.406 | 2777.699 | <0.001 | 0.544 |
| Mean (SD) | 0.412 (0.492) | 0.674 (0.469) | 0.535 (0.499) |  |  |  |  |
| Median [Min, Max] | 0 [0, 1.00] | 1.00 [0, 1.00] | 1.00 [0, 1.00] |  |  |  |  |
| SMDS Problems |  |  |  | -4.420 | 2648.249 | <0.001 | 0.169 |
| Mean (SD) | 0.186 (0.390) | 0.256 (0.437) | 0.219 (0.414) |  |  |  |  |
| Median [Min, Max] | 0 [0, 1.00] | 0 [0, 1.00] | 0 [0, 1.00] |  |  |  |  |
| SMDS Deception |  |  |  | -7.872 | 2515.481 | <0.001 | 0.302 |
| Mean (SD) | 0.151 (0.358) | 0.273 (0.445) | 0.208 (0.406) |  |  |  |  |
| Median [Min, Max] | 0 [0, 1.00] | 0 [0, 1.00] | 0 [0, 1.00] |  |  |  |  |
| SMDS Displacement |  |  |  | -7.488 | 2565.168 | <0.001 | 0.287 |
| Mean (SD) | 0.176 (0.381) | 0.296 (0.457) | 0.233 (0.423) |  |  |  |  |
| Median [Min, Max] | 0 [0, 1.00] | 0 [0, 1.00] | 0 [0, 1.00] |  |  |  |  |
| SMDS Conflict |  |  |  | -5.448 | 2515.245 | <0.001 | 0.209 |
| Mean (SD) | 0.106 (0.308) | 0.178 (0.383) | 0.140 (0.347) |  |  |  |  |
| Median [Min, Max] | 0 [0, 1.00] | 0 [0, 1.00] | 0 [0, 1.00] |  |  |  |  |
| MHCS Headache |  |  |  | -14.256 | 2588.338 | <0.001 | 0.546 |
| Mean (SD) | 2.11 (1.15) | 2.79 (1.35) | 2.43 (1.29) |  |  |  |  |
| Median [Min, Max] | 2.00 [1.00, 5.00] | 3.00 [1.00, 5.00] | 2.00 [1.00, 5.00] |  |  |  |  |
| MHCS Stomach ache |  |  |  | -15.448 | 2546.011 | <0.001 | 0.592 |
| Mean (SD) | 1.81 (1.03) | 2.49 (1.25) | 2.13 (1.19) |  |  |  |  |
| Median [Min, Max] | 2.00 [1.00, 5.00] | 2.00 [1.00, 5.00] | 2.00 [1.00, 5.00] |  |  |  |  |
| MHCS Back pain |  |  |  | -7.574 | 2603.359 | <0.001 | 0.290 |
| Mean (SD) | 1.81 (1.21) | 2.19 (1.41) | 1.99 (1.33) |  |  |  |  |
| Median [Min, Max] | 1.00 [1.00, 5.00] | 2.00 [1.00, 5.00] | 1.00 [1.00, 5.00] |  |  |  |  |
| MHCS Feeling low |  |  |  | -16.751 | 2731.561 | <0.001 | 0.636 |
| Mean (SD) | 2.36 (1.36) | 3.23 (1.40) | 2.77 (1.45) |  |  |  |  |
| Median [Min, Max] | 2.00 [1.00, 5.00] | 3.00 [1.00, 5.00] | 3.00 [1.00, 5.00] |  |  |  |  |
| MHCS Irritability |  |  |  | -12.541 | 2745.551 | <0.001 | 0.476 |
| Mean (SD) | 2.59 (1.38) | 3.25 (1.40) | 2.90 (1.43) |  |  |  |  |
| Median [Min, Max] | 2.00 [1.00, 5.00] | 3.00 [1.00, 5.00] | 3.00 [1.00, 5.00] |  |  |  |  |
| MHCS Nervousness |  |  |  | -20.584 | 2768.898 | <0.001 | 0.779 |
| Mean (SD) | 2.57 (1.38) | 3.63 (1.34) | 3.07 (1.46) |  |  |  |  |
| Median [Min, Max] | 2.00 [1.00, 5.00] | 4.00 [1.00, 5.00] | 3.00 [1.00, 5.00] |  |  |  |  |
| MHCS Problems falling asleep |  |  |  | -9.911 | 2757.214 | <0.001 | 0.376 |
| Mean (SD) | 2.67 (1.56) | 3.25 (1.55) | 2.94 (1.58) |  |  |  |  |
| Median [Min, Max] | 2.00 [1.00, 5.00] | 4.00 [1.00, 5.00] | 3.00 [1.00, 5.00] |  |  |  |  |
| MHCS Dizziness |  |  |  | -16.296 | 2455.545 | <0.001 | 0.627 |
| Mean (SD) | 1.76 (1.16) | 2.59 (1.50) | 2.15 (1.39) |  |  |  |  |
| Median [Min, Max] | 1.00 [1.00, 5.00] | 2.00 [1.00, 5.00] | 2.00 [1.00, 5.00] |  |  |  |  |
| WHO-5 Wellbeing 1 |  |  |  | 11.207 | 2700.825 | <0.001 | 0.426 |
| Mean (SD) | 3.22 (1.21) | 2.68 (1.29) | 2.97 (1.28) |  |  |  |  |
| Median [Min, Max] | 4.00 [0, 5.00] | 3.00 [0, 5.00] | 3.00 [0, 5.00] |  |  |  |  |
| WHO-5 Wellbeing 2 |  |  |  | 14.816 | 2711.018 | <0.001 | 0.563 |
| Mean (SD) | 3.08 (1.26) | 2.35 (1.33) | 2.74 (1.35) |  |  |  |  |
| Median [Min, Max] | 3.00 [0, 5.00] | 2.00 [0, 5.00] | 3.00 [0, 5.00] |  |  |  |  |
| WHO-5 Wellbeing 3 |  |  |  | 15.105 | 2705.046 | <0.001 | 0.575 |
| Mean (SD) | 3.11 (1.31) | 2.34 (1.39) | 2.75 (1.40) |  |  |  |  |
| Median [Min, Max] | 3.00 [0, 5.00] | 2.00 [0, 5.00] | 3.00 [0, 5.00] |  |  |  |  |
| WHO-5 Wellbeing 4 |  |  |  | 13.304 | 2755.731 | <0.001 | 0.504 |
| Mean (SD) | 2.56 (1.53) | 1.79 (1.52) | 2.20 (1.57) |  |  |  |  |
| Median [Min, Max] | 3.00 [0, 5.00] | 2.00 [0, 5.00] | 2.00 [0, 5.00] |  |  |  |  |
| WHO-5 Wellbeing 5 |  |  |  | 11.212 | 2733.103 | <0.001 | 0.426 |
| Mean (SD) | 3.05 (1.44) | 2.43 (1.47) | 2.76 (1.49) |  |  |  |  |
| Median [Min, Max] | 3.00 [0, 5.00] | 2.00 [0, 5.00] | 3.00 [0, 5.00] |  |  |  |  |
| WHO-5 Overall Wellbeing (Averaged) |  |  |  | 16.409 | 2716.125 | <0.001 | 0.624 |
| Mean (SD) | 3.00 (1.07) | 2.32 (1.13) | 2.68 (1.15) |  |  |  |  |
| Median [Min, Max] | 3.20 [0, 5.00] | 2.40 [0, 5.00] | 2.80 [0, 5.00] |  |  |  |  |
| Loneliness |  |  |  | -15.396 | 2779.607 | <0.001 | 0.582 |
| Mean (SD) | 2.43 (1.09) | 3.05 (1.03) | 2.72 (1.10) |  |  |  |  |
| Median [Min, Max] | 2.00 [1.00, 5.00] | 3.00 [1.00, 5.00] | 3.00 [1.00, 5.00] |  |  |  |  |

### IS

Descriptive statistics and gender differences in IS sample

|  | Boys (N=941) | Girls (N=722) | Overall (N=1663) | t | df | p | d |
| --- | --- | --- | --- | --- | --- | --- | --- |
| IGDS Preoccupation |  |  |  | 12.416 | 1619.009 | <0.001 | 0.582 |
| Mean (SD) | 0.346 (0.476) | 0.107 (0.309) | 0.242 (0.429) |  |  |  |  |
| Median [Min, Max] | 0 [0, 1.00] | 0 [0, 1.00] | 0 [0, 1.00] |  |  |  |  |
| IGDS Tolerance |  |  |  | 9.039 | 1660.875 | <0.001 | 0.432 |
| Mean (SD) | 0.337 (0.473) | 0.152 (0.360) | 0.257 (0.437) |  |  |  |  |
| Median [Min, Max] | 0 [0, 1.00] | 0 [0, 1.00] | 0 [0, 1.00] |  |  |  |  |
| IGDS Withdrawal |  |  |  | 7.431 | 1610.744 | <0.001 | 0.348 |
| Mean (SD) | 0.179 (0.383) | 0.0637 (0.244) | 0.129 (0.335) |  |  |  |  |
| Median [Min, Max] | 0 [0, 1.00] | 0 [0, 1.00] | 0 [0, 1.00] |  |  |  |  |
| IGDS Persistence |  |  |  | 5.803 | 1647.542 | <0.001 | 0.281 |
| Mean (SD) | 0.302 (0.459) | 0.181 (0.386) | 0.250 (0.433) |  |  |  |  |
| Median [Min, Max] | 0 [0, 1.00] | 0 [0, 1.00] | 0 [0, 1.00] |  |  |  |  |
| IGDS Escape |  |  |  | 7.462 | 1635.621 | <0.001 | 0.362 |
| Mean (SD) | 0.408 (0.492) | 0.240 (0.427) | 0.335 (0.472) |  |  |  |  |
| Median [Min, Max] | 0 [0, 1.00] | 0 [0, 1.00] | 0 [0, 1.00] |  |  |  |  |
| IGDS Problems |  |  |  | 7.442 | 1594.996 | <0.001 | 0.347 |
| Mean (SD) | 0.168 (0.374) | 0.0568 (0.232) | 0.120 (0.325) |  |  |  |  |
| Median [Min, Max] | 0 [0, 1.00] | 0 [0, 1.00] | 0 [0, 1.00] |  |  |  |  |
| IGDS Deception |  |  |  | 6.913 | 1601.123 | <0.001 | 0.323 |
| Mean (SD) | 0.154 (0.361) | 0.0540 (0.226) | 0.111 (0.314) |  |  |  |  |
| Median [Min, Max] | 0 [0, 1.00] | 0 [0, 1.00] | 0 [0, 1.00] |  |  |  |  |
| IGDS Displacement |  |  |  | 5.590 | 1616.274 | <0.001 | 0.262 |
| Mean (SD) | 0.119 (0.324) | 0.0457 (0.209) | 0.0872 (0.282) |  |  |  |  |
| Median [Min, Max] | 0 [0, 1.00] | 0 [0, 1.00] | 0 [0, 1.00] |  |  |  |  |
| IGDS Conflict |  |  |  | 6.545 | 1563.332 | <0.001 | 0.304 |
| Mean (SD) | 0.123 (0.329) | 0.0388 (0.193) | 0.0866 (0.281) |  |  |  |  |
| Median [Min, Max] | 0 [0, 1.00] | 0 [0, 1.00] | 0 [0, 1.00] |  |  |  |  |
| SMDS Preoccupation |  |  |  | -0.503 | 1538.229 | 0.615 | 0.025 |
| Mean (SD) | 0.217 (0.412) | 0.227 (0.419) | 0.221 (0.415) |  |  |  |  |
| Median [Min, Max] | 0 [0, 1.00] | 0 [0, 1.00] | 0 [0, 1.00] |  |  |  |  |
| SMDS Tolerance |  |  |  | -1.187 | 1514.406 | 0.235 | 0.059 |
| Mean (SD) | 0.182 (0.386) | 0.205 (0.404) | 0.192 (0.394) |  |  |  |  |
| Median [Min, Max] | 0 [0, 1.00] | 0 [0, 1.00] | 0 [0, 1.00] |  |  |  |  |
| SMDS Withdrawal |  |  |  | -2.044 | 1474.815 | 0.041 | 0.102 |
| Mean (SD) | 0.148 (0.355) | 0.186 (0.389) | 0.164 (0.371) |  |  |  |  |
| Median [Min, Max] | 0 [0, 1.00] | 0 [0, 1.00] | 0 [0, 1.00] |  |  |  |  |
| SMDS Persistence |  |  |  | -6.798 | 1482.411 | <0.001 | 0.340 |
| Mean (SD) | 0.302 (0.459) | 0.464 (0.499) | 0.372 (0.484) |  |  |  |  |
| Median [Min, Max] | 0 [0, 1.00] | 0 [0, 1.00] | 0 [0, 1.00] |  |  |  |  |
| SMDS Escape |  |  |  | -7.894 | 1453.491 | <0.001 | 0.397 |
| Mean (SD) | 0.270 (0.444) | 0.456 (0.498) | 0.351 (0.477) |  |  |  |  |
| Median [Min, Max] | 0 [0, 1.00] | 0 [0, 1.00] | 0 [0, 1.00] |  |  |  |  |
| SMDS Problems |  |  |  | -1.096 | 1511.131 | 0.273 | 0.055 |
| Mean (SD) | 0.153 (0.360) | 0.173 (0.379) | 0.162 (0.368) |  |  |  |  |
| Median [Min, Max] | 0 [0, 1.00] | 0 [0, 1.00] | 0 [0, 1.00] |  |  |  |  |
| SMDS Deception |  |  |  | -2.465 | 1455.421 | 0.014 | 0.124 |
| Mean (SD) | 0.140 (0.347) | 0.186 (0.389) | 0.160 (0.367) |  |  |  |  |
| Median [Min, Max] | 0 [0, 1.00] | 0 [0, 1.00] | 0 [0, 1.00] |  |  |  |  |
| SMDS Displacement |  |  |  | 1.778 | 1621.118 | 0.076 | 0.087 |
| Mean (SD) | 0.124 (0.330) | 0.0970 (0.296) | 0.112 (0.316) |  |  |  |  |
| Median [Min, Max] | 0 [0, 1.00] | 0 [0, 1.00] | 0 [0, 1.00] |  |  |  |  |
| SMDS Conflict |  |  |  | 0.707 | 1582.696 | 0.479 | 0.035 |
| Mean (SD) | 0.115 (0.319) | 0.104 (0.305) | 0.110 (0.313) |  |  |  |  |
| Median [Min, Max] | 0 [0, 1.00] | 0 [0, 1.00] | 0 [0, 1.00] |  |  |  |  |
| MHCS Headache |  |  |  | -9.601 | 1430.249 | <0.001 | 0.484 |
| Mean (SD) | 2.17 (1.17) | 2.77 (1.34) | 2.43 (1.28) |  |  |  |  |
| Median [Min, Max] | 2.00 [1.00, 5.00] | 3.00 [1.00, 5.00] | 2.00 [1.00, 5.00] |  |  |  |  |
| MHCS Stomach ache |  |  |  | -10.034 | 1412.081 | <0.001 | 0.507 |
| Mean (SD) | 1.96 (1.10) | 2.57 (1.30) | 2.23 (1.23) |  |  |  |  |
| Median [Min, Max] | 2.00 [1.00, 5.00] | 2.00 [1.00, 5.00] | 2.00 [1.00, 5.00] |  |  |  |  |
| MHCS Back pain |  |  |  | -4.942 | 1440.456 | <0.001 | 0.249 |
| Mean (SD) | 1.97 (1.24) | 2.30 (1.41) | 2.11 (1.32) |  |  |  |  |
| Median [Min, Max] | 1.00 [1.00, 5.00] | 2.00 [1.00, 5.00] | 2.00 [1.00, 5.00] |  |  |  |  |
| MHCS Feeling low |  |  |  | -10.807 | 1502.556 | <0.001 | 0.539 |
| Mean (SD) | 2.11 (1.20) | 2.78 (1.27) | 2.40 (1.27) |  |  |  |  |
| Median [Min, Max] | 2.00 [1.00, 5.00] | 3.00 [1.00, 5.00] | 2.00 [1.00, 5.00] |  |  |  |  |
| MHCS Irritability |  |  |  | -7.940 | 1553.598 | <0.001 | 0.393 |
| Mean (SD) | 2.74 (1.23) | 3.23 (1.23) | 2.95 (1.25) |  |  |  |  |
| Median [Min, Max] | 3.00 [1.00, 5.00] | 3.00 [1.00, 5.00] | 3.00 [1.00, 5.00] |  |  |  |  |
| MHCS Nervousness |  |  |  | -15.704 | 1454.017 | <0.001 | 0.789 |
| Mean (SD) | 2.22 (1.23) | 3.25 (1.38) | 2.67 (1.39) |  |  |  |  |
| Median [Min, Max] | 2.00 [1.00, 5.00] | 3.00 [1.00, 5.00] | 2.00 [1.00, 5.00] |  |  |  |  |
| MHCS Problems falling asleep |  |  |  | -4.030 | 1537.135 | <0.001 | 0.200 |
| Mean (SD) | 2.44 (1.43) | 2.73 (1.45) | 2.56 (1.44) |  |  |  |  |
| Median [Min, Max] | 2.00 [1.00, 5.00] | 2.50 [1.00, 5.00] | 2.00 [1.00, 5.00] |  |  |  |  |
| MHCS Dizziness |  |  |  | -9.822 | 1352.487 | <0.001 | 0.500 |
| Mean (SD) | 1.79 (1.12) | 2.42 (1.40) | 2.06 (1.29) |  |  |  |  |
| Median [Min, Max] | 1.00 [1.00, 5.00] | 2.00 [1.00, 5.00] | 2.00 [1.00, 5.00] |  |  |  |  |
| WHO-5 Wellbeing 1 |  |  |  | 5.360 | 1514.022 | <0.001 | 0.267 |
| Mean (SD) | 3.65 (0.990) | 3.39 (1.04) | 3.54 (1.02) |  |  |  |  |
| Median [Min, Max] | 4.00 [0, 5.00] | 4.00 [0, 5.00] | 4.00 [0, 5.00] |  |  |  |  |
| WHO-5 Wellbeing 2 |  |  |  | 5.749 | 1562.733 | <0.001 | 0.284 |
| Mean (SD) | 3.18 (1.22) | 2.83 (1.20) | 3.03 (1.22) |  |  |  |  |
| Median [Min, Max] | 3.00 [0, 5.00] | 3.00 [0, 5.00] | 3.00 [0, 5.00] |  |  |  |  |
| WHO-5 Wellbeing 3 |  |  |  | 7.209 | 1481.323 | <0.001 | 0.361 |
| Mean (SD) | 3.48 (1.17) | 3.04 (1.27) | 3.29 (1.24) |  |  |  |  |
| Median [Min, Max] | 4.00 [0, 5.00] | 3.00 [0, 5.00] | 4.00 [0, 5.00] |  |  |  |  |
| WHO-5 Wellbeing 4 |  |  |  | 4.759 | 1552.235 | <0.001 | 0.235 |
| Mean (SD) | 2.70 (1.45) | 2.36 (1.45) | 2.55 (1.46) |  |  |  |  |
| Median [Min, Max] | 3.00 [0, 5.00] | 2.00 [0, 5.00] | 3.00 [0, 5.00] |  |  |  |  |
| WHO-5 Wellbeing 5 |  |  |  | 3.813 | 1549.056 | <0.001 | 0.189 |
| Mean (SD) | 3.29 (1.33) | 3.04 (1.33) | 3.18 (1.33) |  |  |  |  |
| Median [Min, Max] | 4.00 [0, 5.00] | 3.00 [0, 5.00] | 3.00 [0, 5.00] |  |  |  |  |
| WHO-5 Overall Wellbeing (Averaged) |  |  |  | 6.758 | 1512.830 | <0.001 | 0.336 |
| Mean (SD) | 3.26 (0.958) | 2.93 (1.00) | 3.12 (0.991) |  |  |  |  |
| Median [Min, Max] | 3.40 [0, 5.00] | 3.00 [0, 5.00] | 3.20 [0, 5.00] |  |  |  |  |
| Loneliness |  |  |  | -8.864 | 1501.012 | <0.001 | 0.442 |
| Mean (SD) | 2.13 (0.979) | 2.58 (1.04) | 2.33 (1.03) |  |  |  |  |
| Median [Min, Max] | 2.00 [1.00, 5.00] | 3.00 [1.00, 5.00] | 2.00 [1.00, 5.00] |  |  |  |  |

### MK

Descriptive statistics and gender differences in MK sample

|  | Boys (N=756) | Girls (N=775) | Overall (N=1531) | t | df | p | d |
| --- | --- | --- | --- | --- | --- | --- | --- |
| IGDS Preoccupation |  |  |  | 13.069 | 1503.037 | <0.001 | 0.669 |
| Mean (SD) | 0.582 (0.494) | 0.268 (0.443) | 0.423 (0.494) |  |  |  |  |
| Median [Min, Max] | 1.00 [0, 1.00] | 0 [0, 1.00] | 0 [0, 1.00] |  |  |  |  |
| IGDS Tolerance |  |  |  | 7.007 | 1483.297 | <0.001 | 0.359 |
| Mean (SD) | 0.390 (0.488) | 0.227 (0.419) | 0.308 (0.462) |  |  |  |  |
| Median [Min, Max] | 0 [0, 1.00] | 0 [0, 1.00] | 0 [0, 1.00] |  |  |  |  |
| IGDS Withdrawal |  |  |  | 6.507 | 1442.162 | <0.001 | 0.334 |
| Mean (SD) | 0.295 (0.456) | 0.157 (0.364) | 0.225 (0.418) |  |  |  |  |
| Median [Min, Max] | 0 [0, 1.00] | 0 [0, 1.00] | 0 [0, 1.00] |  |  |  |  |
| IGDS Persistence |  |  |  | 6.489 | 1464.515 | <0.001 | 0.332 |
| Mean (SD) | 0.331 (0.471) | 0.187 (0.390) | 0.258 (0.438) |  |  |  |  |
| Median [Min, Max] | 0 [0, 1.00] | 0 [0, 1.00] | 0 [0, 1.00] |  |  |  |  |
| IGDS Escape |  |  |  | 3.733 | 1528.374 | <0.001 | 0.191 |
| Mean (SD) | 0.560 (0.497) | 0.465 (0.499) | 0.511 (0.500) |  |  |  |  |
| Median [Min, Max] | 1.00 [0, 1.00] | 0 [0, 1.00] | 1.00 [0, 1.00] |  |  |  |  |
| IGDS Problems |  |  |  | 5.229 | 1435.989 | <0.001 | 0.268 |
| Mean (SD) | 0.226 (0.419) | 0.125 (0.331) | 0.175 (0.380) |  |  |  |  |
| Median [Min, Max] | 0 [0, 1.00] | 0 [0, 1.00] | 0 [0, 1.00] |  |  |  |  |
| IGDS Deception |  |  |  | 3.904 | 1443.691 | <0.001 | 0.200 |
| Mean (SD) | 0.165 (0.372) | 0.0981 (0.298) | 0.131 (0.338) |  |  |  |  |
| Median [Min, Max] | 0 [0, 1.00] | 0 [0, 1.00] | 0 [0, 1.00] |  |  |  |  |
| IGDS Displacement |  |  |  | 2.576 | 1493.029 | 0.010 | 0.132 |
| Mean (SD) | 0.172 (0.378) | 0.125 (0.331) | 0.148 (0.355) |  |  |  |  |
| Median [Min, Max] | 0 [0, 1.00] | 0 [0, 1.00] | 0 [0, 1.00] |  |  |  |  |
| IGDS Conflict |  |  |  | 5.318 | 1395.234 | <0.001 | 0.273 |
| Mean (SD) | 0.187 (0.390) | 0.0929 (0.290) | 0.139 (0.346) |  |  |  |  |
| Median [Min, Max] | 0 [0, 1.00] | 0 [0, 1.00] | 0 [0, 1.00] |  |  |  |  |
| SMDS Preoccupation |  |  |  | -3.490 | 1527.760 | <0.001 | 0.178 |
| Mean (SD) | 0.315 (0.465) | 0.400 (0.490) | 0.358 (0.480) |  |  |  |  |
| Median [Min, Max] | 0 [0, 1.00] | 0 [0, 1.00] | 0 [0, 1.00] |  |  |  |  |
| SMDS Tolerance |  |  |  | -3.589 | 1524.486 | <0.001 | 0.183 |
| Mean (SD) | 0.261 (0.439) | 0.345 (0.476) | 0.303 (0.460) |  |  |  |  |
| Median [Min, Max] | 0 [0, 1.00] | 0 [0, 1.00] | 0 [0, 1.00] |  |  |  |  |
| SMDS Withdrawal |  |  |  | -5.838 | 1520.799 | <0.001 | 0.298 |
| Mean (SD) | 0.275 (0.447) | 0.415 (0.493) | 0.346 (0.476) |  |  |  |  |
| Median [Min, Max] | 0 [0, 1.00] | 0 [0, 1.00] | 0 [0, 1.00] |  |  |  |  |
| SMDS Persistence |  |  |  | -6.377 | 1526.917 | <0.001 | 0.326 |
| Mean (SD) | 0.438 (0.496) | 0.599 (0.490) | 0.519 (0.500) |  |  |  |  |
| Median [Min, Max] | 0 [0, 1.00] | 1.00 [0, 1.00] | 1.00 [0, 1.00] |  |  |  |  |
| SMDS Escape |  |  |  | -6.067 | 1520.893 | <0.001 | 0.310 |
| Mean (SD) | 0.500 (0.500) | 0.652 (0.477) | 0.577 (0.494) |  |  |  |  |
| Median [Min, Max] | 0.500 [0, 1.00] | 1.00 [0, 1.00] | 1.00 [0, 1.00] |  |  |  |  |
| SMDS Problems |  |  |  | -0.970 | 1528.893 | 0.332 | 0.050 |
| Mean (SD) | 0.212 (0.409) | 0.232 (0.423) | 0.222 (0.416) |  |  |  |  |
| Median [Min, Max] | 0 [0, 1.00] | 0 [0, 1.00] | 0 [0, 1.00] |  |  |  |  |
| SMDS Deception |  |  |  | 1.247 | 1518.710 | 0.212 | 0.064 |
| Mean (SD) | 0.177 (0.382) | 0.154 (0.361) | 0.165 (0.372) |  |  |  |  |
| Median [Min, Max] | 0 [0, 1.00] | 0 [0, 1.00] | 0 [0, 1.00] |  |  |  |  |
| SMDS Displacement |  |  |  | -4.814 | 1516.098 | <0.001 | 0.246 |
| Mean (SD) | 0.231 (0.422) | 0.342 (0.475) | 0.287 (0.453) |  |  |  |  |
| Median [Min, Max] | 0 [0, 1.00] | 0 [0, 1.00] | 0 [0, 1.00] |  |  |  |  |
| SMDS Conflict |  |  |  | -2.304 | 1523.004 | 0.021 | 0.118 |
| Mean (SD) | 0.179 (0.383) | 0.226 (0.418) | 0.202 (0.402) |  |  |  |  |
| Median [Min, Max] | 0 [0, 1.00] | 0 [0, 1.00] | 0 [0, 1.00] |  |  |  |  |
| MHCS Headache |  |  |  | -10.406 | 1514.035 | <0.001 | 0.531 |
| Mean (SD) | 1.98 (1.26) | 2.70 (1.43) | 2.34 (1.39) |  |  |  |  |
| Median [Min, Max] | 1.00 [1.00, 5.00] | 3.00 [1.00, 5.00] | 2.00 [1.00, 5.00] |  |  |  |  |
| MHCS Stomach ache |  |  |  | -11.209 | 1522.732 | <0.001 | 0.572 |
| Mean (SD) | 1.61 (0.975) | 2.19 (1.07) | 1.91 (1.06) |  |  |  |  |
| Median [Min, Max] | 1.00 [1.00, 5.00] | 2.00 [1.00, 5.00] | 2.00 [1.00, 5.00] |  |  |  |  |
| MHCS Back pain |  |  |  | -3.143 | 1520.626 | 0.002 | 0.160 |
| Mean (SD) | 1.93 (1.28) | 2.14 (1.41) | 2.04 (1.35) |  |  |  |  |
| Median [Min, Max] | 1.00 [1.00, 5.00] | 2.00 [1.00, 5.00] | 1.00 [1.00, 5.00] |  |  |  |  |
| MHCS Feeling low |  |  |  | -11.565 | 1528.965 | <0.001 | 0.591 |
| Mean (SD) | 2.51 (1.38) | 3.34 (1.43) | 2.93 (1.46) |  |  |  |  |
| Median [Min, Max] | 2.00 [1.00, 5.00] | 4.00 [1.00, 5.00] | 3.00 [1.00, 5.00] |  |  |  |  |
| MHCS Irritability |  |  |  | -7.995 | 1509.824 | <0.001 | 0.408 |
| Mean (SD) | 2.17 (1.34) | 2.76 (1.54) | 2.47 (1.47) |  |  |  |  |
| Median [Min, Max] | 2.00 [1.00, 5.00] | 3.00 [1.00, 5.00] | 2.00 [1.00, 5.00] |  |  |  |  |
| MHCS Nervousness |  |  |  | -9.505 | 1528.125 | <0.001 | 0.486 |
| Mean (SD) | 2.82 (1.43) | 3.52 (1.43) | 3.18 (1.47) |  |  |  |  |
| Median [Min, Max] | 3.00 [1.00, 5.00] | 4.00 [1.00, 5.00] | 3.00 [1.00, 5.00] |  |  |  |  |
| MHCS Problems falling asleep |  |  |  | -3.845 | 1522.886 | <0.001 | 0.196 |
| Mean (SD) | 2.20 (1.44) | 2.50 (1.58) | 2.35 (1.52) |  |  |  |  |
| Median [Min, Max] | 1.00 [1.00, 5.00] | 2.00 [1.00, 5.00] | 2.00 [1.00, 5.00] |  |  |  |  |
| MHCS Dizziness |  |  |  | -6.119 | 1470.568 | <0.001 | 0.312 |
| Mean (SD) | 1.58 (1.08) | 1.96 (1.36) | 1.77 (1.25) |  |  |  |  |
| Median [Min, Max] | 1.00 [1.00, 5.00] | 1.00 [1.00, 5.00] | 1.00 [1.00, 5.00] |  |  |  |  |
| WHO-5 Wellbeing 1 |  |  |  | 7.115 | 1528.289 | <0.001 | 0.364 |
| Mean (SD) | 3.70 (1.21) | 3.24 (1.27) | 3.47 (1.26) |  |  |  |  |
| Median [Min, Max] | 4.00 [0, 5.00] | 4.00 [0, 5.00] | 4.00 [0, 5.00] |  |  |  |  |
| WHO-5 Wellbeing 2 |  |  |  | 12.765 | 1517.395 | <0.001 | 0.652 |
| Mean (SD) | 3.62 (1.27) | 2.74 (1.42) | 3.17 (1.42) |  |  |  |  |
| Median [Min, Max] | 4.00 [0, 5.00] | 3.00 [0, 5.00] | 3.00 [0, 5.00] |  |  |  |  |
| WHO-5 Wellbeing 3 |  |  |  | 10.644 | 1516.609 | <0.001 | 0.543 |
| Mean (SD) | 3.72 (1.31) | 2.96 (1.47) | 3.34 (1.44) |  |  |  |  |
| Median [Min, Max] | 4.00 [0, 5.00] | 3.00 [0, 5.00] | 4.00 [0, 5.00] |  |  |  |  |
| WHO-5 Wellbeing 4 |  |  |  | 9.789 | 1526.437 | <0.001 | 0.501 |
| Mean (SD) | 3.08 (1.67) | 2.25 (1.65) | 2.66 (1.71) |  |  |  |  |
| Median [Min, Max] | 4.00 [0, 5.00] | 2.00 [0, 5.00] | 3.00 [0, 5.00] |  |  |  |  |
| WHO-5 Wellbeing 5 |  |  |  | 8.172 | 1513.418 | <0.001 | 0.417 |
| Mean (SD) | 3.66 (1.40) | 3.03 (1.59) | 3.34 (1.53) |  |  |  |  |
| Median [Min, Max] | 4.00 [0, 5.00] | 3.00 [0, 5.00] | 4.00 [0, 5.00] |  |  |  |  |
| WHO-5 Overall Wellbeing (Averaged) |  |  |  | 12.504 | 1520.644 | <0.001 | 0.638 |
| Mean (SD) | 3.55 (1.05) | 2.85 (1.16) | 3.20 (1.16) |  |  |  |  |
| Median [Min, Max] | 3.60 [0, 5.00] | 2.80 [0, 5.00] | 3.20 [0, 5.00] |  |  |  |  |
| Loneliness |  |  |  | -12.504 | 1523.904 | <0.001 | 0.639 |
| Mean (SD) | 2.16 (1.05) | 2.86 (1.14) | 2.51 (1.15) |  |  |  |  |
| Median [Min, Max] | 2.00 [1.00, 5.00] | 3.00 [1.00, 5.00] | 2.00 [1.00, 5.00] |  |  |  |  |

### MT

Descriptive statistics and gender differences in MT sample

|  | Boys (N=1412) | Girls (N=1279) | Overall (N=2691) | t | df | p | d |
| --- | --- | --- | --- | --- | --- | --- | --- |
| IGDS Preoccupation |  |  |  | 10.488 | 2682.869 | <0.001 | 0.404 |
| Mean (SD) | 0.537 (0.499) | 0.340 (0.474) | 0.443 (0.497) |  |  |  |  |
| Median [Min, Max] | 1.00 [0, 1.00] | 0 [0, 1.00] | 0 [0, 1.00] |  |  |  |  |
| IGDS Tolerance |  |  |  | 6.538 | 2680.071 | <0.001 | 0.252 |
| Mean (SD) | 0.482 (0.500) | 0.358 (0.480) | 0.423 (0.494) |  |  |  |  |
| Median [Min, Max] | 0 [0, 1.00] | 0 [0, 1.00] | 0 [0, 1.00] |  |  |  |  |
| IGDS Withdrawal |  |  |  | 4.376 | 2688.940 | <0.001 | 0.168 |
| Mean (SD) | 0.294 (0.456) | 0.220 (0.415) | 0.259 (0.438) |  |  |  |  |
| Median [Min, Max] | 0 [0, 1.00] | 0 [0, 1.00] | 0 [0, 1.00] |  |  |  |  |
| IGDS Persistence |  |  |  | 4.554 | 2687.637 | <0.001 | 0.175 |
| Mean (SD) | 0.341 (0.474) | 0.261 (0.439) | 0.303 (0.460) |  |  |  |  |
| Median [Min, Max] | 0 [0, 1.00] | 0 [0, 1.00] | 0 [0, 1.00] |  |  |  |  |
| IGDS Escape |  |  |  | 3.235 | 2654.132 | 0.001 | 0.125 |
| Mean (SD) | 0.593 (0.491) | 0.531 (0.499) | 0.563 (0.496) |  |  |  |  |
| Median [Min, Max] | 1.00 [0, 1.00] | 1.00 [0, 1.00] | 1.00 [0, 1.00] |  |  |  |  |
| IGDS Problems |  |  |  | 7.804 | 2671.743 | <0.001 | 0.299 |
| Mean (SD) | 0.311 (0.463) | 0.183 (0.387) | 0.250 (0.433) |  |  |  |  |
| Median [Min, Max] | 0 [0, 1.00] | 0 [0, 1.00] | 0 [0, 1.00] |  |  |  |  |
| IGDS Deception |  |  |  | 3.817 | 2685.274 | <0.001 | 0.146 |
| Mean (SD) | 0.188 (0.391) | 0.134 (0.341) | 0.163 (0.369) |  |  |  |  |
| Median [Min, Max] | 0 [0, 1.00] | 0 [0, 1.00] | 0 [0, 1.00] |  |  |  |  |
| IGDS Displacement |  |  |  | 2.021 | 2688.468 | 0.043 | 0.078 |
| Mean (SD) | 0.145 (0.352) | 0.119 (0.324) | 0.133 (0.339) |  |  |  |  |
| Median [Min, Max] | 0 [0, 1.00] | 0 [0, 1.00] | 0 [0, 1.00] |  |  |  |  |
| IGDS Conflict |  |  |  | 5.032 | 2659.300 | <0.001 | 0.192 |
| Mean (SD) | 0.172 (0.378) | 0.106 (0.307) | 0.140 (0.348) |  |  |  |  |
| Median [Min, Max] | 0 [0, 1.00] | 0 [0, 1.00] | 0 [0, 1.00] |  |  |  |  |
| SMDS Preoccupation |  |  |  | -3.583 | 2632.538 | <0.001 | 0.139 |
| Mean (SD) | 0.305 (0.461) | 0.371 (0.483) | 0.336 (0.473) |  |  |  |  |
| Median [Min, Max] | 0 [0, 1.00] | 0 [0, 1.00] | 0 [0, 1.00] |  |  |  |  |
| SMDS Tolerance |  |  |  | -5.252 | 2611.324 | <0.001 | 0.203 |
| Mean (SD) | 0.283 (0.450) | 0.378 (0.485) | 0.328 (0.469) |  |  |  |  |
| Median [Min, Max] | 0 [0, 1.00] | 0 [0, 1.00] | 0 [0, 1.00] |  |  |  |  |
| SMDS Withdrawal |  |  |  | -7.596 | 2625.578 | <0.001 | 0.294 |
| Mean (SD) | 0.335 (0.472) | 0.478 (0.500) | 0.403 (0.491) |  |  |  |  |
| Median [Min, Max] | 0 [0, 1.00] | 0 [0, 1.00] | 0 [0, 1.00] |  |  |  |  |
| SMDS Persistence |  |  |  | -10.030 | 2653.477 | <0.001 | 0.387 |
| Mean (SD) | 0.384 (0.486) | 0.574 (0.495) | 0.474 (0.499) |  |  |  |  |
| Median [Min, Max] | 0 [0, 1.00] | 1.00 [0, 1.00] | 0 [0, 1.00] |  |  |  |  |
| SMDS Escape |  |  |  | -8.348 | 2685.993 | <0.001 | 0.321 |
| Mean (SD) | 0.521 (0.500) | 0.676 (0.468) | 0.595 (0.491) |  |  |  |  |
| Median [Min, Max] | 1.00 [0, 1.00] | 1.00 [0, 1.00] | 1.00 [0, 1.00] |  |  |  |  |
| SMDS Problems |  |  |  | -4.506 | 2611.264 | <0.001 | 0.175 |
| Mean (SD) | 0.266 (0.442) | 0.346 (0.476) | 0.304 (0.460) |  |  |  |  |
| Median [Min, Max] | 0 [0, 1.00] | 0 [0, 1.00] | 0 [0, 1.00] |  |  |  |  |
| SMDS Deception |  |  |  | -2.306 | 2612.055 | 0.021 | 0.089 |
| Mean (SD) | 0.166 (0.373) | 0.201 (0.401) | 0.183 (0.387) |  |  |  |  |
| Median [Min, Max] | 0 [0, 1.00] | 0 [0, 1.00] | 0 [0, 1.00] |  |  |  |  |
| SMDS Displacement |  |  |  | -5.645 | 2535.592 | <0.001 | 0.219 |
| Mean (SD) | 0.176 (0.381) | 0.266 (0.442) | 0.219 (0.413) |  |  |  |  |
| Median [Min, Max] | 0 [0, 1.00] | 0 [0, 1.00] | 0 [0, 1.00] |  |  |  |  |
| SMDS Conflict |  |  |  | -4.325 | 2555.185 | <0.001 | 0.168 |
| Mean (SD) | 0.160 (0.367) | 0.226 (0.418) | 0.191 (0.393) |  |  |  |  |
| Median [Min, Max] | 0 [0, 1.00] | 0 [0, 1.00] | 0 [0, 1.00] |  |  |  |  |
| MHCS Headache |  |  |  | -10.551 | 2587.577 | <0.001 | 0.409 |
| Mean (SD) | 2.14 (1.29) | 2.69 (1.43) | 2.40 (1.39) |  |  |  |  |
| Median [Min, Max] | 2.00 [1.00, 5.00] | 2.00 [1.00, 5.00] | 2.00 [1.00, 5.00] |  |  |  |  |
| MHCS Stomach ache |  |  |  | -12.916 | 2512.154 | <0.001 | 0.503 |
| Mean (SD) | 1.76 (1.09) | 2.36 (1.29) | 2.05 (1.23) |  |  |  |  |
| Median [Min, Max] | 1.00 [1.00, 5.00] | 2.00 [1.00, 5.00] | 2.00 [1.00, 5.00] |  |  |  |  |
| MHCS Back pain |  |  |  | -9.822 | 2514.201 | <0.001 | 0.382 |
| Mean (SD) | 1.91 (1.26) | 2.44 (1.48) | 2.16 (1.39) |  |  |  |  |
| Median [Min, Max] | 1.00 [1.00, 5.00] | 2.00 [1.00, 5.00] | 2.00 [1.00, 5.00] |  |  |  |  |
| MHCS Feeling low |  |  |  | -13.988 | 2570.658 | <0.001 | 0.543 |
| Mean (SD) | 2.13 (1.33) | 2.90 (1.50) | 2.50 (1.46) |  |  |  |  |
| Median [Min, Max] | 2.00 [1.00, 5.00] | 3.00 [1.00, 5.00] | 2.00 [1.00, 5.00] |  |  |  |  |
| MHCS Irritability |  |  |  | -11.233 | 2607.020 | <0.001 | 0.435 |
| Mean (SD) | 2.35 (1.34) | 2.95 (1.45) | 2.63 (1.43) |  |  |  |  |
| Median [Min, Max] | 2.00 [1.00, 5.00] | 3.00 [1.00, 5.00] | 2.00 [1.00, 5.00] |  |  |  |  |
| MHCS Nervousness |  |  |  | -15.117 | 2643.708 | <0.001 | 0.584 |
| Mean (SD) | 2.58 (1.37) | 3.40 (1.41) | 2.97 (1.45) |  |  |  |  |
| Median [Min, Max] | 2.00 [1.00, 5.00] | 4.00 [1.00, 5.00] | 3.00 [1.00, 5.00] |  |  |  |  |
| MHCS Problems falling asleep |  |  |  | -9.881 | 2572.850 | <0.001 | 0.384 |
| Mean (SD) | 2.12 (1.43) | 2.70 (1.60) | 2.39 (1.54) |  |  |  |  |
| Median [Min, Max] | 1.00 [1.00, 5.00] | 2.00 [1.00, 5.00] | 2.00 [1.00, 5.00] |  |  |  |  |
| MHCS Dizziness |  |  |  | -12.810 | 2355.760 | <0.001 | 0.501 |
| Mean (SD) | 1.67 (1.11) | 2.32 (1.48) | 1.98 (1.34) |  |  |  |  |
| Median [Min, Max] | 1.00 [1.00, 5.00] | 2.00 [1.00, 5.00] | 1.00 [1.00, 5.00] |  |  |  |  |
| WHO-5 Wellbeing 1 |  |  |  | 8.638 | 2634.419 | <0.001 | 0.334 |
| Mean (SD) | 3.48 (1.25) | 3.05 (1.31) | 3.28 (1.29) |  |  |  |  |
| Median [Min, Max] | 4.00 [0, 5.00] | 3.00 [0, 5.00] | 4.00 [0, 5.00] |  |  |  |  |
| WHO-5 Wellbeing 2 |  |  |  | 10.925 | 2624.644 | <0.001 | 0.423 |
| Mean (SD) | 3.27 (1.34) | 2.69 (1.42) | 2.99 (1.41) |  |  |  |  |
| Median [Min, Max] | 4.00 [0, 5.00] | 3.00 [0, 5.00] | 3.00 [0, 5.00] |  |  |  |  |
| WHO-5 Wellbeing 3 |  |  |  | 11.160 | 2613.640 | <0.001 | 0.432 |
| Mean (SD) | 3.28 (1.38) | 2.67 (1.48) | 2.99 (1.46) |  |  |  |  |
| Median [Min, Max] | 4.00 [0, 5.00] | 3.00 [0, 5.00] | 3.00 [0, 5.00] |  |  |  |  |
| WHO-5 Wellbeing 4 |  |  |  | 9.110 | 2667.636 | <0.001 | 0.351 |
| Mean (SD) | 2.90 (1.64) | 2.32 (1.62) | 2.62 (1.66) |  |  |  |  |
| Median [Min, Max] | 3.00 [0, 5.00] | 2.00 [0, 5.00] | 3.00 [0, 5.00] |  |  |  |  |
| WHO-5 Wellbeing 5 |  |  |  | 11.119 | 2610.701 | <0.001 | 0.431 |
| Mean (SD) | 3.38 (1.40) | 2.75 (1.51) | 3.08 (1.48) |  |  |  |  |
| Median [Min, Max] | 4.00 [0, 5.00] | 3.00 [0, 5.00] | 3.00 [0, 5.00] |  |  |  |  |
| WHO-5 Overall Wellbeing (Averaged) |  |  |  | 12.502 | 2623.363 | <0.001 | 0.484 |
| Mean (SD) | 3.26 (1.13) | 2.70 (1.20) | 2.99 (1.20) |  |  |  |  |
| Median [Min, Max] | 3.40 [0, 5.00] | 2.80 [0, 5.00] | 3.00 [0, 5.00] |  |  |  |  |
| Loneliness |  |  |  | -11.417 | 2660.839 | <0.001 | 0.441 |
| Mean (SD) | 2.30 (1.05) | 2.76 (1.05) | 2.52 (1.07) |  |  |  |  |
| Median [Min, Max] | 2.00 [1.00, 5.00] | 3.00 [1.00, 5.00] | 2.00 [1.00, 5.00] |  |  |  |  |

### NL

Descriptive statistics and gender differences in NL sample

|  | Boys (N=1953) | Girls (N=1372) | Overall (N=3325) | t | df | p | d |
| --- | --- | --- | --- | --- | --- | --- | --- |
| IGDS Preoccupation |  |  |  | 11.153 | 3307.855 | <0.001 | 0.374 |
| Mean (SD) | 0.287 (0.452) | 0.133 (0.340) | 0.223 (0.417) |  |  |  |  |
| Median [Min, Max] | 0 [0, 1.00] | 0 [0, 1.00] | 0 [0, 1.00] |  |  |  |  |
| IGDS Tolerance |  |  |  | 9.934 | 3322.966 | <0.001 | 0.330 |
| Mean (SD) | 0.207 (0.405) | 0.0882 (0.284) | 0.158 (0.365) |  |  |  |  |
| Median [Min, Max] | 0 [0, 1.00] | 0 [0, 1.00] | 0 [0, 1.00] |  |  |  |  |
| IGDS Withdrawal |  |  |  | 10.053 | 3317.963 | <0.001 | 0.336 |
| Mean (SD) | 0.236 (0.425) | 0.108 (0.310) | 0.183 (0.387) |  |  |  |  |
| Median [Min, Max] | 0 [0, 1.00] | 0 [0, 1.00] | 0 [0, 1.00] |  |  |  |  |
| IGDS Persistence |  |  |  | 7.091 | 3315.313 | <0.001 | 0.237 |
| Mean (SD) | 0.154 (0.361) | 0.0765 (0.266) | 0.122 (0.327) |  |  |  |  |
| Median [Min, Max] | 0 [0, 1.00] | 0 [0, 1.00] | 0 [0, 1.00] |  |  |  |  |
| IGDS Escape |  |  |  | 4.827 | 3013.037 | <0.001 | 0.169 |
| Mean (SD) | 0.441 (0.497) | 0.359 (0.480) | 0.407 (0.491) |  |  |  |  |
| Median [Min, Max] | 0 [0, 1.00] | 0 [0, 1.00] | 0 [0, 1.00] |  |  |  |  |
| IGDS Problems |  |  |  | 9.221 | 3318.880 | <0.001 | 0.304 |
| Mean (SD) | 0.171 (0.377) | 0.0700 (0.255) | 0.129 (0.336) |  |  |  |  |
| Median [Min, Max] | 0 [0, 1.00] | 0 [0, 1.00] | 0 [0, 1.00] |  |  |  |  |
| IGDS Deception |  |  |  | 1.345 | 3070.706 | 0.179 | 0.047 |
| Mean (SD) | 0.0942 (0.292) | 0.0809 (0.273) | 0.0887 (0.284) |  |  |  |  |
| Median [Min, Max] | 0 [0, 1.00] | 0 [0, 1.00] | 0 [0, 1.00] |  |  |  |  |
| IGDS Displacement |  |  |  | 4.756 | 3304.650 | <0.001 | 0.160 |
| Mean (SD) | 0.0932 (0.291) | 0.0510 (0.220) | 0.0758 (0.265) |  |  |  |  |
| Median [Min, Max] | 0 [0, 1.00] | 0 [0, 1.00] | 0 [0, 1.00] |  |  |  |  |
| IGDS Conflict |  |  |  | 3.640 | 3320.234 | <0.001 | 0.121 |
| Mean (SD) | 0.0471 (0.212) | 0.0241 (0.153) | 0.0376 (0.190) |  |  |  |  |
| Median [Min, Max] | 0 [0, 1.00] | 0 [0, 1.00] | 0 [0, 1.00] |  |  |  |  |
| SMDS Preoccupation |  |  |  | -0.823 | 2925.569 | 0.410 | 0.029 |
| Mean (SD) | 0.281 (0.449) | 0.294 (0.456) | 0.286 (0.452) |  |  |  |  |
| Median [Min, Max] | 0 [0, 1.00] | 0 [0, 1.00] | 0 [0, 1.00] |  |  |  |  |
| SMDS Tolerance |  |  |  | 0.161 | 2965.239 | 0.872 | 0.006 |
| Mean (SD) | 0.0942 (0.292) | 0.0926 (0.290) | 0.0935 (0.291) |  |  |  |  |
| Median [Min, Max] | 0 [0, 1.00] | 0 [0, 1.00] | 0 [0, 1.00] |  |  |  |  |
| SMDS Withdrawal |  |  |  | -4.133 | 2688.676 | <0.001 | 0.149 |
| Mean (SD) | 0.135 (0.341) | 0.189 (0.391) | 0.157 (0.364) |  |  |  |  |
| Median [Min, Max] | 0 [0, 1.00] | 0 [0, 1.00] | 0 [0, 1.00] |  |  |  |  |
| SMDS Persistence |  |  |  | -8.601 | 2712.434 | <0.001 | 0.310 |
| Mean (SD) | 0.244 (0.430) | 0.385 (0.487) | 0.302 (0.459) |  |  |  |  |
| Median [Min, Max] | 0 [0, 1.00] | 0 [0, 1.00] | 0 [0, 1.00] |  |  |  |  |
| SMDS Escape |  |  |  | -8.109 | 2884.410 | <0.001 | 0.287 |
| Mean (SD) | 0.369 (0.483) | 0.509 (0.500) | 0.427 (0.495) |  |  |  |  |
| Median [Min, Max] | 0 [0, 1.00] | 1.00 [0, 1.00] | 0 [0, 1.00] |  |  |  |  |
| SMDS Problems |  |  |  | -0.932 | 2888.854 | 0.351 | 0.033 |
| Mean (SD) | 0.139 (0.346) | 0.151 (0.358) | 0.144 (0.351) |  |  |  |  |
| Median [Min, Max] | 0 [0, 1.00] | 0 [0, 1.00] | 0 [0, 1.00] |  |  |  |  |
| SMDS Deception |  |  |  | -2.279 | 2772.412 | 0.023 | 0.082 |
| Mean (SD) | 0.109 (0.312) | 0.136 (0.342) | 0.120 (0.325) |  |  |  |  |
| Median [Min, Max] | 0 [0, 1.00] | 0 [0, 1.00] | 0 [0, 1.00] |  |  |  |  |
| SMDS Displacement |  |  |  | -4.092 | 2695.767 | <0.001 | 0.148 |
| Mean (SD) | 0.138 (0.345) | 0.192 (0.394) | 0.160 (0.367) |  |  |  |  |
| Median [Min, Max] | 0 [0, 1.00] | 0 [0, 1.00] | 0 [0, 1.00] |  |  |  |  |
| SMDS Conflict |  |  |  | -0.698 | 2854.674 | 0.485 | 0.025 |
| Mean (SD) | 0.0471 (0.212) | 0.0525 (0.223) | 0.0493 (0.217) |  |  |  |  |
| Median [Min, Max] | 0 [0, 1.00] | 0 [0, 1.00] | 0 [0, 1.00] |  |  |  |  |
| MHCS Headache |  |  |  | -12.725 | 2563.793 | <0.001 | 0.464 |
| Mean (SD) | 1.89 (1.14) | 2.47 (1.40) | 2.13 (1.29) |  |  |  |  |
| Median [Min, Max] | 1.00 [1.00, 5.00] | 2.00 [1.00, 5.00] | 2.00 [1.00, 5.00] |  |  |  |  |
| MHCS Stomach ache |  |  |  | -12.371 | 2430.854 | <0.001 | 0.457 |
| Mean (SD) | 1.55 (0.949) | 2.05 (1.25) | 1.76 (1.11) |  |  |  |  |
| Median [Min, Max] | 1.00 [1.00, 5.00] | 2.00 [1.00, 5.00] | 1.00 [1.00, 5.00] |  |  |  |  |
| MHCS Back pain |  |  |  | -5.451 | 2659.034 | <0.001 | 0.197 |
| Mean (SD) | 1.67 (1.10) | 1.90 (1.28) | 1.77 (1.18) |  |  |  |  |
| Median [Min, Max] | 1.00 [1.00, 5.00] | 1.00 [1.00, 5.00] | 1.00 [1.00, 5.00] |  |  |  |  |
| MHCS Feeling low |  |  |  | -12.871 | 2585.267 | <0.001 | 0.469 |
| Mean (SD) | 1.74 (1.18) | 2.34 (1.42) | 1.99 (1.32) |  |  |  |  |
| Median [Min, Max] | 1.00 [1.00, 5.00] | 2.00 [1.00, 5.00] | 1.00 [1.00, 5.00] |  |  |  |  |
| MHCS Irritability |  |  |  | -11.444 | 2838.648 | <0.001 | 0.407 |
| Mean (SD) | 2.42 (1.22) | 2.93 (1.30) | 2.63 (1.28) |  |  |  |  |
| Median [Min, Max] | 2.00 [1.00, 5.00] | 3.00 [1.00, 5.00] | 2.00 [1.00, 5.00] |  |  |  |  |
| MHCS Nervousness |  |  |  | -12.734 | 2646.943 | <0.001 | 0.461 |
| Mean (SD) | 2.13 (1.15) | 2.70 (1.35) | 2.36 (1.27) |  |  |  |  |
| Median [Min, Max] | 2.00 [1.00, 5.00] | 3.00 [1.00, 5.00] | 2.00 [1.00, 5.00] |  |  |  |  |
| MHCS Problems falling asleep |  |  |  | -9.530 | 2854.547 | <0.001 | 0.339 |
| Mean (SD) | 2.44 (1.52) | 2.97 (1.60) | 2.66 (1.58) |  |  |  |  |
| Median [Min, Max] | 2.00 [1.00, 5.00] | 3.00 [1.00, 5.00] | 2.00 [1.00, 5.00] |  |  |  |  |
| MHCS Dizziness |  |  |  | -9.884 | 2507.441 | <0.001 | 0.362 |
| Mean (SD) | 1.66 (1.09) | 2.10 (1.38) | 1.84 (1.24) |  |  |  |  |
| Median [Min, Max] | 1.00 [1.00, 5.00] | 1.00 [1.00, 5.00] | 1.00 [1.00, 5.00] |  |  |  |  |
| WHO-5 Wellbeing 1 |  |  |  | 8.816 | 2817.597 | <0.001 | 0.314 |
| Mean (SD) | 3.62 (1.11) | 3.26 (1.19) | 3.47 (1.16) |  |  |  |  |
| Median [Min, Max] | 4.00 [0, 5.00] | 4.00 [0, 5.00] | 4.00 [0, 5.00] |  |  |  |  |
| WHO-5 Wellbeing 2 |  |  |  | 12.378 | 2816.340 | <0.001 | 0.441 |
| Mean (SD) | 3.32 (1.24) | 2.76 (1.33) | 3.09 (1.31) |  |  |  |  |
| Median [Min, Max] | 4.00 [0, 5.00] | 3.00 [0, 5.00] | 3.00 [0, 5.00] |  |  |  |  |
| WHO-5 Wellbeing 3 |  |  |  | 11.066 | 2738.865 | <0.001 | 0.397 |
| Mean (SD) | 3.57 (1.27) | 3.04 (1.42) | 3.35 (1.36) |  |  |  |  |
| Median [Min, Max] | 4.00 [0, 5.00] | 3.00 [0, 5.00] | 4.00 [0, 5.00] |  |  |  |  |
| WHO-5 Wellbeing 4 |  |  |  | 9.237 | 2930.299 | <0.001 | 0.326 |
| Mean (SD) | 2.87 (1.57) | 2.35 (1.59) | 2.66 (1.60) |  |  |  |  |
| Median [Min, Max] | 3.00 [0, 5.00] | 2.00 [0, 5.00] | 3.00 [0, 5.00] |  |  |  |  |
| WHO-5 Wellbeing 5 |  |  |  | 10.897 | 2756.367 | <0.001 | 0.391 |
| Mean (SD) | 3.46 (1.28) | 2.94 (1.42) | 3.24 (1.36) |  |  |  |  |
| Median [Min, Max] | 4.00 [0, 5.00] | 3.00 [0, 5.00] | 4.00 [0, 5.00] |  |  |  |  |
| WHO-5 Overall Wellbeing (Averaged) |  |  |  | 13.422 | 2750.134 | <0.001 | 0.482 |
| Mean (SD) | 3.37 (0.988) | 2.87 (1.10) | 3.16 (1.06) |  |  |  |  |
| Median [Min, Max] | 3.60 [0, 5.00] | 3.00 [0, 5.00] | 3.40 [0, 5.00] |  |  |  |  |
| Loneliness |  |  |  | -15.131 | 2816.469 | <0.001 | 0.540 |
| Mean (SD) | 1.94 (0.938) | 2.46 (1.01) | 2.16 (1.00) |  |  |  |  |
| Median [Min, Max] | 2.00 [1.00, 5.00] | 2.00 [1.00, 5.00] | 2.00 [1.00, 5.00] |  |  |  |  |

### GB-SCT

Descriptive statistics and gender differences in GB-SCT sample

|  | Boys (N=1126) | Girls (N=900) | Overall (N=2026) | t | df | p | d |
| --- | --- | --- | --- | --- | --- | --- | --- |
| IGDS Preoccupation |  |  |  | 13.417 | 1987.503 | <0.001 | 0.594 |
| Mean (SD) | 0.571 (0.495) | 0.288 (0.453) | 0.445 (0.497) |  |  |  |  |
| Median [Min, Max] | 1.00 [0, 1.00] | 0 [0, 1.00] | 0 [0, 1.00] |  |  |  |  |
| IGDS Tolerance |  |  |  | 8.466 | 2008.975 | <0.001 | 0.373 |
| Mean (SD) | 0.418 (0.493) | 0.244 (0.430) | 0.341 (0.474) |  |  |  |  |
| Median [Min, Max] | 0 [0, 1.00] | 0 [0, 1.00] | 0 [0, 1.00] |  |  |  |  |
| IGDS Withdrawal |  |  |  | 3.307 | 2004.040 | <0.001 | 0.146 |
| Mean (SD) | 0.206 (0.405) | 0.150 (0.357) | 0.181 (0.385) |  |  |  |  |
| Median [Min, Max] | 0 [0, 1.00] | 0 [0, 1.00] | 0 [0, 1.00] |  |  |  |  |
| IGDS Persistence |  |  |  | 4.142 | 2001.897 | <0.001 | 0.183 |
| Mean (SD) | 0.268 (0.443) | 0.191 (0.393) | 0.234 (0.423) |  |  |  |  |
| Median [Min, Max] | 0 [0, 1.00] | 0 [0, 1.00] | 0 [0, 1.00] |  |  |  |  |
| IGDS Escape |  |  |  | 7.878 | 1909.800 | <0.001 | 0.353 |
| Mean (SD) | 0.615 (0.487) | 0.441 (0.497) | 0.538 (0.499) |  |  |  |  |
| Median [Min, Max] | 1.00 [0, 1.00] | 0 [0, 1.00] | 1.00 [0, 1.00] |  |  |  |  |
| IGDS Problems |  |  |  | 10.473 | 1977.868 | <0.001 | 0.450 |
| Mean (SD) | 0.282 (0.450) | 0.106 (0.307) | 0.204 (0.403) |  |  |  |  |
| Median [Min, Max] | 0 [0, 1.00] | 0 [0, 1.00] | 0 [0, 1.00] |  |  |  |  |
| IGDS Deception |  |  |  | 1.852 | 1990.742 | 0.064 | 0.082 |
| Mean (SD) | 0.136 (0.343) | 0.109 (0.312) | 0.124 (0.330) |  |  |  |  |
| Median [Min, Max] | 0 [0, 1.00] | 0 [0, 1.00] | 0 [0, 1.00] |  |  |  |  |
| IGDS Displacement |  |  |  | 2.677 | 2014.597 | 0.007 | 0.118 |
| Mean (SD) | 0.122 (0.327) | 0.0856 (0.280) | 0.106 (0.307) |  |  |  |  |
| Median [Min, Max] | 0 [0, 1.00] | 0 [0, 1.00] | 0 [0, 1.00] |  |  |  |  |
| IGDS Conflict |  |  |  | 4.725 | 2009.754 | <0.001 | 0.204 |
| Mean (SD) | 0.119 (0.324) | 0.0600 (0.238) | 0.0928 (0.290) |  |  |  |  |
| Median [Min, Max] | 0 [0, 1.00] | 0 [0, 1.00] | 0 [0, 1.00] |  |  |  |  |
| SMDS Preoccupation |  |  |  | -6.095 | 1789.914 | <0.001 | 0.277 |
| Mean (SD) | 0.214 (0.410) | 0.336 (0.472) | 0.268 (0.443) |  |  |  |  |
| Median [Min, Max] | 0 [0, 1.00] | 0 [0, 1.00] | 0 [0, 1.00] |  |  |  |  |
| SMDS Tolerance |  |  |  | -8.303 | 1722.990 | <0.001 | 0.380 |
| Mean (SD) | 0.187 (0.390) | 0.351 (0.478) | 0.260 (0.439) |  |  |  |  |
| Median [Min, Max] | 0 [0, 1.00] | 0 [0, 1.00] | 0 [0, 1.00] |  |  |  |  |
| SMDS Withdrawal |  |  |  | -7.859 | 1743.005 | <0.001 | 0.359 |
| Mean (SD) | 0.197 (0.398) | 0.353 (0.478) | 0.267 (0.442) |  |  |  |  |
| Median [Min, Max] | 0 [0, 1.00] | 0 [0, 1.00] | 0 [0, 1.00] |  |  |  |  |
| SMDS Persistence |  |  |  | -12.463 | 1829.314 | <0.001 | 0.564 |
| Mean (SD) | 0.280 (0.449) | 0.546 (0.498) | 0.398 (0.490) |  |  |  |  |
| Median [Min, Max] | 0 [0, 1.00] | 1.00 [0, 1.00] | 0 [0, 1.00] |  |  |  |  |
| SMDS Escape |  |  |  | -11.909 | 1960.738 | <0.001 | 0.530 |
| Mean (SD) | 0.413 (0.493) | 0.669 (0.471) | 0.527 (0.499) |  |  |  |  |
| Median [Min, Max] | 0 [0, 1.00] | 1.00 [0, 1.00] | 1.00 [0, 1.00] |  |  |  |  |
| SMDS Problems |  |  |  | -6.001 | 1772.680 | <0.001 | 0.273 |
| Mean (SD) | 0.194 (0.396) | 0.311 (0.463) | 0.246 (0.431) |  |  |  |  |
| Median [Min, Max] | 0 [0, 1.00] | 0 [0, 1.00] | 0 [0, 1.00] |  |  |  |  |
| SMDS Deception |  |  |  | -6.339 | 1691.167 | <0.001 | 0.291 |
| Mean (SD) | 0.137 (0.344) | 0.249 (0.433) | 0.187 (0.390) |  |  |  |  |
| Median [Min, Max] | 0 [0, 1.00] | 0 [0, 1.00] | 0 [0, 1.00] |  |  |  |  |
| SMDS Displacement |  |  |  | -8.218 | 1646.018 | <0.001 | 0.378 |
| Mean (SD) | 0.139 (0.347) | 0.290 (0.454) | 0.206 (0.405) |  |  |  |  |
| Median [Min, Max] | 0 [0, 1.00] | 0 [0, 1.00] | 0 [0, 1.00] |  |  |  |  |
| SMDS Conflict |  |  |  | -6.974 | 1544.712 | <0.001 | 0.324 |
| Mean (SD) | 0.0799 (0.271) | 0.187 (0.390) | 0.127 (0.333) |  |  |  |  |
| Median [Min, Max] | 0 [0, 1.00] | 0 [0, 1.00] | 0 [0, 1.00] |  |  |  |  |
| MHCS Headache |  |  |  | -14.322 | 1768.100 | <0.001 | 0.652 |
| Mean (SD) | 2.07 (1.12) | 2.86 (1.31) | 2.42 (1.27) |  |  |  |  |
| Median [Min, Max] | 2.00 [1.00, 5.00] | 3.00 [1.00, 5.00] | 2.00 [1.00, 5.00] |  |  |  |  |
| MHCS Stomach ache |  |  |  | -16.103 | 1686.578 | <0.001 | 0.739 |
| Mean (SD) | 1.75 (0.969) | 2.55 (1.22) | 2.10 (1.16) |  |  |  |  |
| Median [Min, Max] | 1.00 [1.00, 5.00] | 2.00 [1.00, 5.00] | 2.00 [1.00, 5.00] |  |  |  |  |
| MHCS Back pain |  |  |  | -5.938 | 1780.536 | <0.001 | 0.270 |
| Mean (SD) | 1.92 (1.22) | 2.28 (1.42) | 2.08 (1.33) |  |  |  |  |
| Median [Min, Max] | 1.00 [1.00, 5.00] | 2.00 [1.00, 5.00] | 2.00 [1.00, 5.00] |  |  |  |  |
| MHCS Feeling low |  |  |  | -15.793 | 1900.398 | <0.001 | 0.709 |
| Mean (SD) | 2.26 (1.29) | 3.19 (1.33) | 2.67 (1.39) |  |  |  |  |
| Median [Min, Max] | 2.00 [1.00, 5.00] | 3.00 [1.00, 5.00] | 2.00 [1.00, 5.00] |  |  |  |  |
| MHCS Irritability |  |  |  | -9.935 | 1904.214 | <0.001 | 0.446 |
| Mean (SD) | 2.59 (1.38) | 3.21 (1.42) | 2.87 (1.43) |  |  |  |  |
| Median [Min, Max] | 2.00 [1.00, 5.00] | 3.00 [1.00, 5.00] | 3.00 [1.00, 5.00] |  |  |  |  |
| MHCS Nervousness |  |  |  | -18.700 | 1946.869 | <0.001 | 0.834 |
| Mean (SD) | 2.56 (1.33) | 3.66 (1.30) | 3.05 (1.43) |  |  |  |  |
| Median [Min, Max] | 2.00 [1.00, 5.00] | 4.00 [1.00, 5.00] | 3.00 [1.00, 5.00] |  |  |  |  |
| MHCS Problems falling asleep |  |  |  | -9.386 | 1903.227 | <0.001 | 0.421 |
| Mean (SD) | 2.52 (1.50) | 3.16 (1.54) | 2.81 (1.55) |  |  |  |  |
| Median [Min, Max] | 2.00 [1.00, 5.00] | 3.00 [1.00, 5.00] | 3.00 [1.00, 5.00] |  |  |  |  |
| MHCS Dizziness |  |  |  | -15.798 | 1691.063 | <0.001 | 0.724 |
| Mean (SD) | 1.83 (1.18) | 2.80 (1.49) | 2.26 (1.41) |  |  |  |  |
| Median [Min, Max] | 1.00 [1.00, 5.00] | 3.00 [1.00, 5.00] | 2.00 [1.00, 5.00] |  |  |  |  |
| WHO-5 Wellbeing 1 |  |  |  | 11.012 | 1911.773 | <0.001 | 0.493 |
| Mean (SD) | 3.18 (1.22) | 2.58 (1.24) | 2.92 (1.27) |  |  |  |  |
| Median [Min, Max] | 4.00 [0, 5.00] | 3.00 [0, 5.00] | 3.00 [0, 5.00] |  |  |  |  |
| WHO-5 Wellbeing 2 |  |  |  | 13.549 | 1939.578 | <0.001 | 0.605 |
| Mean (SD) | 3.03 (1.28) | 2.26 (1.26) | 2.69 (1.32) |  |  |  |  |
| Median [Min, Max] | 3.00 [0, 5.00] | 2.00 [0, 5.00] | 3.00 [0, 5.00] |  |  |  |  |
| WHO-5 Wellbeing 3 |  |  |  | 13.438 | 1917.217 | <0.001 | 0.602 |
| Mean (SD) | 3.16 (1.33) | 2.36 (1.34) | 2.81 (1.39) |  |  |  |  |
| Median [Min, Max] | 3.00 [0, 5.00] | 2.00 [0, 5.00] | 3.00 [0, 5.00] |  |  |  |  |
| WHO-5 Wellbeing 4 |  |  |  | 11.269 | 1965.994 | <0.001 | 0.501 |
| Mean (SD) | 2.49 (1.48) | 1.76 (1.41) | 2.17 (1.49) |  |  |  |  |
| Median [Min, Max] | 3.00 [0, 5.00] | 2.00 [0, 5.00] | 2.00 [0, 5.00] |  |  |  |  |
| WHO-5 Wellbeing 5 |  |  |  | 10.793 | 1842.985 | <0.001 | 0.487 |
| Mean (SD) | 3.31 (1.30) | 2.65 (1.42) | 3.01 (1.39) |  |  |  |  |
| Median [Min, Max] | 4.00 [0, 5.00] | 3.00 [0, 5.00] | 3.00 [0, 5.00] |  |  |  |  |
| WHO-5 Overall Wellbeing (Averaged) |  |  |  | 14.924 | 1900.485 | <0.001 | 0.670 |
| Mean (SD) | 3.03 (1.05) | 2.32 (1.08) | 2.72 (1.12) |  |  |  |  |
| Median [Min, Max] | 3.20 [0, 5.00] | 2.40 [0, 5.00] | 2.80 [0, 5.00] |  |  |  |  |
| Loneliness |  |  |  | -13.902 | 1928.578 | <0.001 | 0.621 |
| Mean (SD) | 2.37 (0.993) | 2.98 (0.991) | 2.64 (1.04) |  |  |  |  |
| Median [Min, Max] | 2.00 [1.00, 5.00] | 3.00 [1.00, 5.00] | 3.00 [1.00, 5.00] |  |  |  |  |

### SI

Descriptive statistics and gender differences in SI sample

|  | Boys (N=2249) | Girls (N=1844) | Overall (N=4093) | t | df | p | d |
| --- | --- | --- | --- | --- | --- | --- | --- |
| IGDS Preoccupation |  |  |  | 13.613 | 4090.909 | <0.001 | 0.419 |
| Mean (SD) | 0.384 (0.486) | 0.196 (0.397) | 0.299 (0.458) |  |  |  |  |
| Median [Min, Max] | 0 [0, 1.00] | 0 [0, 1.00] | 0 [0, 1.00] |  |  |  |  |
| IGDS Tolerance |  |  |  | 8.774 | 4088.481 | <0.001 | 0.270 |
| Mean (SD) | 0.241 (0.428) | 0.135 (0.342) | 0.193 (0.395) |  |  |  |  |
| Median [Min, Max] | 0 [0, 1.00] | 0 [0, 1.00] | 0 [0, 1.00] |  |  |  |  |
| IGDS Withdrawal |  |  |  | 7.889 | 4086.901 | <0.001 | 0.242 |
| Mean (SD) | 0.207 (0.405) | 0.117 (0.322) | 0.166 (0.372) |  |  |  |  |
| Median [Min, Max] | 0 [0, 1.00] | 0 [0, 1.00] | 0 [0, 1.00] |  |  |  |  |
| IGDS Persistence |  |  |  | 5.947 | 4088.889 | <0.001 | 0.184 |
| Mean (SD) | 0.192 (0.394) | 0.125 (0.331) | 0.162 (0.368) |  |  |  |  |
| Median [Min, Max] | 0 [0, 1.00] | 0 [0, 1.00] | 0 [0, 1.00] |  |  |  |  |
| IGDS Escape |  |  |  | 2.818 | 3984.447 | 0.005 | 0.088 |
| Mean (SD) | 0.334 (0.472) | 0.293 (0.455) | 0.316 (0.465) |  |  |  |  |
| Median [Min, Max] | 0 [0, 1.00] | 0 [0, 1.00] | 0 [0, 1.00] |  |  |  |  |
| IGDS Problems |  |  |  | 6.841 | 4087.813 | <0.001 | 0.210 |
| Mean (SD) | 0.176 (0.381) | 0.102 (0.303) | 0.143 (0.350) |  |  |  |  |
| Median [Min, Max] | 0 [0, 1.00] | 0 [0, 1.00] | 0 [0, 1.00] |  |  |  |  |
| IGDS Deception |  |  |  | 5.908 | 4090.980 | <0.001 | 0.182 |
| Mean (SD) | 0.169 (0.375) | 0.106 (0.308) | 0.141 (0.348) |  |  |  |  |
| Median [Min, Max] | 0 [0, 1.00] | 0 [0, 1.00] | 0 [0, 1.00] |  |  |  |  |
| IGDS Displacement |  |  |  | 2.975 | 4065.918 | 0.003 | 0.092 |
| Mean (SD) | 0.120 (0.325) | 0.0911 (0.288) | 0.107 (0.309) |  |  |  |  |
| Median [Min, Max] | 0 [0, 1.00] | 0 [0, 1.00] | 0 [0, 1.00] |  |  |  |  |
| IGDS Conflict |  |  |  | 5.926 | 4072.036 | <0.001 | 0.181 |
| Mean (SD) | 0.118 (0.322) | 0.0651 (0.247) | 0.0941 (0.292) |  |  |  |  |
| Median [Min, Max] | 0 [0, 1.00] | 0 [0, 1.00] | 0 [0, 1.00] |  |  |  |  |
| SMDS Preoccupation |  |  |  | -5.238 | 3699.489 | <0.001 | 0.167 |
| Mean (SD) | 0.161 (0.368) | 0.227 (0.419) | 0.191 (0.393) |  |  |  |  |
| Median [Min, Max] | 0 [0, 1.00] | 0 [0, 1.00] | 0 [0, 1.00] |  |  |  |  |
| SMDS Tolerance |  |  |  | -3.691 | 3750.948 | <0.001 | 0.117 |
| Mean (SD) | 0.146 (0.353) | 0.190 (0.392) | 0.166 (0.372) |  |  |  |  |
| Median [Min, Max] | 0 [0, 1.00] | 0 [0, 1.00] | 0 [0, 1.00] |  |  |  |  |
| SMDS Withdrawal |  |  |  | -3.135 | 3788.339 | 0.002 | 0.099 |
| Mean (SD) | 0.156 (0.363) | 0.194 (0.395) | 0.173 (0.378) |  |  |  |  |
| Median [Min, Max] | 0 [0, 1.00] | 0 [0, 1.00] | 0 [0, 1.00] |  |  |  |  |
| SMDS Persistence |  |  |  | -8.398 | 3777.758 | <0.001 | 0.266 |
| Mean (SD) | 0.278 (0.448) | 0.402 (0.491) | 0.334 (0.472) |  |  |  |  |
| Median [Min, Max] | 0 [0, 1.00] | 0 [0, 1.00] | 0 [0, 1.00] |  |  |  |  |
| SMDS Escape |  |  |  | -11.075 | 3740.649 | <0.001 | 0.352 |
| Mean (SD) | 0.271 (0.445) | 0.436 (0.496) | 0.345 (0.476) |  |  |  |  |
| Median [Min, Max] | 0 [0, 1.00] | 0 [0, 1.00] | 0 [0, 1.00] |  |  |  |  |
| SMDS Problems |  |  |  | -5.093 | 3665.570 | <0.001 | 0.162 |
| Mean (SD) | 0.137 (0.344) | 0.197 (0.398) | 0.164 (0.370) |  |  |  |  |
| Median [Min, Max] | 0 [0, 1.00] | 0 [0, 1.00] | 0 [0, 1.00] |  |  |  |  |
| SMDS Deception |  |  |  | -4.696 | 3694.864 | <0.001 | 0.149 |
| Mean (SD) | 0.142 (0.349) | 0.198 (0.399) | 0.167 (0.373) |  |  |  |  |
| Median [Min, Max] | 0 [0, 1.00] | 0 [0, 1.00] | 0 [0, 1.00] |  |  |  |  |
| SMDS Displacement |  |  |  | -3.699 | 3745.665 | <0.001 | 0.117 |
| Mean (SD) | 0.142 (0.349) | 0.185 (0.389) | 0.162 (0.368) |  |  |  |  |
| Median [Min, Max] | 0 [0, 1.00] | 0 [0, 1.00] | 0 [0, 1.00] |  |  |  |  |
| SMDS Conflict |  |  |  | -3.429 | 3753.376 | <0.001 | 0.109 |
| Mean (SD) | 0.137 (0.344) | 0.176 (0.381) | 0.155 (0.362) |  |  |  |  |
| Median [Min, Max] | 0 [0, 1.00] | 0 [0, 1.00] | 0 [0, 1.00] |  |  |  |  |
| MHCS Headache |  |  |  | -13.231 | 3389.769 | <0.001 | 0.427 |
| Mean (SD) | 1.62 (0.958) | 2.09 (1.26) | 1.83 (1.13) |  |  |  |  |
| Median [Min, Max] | 1.00 [1.00, 5.00] | 2.00 [1.00, 5.00] | 1.00 [1.00, 5.00] |  |  |  |  |
| MHCS Stomach ache |  |  |  | -13.819 | 3407.614 | <0.001 | 0.445 |
| Mean (SD) | 1.48 (0.830) | 1.90 (1.08) | 1.67 (0.973) |  |  |  |  |
| Median [Min, Max] | 1.00 [1.00, 5.00] | 2.00 [1.00, 5.00] | 1.00 [1.00, 5.00] |  |  |  |  |
| MHCS Back pain |  |  |  | -5.020 | 3658.987 | <0.001 | 0.160 |
| Mean (SD) | 1.66 (1.09) | 1.85 (1.26) | 1.75 (1.17) |  |  |  |  |
| Median [Min, Max] | 1.00 [1.00, 5.00] | 1.00 [1.00, 5.00] | 1.00 [1.00, 5.00] |  |  |  |  |
| MHCS Feeling low |  |  |  | -20.213 | 3474.590 | <0.001 | 0.650 |
| Mean (SD) | 1.75 (1.13) | 2.57 (1.43) | 2.12 (1.34) |  |  |  |  |
| Median [Min, Max] | 1.00 [1.00, 5.00] | 2.00 [1.00, 5.00] | 2.00 [1.00, 5.00] |  |  |  |  |
| MHCS Irritability |  |  |  | -15.804 | 3743.503 | <0.001 | 0.502 |
| Mean (SD) | 2.14 (1.17) | 2.75 (1.30) | 2.42 (1.27) |  |  |  |  |
| Median [Min, Max] | 2.00 [1.00, 5.00] | 3.00 [1.00, 5.00] | 2.00 [1.00, 5.00] |  |  |  |  |
| MHCS Nervousness |  |  |  | -15.280 | 3714.575 | <0.001 | 0.486 |
| Mean (SD) | 2.29 (1.23) | 2.93 (1.39) | 2.58 (1.35) |  |  |  |  |
| Median [Min, Max] | 2.00 [1.00, 5.00] | 3.00 [1.00, 5.00] | 2.00 [1.00, 5.00] |  |  |  |  |
| MHCS Problems falling asleep |  |  |  | -13.226 | 3736.831 | <0.001 | 0.420 |
| Mean (SD) | 2.18 (1.36) | 2.78 (1.52) | 2.45 (1.46) |  |  |  |  |
| Median [Min, Max] | 2.00 [1.00, 5.00] | 3.00 [1.00, 5.00] | 2.00 [1.00, 5.00] |  |  |  |  |
| MHCS Dizziness |  |  |  | -11.953 | 3241.574 | <0.001 | 0.388 |
| Mean (SD) | 1.35 (0.856) | 1.75 (1.20) | 1.53 (1.04) |  |  |  |  |
| Median [Min, Max] | 1.00 [1.00, 5.00] | 1.00 [1.00, 5.00] | 1.00 [1.00, 5.00] |  |  |  |  |
| WHO-5 Wellbeing 1 |  |  |  | 12.235 | 3779.496 | <0.001 | 0.388 |
| Mean (SD) | 3.33 (1.26) | 2.82 (1.38) | 3.10 (1.34) |  |  |  |  |
| Median [Min, Max] | 4.00 [0, 5.00] | 3.00 [0, 5.00] | 4.00 [0, 5.00] |  |  |  |  |
| WHO-5 Wellbeing 2 |  |  |  | 14.810 | 3887.530 | <0.001 | 0.467 |
| Mean (SD) | 2.93 (1.40) | 2.27 (1.44) | 2.64 (1.46) |  |  |  |  |
| Median [Min, Max] | 3.00 [0, 5.00] | 2.00 [0, 5.00] | 3.00 [0, 5.00] |  |  |  |  |
| WHO-5 Wellbeing 3 |  |  |  | 13.655 | 3813.149 | <0.001 | 0.432 |
| Mean (SD) | 3.14 (1.42) | 2.51 (1.52) | 2.85 (1.50) |  |  |  |  |
| Median [Min, Max] | 3.00 [0, 5.00] | 3.00 [0, 5.00] | 3.00 [0, 5.00] |  |  |  |  |
| WHO-5 Wellbeing 4 |  |  |  | 11.567 | 3979.928 | <0.001 | 0.362 |
| Mean (SD) | 2.44 (1.66) | 1.85 (1.60) | 2.18 (1.66) |  |  |  |  |
| Median [Min, Max] | 2.00 [0, 5.00] | 1.00 [0, 5.00] | 2.00 [0, 5.00] |  |  |  |  |
| WHO-5 Wellbeing 5 |  |  |  | 11.761 | 3915.856 | <0.001 | 0.370 |
| Mean (SD) | 2.78 (1.48) | 2.23 (1.50) | 2.53 (1.51) |  |  |  |  |
| Median [Min, Max] | 3.00 [0, 5.00] | 2.00 [0, 5.00] | 3.00 [0, 5.00] |  |  |  |  |
| WHO-5 Overall Wellbeing (Averaged) |  |  |  | 16.063 | 3825.464 | <0.001 | 0.508 |
| Mean (SD) | 2.93 (1.13) | 2.34 (1.20) | 2.66 (1.20) |  |  |  |  |
| Median [Min, Max] | 3.00 [0, 5.00] | 2.40 [0, 5.00] | 2.80 [0, 5.00] |  |  |  |  |
| Loneliness |  |  |  | -16.623 | 3815.195 | <0.001 | 0.526 |
| Mean (SD) | 2.02 (1.03) | 2.58 (1.10) | 2.28 (1.10) |  |  |  |  |
| Median [Min, Max] | 2.00 [1.00, 5.00] | 3.00 [1.00, 5.00] | 2.00 [1.00, 5.00] |  |  |  |  |

# Correlation Analysis

## Corr. Coefs.

## Corr. Diffs.

# Network Analysis

## Figure 3

### Overall

#### Figure

#### Edges

##### IGDS

```
##      From    To Weight_Boys Weight_Girls
## 1     Pbl   Cfl       0.599        0.878
## 2     Tol   Wth       0.627        0.691
## 3     Pre   Tol       0.500        0.607
## 4     Pre   Wth       0.429        0.515
## 5     Dec   Cfl       0.483        0.391
## 6     Pbl   Dis       0.386        0.479
## 7     Pbl   Dec       0.287        0.438
## 8     Dis   Cfl       0.438        0.327
## 9     Pre   Esc       0.407        0.436
## 10    Dec   Dis       0.408        0.371
## 11    Prs   Dis       0.380        0.391
## 12    Tol   Dis       0.278        0.359
## 13    Prs   Dec       0.310        0.352
## 14    Tol   Esc       0.145        0.345
## 15    Prs   Pbl       0.281        0.345
## 16    Esc   Dec       0.261        0.328
## 17    Wth   Cfl       0.324        0.287
## 18    Tol   Pbl       0.314        0.264
## 19    Pre   Prs       0.182        0.312
## 20    Wth   Dec       0.306        0.295
## 21    Wth   Dis       0.291        0.284
## 22   Lone    WB      -0.241       -0.275
## 23    Tol   Prs       0.274        0.237
## 24    Prs   Esc       0.179        0.270
## 25   Irri  Nerv       0.260        0.266
## 26    Wth   Esc       0.214        0.262
## 27    Pre   Dis       0.149        0.258
## 28    Low  Lone       0.257        0.244
## 29    Low  Irri       0.236        0.245
## 30   Head Dizzy       0.201        0.241
## 31    Prs   Cfl       0.234        0.211
## 32    Esc   Pbl       0.187        0.234
## 33   Head  Stom       0.226        0.196
## 34    Pre   Pbl       0.214        0.198
## 35    Pre   Dec       0.189        0.207
## 36    Esc   Cfl       0.172        0.206
## 37    Pre   Cfl       0.124        0.187
## 38   Stom Dizzy       0.149        0.186
## 39    Wth   Prs       0.184        0.175
## 40    Low  Nerv       0.155        0.176
## 41    Tol   Dec       0.171        0.152
## 42    Low    WB      -0.120       -0.163
## 43  Sleep Dizzy       0.134        0.151
## 44    Wth   Pbl       0.149        0.144
## 45   Head  Back       0.126        0.148
## 46    Esc   Dis       0.000        0.141
## 47   Lone   Esc       0.119        0.133
## 48  Sleep    WB      -0.123       -0.123
## 49     WB   Dis       0.000       -0.123
## 50    Low Dizzy       0.085        0.116
## 51   Irri    WB      -0.114       -0.114
## 52  Sleep   Dec       0.000        0.108
## 53    Tol   Cfl       0.107        0.060
## 54   Stom  Back       0.091        0.099
## 55   Irri   Pbl       0.090        0.027
## 56   Head Sleep       0.089        0.063
## 57   Head   Low       0.077        0.088
## 58   Back Dizzy       0.088        0.082
## 59   Nerv Sleep       0.067        0.086
## 60   Nerv  Lone       0.070        0.078
## 61   Irri Sleep       0.067        0.070
## 62   Stom   Low       0.048        0.068
## 63   Nerv    WB      -0.066       -0.068
## 64    Low   Esc       0.067        0.048
## 65   Back  Irri       0.064        0.049
## 66   Stom  Nerv       0.051        0.063
## 67   Back   Low       0.063        0.047
## 68   Head  Nerv       0.062        0.056
## 69   Back Sleep       0.062        0.035
## 70  Sleep   Esc       0.062        0.055
## 71   Nerv   Esc       0.061        0.044
## 72  Dizzy   Cfl       0.061        0.000
## 73   Stom Sleep       0.059        0.042
## 74     WB   Esc      -0.053       -0.054
## 75   Back  Nerv       0.046        0.052
## 76  Sleep  Lone       0.052        0.048
## 77    Low Sleep       0.048        0.051
## 78   Nerv Dizzy       0.039        0.051
## 79   Irri  Lone       0.043        0.046
## 80   Head  Irri       0.043        0.044
## 81     WB   Tol      -0.042        0.000
## 82   Back   Dec       0.041        0.000
## 83   Stom  Irri       0.038        0.022
## 84   Irri   Wth       0.035        0.000
## 85   Nerv   Pre       0.034        0.000
## 86  Dizzy   Tol       0.000        0.034
## 87    Low   Cfl       0.028        0.000
## 88  Sleep   Pbl       0.000        0.028
## 89  Dizzy  Lone       0.028        0.018
## 90  Dizzy   Dis       0.000        0.027
## 91    Low   Tol       0.026        0.000
## 92   Irri Dizzy       0.026        0.000
## 93   Back   Dis       0.000        0.023
## 94   Lone   Dis       0.023        0.000
## 95  Sleep   Wth       0.022        0.000
## 96   Back  Lone       0.021        0.000
## 97  Sleep   Dis       0.021        0.000
## 98   Head    WB       0.000       -0.020
## 99   Back    WB      -0.019        0.000
## 100  Lone   Tol       0.019        0.000
## 101  Lone   Dec       0.019        0.000
## 102    WB   Pre      -0.019        0.000
## 103  Lone   Pre       0.018        0.000
## 104 Dizzy   Dec       0.016        0.000
## 105  Head  Lone       0.014        0.000
## 106  Head   Pre       0.000        0.000
## 107  Head   Tol       0.000        0.000
## 108  Head   Wth       0.000        0.000
## 109  Head   Prs       0.000        0.000
## 110  Head   Esc       0.000        0.000
## 111  Head   Pbl       0.000        0.000
## 112  Head   Dec       0.000        0.000
## 113  Head   Dis       0.000        0.000
## 114  Head   Cfl       0.000        0.000
## 115  Stom  Lone       0.000        0.000
## 116  Stom    WB       0.000        0.000
## 117  Stom   Pre       0.000        0.000
## 118  Stom   Tol       0.000        0.000
## 119  Stom   Wth       0.000        0.000
## 120  Stom   Prs       0.000        0.000
## 121  Stom   Esc       0.000        0.000
## 122  Stom   Pbl       0.000        0.000
## 123  Stom   Dec       0.000        0.000
## 124  Stom   Dis       0.000        0.000
## 125  Stom   Cfl       0.000        0.000
## 126  Back   Pre       0.000        0.000
## 127  Back   Tol       0.000        0.000
## 128  Back   Wth       0.000        0.000
## 129  Back   Prs       0.000        0.000
## 130  Back   Esc       0.000        0.000
## 131  Back   Pbl       0.000        0.000
## 132  Back   Cfl       0.000        0.000
## 133   Low   Pre       0.000        0.000
## 134   Low   Wth       0.000        0.000
## 135   Low   Prs       0.000        0.000
## 136   Low   Pbl       0.000        0.000
## 137   Low   Dec       0.000        0.000
## 138   Low   Dis       0.000        0.000
## 139  Irri   Pre       0.000        0.000
## 140  Irri   Tol       0.000        0.000
## 141  Irri   Prs       0.000        0.000
## 142  Irri   Esc       0.000        0.000
## 143  Irri   Dec       0.000        0.000
## 144  Irri   Dis       0.000        0.000
## 145  Irri   Cfl       0.000        0.000
## 146  Nerv   Tol       0.000        0.000
## 147  Nerv   Wth       0.000        0.000
## 148  Nerv   Prs       0.000        0.000
## 149  Nerv   Pbl       0.000        0.000
## 150  Nerv   Dec       0.000        0.000
## 151  Nerv   Dis       0.000        0.000
## 152  Nerv   Cfl       0.000        0.000
## 153 Sleep   Pre       0.000        0.000
## 154 Sleep   Tol       0.000        0.000
## 155 Sleep   Prs       0.000        0.000
## 156 Sleep   Cfl       0.000        0.000
## 157 Dizzy    WB       0.000        0.000
## 158 Dizzy   Pre       0.000        0.000
## 159 Dizzy   Wth       0.000        0.000
## 160 Dizzy   Prs       0.000        0.000
## 161 Dizzy   Esc       0.000        0.000
## 162 Dizzy   Pbl       0.000        0.000
## 163  Lone   Wth       0.000        0.000
## 164  Lone   Prs       0.000        0.000
## 165  Lone   Pbl       0.000        0.000
## 166  Lone   Cfl       0.000        0.000
## 167    WB   Wth       0.000        0.000
## 168    WB   Prs       0.000        0.000
## 169    WB   Pbl       0.000        0.000
## 170    WB   Dec       0.000        0.000
## 171    WB   Cfl       0.000        0.000
```

##### SMDS

```
##      From    To Weight_Boys Weight_Girls
## 1     Dec   Cfl       0.681        0.516
## 2     Tol   Wth       0.620        0.670
## 3     Pbl   Cfl       0.597        0.650
## 4     Pre   Tol       0.510        0.604
## 5     Pre   Wth       0.470        0.431
## 6     Dec   Dis       0.375        0.221
## 7     Pbl   Dec       0.282        0.359
## 8     Dis   Cfl       0.353        0.167
## 9     Prs   Esc       0.352        0.350
## 10    Tol   Pbl       0.344        0.248
## 11    Esc   Pbl       0.335        0.225
## 12    Wth   Dec       0.311        0.198
## 13    Tol   Esc       0.304        0.195
## 14    Prs   Dis       0.205        0.285
## 15    Prs   Dec       0.283        0.183
## 16    Pbl   Dis       0.274        0.228
## 17    Wth   Cfl       0.266        0.197
## 18   Lone    WB      -0.244       -0.265
## 19   Irri  Nerv       0.259        0.261
## 20    Pre   Dis       0.255        0.186
## 21    Low  Lone       0.253        0.240
## 22    Wth   Esc       0.251        0.233
## 23    Prs   Pbl       0.248        0.193
## 24    Wth   Pbl       0.244        0.192
## 25   Head Dizzy       0.197        0.242
## 26    Low  Irri       0.235        0.241
## 27    Tol   Prs       0.240        0.154
## 28    Pre   Esc       0.188        0.237
## 29    Wth   Dis       0.232        0.227
## 30   Head  Stom       0.225        0.200
## 31    Pre   Prs       0.189        0.206
## 32    Tol   Cfl       0.203        0.170
## 33    Pre   Cfl       0.199        0.115
## 34    Pre   Dec       0.198        0.162
## 35   Stom Dizzy       0.145        0.187
## 36    Esc   Dis       0.112        0.185
## 37    Esc   Dec       0.115        0.184
## 38    Low  Nerv       0.153        0.170
## 39    Tol   Dis       0.160        0.110
## 40    Low    WB      -0.121       -0.158
## 41   Lone   Esc       0.154        0.140
## 42  Sleep Dizzy       0.131        0.150
## 43   Head  Back       0.123        0.148
## 44    Esc   Cfl       0.135        0.127
## 45  Sleep    WB      -0.127       -0.120
## 46     WB   Esc      -0.036       -0.121
## 47    Pre   Pbl       0.096        0.121
## 48    Prs   Cfl       0.121        0.109
## 49   Irri    WB      -0.118       -0.109
## 50    Tol   Dec       0.113        0.116
## 51    Low Dizzy       0.085        0.113
## 52    Low   Esc       0.105        0.110
## 53   Nerv   Esc       0.089        0.109
## 54   Stom  Back       0.089        0.101
## 55   Head   Low       0.077        0.089
## 56   Head Sleep       0.088        0.063
## 57   Nerv Sleep       0.068        0.085
## 58   Back Dizzy       0.083        0.082
## 59   Nerv  Lone       0.066        0.071
## 60   Irri Sleep       0.070        0.070
## 61   Stom   Low       0.048        0.067
## 62   Nerv    WB      -0.067       -0.061
## 63   Back  Irri       0.065        0.048
## 64   Back   Low       0.063        0.049
## 65   Back Sleep       0.062        0.031
## 66   Head  Nerv       0.061        0.056
## 67   Stom  Nerv       0.050        0.061
## 68    Wth   Prs       0.058        0.061
## 69   Stom Sleep       0.059        0.043
## 70   Irri   Pbl       0.057        0.058
## 71   Irri   Cfl       0.022        0.057
## 72  Sleep  Lone       0.056        0.050
## 73    Low Sleep       0.049        0.054
## 74   Back  Nerv       0.045        0.049
## 75     WB   Dis      -0.049       -0.031
## 76   Nerv Dizzy       0.035        0.048
## 77  Sleep   Dec       0.022        0.048
## 78   Head  Irri       0.043        0.045
## 79   Back   Dec       0.045        0.000
## 80   Irri  Lone       0.043        0.042
## 81   Lone   Cfl       0.000        0.041
## 82     WB   Cfl       0.000       -0.041
## 83   Stom  Irri       0.040        0.022
## 84   Nerv   Pbl       0.000        0.038
## 85  Dizzy   Tol       0.000        0.038
## 86   Irri   Wth       0.014        0.037
## 87   Irri   Dec       0.033        0.037
## 88   Stom   Wth       0.000        0.035
## 89    Low   Tol       0.000        0.034
## 90    Low   Dis       0.000        0.034
## 91   Irri   Pre       0.000        0.034
## 92  Dizzy   Dec       0.000        0.034
## 93     WB   Dec       0.000       -0.033
## 94   Irri Dizzy       0.026        0.000
## 95   Stom   Pre       0.000        0.025
## 96  Dizzy   Pbl       0.000        0.025
## 97   Irri   Esc       0.021        0.023
## 98  Dizzy  Lone       0.023        0.000
## 99   Back  Lone       0.020        0.000
## 100  Nerv   Prs       0.020        0.000
## 101  Head    WB       0.000       -0.019
## 102  Back    WB      -0.019        0.000
## 103  Back   Pre       0.000        0.019
## 104  Back   Cfl       0.000        0.018
## 105 Sleep   Prs       0.000        0.016
## 106  Irri   Tol       0.015        0.000
## 107  Head  Lone       0.013        0.000
## 108  Head   Pre       0.000        0.000
## 109  Head   Tol       0.000        0.000
## 110  Head   Wth       0.000        0.000
## 111  Head   Prs       0.000        0.000
## 112  Head   Esc       0.000        0.000
## 113  Head   Pbl       0.000        0.000
## 114  Head   Dec       0.000        0.000
## 115  Head   Dis       0.000        0.000
## 116  Head   Cfl       0.000        0.000
## 117  Stom  Lone       0.000        0.000
## 118  Stom    WB       0.000        0.000
## 119  Stom   Tol       0.000        0.000
## 120  Stom   Prs       0.000        0.000
## 121  Stom   Esc       0.000        0.000
## 122  Stom   Pbl       0.000        0.000
## 123  Stom   Dec       0.000        0.000
## 124  Stom   Dis       0.000        0.000
## 125  Stom   Cfl       0.000        0.000
## 126  Back   Tol       0.000        0.000
## 127  Back   Wth       0.000        0.000
## 128  Back   Prs       0.000        0.000
## 129  Back   Esc       0.000        0.000
## 130  Back   Pbl       0.000        0.000
## 131  Back   Dis       0.000        0.000
## 132   Low   Pre       0.000        0.000
## 133   Low   Wth       0.000        0.000
## 134   Low   Prs       0.000        0.000
## 135   Low   Pbl       0.000        0.000
## 136   Low   Dec       0.000        0.000
## 137   Low   Cfl       0.000        0.000
## 138  Irri   Prs       0.000        0.000
## 139  Irri   Dis       0.000        0.000
## 140  Nerv   Pre       0.000        0.000
## 141  Nerv   Tol       0.000        0.000
## 142  Nerv   Wth       0.000        0.000
## 143  Nerv   Dec       0.000        0.000
## 144  Nerv   Dis       0.000        0.000
## 145  Nerv   Cfl       0.000        0.000
## 146 Sleep   Pre       0.000        0.000
## 147 Sleep   Tol       0.000        0.000
## 148 Sleep   Wth       0.000        0.000
## 149 Sleep   Esc       0.000        0.000
## 150 Sleep   Pbl       0.000        0.000
## 151 Sleep   Dis       0.000        0.000
## 152 Sleep   Cfl       0.000        0.000
## 153 Dizzy    WB       0.000        0.000
## 154 Dizzy   Pre       0.000        0.000
## 155 Dizzy   Wth       0.000        0.000
## 156 Dizzy   Prs       0.000        0.000
## 157 Dizzy   Esc       0.000        0.000
## 158 Dizzy   Dis       0.000        0.000
## 159 Dizzy   Cfl       0.000        0.000
## 160  Lone   Pre       0.000        0.000
## 161  Lone   Tol       0.000        0.000
## 162  Lone   Wth       0.000        0.000
## 163  Lone   Prs       0.000        0.000
## 164  Lone   Pbl       0.000        0.000
## 165  Lone   Dec       0.000        0.000
## 166  Lone   Dis       0.000        0.000
## 167    WB   Pre       0.000        0.000
## 168    WB   Tol       0.000        0.000
## 169    WB   Wth       0.000        0.000
## 170    WB   Prs       0.000        0.000
## 171    WB   Pbl       0.000        0.000
```

#### Centrality

##### IGDS

**The centrality estimates are relatively stable when the
correlation stability coefficient (CS coefficient) is ≥ 0.5**
while set at correlation stability = 0.7 (Epskamp et al.,
2018). Centrality indices with CS coefficient of 0.25-0.5 should be
interpreted with caution; CS coefficient < 0.25 indicated large
instability of the centrality index.

```
##   region scale group        centrality    CS_r07
## 1  TOTAL  IGDS  Boys          strength 0.7500000
## 2  TOTAL  IGDS  Boys         closeness 0.7500000
## 3  TOTAL  IGDS  Boys       betweenness 0.1277523
## 4  TOTAL  IGDS  Boys expectedInfluence 0.7500000
## 5  TOTAL  IGDS Girls          strength 0.7499672
## 6  TOTAL  IGDS Girls         closeness 0.7499672
## 7  TOTAL  IGDS Girls       betweenness 0.2833049
## 8  TOTAL  IGDS Girls expectedInfluence 0.7499672
```

##### SMDS

**The centrality estimates are relatively stable when the
correlation stability coefficient (CS coefficient) is ≥ 0.5**
while set at correlation stability = 0.7 (Epskamp et al.,
2018). Centrality indices with CS coefficient of 0.25-0.5 should be
interpreted with caution; CS coefficient < 0.25 indicated large
instability of the centrality index.

```
##    region scale group        centrality    CS_r07
## 9   TOTAL  SMDS  Boys          strength 0.7500000
## 10  TOTAL  SMDS  Boys         closeness 0.7500000
## 11  TOTAL  SMDS  Boys       betweenness 0.7500000
## 12  TOTAL  SMDS  Boys expectedInfluence 0.7500000
## 13  TOTAL  SMDS Girls          strength 0.7499672
## 14  TOTAL  SMDS Girls         closeness 0.7499672
## 15  TOTAL  SMDS Girls       betweenness 0.7499672
## 16  TOTAL  SMDS Girls expectedInfluence 0.7499672
```

#### Bootstrapped Edges

##### IGDS

##### SMDS

### CY

#### Figure

#### Edges

##### IGDS

```
##      From    To Weight_Boys Weight_Girls
## 1     Tol   Wth       0.728        0.690
## 2     Pbl   Cfl       0.471        0.550
## 3     Pre   Wth       0.331        0.544
## 4     Dec   Cfl       0.514        0.217
## 5    Irri  Nerv       0.513        0.503
## 6     Pbl   Dis       0.142        0.498
## 7     Dis   Cfl       0.484        0.390
## 8     Prs   Dis       0.468        0.360
## 9     Dec   Dis       0.341        0.443
## 10    Pre   Esc       0.438        0.342
## 11    Prs   Pbl       0.418        0.370
## 12    Tol   Esc       0.155        0.412
## 13    Tol   Pbl       0.391        0.320
## 14    Pre   Tol       0.382        0.365
## 15    Prs   Dec       0.200        0.381
## 16    Pbl   Dec       0.359        0.360
## 17    Prs   Esc       0.202        0.344
## 18   Lone    WB      -0.241       -0.339
## 19    Wth   Dec       0.316        0.188
## 20    Wth   Cfl       0.304        0.313
## 21    Wth   Prs       0.311        0.271
## 22   Head Dizzy       0.309        0.310
## 23    Pre   Prs       0.133        0.305
## 24    Esc   Cfl       0.251        0.305
## 25    Tol   Dis       0.302        0.148
## 26    Wth   Esc       0.156        0.290
## 27    Pre   Pbl       0.280        0.234
## 28    Esc   Dec       0.268        0.263
## 29    Prs   Cfl       0.197        0.228
## 30    Pre   Dec       0.098        0.220
## 31    Low    WB      -0.202       -0.217
## 32    Tol   Dec       0.100        0.216
## 33    Tol   Prs       0.119        0.204
## 34   Head  Stom       0.202        0.178
## 35    Pre   Cfl       0.140        0.194
## 36  Sleep Dizzy       0.116        0.175
## 37    Esc   Pbl       0.087        0.165
## 38    Low  Irri       0.135        0.125
## 39   Lone   Esc       0.134        0.000
## 40   Stom Dizzy       0.130        0.115
## 41    Low  Nerv       0.051        0.127
## 42   Head  Back       0.101        0.119
## 43   Stom  Back       0.000        0.116
## 44    Wth   Dis       0.082        0.116
## 45    Low  Lone       0.066        0.112
## 46   Nerv    WB      -0.082       -0.110
## 47    Wth   Pbl       0.000        0.108
## 48    Low Sleep       0.000        0.106
## 49    Tol   Cfl       0.104        0.000
## 50    Low Dizzy       0.000        0.100
## 51  Sleep  Lone       0.100        0.085
## 52   Head   Low       0.093        0.087
## 53  Sleep   Dec       0.093        0.000
## 54   Back   Low       0.091        0.000
## 55  Sleep    WB      -0.090        0.000
## 56   Back Dizzy       0.000        0.088
## 57   Nerv Sleep       0.000        0.075
## 58   Head    WB       0.000       -0.073
## 59   Stom  Nerv       0.000        0.071
## 60   Stom   Low       0.070        0.060
## 61  Dizzy   Dec       0.000        0.068
## 62   Back Sleep       0.063        0.057
## 63   Head Sleep       0.055        0.000
## 64   Stom Sleep       0.054        0.000
## 65    Low   Esc       0.047        0.000
## 66  Sleep   Esc       0.000        0.041
## 67   Head  Irri       0.000        0.000
## 68   Head  Nerv       0.000        0.000
## 69   Head  Lone       0.000        0.000
## 70   Head   Pre       0.000        0.000
## 71   Head   Tol       0.000        0.000
## 72   Head   Wth       0.000        0.000
## 73   Head   Prs       0.000        0.000
## 74   Head   Esc       0.000        0.000
## 75   Head   Pbl       0.000        0.000
## 76   Head   Dec       0.000        0.000
## 77   Head   Dis       0.000        0.000
## 78   Head   Cfl       0.000        0.000
## 79   Stom  Irri       0.000        0.000
## 80   Stom  Lone       0.000        0.000
## 81   Stom    WB       0.000        0.000
## 82   Stom   Pre       0.000        0.000
## 83   Stom   Tol       0.000        0.000
## 84   Stom   Wth       0.000        0.000
## 85   Stom   Prs       0.000        0.000
## 86   Stom   Esc       0.000        0.000
## 87   Stom   Pbl       0.000        0.000
## 88   Stom   Dec       0.000        0.000
## 89   Stom   Dis       0.000        0.000
## 90   Stom   Cfl       0.000        0.000
## 91   Back  Irri       0.000        0.000
## 92   Back  Nerv       0.000        0.000
## 93   Back  Lone       0.000        0.000
## 94   Back    WB       0.000        0.000
## 95   Back   Pre       0.000        0.000
## 96   Back   Tol       0.000        0.000
## 97   Back   Wth       0.000        0.000
## 98   Back   Prs       0.000        0.000
## 99   Back   Esc       0.000        0.000
## 100  Back   Pbl       0.000        0.000
## 101  Back   Dec       0.000        0.000
## 102  Back   Dis       0.000        0.000
## 103  Back   Cfl       0.000        0.000
## 104   Low   Pre       0.000        0.000
## 105   Low   Tol       0.000        0.000
## 106   Low   Wth       0.000        0.000
## 107   Low   Prs       0.000        0.000
## 108   Low   Pbl       0.000        0.000
## 109   Low   Dec       0.000        0.000
## 110   Low   Dis       0.000        0.000
## 111   Low   Cfl       0.000        0.000
## 112  Irri Sleep       0.000        0.000
## 113  Irri Dizzy       0.000        0.000
## 114  Irri  Lone       0.000        0.000
## 115  Irri    WB       0.000        0.000
## 116  Irri   Pre       0.000        0.000
## 117  Irri   Tol       0.000        0.000
## 118  Irri   Wth       0.000        0.000
## 119  Irri   Prs       0.000        0.000
## 120  Irri   Esc       0.000        0.000
## 121  Irri   Pbl       0.000        0.000
## 122  Irri   Dec       0.000        0.000
## 123  Irri   Dis       0.000        0.000
## 124  Irri   Cfl       0.000        0.000
## 125  Nerv Dizzy       0.000        0.000
## 126  Nerv  Lone       0.000        0.000
## 127  Nerv   Pre       0.000        0.000
## 128  Nerv   Tol       0.000        0.000
## 129  Nerv   Wth       0.000        0.000
## 130  Nerv   Prs       0.000        0.000
## 131  Nerv   Esc       0.000        0.000
## 132  Nerv   Pbl       0.000        0.000
## 133  Nerv   Dec       0.000        0.000
## 134  Nerv   Dis       0.000        0.000
## 135  Nerv   Cfl       0.000        0.000
## 136 Sleep   Pre       0.000        0.000
## 137 Sleep   Tol       0.000        0.000
## 138 Sleep   Wth       0.000        0.000
## 139 Sleep   Prs       0.000        0.000
## 140 Sleep   Pbl       0.000        0.000
## 141 Sleep   Dis       0.000        0.000
## 142 Sleep   Cfl       0.000        0.000
## 143 Dizzy  Lone       0.000        0.000
## 144 Dizzy    WB       0.000        0.000
## 145 Dizzy   Pre       0.000        0.000
## 146 Dizzy   Tol       0.000        0.000
## 147 Dizzy   Wth       0.000        0.000
## 148 Dizzy   Prs       0.000        0.000
## 149 Dizzy   Esc       0.000        0.000
## 150 Dizzy   Pbl       0.000        0.000
## 151 Dizzy   Dis       0.000        0.000
## 152 Dizzy   Cfl       0.000        0.000
## 153  Lone   Pre       0.000        0.000
## 154  Lone   Tol       0.000        0.000
## 155  Lone   Wth       0.000        0.000
## 156  Lone   Prs       0.000        0.000
## 157  Lone   Pbl       0.000        0.000
## 158  Lone   Dec       0.000        0.000
## 159  Lone   Dis       0.000        0.000
## 160  Lone   Cfl       0.000        0.000
## 161    WB   Pre       0.000        0.000
## 162    WB   Tol       0.000        0.000
## 163    WB   Wth       0.000        0.000
## 164    WB   Prs       0.000        0.000
## 165    WB   Esc       0.000        0.000
## 166    WB   Pbl       0.000        0.000
## 167    WB   Dec       0.000        0.000
## 168    WB   Dis       0.000        0.000
## 169    WB   Cfl       0.000        0.000
## 170   Pre   Dis       0.000        0.000
## 171   Esc   Dis       0.000        0.000
```

##### SMDS

```
##      From    To Weight_Boys Weight_Girls
## 1     Dec   Cfl       0.825        0.543
## 2     Pbl   Cfl       0.456        0.520
## 3    Irri  Nerv       0.516        0.493
## 4     Tol   Wth       0.511        0.483
## 5     Pre   Wth       0.250        0.446
## 6     Pre   Tol       0.379        0.403
## 7     Tol   Esc       0.379        0.219
## 8     Wth   Pbl       0.341        0.267
## 9    Lone    WB      -0.255       -0.325
## 10   Head Dizzy       0.311        0.307
## 11    Esc   Cfl       0.303        0.189
## 12    Pre   Esc       0.242        0.301
## 13    Wth   Dec       0.291        0.207
## 14    Prs   Cfl       0.102        0.279
## 15    Pbl   Dis       0.276        0.220
## 16    Esc   Pbl       0.269        0.157
## 17    Tol   Cfl       0.264        0.210
## 18    Dis   Cfl       0.249        0.155
## 19    Prs   Esc       0.242        0.236
## 20    Dec   Dis       0.119        0.233
## 21    Tol   Pbl       0.227        0.176
## 22    Pre   Cfl       0.223        0.087
## 23    Pre   Dis       0.189        0.221
## 24    Wth   Esc       0.218        0.146
## 25     WB   Esc       0.000       -0.213
## 26    Low    WB      -0.210       -0.212
## 27    Prs   Pbl       0.208        0.178
## 28   Head  Stom       0.206        0.182
## 29    Pre   Prs       0.200        0.150
## 30    Prs   Dec       0.200        0.070
## 31    Wth   Dis       0.163        0.187
## 32    Prs   Dis       0.074        0.178
## 33    Pre   Pbl       0.171        0.110
## 34    Wth   Prs       0.000        0.171
## 35    Pbl   Dec       0.082        0.168
## 36    Pre   Dec       0.167        0.107
## 37  Sleep Dizzy       0.117        0.165
## 38    Wth   Cfl       0.073        0.164
## 39   Lone   Esc       0.146        0.000
## 40   Stom Dizzy       0.137        0.105
## 41    Low  Irri       0.133        0.122
## 42    Esc   Dec       0.000        0.131
## 43    Low  Nerv       0.052        0.125
## 44   Head  Back       0.097        0.123
## 45   Stom  Back       0.000        0.115
## 46    Tol   Dec       0.111        0.087
## 47    Low  Lone       0.067        0.109
## 48    Tol   Dis       0.109        0.000
## 49    Low Sleep       0.000        0.103
## 50  Sleep  Lone       0.103        0.083
## 51  Sleep    WB      -0.101        0.000
## 52   Nerv    WB      -0.090       -0.098
## 53   Irri   Cfl       0.000        0.095
## 54    Low Dizzy       0.000        0.094
## 55   Head   Low       0.092        0.086
## 56   Stom   Cfl       0.087        0.000
## 57   Back   Low       0.086        0.000
## 58   Back Dizzy       0.000        0.076
## 59   Head    WB       0.000       -0.072
## 60   Stom   Low       0.070        0.057
## 61   Nerv Sleep       0.000        0.070
## 62   Stom  Nerv       0.000        0.065
## 63  Dizzy   Cfl       0.061        0.000
## 64    Tol   Prs       0.059        0.000
## 65   Stom Sleep       0.056        0.000
## 66   Back Sleep       0.052        0.055
## 67   Lone   Pre       0.053        0.000
## 68   Head Sleep       0.052        0.000
## 69   Head  Irri       0.000        0.000
## 70   Head  Nerv       0.000        0.000
## 71   Head  Lone       0.000        0.000
## 72   Head   Pre       0.000        0.000
## 73   Head   Tol       0.000        0.000
## 74   Head   Wth       0.000        0.000
## 75   Head   Prs       0.000        0.000
## 76   Head   Esc       0.000        0.000
## 77   Head   Pbl       0.000        0.000
## 78   Head   Dec       0.000        0.000
## 79   Head   Dis       0.000        0.000
## 80   Head   Cfl       0.000        0.000
## 81   Stom  Irri       0.000        0.000
## 82   Stom  Lone       0.000        0.000
## 83   Stom    WB       0.000        0.000
## 84   Stom   Pre       0.000        0.000
## 85   Stom   Tol       0.000        0.000
## 86   Stom   Wth       0.000        0.000
## 87   Stom   Prs       0.000        0.000
## 88   Stom   Esc       0.000        0.000
## 89   Stom   Pbl       0.000        0.000
## 90   Stom   Dec       0.000        0.000
## 91   Stom   Dis       0.000        0.000
## 92   Back  Irri       0.000        0.000
## 93   Back  Nerv       0.000        0.000
## 94   Back  Lone       0.000        0.000
## 95   Back    WB       0.000        0.000
## 96   Back   Pre       0.000        0.000
## 97   Back   Tol       0.000        0.000
## 98   Back   Wth       0.000        0.000
## 99   Back   Prs       0.000        0.000
## 100  Back   Esc       0.000        0.000
## 101  Back   Pbl       0.000        0.000
## 102  Back   Dec       0.000        0.000
## 103  Back   Dis       0.000        0.000
## 104  Back   Cfl       0.000        0.000
## 105   Low   Pre       0.000        0.000
## 106   Low   Tol       0.000        0.000
## 107   Low   Wth       0.000        0.000
## 108   Low   Prs       0.000        0.000
## 109   Low   Esc       0.000        0.000
## 110   Low   Pbl       0.000        0.000
## 111   Low   Dec       0.000        0.000
## 112   Low   Dis       0.000        0.000
## 113   Low   Cfl       0.000        0.000
## 114  Irri Sleep       0.000        0.000
## 115  Irri Dizzy       0.000        0.000
## 116  Irri  Lone       0.000        0.000
## 117  Irri    WB       0.000        0.000
## 118  Irri   Pre       0.000        0.000
## 119  Irri   Tol       0.000        0.000
## 120  Irri   Wth       0.000        0.000
## 121  Irri   Prs       0.000        0.000
## 122  Irri   Esc       0.000        0.000
## 123  Irri   Pbl       0.000        0.000
## 124  Irri   Dec       0.000        0.000
## 125  Irri   Dis       0.000        0.000
## 126  Nerv Dizzy       0.000        0.000
## 127  Nerv  Lone       0.000        0.000
## 128  Nerv   Pre       0.000        0.000
## 129  Nerv   Tol       0.000        0.000
## 130  Nerv   Wth       0.000        0.000
## 131  Nerv   Prs       0.000        0.000
## 132  Nerv   Esc       0.000        0.000
## 133  Nerv   Pbl       0.000        0.000
## 134  Nerv   Dec       0.000        0.000
## 135  Nerv   Dis       0.000        0.000
## 136  Nerv   Cfl       0.000        0.000
## 137 Sleep   Pre       0.000        0.000
## 138 Sleep   Tol       0.000        0.000
## 139 Sleep   Wth       0.000        0.000
## 140 Sleep   Prs       0.000        0.000
## 141 Sleep   Esc       0.000        0.000
## 142 Sleep   Pbl       0.000        0.000
## 143 Sleep   Dec       0.000        0.000
## 144 Sleep   Dis       0.000        0.000
## 145 Sleep   Cfl       0.000        0.000
## 146 Dizzy  Lone       0.000        0.000
## 147 Dizzy    WB       0.000        0.000
## 148 Dizzy   Pre       0.000        0.000
## 149 Dizzy   Tol       0.000        0.000
## 150 Dizzy   Wth       0.000        0.000
## 151 Dizzy   Prs       0.000        0.000
## 152 Dizzy   Esc       0.000        0.000
## 153 Dizzy   Pbl       0.000        0.000
## 154 Dizzy   Dec       0.000        0.000
## 155 Dizzy   Dis       0.000        0.000
## 156  Lone   Tol       0.000        0.000
## 157  Lone   Wth       0.000        0.000
## 158  Lone   Prs       0.000        0.000
## 159  Lone   Pbl       0.000        0.000
## 160  Lone   Dec       0.000        0.000
## 161  Lone   Dis       0.000        0.000
## 162  Lone   Cfl       0.000        0.000
## 163    WB   Pre       0.000        0.000
## 164    WB   Tol       0.000        0.000
## 165    WB   Wth       0.000        0.000
## 166    WB   Prs       0.000        0.000
## 167    WB   Pbl       0.000        0.000
## 168    WB   Dec       0.000        0.000
## 169    WB   Dis       0.000        0.000
## 170    WB   Cfl       0.000        0.000
## 171   Esc   Dis       0.000        0.000
```

#### Centrality

##### IGDS

**The centrality estimates are relatively stable when the
correlation stability coefficient (CS coefficient) is ≥ 0.5**
while set at correlation stability = 0.7 (Epskamp et al.,
2018). Centrality indices with CS coefficient of 0.25-0.5 should be
interpreted with caution; CS coefficient < 0.25 indicated large
instability of the centrality index.

```
##   region scale group        centrality    CS_r07
## 1     CY  IGDS  Boys          strength 0.7502851
## 2     CY  IGDS  Boys         closeness 0.2833523
## 3     CY  IGDS  Boys       betweenness 0.0000000
## 4     CY  IGDS  Boys expectedInfluence 0.7502851
## 5     CY  IGDS Girls          strength 0.7500000
## 6     CY  IGDS Girls         closeness 0.0000000
## 7     CY  IGDS Girls       betweenness 0.0000000
## 8     CY  IGDS Girls expectedInfluence 0.7500000
```

##### SMDS

**The centrality estimates are relatively stable when the
correlation stability coefficient (CS coefficient) is ≥ 0.5**
while set at correlation stability = 0.7 (Epskamp et al.,
2018). Centrality indices with CS coefficient of 0.25-0.5 should be
interpreted with caution; CS coefficient < 0.25 indicated large
instability of the centrality index.

```
##    region scale group        centrality    CS_r07
## 9      CY  SMDS  Boys          strength 0.6721779
## 10     CY  SMDS  Boys         closeness 0.2833523
## 11     CY  SMDS  Boys       betweenness 0.2058153
## 12     CY  SMDS  Boys expectedInfluence 0.7502851
## 13     CY  SMDS Girls          strength 0.6722561
## 14     CY  SMDS Girls         closeness 0.5167683
## 15     CY  SMDS Girls       betweenness 0.2835366
## 16     CY  SMDS Girls expectedInfluence 0.7500000
```

#### Bootstrapped Edges

##### IGDS

##### SMDS

### CZ

#### Figure

#### Edges

##### IGDS

```
##      From    To Weight_Boys Weight_Girls
## 1     Pbl   Cfl       0.526        0.961
## 2     Pre   Tol       0.507        0.807
## 3     Tol   Wth       0.662        0.645
## 4     Pre   Wth       0.524        0.597
## 5     Pbl   Dis       0.505        0.337
## 6     Pre   Esc       0.490        0.502
## 7     Wth   Dis       0.359        0.500
## 8     Dec   Cfl       0.452        0.495
## 9     Prs   Dec       0.327        0.477
## 10    Dis   Cfl       0.347        0.455
## 11    Tol   Dis       0.282        0.430
## 12    Wth   Cfl       0.392        0.000
## 13    Wth   Dec       0.360        0.336
## 14    Prs   Dis       0.360        0.261
## 15    Pbl   Dec       0.239        0.352
## 16    Tol   Pbl       0.322        0.123
## 17    Wth   Pbl       0.082        0.320
## 18    Esc   Dec       0.246        0.320
## 19    Prs   Esc       0.116        0.315
## 20    Dec   Dis       0.303        0.189
## 21    Prs   Pbl       0.185        0.294
## 22    Low  Irri       0.289        0.259
## 23   Lone    WB      -0.223       -0.268
## 24    Esc   Cfl       0.263        0.184
## 25    Prs   Cfl       0.240        0.262
## 26   Irri  Nerv       0.249        0.219
## 27    Pre   Dis       0.000        0.244
## 28    Tol   Prs       0.238        0.217
## 29    Low  Lone       0.235        0.235
## 30    Pre   Dec       0.152        0.226
## 31    Pre   Pbl       0.217        0.123
## 32    Tol   Esc       0.043        0.214
## 33    Tol   Dec       0.210        0.057
## 34    Pre   Cfl       0.000        0.208
## 35   Irri    WB      -0.207       -0.178
## 36    Wth   Esc       0.206        0.180
## 37    Esc   Pbl       0.120        0.206
## 38    Pre   Prs       0.053        0.204
## 39   Stom Dizzy       0.182        0.175
## 40   Head  Stom       0.178        0.155
## 41    Wth   Prs       0.097        0.168
## 42   Head Dizzy       0.123        0.157
## 43   Head  Back       0.133        0.148
## 44    Low  Nerv       0.079        0.147
## 45    Tol   Cfl       0.135        0.000
## 46  Sleep Dizzy       0.061        0.129
## 47    Esc   Dis       0.000        0.125
## 48    Low Dizzy       0.089        0.119
## 49   Irri Sleep       0.069        0.114
## 50   Irri  Lone       0.113        0.106
## 51     WB   Dis       0.000       -0.112
## 52   Head  Nerv       0.109        0.072
## 53   Head Sleep       0.105        0.090
## 54   Lone   Esc       0.072        0.104
## 55    Low    WB      -0.063       -0.101
## 56   Nerv Sleep       0.066        0.093
## 57   Head   Low       0.058        0.092
## 58    Low   Prs      -0.090        0.000
## 59   Back Dizzy       0.089        0.075
## 60  Sleep   Dec       0.000        0.088
## 61   Back   Low       0.060        0.087
## 62  Sleep    WB      -0.084       -0.059
## 63   Stom Sleep       0.083        0.037
## 64   Head  Irri       0.000        0.077
## 65  Dizzy   Cfl       0.077        0.000
## 66   Stom  Nerv       0.048        0.076
## 67   Back  Irri       0.075        0.000
## 68   Back  Nerv       0.027        0.074
## 69   Head   Cfl       0.000        0.071
## 70   Nerv   Prs       0.071        0.000
## 71   Nerv  Lone       0.056        0.069
## 72   Nerv   Esc       0.067        0.045
## 73  Sleep  Lone       0.061        0.067
## 74  Sleep   Esc       0.044        0.066
## 75   Nerv   Cfl       0.063        0.000
## 76   Back Sleep       0.061        0.047
## 77    Low   Cfl       0.058        0.000
## 78  Sleep   Prs       0.048        0.053
## 79     WB   Esc      -0.050       -0.053
## 80    Low Sleep       0.052        0.000
## 81   Nerv Dizzy       0.052        0.049
## 82   Nerv    WB      -0.040       -0.052
## 83   Stom  Irri       0.048        0.000
## 84   Irri   Pre       0.000        0.048
## 85   Stom  Back       0.038        0.038
## 86   Stom   Low       0.038        0.000
## 87    Low   Esc       0.038        0.000
## 88  Sleep   Pbl       0.000        0.038
## 89     WB   Tol      -0.037        0.000
## 90   Head  Lone       0.000        0.000
## 91   Head    WB       0.000        0.000
## 92   Head   Pre       0.000        0.000
## 93   Head   Tol       0.000        0.000
## 94   Head   Wth       0.000        0.000
## 95   Head   Prs       0.000        0.000
## 96   Head   Esc       0.000        0.000
## 97   Head   Pbl       0.000        0.000
## 98   Head   Dec       0.000        0.000
## 99   Head   Dis       0.000        0.000
## 100  Stom  Lone       0.000        0.000
## 101  Stom    WB       0.000        0.000
## 102  Stom   Pre       0.000        0.000
## 103  Stom   Tol       0.000        0.000
## 104  Stom   Wth       0.000        0.000
## 105  Stom   Prs       0.000        0.000
## 106  Stom   Esc       0.000        0.000
## 107  Stom   Pbl       0.000        0.000
## 108  Stom   Dec       0.000        0.000
## 109  Stom   Dis       0.000        0.000
## 110  Stom   Cfl       0.000        0.000
## 111  Back  Lone       0.000        0.000
## 112  Back    WB       0.000        0.000
## 113  Back   Pre       0.000        0.000
## 114  Back   Tol       0.000        0.000
## 115  Back   Wth       0.000        0.000
## 116  Back   Prs       0.000        0.000
## 117  Back   Esc       0.000        0.000
## 118  Back   Pbl       0.000        0.000
## 119  Back   Dec       0.000        0.000
## 120  Back   Dis       0.000        0.000
## 121  Back   Cfl       0.000        0.000
## 122   Low   Pre       0.000        0.000
## 123   Low   Tol       0.000        0.000
## 124   Low   Wth       0.000        0.000
## 125   Low   Pbl       0.000        0.000
## 126   Low   Dec       0.000        0.000
## 127   Low   Dis       0.000        0.000
## 128  Irri Dizzy       0.000        0.000
## 129  Irri   Tol       0.000        0.000
## 130  Irri   Wth       0.000        0.000
## 131  Irri   Prs       0.000        0.000
## 132  Irri   Esc       0.000        0.000
## 133  Irri   Pbl       0.000        0.000
## 134  Irri   Dec       0.000        0.000
## 135  Irri   Dis       0.000        0.000
## 136  Irri   Cfl       0.000        0.000
## 137  Nerv   Pre       0.000        0.000
## 138  Nerv   Tol       0.000        0.000
## 139  Nerv   Wth       0.000        0.000
## 140  Nerv   Pbl       0.000        0.000
## 141  Nerv   Dec       0.000        0.000
## 142  Nerv   Dis       0.000        0.000
## 143 Sleep   Pre       0.000        0.000
## 144 Sleep   Tol       0.000        0.000
## 145 Sleep   Wth       0.000        0.000
## 146 Sleep   Dis       0.000        0.000
## 147 Sleep   Cfl       0.000        0.000
## 148 Dizzy  Lone       0.000        0.000
## 149 Dizzy    WB       0.000        0.000
## 150 Dizzy   Pre       0.000        0.000
## 151 Dizzy   Tol       0.000        0.000
## 152 Dizzy   Wth       0.000        0.000
## 153 Dizzy   Prs       0.000        0.000
## 154 Dizzy   Esc       0.000        0.000
## 155 Dizzy   Pbl       0.000        0.000
## 156 Dizzy   Dec       0.000        0.000
## 157 Dizzy   Dis       0.000        0.000
## 158  Lone   Pre       0.000        0.000
## 159  Lone   Tol       0.000        0.000
## 160  Lone   Wth       0.000        0.000
## 161  Lone   Prs       0.000        0.000
## 162  Lone   Pbl       0.000        0.000
## 163  Lone   Dec       0.000        0.000
## 164  Lone   Dis       0.000        0.000
## 165  Lone   Cfl       0.000        0.000
## 166    WB   Pre       0.000        0.000
## 167    WB   Wth       0.000        0.000
## 168    WB   Prs       0.000        0.000
## 169    WB   Pbl       0.000        0.000
## 170    WB   Dec       0.000        0.000
## 171    WB   Cfl       0.000        0.000
```

##### SMDS

```
##      From    To Weight_Boys Weight_Girls
## 1     Dec   Cfl       0.664        0.465
## 2     Pre   Tol       0.416        0.658
## 3     Pre   Wth       0.652        0.535
## 4     Pbl   Cfl       0.549        0.641
## 5     Tol   Wth       0.579        0.635
## 6     Dec   Dis       0.409        0.226
## 7     Dis   Cfl       0.407        0.079
## 8     Tol   Pbl       0.371        0.291
## 9     Esc   Pbl       0.349        0.227
## 10    Tol   Prs       0.347        0.165
## 11    Wth   Dec       0.339        0.290
## 12    Pre   Dis       0.320        0.118
## 13    Prs   Esc       0.307        0.319
## 14    Esc   Dec       0.000        0.314
## 15    Low  Irri       0.287        0.256
## 16    Prs   Dis       0.238        0.278
## 17    Pre   Dec       0.271        0.239
## 18    Wth   Dis       0.268        0.159
## 19    Prs   Pbl       0.264        0.139
## 20   Lone    WB      -0.220       -0.259
## 21    Tol   Esc       0.249        0.149
## 22   Irri  Nerv       0.248        0.217
## 23    Wth   Cfl       0.231        0.213
## 24    Low  Lone       0.228        0.225
## 25    Pbl   Dec       0.223        0.217
## 26   Irri    WB      -0.208       -0.174
## 27    Pre   Esc       0.151        0.206
## 28    Pre   Prs       0.202        0.194
## 29    Esc   Cfl       0.202        0.131
## 30   Stom Dizzy       0.189        0.172
## 31    Wth   Pbl       0.147        0.186
## 32   Head  Stom       0.185        0.151
## 33    Esc   Dis       0.000        0.179
## 34    Wth   Esc       0.173        0.086
## 35   Lone   Esc       0.126        0.169
## 36    Pre   Pbl       0.155        0.168
## 37    Prs   Dec       0.164        0.157
## 38    Tol   Dis       0.119        0.163
## 39   Head Dizzy       0.126        0.153
## 40    Tol   Cfl       0.147        0.114
## 41   Head  Back       0.136        0.143
## 42    Low   Esc       0.087        0.143
## 43    Low  Nerv       0.074        0.139
## 44    Tol   Dec       0.139        0.091
## 45  Sleep Dizzy       0.062        0.127
## 46    Pbl   Dis       0.124        0.120
## 47   Nerv   Esc       0.095        0.121
## 48    Low Dizzy       0.088        0.119
## 49    Pre   Cfl       0.118        0.114
## 50   Irri Sleep       0.069        0.111
## 51   Irri  Lone       0.111        0.105
## 52   Head  Nerv       0.110        0.065
## 53   Head Sleep       0.108        0.088
## 54     WB   Esc       0.000       -0.097
## 55    Low    WB      -0.059       -0.093
## 56   Nerv Sleep       0.067        0.093
## 57   Back Dizzy       0.089        0.072
## 58   Head   Low       0.057        0.088
## 59   Back   Low       0.058        0.088
## 60   Stom Sleep       0.087        0.036
## 61  Sleep    WB      -0.083       -0.055
## 62   Irri   Pbl       0.000        0.078
## 63   Back  Irri       0.076        0.000
## 64    Prs   Cfl       0.076        0.076
## 65   Head  Irri       0.000        0.073
## 66  Sleep  Lone       0.060        0.073
## 67   Stom  Nerv       0.052        0.072
## 68   Stom   Cfl       0.000        0.071
## 69   Back  Nerv       0.026        0.067
## 70  Sleep   Dec       0.058        0.063
## 71     WB   Tol       0.000       -0.063
## 72   Back Sleep       0.061        0.048
## 73   Nerv  Lone       0.051        0.060
## 74   Stom   Prs       0.058        0.000
## 75   Nerv   Pre       0.058        0.000
## 76   Irri   Esc       0.052        0.000
## 77   Nerv Dizzy       0.052        0.043
## 78   Stom  Irri       0.051        0.000
## 79    Low Sleep       0.050        0.000
## 80    Low   Dis       0.000        0.050
## 81   Stom   Wth       0.000        0.048
## 82  Dizzy   Cfl       0.048        0.000
## 83   Irri   Wth       0.000        0.047
## 84   Head   Esc       0.000        0.045
## 85   Stom  Back       0.043        0.036
## 86   Back   Pre       0.000        0.043
## 87   Nerv   Tol       0.043        0.000
## 88   Nerv   Cfl       0.043        0.000
## 89    Low   Prs      -0.042        0.000
## 90   Stom   Low       0.041        0.000
## 91   Nerv    WB      -0.040       -0.039
## 92   Stom   Dis       0.030        0.000
## 93   Head  Lone       0.000        0.000
## 94   Head    WB       0.000        0.000
## 95   Head   Pre       0.000        0.000
## 96   Head   Tol       0.000        0.000
## 97   Head   Wth       0.000        0.000
## 98   Head   Prs       0.000        0.000
## 99   Head   Pbl       0.000        0.000
## 100  Head   Dec       0.000        0.000
## 101  Head   Dis       0.000        0.000
## 102  Head   Cfl       0.000        0.000
## 103  Stom  Lone       0.000        0.000
## 104  Stom    WB       0.000        0.000
## 105  Stom   Pre       0.000        0.000
## 106  Stom   Tol       0.000        0.000
## 107  Stom   Esc       0.000        0.000
## 108  Stom   Pbl       0.000        0.000
## 109  Stom   Dec       0.000        0.000
## 110  Back  Lone       0.000        0.000
## 111  Back    WB       0.000        0.000
## 112  Back   Tol       0.000        0.000
## 113  Back   Wth       0.000        0.000
## 114  Back   Prs       0.000        0.000
## 115  Back   Esc       0.000        0.000
## 116  Back   Pbl       0.000        0.000
## 117  Back   Dec       0.000        0.000
## 118  Back   Dis       0.000        0.000
## 119  Back   Cfl       0.000        0.000
## 120   Low   Pre       0.000        0.000
## 121   Low   Tol       0.000        0.000
## 122   Low   Wth       0.000        0.000
## 123   Low   Pbl       0.000        0.000
## 124   Low   Dec       0.000        0.000
## 125   Low   Cfl       0.000        0.000
## 126  Irri Dizzy       0.000        0.000
## 127  Irri   Pre       0.000        0.000
## 128  Irri   Tol       0.000        0.000
## 129  Irri   Prs       0.000        0.000
## 130  Irri   Dec       0.000        0.000
## 131  Irri   Dis       0.000        0.000
## 132  Irri   Cfl       0.000        0.000
## 133  Nerv   Wth       0.000        0.000
## 134  Nerv   Prs       0.000        0.000
## 135  Nerv   Pbl       0.000        0.000
## 136  Nerv   Dec       0.000        0.000
## 137  Nerv   Dis       0.000        0.000
## 138 Sleep   Pre       0.000        0.000
## 139 Sleep   Tol       0.000        0.000
## 140 Sleep   Wth       0.000        0.000
## 141 Sleep   Prs       0.000        0.000
## 142 Sleep   Esc       0.000        0.000
## 143 Sleep   Pbl       0.000        0.000
## 144 Sleep   Dis       0.000        0.000
## 145 Sleep   Cfl       0.000        0.000
## 146 Dizzy  Lone       0.000        0.000
## 147 Dizzy    WB       0.000        0.000
## 148 Dizzy   Pre       0.000        0.000
## 149 Dizzy   Tol       0.000        0.000
## 150 Dizzy   Wth       0.000        0.000
## 151 Dizzy   Prs       0.000        0.000
## 152 Dizzy   Esc       0.000        0.000
## 153 Dizzy   Pbl       0.000        0.000
## 154 Dizzy   Dec       0.000        0.000
## 155 Dizzy   Dis       0.000        0.000
## 156  Lone   Pre       0.000        0.000
## 157  Lone   Tol       0.000        0.000
## 158  Lone   Wth       0.000        0.000
## 159  Lone   Prs       0.000        0.000
## 160  Lone   Pbl       0.000        0.000
## 161  Lone   Dec       0.000        0.000
## 162  Lone   Dis       0.000        0.000
## 163  Lone   Cfl       0.000        0.000
## 164    WB   Pre       0.000        0.000
## 165    WB   Wth       0.000        0.000
## 166    WB   Prs       0.000        0.000
## 167    WB   Pbl       0.000        0.000
## 168    WB   Dec       0.000        0.000
## 169    WB   Dis       0.000        0.000
## 170    WB   Cfl       0.000        0.000
## 171   Wth   Prs       0.000        0.000
```

#### Centrality

##### IGDS

**The centrality estimates are relatively stable when the
correlation stability coefficient (CS coefficient) is ≥ 0.5**
while set at correlation stability = 0.7 (Epskamp et al.,
2018). Centrality indices with CS coefficient of 0.25-0.5 should be
interpreted with caution; CS coefficient < 0.25 indicated large
instability of the centrality index.

```
##   region scale group        centrality    CS_r07
## 1     CZ  IGDS  Boys          strength 0.7498924
## 2     CZ  IGDS  Boys         closeness 0.2832544
## 3     CZ  IGDS  Boys       betweenness 0.0000000
## 4     CZ  IGDS  Boys expectedInfluence 0.7498924
## 5     CZ  IGDS Girls          strength 0.7498623
## 6     CZ  IGDS Girls         closeness 0.2831956
## 7     CZ  IGDS Girls       betweenness 0.0000000
## 8     CZ  IGDS Girls expectedInfluence 0.7498623
```

##### SMDS

**The centrality estimates are relatively stable when the
correlation stability coefficient (CS coefficient) is ≥ 0.5**
while set at correlation stability = 0.7 (Epskamp et al.,
2018). Centrality indices with CS coefficient of 0.25-0.5 should be
interpreted with caution; CS coefficient < 0.25 indicated large
instability of the centrality index.

```
##    region scale group        centrality     CS_r07
## 9      CZ  SMDS  Boys          strength 0.74989238
## 10     CZ  SMDS  Boys         closeness 0.59448988
## 11     CZ  SMDS  Boys       betweenness 0.04993543
## 12     CZ  SMDS  Boys expectedInfluence 0.74989238
## 13     CZ  SMDS Girls          strength 0.74986226
## 14     CZ  SMDS Girls         closeness 0.67217631
## 15     CZ  SMDS Girls       betweenness 0.43884298
## 16     CZ  SMDS Girls expectedInfluence 0.74986226
```

#### Bootstrapped Edges

##### IGDS

##### SMDS

### EE

#### Figure

#### Edges

##### IGDS

```
##      From    To Weight_Boys Weight_Girls
## 1     Tol   Wth       0.630        0.747
## 2     Pbl   Cfl       0.578        0.674
## 3     Pbl   Dis       0.255        0.625
## 4     Dec   Dis       0.569        0.260
## 5     Pre   Tol       0.411        0.508
## 6     Dis   Cfl       0.449        0.176
## 7     Prs   Pbl       0.437        0.341
## 8     Prs   Dis       0.380        0.256
## 9     Prs   Dec       0.272        0.368
## 10   Irri  Nerv       0.325        0.361
## 11    Low  Irri       0.317        0.357
## 12    Pre   Esc       0.354        0.267
## 13    Dec   Cfl       0.350        0.128
## 14    Tol   Prs       0.344        0.313
## 15    Pbl   Dec       0.230        0.342
## 16    Tol   Dis       0.177        0.339
## 17    Pre   Cfl       0.096        0.337
## 18    Pre   Dis       0.328        0.258
## 19    Pre   Wth       0.319        0.294
## 20   Head  Stom       0.305        0.246
## 21    Wth   Esc       0.101        0.305
## 22    Tol   Pbl       0.294        0.160
## 23    Wth   Dec       0.220        0.282
## 24   Lone    WB      -0.216       -0.277
## 25    Pre   Prs       0.153        0.277
## 26    Esc   Pbl       0.202        0.276
## 27    Prs   Esc       0.091        0.272
## 28    Low  Lone       0.271        0.258
## 29    Wth   Dis       0.265        0.048
## 30    Wth   Cfl       0.227        0.226
## 31   Head Dizzy       0.107        0.216
## 32    Pre   Dec       0.213        0.178
## 33  Sleep Dizzy       0.208        0.183
## 34    Esc   Dec       0.153        0.202
## 35    Prs   Cfl       0.196        0.081
## 36    Tol   Esc       0.193        0.079
## 37    Tol   Dec       0.111        0.184
## 38    Pre   Pbl       0.160        0.168
## 39   Back Dizzy       0.123        0.154
## 40  Sleep    WB      -0.129       -0.116
## 41   Irri    WB      -0.128       -0.126
## 42    Low  Nerv       0.118        0.126
## 43    Low    WB      -0.080       -0.125
## 44     WB   Wth       0.000       -0.121
## 45    Wth   Pbl       0.105        0.121
## 46   Head  Back       0.107        0.111
## 47    Wth   Prs       0.109        0.000
## 48   Stom  Back       0.106        0.057
## 49   Stom Dizzy       0.048        0.104
## 50   Lone   Esc       0.100        0.000
## 51    Tol   Cfl       0.000        0.097
## 52   Back  Nerv       0.093        0.000
## 53   Nerv    WB      -0.083       -0.086
## 54   Head  Nerv       0.052        0.082
## 55   Irri Sleep       0.000        0.082
## 56   Nerv Sleep       0.079        0.052
## 57     WB   Esc      -0.077        0.000
## 58  Sleep   Esc       0.075        0.000
## 59   Stom   Low       0.000        0.074
## 60   Irri  Lone       0.074        0.000
## 61   Nerv Dizzy       0.000        0.069
## 62   Stom  Irri       0.066        0.000
## 63   Irri Dizzy       0.064        0.000
## 64   Head  Irri       0.062        0.000
## 65   Back Sleep       0.060        0.000
## 66   Irri   Pbl       0.057        0.000
## 67   Head    WB       0.000       -0.056
## 68   Nerv   Esc       0.053        0.000
## 69   Irri   Tol       0.049        0.000
## 70   Stom Sleep       0.045        0.000
## 71  Sleep  Lone       0.043        0.000
## 72   Head   Low       0.000        0.000
## 73   Head Sleep       0.000        0.000
## 74   Head  Lone       0.000        0.000
## 75   Head   Pre       0.000        0.000
## 76   Head   Tol       0.000        0.000
## 77   Head   Wth       0.000        0.000
## 78   Head   Prs       0.000        0.000
## 79   Head   Esc       0.000        0.000
## 80   Head   Pbl       0.000        0.000
## 81   Head   Dec       0.000        0.000
## 82   Head   Dis       0.000        0.000
## 83   Head   Cfl       0.000        0.000
## 84   Stom  Nerv       0.000        0.000
## 85   Stom  Lone       0.000        0.000
## 86   Stom    WB       0.000        0.000
## 87   Stom   Pre       0.000        0.000
## 88   Stom   Tol       0.000        0.000
## 89   Stom   Wth       0.000        0.000
## 90   Stom   Prs       0.000        0.000
## 91   Stom   Esc       0.000        0.000
## 92   Stom   Pbl       0.000        0.000
## 93   Stom   Dec       0.000        0.000
## 94   Stom   Dis       0.000        0.000
## 95   Stom   Cfl       0.000        0.000
## 96   Back   Low       0.000        0.000
## 97   Back  Irri       0.000        0.000
## 98   Back  Lone       0.000        0.000
## 99   Back    WB       0.000        0.000
## 100  Back   Pre       0.000        0.000
## 101  Back   Tol       0.000        0.000
## 102  Back   Wth       0.000        0.000
## 103  Back   Prs       0.000        0.000
## 104  Back   Esc       0.000        0.000
## 105  Back   Pbl       0.000        0.000
## 106  Back   Dec       0.000        0.000
## 107  Back   Dis       0.000        0.000
## 108  Back   Cfl       0.000        0.000
## 109   Low Sleep       0.000        0.000
## 110   Low Dizzy       0.000        0.000
## 111   Low   Pre       0.000        0.000
## 112   Low   Tol       0.000        0.000
## 113   Low   Wth       0.000        0.000
## 114   Low   Prs       0.000        0.000
## 115   Low   Esc       0.000        0.000
## 116   Low   Pbl       0.000        0.000
## 117   Low   Dec       0.000        0.000
## 118   Low   Dis       0.000        0.000
## 119   Low   Cfl       0.000        0.000
## 120  Irri   Pre       0.000        0.000
## 121  Irri   Wth       0.000        0.000
## 122  Irri   Prs       0.000        0.000
## 123  Irri   Esc       0.000        0.000
## 124  Irri   Dec       0.000        0.000
## 125  Irri   Dis       0.000        0.000
## 126  Irri   Cfl       0.000        0.000
## 127  Nerv  Lone       0.000        0.000
## 128  Nerv   Pre       0.000        0.000
## 129  Nerv   Tol       0.000        0.000
## 130  Nerv   Wth       0.000        0.000
## 131  Nerv   Prs       0.000        0.000
## 132  Nerv   Pbl       0.000        0.000
## 133  Nerv   Dec       0.000        0.000
## 134  Nerv   Dis       0.000        0.000
## 135  Nerv   Cfl       0.000        0.000
## 136 Sleep   Pre       0.000        0.000
## 137 Sleep   Tol       0.000        0.000
## 138 Sleep   Wth       0.000        0.000
## 139 Sleep   Prs       0.000        0.000
## 140 Sleep   Pbl       0.000        0.000
## 141 Sleep   Dec       0.000        0.000
## 142 Sleep   Dis       0.000        0.000
## 143 Sleep   Cfl       0.000        0.000
## 144 Dizzy  Lone       0.000        0.000
## 145 Dizzy    WB       0.000        0.000
## 146 Dizzy   Pre       0.000        0.000
## 147 Dizzy   Tol       0.000        0.000
## 148 Dizzy   Wth       0.000        0.000
## 149 Dizzy   Prs       0.000        0.000
## 150 Dizzy   Esc       0.000        0.000
## 151 Dizzy   Pbl       0.000        0.000
## 152 Dizzy   Dec       0.000        0.000
## 153 Dizzy   Dis       0.000        0.000
## 154 Dizzy   Cfl       0.000        0.000
## 155  Lone   Pre       0.000        0.000
## 156  Lone   Tol       0.000        0.000
## 157  Lone   Wth       0.000        0.000
## 158  Lone   Prs       0.000        0.000
## 159  Lone   Pbl       0.000        0.000
## 160  Lone   Dec       0.000        0.000
## 161  Lone   Dis       0.000        0.000
## 162  Lone   Cfl       0.000        0.000
## 163    WB   Pre       0.000        0.000
## 164    WB   Tol       0.000        0.000
## 165    WB   Prs       0.000        0.000
## 166    WB   Pbl       0.000        0.000
## 167    WB   Dec       0.000        0.000
## 168    WB   Dis       0.000        0.000
## 169    WB   Cfl       0.000        0.000
## 170   Esc   Dis       0.000        0.000
## 171   Esc   Cfl       0.000        0.000
```

##### SMDS

```
##      From    To Weight_Boys Weight_Girls
## 1     Pre   Tol       0.730        0.720
## 2     Pbl   Cfl       0.729        0.540
## 3     Tol   Wth       0.485        0.581
## 4     Dec   Cfl       0.543        0.546
## 5     Wth   Dec       0.500        0.223
## 6     Pbl   Dec       0.473        0.417
## 7     Dis   Cfl       0.361        0.423
## 8     Pbl   Dis       0.392        0.000
## 9     Pre   Wth       0.306        0.388
## 10    Dec   Dis       0.385        0.000
## 11    Tol   Pbl       0.358        0.216
## 12   Irri  Nerv       0.326        0.357
## 13    Low  Irri       0.312        0.354
## 14    Tol   Esc       0.350        0.170
## 15    Wth   Cfl       0.350        0.129
## 16    Pre   Cfl       0.330        0.098
## 17    Tol   Cfl       0.000        0.313
## 18    Prs   Esc       0.245        0.307
## 19   Head  Stom       0.304        0.236
## 20    Prs   Dis       0.170        0.289
## 21    Prs   Dec       0.288        0.229
## 22    Low  Lone       0.275        0.257
## 23    Esc   Dis       0.149        0.271
## 24   Lone    WB      -0.217       -0.267
## 25    Pre   Dec       0.224        0.243
## 26    Esc   Cfl       0.227        0.111
## 27   Head Dizzy       0.103        0.209
## 28  Sleep Dizzy       0.207        0.180
## 29    Tol   Dec       0.201        0.000
## 30    Esc   Pbl       0.193        0.000
## 31    Wth   Esc       0.183        0.124
## 32    Tol   Prs       0.175        0.182
## 33    Wth   Dis       0.171        0.000
## 34    Pre   Prs       0.165        0.153
## 35    Prs   Pbl       0.155        0.000
## 36   Back Dizzy       0.124        0.150
## 37    Pre   Esc       0.000        0.149
## 38    Pre   Pbl       0.000        0.148
## 39    Wth   Pbl       0.143        0.000
## 40    Pre   Dis       0.099        0.138
## 41  Sleep    WB      -0.134       -0.106
## 42   Irri    WB      -0.124       -0.128
## 43    Prs   Cfl       0.125        0.000
## 44    Low  Nerv       0.124        0.122
## 45   Lone   Esc       0.123        0.074
## 46    Low    WB      -0.086       -0.122
## 47    Low   Esc       0.111        0.119
## 48  Dizzy   Esc       0.000        0.114
## 49   Head  Back       0.112        0.100
## 50   Stom  Back       0.112        0.000
## 51   Stom Dizzy       0.043        0.102
## 52   Back  Nerv       0.098        0.000
## 53   Nerv    WB      -0.087       -0.079
## 54   Nerv   Esc       0.000        0.087
## 55   Nerv Sleep       0.084        0.000
## 56    Esc   Dec       0.000        0.083
## 57   Lone   Wth       0.000        0.081
## 58   Stom   Low       0.000        0.077
## 59   Head  Nerv       0.052        0.073
## 60    Tol   Dis       0.069        0.000
## 61   Back Sleep       0.068        0.000
## 62   Irri  Lone       0.067        0.000
## 63    Low Dizzy       0.063        0.000
## 64   Irri Dizzy       0.063        0.000
## 65   Nerv Dizzy       0.000        0.062
## 66   Stom  Irri       0.061        0.000
## 67   Head  Irri       0.057        0.000
## 68   Irri   Esc       0.056        0.000
## 69   Stom   Wth       0.054        0.000
## 70     WB   Esc      -0.054        0.000
## 71  Sleep  Lone       0.049        0.000
## 72   Stom Sleep       0.046        0.000
## 73   Head   Low       0.043        0.000
## 74   Head Sleep       0.000        0.000
## 75   Head  Lone       0.000        0.000
## 76   Head    WB       0.000        0.000
## 77   Head   Pre       0.000        0.000
## 78   Head   Tol       0.000        0.000
## 79   Head   Wth       0.000        0.000
## 80   Head   Prs       0.000        0.000
## 81   Head   Esc       0.000        0.000
## 82   Head   Pbl       0.000        0.000
## 83   Head   Dec       0.000        0.000
## 84   Head   Dis       0.000        0.000
## 85   Head   Cfl       0.000        0.000
## 86   Stom  Nerv       0.000        0.000
## 87   Stom  Lone       0.000        0.000
## 88   Stom    WB       0.000        0.000
## 89   Stom   Pre       0.000        0.000
## 90   Stom   Tol       0.000        0.000
## 91   Stom   Prs       0.000        0.000
## 92   Stom   Esc       0.000        0.000
## 93   Stom   Pbl       0.000        0.000
## 94   Stom   Dec       0.000        0.000
## 95   Stom   Dis       0.000        0.000
## 96   Stom   Cfl       0.000        0.000
## 97   Back   Low       0.000        0.000
## 98   Back  Irri       0.000        0.000
## 99   Back  Lone       0.000        0.000
## 100  Back    WB       0.000        0.000
## 101  Back   Pre       0.000        0.000
## 102  Back   Tol       0.000        0.000
## 103  Back   Wth       0.000        0.000
## 104  Back   Prs       0.000        0.000
## 105  Back   Esc       0.000        0.000
## 106  Back   Pbl       0.000        0.000
## 107  Back   Dec       0.000        0.000
## 108  Back   Dis       0.000        0.000
## 109  Back   Cfl       0.000        0.000
## 110   Low Sleep       0.000        0.000
## 111   Low   Pre       0.000        0.000
## 112   Low   Tol       0.000        0.000
## 113   Low   Wth       0.000        0.000
## 114   Low   Prs       0.000        0.000
## 115   Low   Pbl       0.000        0.000
## 116   Low   Dec       0.000        0.000
## 117   Low   Dis       0.000        0.000
## 118   Low   Cfl       0.000        0.000
## 119  Irri Sleep       0.000        0.000
## 120  Irri   Pre       0.000        0.000
## 121  Irri   Tol       0.000        0.000
## 122  Irri   Wth       0.000        0.000
## 123  Irri   Prs       0.000        0.000
## 124  Irri   Pbl       0.000        0.000
## 125  Irri   Dec       0.000        0.000
## 126  Irri   Dis       0.000        0.000
## 127  Irri   Cfl       0.000        0.000
## 128  Nerv  Lone       0.000        0.000
## 129  Nerv   Pre       0.000        0.000
## 130  Nerv   Tol       0.000        0.000
## 131  Nerv   Wth       0.000        0.000
## 132  Nerv   Prs       0.000        0.000
## 133  Nerv   Pbl       0.000        0.000
## 134  Nerv   Dec       0.000        0.000
## 135  Nerv   Dis       0.000        0.000
## 136  Nerv   Cfl       0.000        0.000
## 137 Sleep   Pre       0.000        0.000
## 138 Sleep   Tol       0.000        0.000
## 139 Sleep   Wth       0.000        0.000
## 140 Sleep   Prs       0.000        0.000
## 141 Sleep   Esc       0.000        0.000
## 142 Sleep   Pbl       0.000        0.000
## 143 Sleep   Dec       0.000        0.000
## 144 Sleep   Dis       0.000        0.000
## 145 Sleep   Cfl       0.000        0.000
## 146 Dizzy  Lone       0.000        0.000
## 147 Dizzy    WB       0.000        0.000
## 148 Dizzy   Pre       0.000        0.000
## 149 Dizzy   Tol       0.000        0.000
## 150 Dizzy   Wth       0.000        0.000
## 151 Dizzy   Prs       0.000        0.000
## 152 Dizzy   Pbl       0.000        0.000
## 153 Dizzy   Dec       0.000        0.000
## 154 Dizzy   Dis       0.000        0.000
## 155 Dizzy   Cfl       0.000        0.000
## 156  Lone   Pre       0.000        0.000
## 157  Lone   Tol       0.000        0.000
## 158  Lone   Prs       0.000        0.000
## 159  Lone   Pbl       0.000        0.000
## 160  Lone   Dec       0.000        0.000
## 161  Lone   Dis       0.000        0.000
## 162  Lone   Cfl       0.000        0.000
## 163    WB   Pre       0.000        0.000
## 164    WB   Tol       0.000        0.000
## 165    WB   Wth       0.000        0.000
## 166    WB   Prs       0.000        0.000
## 167    WB   Pbl       0.000        0.000
## 168    WB   Dec       0.000        0.000
## 169    WB   Dis       0.000        0.000
## 170    WB   Cfl       0.000        0.000
## 171   Wth   Prs       0.000        0.000
```

#### Centrality

##### IGDS

**The centrality estimates are relatively stable when the
correlation stability coefficient (CS coefficient) is ≥ 0.5**
while set at correlation stability = 0.7 (Epskamp et al.,
2018). Centrality indices with CS coefficient of 0.25-0.5 should be
interpreted with caution; CS coefficient < 0.25 indicated large
instability of the centrality index.

```
##   region scale group        centrality    CS_r07
## 1     EE  IGDS  Boys          strength 0.7497537
## 2     EE  IGDS  Boys         closeness 0.1275862
## 3     EE  IGDS  Boys       betweenness 0.0000000
## 4     EE  IGDS  Boys expectedInfluence 0.7497537
## 5     EE  IGDS Girls          strength 0.6724739
## 6     EE  IGDS Girls         closeness 0.1275261
## 7     EE  IGDS Girls       betweenness 0.1275261
## 8     EE  IGDS Girls expectedInfluence 0.6724739
```

##### SMDS

**The centrality estimates are relatively stable when the
correlation stability coefficient (CS coefficient) is ≥ 0.5**
while set at correlation stability = 0.7 (Epskamp et al.,
2018). Centrality indices with CS coefficient of 0.25-0.5 should be
interpreted with caution; CS coefficient < 0.25 indicated large
instability of the centrality index.

```
##    region scale group        centrality    CS_r07
## 9      EE  SMDS  Boys          strength 0.7497537
## 10     EE  SMDS  Boys         closeness 0.4389163
## 11     EE  SMDS  Boys       betweenness 0.0000000
## 12     EE  SMDS  Boys expectedInfluence 0.7497537
## 13     EE  SMDS Girls          strength 0.5944251
## 14     EE  SMDS Girls         closeness 0.1275261
## 15     EE  SMDS Girls       betweenness 0.1275261
## 16     EE  SMDS Girls expectedInfluence 0.7498258
```

#### Bootstrapped Edges

##### IGDS

###### Boys

###### Girls

##### SMDS

###### Boys

###### Girls

### GB-ENG

#### Figure

#### Edges

##### IGDS

```
##      From    To Weight_Boys Weight_Girls
## 1     Pbl   Cfl       0.678        1.056
## 2     Pre   Wth       0.563        0.345
## 3     Tol   Wth       0.501        0.560
## 4     Pre   Tol       0.396        0.541
## 5     Wth   Cfl       0.394        0.533
## 6     Pre   Esc       0.337        0.496
## 7     Dec   Dis       0.272        0.493
## 8     Wth   Dis       0.114        0.473
## 9     Pbl   Dec       0.459        0.404
## 10    Tol   Esc       0.339        0.411
## 11    Tol   Dis       0.378        0.353
## 12    Dec   Cfl       0.376        0.304
## 13    Pbl   Dis       0.307        0.347
## 14    Prs   Dec       0.182        0.335
## 15    Pre   Prs       0.140        0.326
## 16    Prs   Dis       0.248        0.313
## 17    Wth   Esc       0.238        0.309
## 18    Esc   Pbl       0.267        0.000
## 19    Prs   Cfl       0.181        0.266
## 20    Low  Irri       0.169        0.263
## 21    Pre   Dec       0.178        0.261
## 22    Dis   Cfl       0.261        0.106
## 23    Low  Lone       0.259        0.209
## 24   Lone    WB      -0.239       -0.244
## 25    Prs   Pbl       0.241        0.000
## 26    Low    WB      -0.227       -0.240
## 27   Head Dizzy       0.233        0.237
## 28    Low  Nerv       0.236        0.177
## 29   Head  Stom       0.221        0.224
## 30    Pre   Pbl       0.000        0.224
## 31    Wth   Dec       0.222        0.000
## 32    Tol   Prs       0.221        0.000
## 33    Wth   Pbl       0.208        0.000
## 34   Head  Back       0.000        0.198
## 35    Pre   Dis       0.000        0.195
## 36    Esc   Dec       0.190        0.124
## 37   Stom Dizzy       0.115        0.180
## 38    Prs   Esc       0.180        0.153
## 39    Tol   Dec       0.078        0.179
## 40  Sleep    WB      -0.151       -0.108
## 41   Nerv  Lone       0.098        0.133
## 42   Irri Sleep       0.131        0.000
## 43     WB   Dis       0.000       -0.129
## 44   Nerv Dizzy       0.124        0.071
## 45   Nerv Sleep       0.000        0.123
## 46   Stom  Back       0.119        0.117
## 47  Sleep Dizzy       0.114        0.112
## 48    Tol   Pbl       0.108        0.088
## 49   Irri  Nerv       0.054        0.107
## 50   Stom  Irri       0.101        0.000
## 51  Dizzy   Cfl       0.000        0.097
## 52   Stom  Nerv       0.096        0.050
## 53   Irri   Pbl       0.093        0.000
## 54    Esc   Dis       0.089        0.000
## 55   Head   Low       0.087        0.075
## 56    Low Dizzy       0.000        0.081
## 57    Wth   Prs       0.078        0.078
## 58   Head Sleep       0.073        0.077
## 59    Low Sleep       0.000        0.073
## 60   Stom   Low       0.000        0.061
## 61   Stom    WB       0.000       -0.061
## 62   Head  Irri       0.060        0.000
## 63   Nerv    WB       0.000       -0.058
## 64  Sleep   Dec       0.000        0.055
## 65   Back Sleep       0.053        0.000
## 66  Sleep   Esc       0.049        0.000
## 67   Irri    WB      -0.048        0.000
## 68   Head  Nerv       0.000        0.000
## 69   Head  Lone       0.000        0.000
## 70   Head    WB       0.000        0.000
## 71   Head   Pre       0.000        0.000
## 72   Head   Tol       0.000        0.000
## 73   Head   Wth       0.000        0.000
## 74   Head   Prs       0.000        0.000
## 75   Head   Esc       0.000        0.000
## 76   Head   Pbl       0.000        0.000
## 77   Head   Dec       0.000        0.000
## 78   Head   Dis       0.000        0.000
## 79   Head   Cfl       0.000        0.000
## 80   Stom Sleep       0.000        0.000
## 81   Stom  Lone       0.000        0.000
## 82   Stom   Pre       0.000        0.000
## 83   Stom   Tol       0.000        0.000
## 84   Stom   Wth       0.000        0.000
## 85   Stom   Prs       0.000        0.000
## 86   Stom   Esc       0.000        0.000
## 87   Stom   Pbl       0.000        0.000
## 88   Stom   Dec       0.000        0.000
## 89   Stom   Dis       0.000        0.000
## 90   Stom   Cfl       0.000        0.000
## 91   Back   Low       0.000        0.000
## 92   Back  Irri       0.000        0.000
## 93   Back  Nerv       0.000        0.000
## 94   Back Dizzy       0.000        0.000
## 95   Back  Lone       0.000        0.000
## 96   Back    WB       0.000        0.000
## 97   Back   Pre       0.000        0.000
## 98   Back   Tol       0.000        0.000
## 99   Back   Wth       0.000        0.000
## 100  Back   Prs       0.000        0.000
## 101  Back   Esc       0.000        0.000
## 102  Back   Pbl       0.000        0.000
## 103  Back   Dec       0.000        0.000
## 104  Back   Dis       0.000        0.000
## 105  Back   Cfl       0.000        0.000
## 106   Low   Pre       0.000        0.000
## 107   Low   Tol       0.000        0.000
## 108   Low   Wth       0.000        0.000
## 109   Low   Prs       0.000        0.000
## 110   Low   Esc       0.000        0.000
## 111   Low   Pbl       0.000        0.000
## 112   Low   Dec       0.000        0.000
## 113   Low   Dis       0.000        0.000
## 114   Low   Cfl       0.000        0.000
## 115  Irri Dizzy       0.000        0.000
## 116  Irri  Lone       0.000        0.000
## 117  Irri   Pre       0.000        0.000
## 118  Irri   Tol       0.000        0.000
## 119  Irri   Wth       0.000        0.000
## 120  Irri   Prs       0.000        0.000
## 121  Irri   Esc       0.000        0.000
## 122  Irri   Dec       0.000        0.000
## 123  Irri   Dis       0.000        0.000
## 124  Irri   Cfl       0.000        0.000
## 125  Nerv   Pre       0.000        0.000
## 126  Nerv   Tol       0.000        0.000
## 127  Nerv   Wth       0.000        0.000
## 128  Nerv   Prs       0.000        0.000
## 129  Nerv   Esc       0.000        0.000
## 130  Nerv   Pbl       0.000        0.000
## 131  Nerv   Dec       0.000        0.000
## 132  Nerv   Dis       0.000        0.000
## 133  Nerv   Cfl       0.000        0.000
## 134 Sleep  Lone       0.000        0.000
## 135 Sleep   Pre       0.000        0.000
## 136 Sleep   Tol       0.000        0.000
## 137 Sleep   Wth       0.000        0.000
## 138 Sleep   Prs       0.000        0.000
## 139 Sleep   Pbl       0.000        0.000
## 140 Sleep   Dis       0.000        0.000
## 141 Sleep   Cfl       0.000        0.000
## 142 Dizzy  Lone       0.000        0.000
## 143 Dizzy    WB       0.000        0.000
## 144 Dizzy   Pre       0.000        0.000
## 145 Dizzy   Tol       0.000        0.000
## 146 Dizzy   Wth       0.000        0.000
## 147 Dizzy   Prs       0.000        0.000
## 148 Dizzy   Esc       0.000        0.000
## 149 Dizzy   Pbl       0.000        0.000
## 150 Dizzy   Dec       0.000        0.000
## 151 Dizzy   Dis       0.000        0.000
## 152  Lone   Pre       0.000        0.000
## 153  Lone   Tol       0.000        0.000
## 154  Lone   Wth       0.000        0.000
## 155  Lone   Prs       0.000        0.000
## 156  Lone   Esc       0.000        0.000
## 157  Lone   Pbl       0.000        0.000
## 158  Lone   Dec       0.000        0.000
## 159  Lone   Dis       0.000        0.000
## 160  Lone   Cfl       0.000        0.000
## 161    WB   Pre       0.000        0.000
## 162    WB   Tol       0.000        0.000
## 163    WB   Wth       0.000        0.000
## 164    WB   Prs       0.000        0.000
## 165    WB   Esc       0.000        0.000
## 166    WB   Pbl       0.000        0.000
## 167    WB   Dec       0.000        0.000
## 168    WB   Cfl       0.000        0.000
## 169   Pre   Cfl       0.000        0.000
## 170   Tol   Cfl       0.000        0.000
## 171   Esc   Cfl       0.000        0.000
```

##### SMDS

```
##      From    To Weight_Boys Weight_Girls
## 1     Pre   Tol       0.715        0.815
## 2     Tol   Wth       0.577        0.765
## 3     Pbl   Cfl       0.735        0.751
## 4     Dec   Cfl       0.660        0.546
## 5     Pre   Wth       0.498        0.319
## 6     Prs   Esc       0.322        0.492
## 7     Prs   Dis       0.335        0.145
## 8     Pbl   Dec       0.190        0.331
## 9     Esc   Dec       0.303        0.109
## 10    Esc   Pbl       0.293        0.142
## 11    Wth   Cfl       0.284        0.164
## 12    Dis   Cfl       0.281        0.000
## 13    Tol   Pbl       0.268        0.274
## 14    Low  Irri       0.170        0.262
## 15    Low  Lone       0.256        0.207
## 16    Tol   Esc       0.241        0.121
## 17    Prs   Dec       0.236        0.239
## 18   Lone    WB      -0.238       -0.238
## 19    Low    WB      -0.228       -0.237
## 20    Esc   Dis       0.168        0.237
## 21    Low  Nerv       0.233        0.174
## 22    Pre   Esc       0.093        0.233
## 23   Head Dizzy       0.227        0.230
## 24   Head  Stom       0.203        0.222
## 25    Wth   Dis       0.167        0.216
## 26    Prs   Pbl       0.214        0.125
## 27    Tol   Dec       0.000        0.213
## 28    Pre   Dis       0.191        0.212
## 29    Tol   Prs       0.209        0.000
## 30   Head  Back       0.000        0.194
## 31    Pre   Cfl       0.194        0.134
## 32    Wth   Esc       0.179        0.098
## 33    Dec   Dis       0.095        0.174
## 34    Pbl   Dis       0.165        0.158
## 35   Stom Dizzy       0.101        0.163
## 36    Wth   Prs       0.000        0.162
## 37    Pre   Pbl       0.161        0.066
## 38    Wth   Pbl       0.154        0.095
## 39  Sleep    WB      -0.149       -0.106
## 40   Irri Sleep       0.143        0.000
## 41   Nerv  Lone       0.096        0.133
## 42     WB   Esc       0.000       -0.133
## 43   Nerv Sleep       0.000        0.124
## 44   Nerv Dizzy       0.123        0.064
## 45    Tol   Dis       0.100        0.119
## 46    Low   Esc       0.116        0.000
## 47  Sleep Dizzy       0.115        0.098
## 48   Lone   Esc       0.113        0.000
## 49   Stom  Back       0.101        0.107
## 50   Nerv   Esc       0.104        0.000
## 51   Stom  Irri       0.102        0.000
## 52   Irri  Nerv       0.062        0.099
## 53   Head   Low       0.092        0.074
## 54   Stom  Nerv       0.082        0.000
## 55    Low Dizzy       0.000        0.080
## 56   Irri   Pbl       0.077        0.000
## 57   Head Sleep       0.070        0.075
## 58   Stom   Tol       0.000        0.068
## 59    Pre   Dec       0.063        0.000
## 60   Head  Irri       0.062        0.000
## 61   Back Sleep       0.057        0.000
## 62    Pre   Prs       0.056        0.055
## 63   Irri    WB      -0.055        0.000
## 64   Back  Irri       0.049        0.000
## 65   Head  Nerv       0.000        0.000
## 66   Head  Lone       0.000        0.000
## 67   Head    WB       0.000        0.000
## 68   Head   Pre       0.000        0.000
## 69   Head   Tol       0.000        0.000
## 70   Head   Wth       0.000        0.000
## 71   Head   Prs       0.000        0.000
## 72   Head   Esc       0.000        0.000
## 73   Head   Pbl       0.000        0.000
## 74   Head   Dec       0.000        0.000
## 75   Head   Dis       0.000        0.000
## 76   Head   Cfl       0.000        0.000
## 77   Stom   Low       0.000        0.000
## 78   Stom Sleep       0.000        0.000
## 79   Stom  Lone       0.000        0.000
## 80   Stom    WB       0.000        0.000
## 81   Stom   Pre       0.000        0.000
## 82   Stom   Wth       0.000        0.000
## 83   Stom   Prs       0.000        0.000
## 84   Stom   Esc       0.000        0.000
## 85   Stom   Pbl       0.000        0.000
## 86   Stom   Dec       0.000        0.000
## 87   Stom   Dis       0.000        0.000
## 88   Stom   Cfl       0.000        0.000
## 89   Back   Low       0.000        0.000
## 90   Back  Nerv       0.000        0.000
## 91   Back Dizzy       0.000        0.000
## 92   Back  Lone       0.000        0.000
## 93   Back    WB       0.000        0.000
## 94   Back   Pre       0.000        0.000
## 95   Back   Tol       0.000        0.000
## 96   Back   Wth       0.000        0.000
## 97   Back   Prs       0.000        0.000
## 98   Back   Esc       0.000        0.000
## 99   Back   Pbl       0.000        0.000
## 100  Back   Dec       0.000        0.000
## 101  Back   Dis       0.000        0.000
## 102  Back   Cfl       0.000        0.000
## 103   Low Sleep       0.000        0.000
## 104   Low   Pre       0.000        0.000
## 105   Low   Tol       0.000        0.000
## 106   Low   Wth       0.000        0.000
## 107   Low   Prs       0.000        0.000
## 108   Low   Pbl       0.000        0.000
## 109   Low   Dec       0.000        0.000
## 110   Low   Dis       0.000        0.000
## 111   Low   Cfl       0.000        0.000
## 112  Irri Dizzy       0.000        0.000
## 113  Irri  Lone       0.000        0.000
## 114  Irri   Pre       0.000        0.000
## 115  Irri   Tol       0.000        0.000
## 116  Irri   Wth       0.000        0.000
## 117  Irri   Prs       0.000        0.000
## 118  Irri   Esc       0.000        0.000
## 119  Irri   Dec       0.000        0.000
## 120  Irri   Dis       0.000        0.000
## 121  Irri   Cfl       0.000        0.000
## 122  Nerv    WB       0.000        0.000
## 123  Nerv   Pre       0.000        0.000
## 124  Nerv   Tol       0.000        0.000
## 125  Nerv   Wth       0.000        0.000
## 126  Nerv   Prs       0.000        0.000
## 127  Nerv   Pbl       0.000        0.000
## 128  Nerv   Dec       0.000        0.000
## 129  Nerv   Dis       0.000        0.000
## 130  Nerv   Cfl       0.000        0.000
## 131 Sleep  Lone       0.000        0.000
## 132 Sleep   Pre       0.000        0.000
## 133 Sleep   Tol       0.000        0.000
## 134 Sleep   Wth       0.000        0.000
## 135 Sleep   Prs       0.000        0.000
## 136 Sleep   Esc       0.000        0.000
## 137 Sleep   Pbl       0.000        0.000
## 138 Sleep   Dec       0.000        0.000
## 139 Sleep   Dis       0.000        0.000
## 140 Sleep   Cfl       0.000        0.000
## 141 Dizzy  Lone       0.000        0.000
## 142 Dizzy    WB       0.000        0.000
## 143 Dizzy   Pre       0.000        0.000
## 144 Dizzy   Tol       0.000        0.000
## 145 Dizzy   Wth       0.000        0.000
## 146 Dizzy   Prs       0.000        0.000
## 147 Dizzy   Esc       0.000        0.000
## 148 Dizzy   Pbl       0.000        0.000
## 149 Dizzy   Dec       0.000        0.000
## 150 Dizzy   Dis       0.000        0.000
## 151 Dizzy   Cfl       0.000        0.000
## 152  Lone   Pre       0.000        0.000
## 153  Lone   Tol       0.000        0.000
## 154  Lone   Wth       0.000        0.000
## 155  Lone   Prs       0.000        0.000
## 156  Lone   Pbl       0.000        0.000
## 157  Lone   Dec       0.000        0.000
## 158  Lone   Dis       0.000        0.000
## 159  Lone   Cfl       0.000        0.000
## 160    WB   Pre       0.000        0.000
## 161    WB   Tol       0.000        0.000
## 162    WB   Wth       0.000        0.000
## 163    WB   Prs       0.000        0.000
## 164    WB   Pbl       0.000        0.000
## 165    WB   Dec       0.000        0.000
## 166    WB   Dis       0.000        0.000
## 167    WB   Cfl       0.000        0.000
## 168   Tol   Cfl       0.000        0.000
## 169   Wth   Dec       0.000        0.000
## 170   Prs   Cfl       0.000        0.000
## 171   Esc   Cfl       0.000        0.000
```

#### Centrality

##### IGDS

**The centrality estimates are relatively stable when the
correlation stability coefficient (CS coefficient) is ≥ 0.5**
while set at correlation stability = 0.7 (Epskamp et al.,
2018). Centrality indices with CS coefficient of 0.25-0.5 should be
interpreted with caution; CS coefficient < 0.25 indicated large
instability of the centrality index.

```
##   region scale group        centrality     CS_r07
## 1 GB-ENG  IGDS  Boys          strength 0.75016880
## 2 GB-ENG  IGDS  Boys         closeness 0.12761648
## 3 GB-ENG  IGDS  Boys       betweenness 0.04996624
## 4 GB-ENG  IGDS  Boys expectedInfluence 0.75016880
## 5 GB-ENG  IGDS Girls          strength 0.67250571
## 6 GB-ENG  IGDS Girls         closeness 0.05026657
## 7 GB-ENG  IGDS Girls       betweenness 0.00000000
## 8 GB-ENG  IGDS Girls expectedInfluence 0.75019040
```

##### SMDS

**The centrality estimates are relatively stable when the
correlation stability coefficient (CS coefficient) is ≥ 0.5**
while set at correlation stability = 0.7 (Epskamp et al.,
2018). Centrality indices with CS coefficient of 0.25-0.5 should be
interpreted with caution; CS coefficient < 0.25 indicated large
instability of the centrality index.

```
##    region scale group        centrality    CS_r07
## 9  GB-ENG  SMDS  Boys          strength 0.6725186
## 10 GB-ENG  SMDS  Boys         closeness 0.2052667
## 11 GB-ENG  SMDS  Boys       betweenness 0.2052667
## 12 GB-ENG  SMDS  Boys expectedInfluence 0.7501688
## 13 GB-ENG  SMDS Girls          strength 0.5948210
## 14 GB-ENG  SMDS Girls         closeness 0.2833206
## 15 GB-ENG  SMDS Girls       betweenness 0.2056359
## 16 GB-ENG  SMDS Girls expectedInfluence 0.7501904
```

#### Bootstrapped Edges

##### IGDS

###### Boys

###### Girls

##### SMDS

###### Boys

###### Girls

### IS

#### Figure

#### Edges

##### IGDS

```
##      From    To Weight_Boys Weight_Girls
## 1     Pre   Wth       0.314        0.950
## 2     Pbl   Dis       0.379        0.899
## 3     Wth   Cfl       0.576        0.853
## 4     Wth   Dec       0.177        0.713
## 5     Prs   Dis       0.242        0.681
## 6     Tol   Dis       0.103        0.649
## 7     Pbl   Cfl       0.240        0.648
## 8     Pre   Esc       0.371        0.623
## 9     Tol   Esc       0.000        0.562
## 10    Dec   Cfl       0.559        0.296
## 11    Pre   Dec       0.227        0.510
## 12    Tol   Wth       0.489        0.510
## 13    Dec   Dis       0.503        0.203
## 14    Tol   Pbl       0.468        0.000
## 15    Prs   Pbl       0.284        0.468
## 16    Wth   Esc       0.467        0.238
## 17    Pre   Tol       0.465        0.396
## 18    Esc   Cfl       0.000        0.454
## 19    Pbl   Dec       0.265        0.448
## 20    Wth   Dis       0.418        0.000
## 21    Tol   Cfl       0.413        0.000
## 22    Dis   Cfl       0.344        0.000
## 23    Pre   Pbl       0.317        0.000
## 24    Esc   Pbl       0.295        0.000
## 25    Pre   Dis       0.282        0.285
## 26    Low  Irri       0.194        0.279
## 27   Head Dizzy       0.219        0.263
## 28    Low  Nerv       0.255        0.221
## 29   Irri  Nerv       0.107        0.250
## 30    Low  Lone       0.248        0.222
## 31   Head  Stom       0.237        0.189
## 32    Prs   Esc       0.138        0.233
## 33    Prs   Dec       0.232        0.000
## 34    Tol   Dec       0.220        0.000
## 35    Esc   Dec       0.215        0.000
## 36   Lone    WB      -0.160       -0.202
## 37    Low    WB      -0.197       -0.177
## 38    Tol   Prs       0.185        0.000
## 39     WB   Esc      -0.158        0.000
## 40     WB   Dis       0.000       -0.156
## 41    Low Sleep       0.152        0.000
## 42  Sleep Dizzy       0.000        0.149
## 43    Wth   Prs       0.142        0.000
## 44   Nerv Dizzy       0.137        0.000
## 45   Stom  Back       0.000        0.134
## 46  Sleep    WB      -0.129       -0.106
## 47    Low Dizzy       0.000        0.127
## 48    Pre   Prs       0.106        0.000
## 49   Back Dizzy       0.074        0.105
## 50   Head  Back       0.101        0.095
## 51   Back Sleep       0.098        0.000
## 52   Stom  Nerv       0.088        0.094
## 53   Stom Dizzy       0.086        0.088
## 54     WB   Pre      -0.088        0.000
## 55   Lone   Esc       0.086        0.000
## 56   Stom   Low       0.083        0.000
## 57    Wth   Pbl       0.080        0.000
## 58   Back  Nerv       0.078        0.000
## 59   Irri   Tol       0.075        0.000
## 60   Head Sleep       0.066        0.000
## 61   Stom Sleep       0.066        0.000
## 62   Head   Low       0.000        0.000
## 63   Head  Irri       0.000        0.000
## 64   Head  Nerv       0.000        0.000
## 65   Head  Lone       0.000        0.000
## 66   Head    WB       0.000        0.000
## 67   Head   Pre       0.000        0.000
## 68   Head   Tol       0.000        0.000
## 69   Head   Wth       0.000        0.000
## 70   Head   Prs       0.000        0.000
## 71   Head   Esc       0.000        0.000
## 72   Head   Pbl       0.000        0.000
## 73   Head   Dec       0.000        0.000
## 74   Head   Dis       0.000        0.000
## 75   Head   Cfl       0.000        0.000
## 76   Stom  Irri       0.000        0.000
## 77   Stom  Lone       0.000        0.000
## 78   Stom    WB       0.000        0.000
## 79   Stom   Pre       0.000        0.000
## 80   Stom   Tol       0.000        0.000
## 81   Stom   Wth       0.000        0.000
## 82   Stom   Prs       0.000        0.000
## 83   Stom   Esc       0.000        0.000
## 84   Stom   Pbl       0.000        0.000
## 85   Stom   Dec       0.000        0.000
## 86   Stom   Dis       0.000        0.000
## 87   Stom   Cfl       0.000        0.000
## 88   Back   Low       0.000        0.000
## 89   Back  Irri       0.000        0.000
## 90   Back  Lone       0.000        0.000
## 91   Back    WB       0.000        0.000
## 92   Back   Pre       0.000        0.000
## 93   Back   Tol       0.000        0.000
## 94   Back   Wth       0.000        0.000
## 95   Back   Prs       0.000        0.000
## 96   Back   Esc       0.000        0.000
## 97   Back   Pbl       0.000        0.000
## 98   Back   Dec       0.000        0.000
## 99   Back   Dis       0.000        0.000
## 100  Back   Cfl       0.000        0.000
## 101   Low   Pre       0.000        0.000
## 102   Low   Tol       0.000        0.000
## 103   Low   Wth       0.000        0.000
## 104   Low   Prs       0.000        0.000
## 105   Low   Esc       0.000        0.000
## 106   Low   Pbl       0.000        0.000
## 107   Low   Dec       0.000        0.000
## 108   Low   Dis       0.000        0.000
## 109   Low   Cfl       0.000        0.000
## 110  Irri Sleep       0.000        0.000
## 111  Irri Dizzy       0.000        0.000
## 112  Irri  Lone       0.000        0.000
## 113  Irri    WB       0.000        0.000
## 114  Irri   Pre       0.000        0.000
## 115  Irri   Wth       0.000        0.000
## 116  Irri   Prs       0.000        0.000
## 117  Irri   Esc       0.000        0.000
## 118  Irri   Pbl       0.000        0.000
## 119  Irri   Dec       0.000        0.000
## 120  Irri   Dis       0.000        0.000
## 121  Irri   Cfl       0.000        0.000
## 122  Nerv Sleep       0.000        0.000
## 123  Nerv  Lone       0.000        0.000
## 124  Nerv    WB       0.000        0.000
## 125  Nerv   Pre       0.000        0.000
## 126  Nerv   Tol       0.000        0.000
## 127  Nerv   Wth       0.000        0.000
## 128  Nerv   Prs       0.000        0.000
## 129  Nerv   Esc       0.000        0.000
## 130  Nerv   Pbl       0.000        0.000
## 131  Nerv   Dec       0.000        0.000
## 132  Nerv   Dis       0.000        0.000
## 133  Nerv   Cfl       0.000        0.000
## 134 Sleep  Lone       0.000        0.000
## 135 Sleep   Pre       0.000        0.000
## 136 Sleep   Tol       0.000        0.000
## 137 Sleep   Wth       0.000        0.000
## 138 Sleep   Prs       0.000        0.000
## 139 Sleep   Esc       0.000        0.000
## 140 Sleep   Pbl       0.000        0.000
## 141 Sleep   Dec       0.000        0.000
## 142 Sleep   Dis       0.000        0.000
## 143 Sleep   Cfl       0.000        0.000
## 144 Dizzy  Lone       0.000        0.000
## 145 Dizzy    WB       0.000        0.000
## 146 Dizzy   Pre       0.000        0.000
## 147 Dizzy   Tol       0.000        0.000
## 148 Dizzy   Wth       0.000        0.000
## 149 Dizzy   Prs       0.000        0.000
## 150 Dizzy   Esc       0.000        0.000
## 151 Dizzy   Pbl       0.000        0.000
## 152 Dizzy   Dec       0.000        0.000
## 153 Dizzy   Dis       0.000        0.000
## 154 Dizzy   Cfl       0.000        0.000
## 155  Lone   Pre       0.000        0.000
## 156  Lone   Tol       0.000        0.000
## 157  Lone   Wth       0.000        0.000
## 158  Lone   Prs       0.000        0.000
## 159  Lone   Pbl       0.000        0.000
## 160  Lone   Dec       0.000        0.000
## 161  Lone   Dis       0.000        0.000
## 162  Lone   Cfl       0.000        0.000
## 163    WB   Tol       0.000        0.000
## 164    WB   Wth       0.000        0.000
## 165    WB   Prs       0.000        0.000
## 166    WB   Pbl       0.000        0.000
## 167    WB   Dec       0.000        0.000
## 168    WB   Cfl       0.000        0.000
## 169   Pre   Cfl       0.000        0.000
## 170   Prs   Cfl       0.000        0.000
## 171   Esc   Dis       0.000        0.000
```

##### SMDS

```
##      From    To Weight_Boys Weight_Girls
## 1     Pbl   Cfl       0.790        0.469
## 2     Pre   Tol       0.723        0.423
## 3     Tol   Wth       0.599        0.713
## 4     Dec   Cfl       0.662        0.421
## 5     Pre   Dis       0.584        0.268
## 6     Esc   Pbl       0.582        0.233
## 7     Pre   Wth       0.445        0.508
## 8     Pre   Cfl       0.461        0.000
## 9     Dis   Cfl       0.403        0.442
## 10    Prs   Esc       0.343        0.408
## 11    Dec   Dis       0.384        0.235
## 12    Pre   Prs       0.382        0.354
## 13    Tol   Pbl       0.377        0.000
## 14    Pre   Esc       0.370        0.231
## 15    Pbl   Dec       0.286        0.369
## 16    Wth   Pbl       0.360        0.215
## 17    Tol   Dis       0.168        0.349
## 18    Esc   Dec       0.299        0.000
## 19    Prs   Dec       0.283        0.239
## 20    Low  Irri       0.195        0.269
## 21   Head Dizzy       0.215        0.263
## 22    Low  Nerv       0.255        0.215
## 23    Pbl   Dis       0.245        0.255
## 24    Low  Lone       0.247        0.206
## 25    Wth   Dec       0.244        0.000
## 26   Head  Stom       0.240        0.181
## 27   Irri  Nerv       0.103        0.240
## 28    Pre   Dec       0.000        0.227
## 29    Wth   Cfl       0.000        0.223
## 30   Lone    WB      -0.168       -0.200
## 31    Low    WB      -0.199       -0.160
## 32    Wth   Esc       0.189        0.000
## 33    Wth   Prs       0.186        0.000
## 34    Prs   Pbl       0.180        0.182
## 35    Tol   Dec       0.169        0.105
## 36    Tol   Esc       0.139        0.161
## 37    Low Sleep       0.154        0.000
## 38    Tol   Prs       0.000        0.151
## 39  Sleep Dizzy       0.000        0.146
## 40  Sleep    WB      -0.135       -0.111
## 41   Nerv Dizzy       0.134        0.000
## 42    Tol   Cfl       0.000        0.128
## 43   Stom  Back       0.000        0.127
## 44     WB   Esc      -0.109       -0.126
## 45    Pre   Pbl       0.000        0.117
## 46    Low Dizzy       0.000        0.116
## 47   Back Dizzy       0.068        0.109
## 48   Back   Pre       0.000        0.107
## 49   Head  Back       0.101        0.102
## 50   Stom  Nerv       0.091        0.000
## 51   Back Sleep       0.091        0.000
## 52   Stom   Low       0.087        0.000
## 53   Stom Dizzy       0.087        0.084
## 54   Back  Nerv       0.078        0.000
## 55   Lone   Esc       0.078        0.000
## 56   Stom Sleep       0.067        0.000
## 57   Head Sleep       0.061        0.000
## 58   Stom  Irri       0.061        0.000
## 59   Head   Low       0.000        0.000
## 60   Head  Irri       0.000        0.000
## 61   Head  Nerv       0.000        0.000
## 62   Head  Lone       0.000        0.000
## 63   Head    WB       0.000        0.000
## 64   Head   Pre       0.000        0.000
## 65   Head   Tol       0.000        0.000
## 66   Head   Wth       0.000        0.000
## 67   Head   Prs       0.000        0.000
## 68   Head   Esc       0.000        0.000
## 69   Head   Pbl       0.000        0.000
## 70   Head   Dec       0.000        0.000
## 71   Head   Dis       0.000        0.000
## 72   Head   Cfl       0.000        0.000
## 73   Stom  Lone       0.000        0.000
## 74   Stom    WB       0.000        0.000
## 75   Stom   Pre       0.000        0.000
## 76   Stom   Tol       0.000        0.000
## 77   Stom   Wth       0.000        0.000
## 78   Stom   Prs       0.000        0.000
## 79   Stom   Esc       0.000        0.000
## 80   Stom   Pbl       0.000        0.000
## 81   Stom   Dec       0.000        0.000
## 82   Stom   Dis       0.000        0.000
## 83   Stom   Cfl       0.000        0.000
## 84   Back   Low       0.000        0.000
## 85   Back  Irri       0.000        0.000
## 86   Back  Lone       0.000        0.000
## 87   Back    WB       0.000        0.000
## 88   Back   Tol       0.000        0.000
## 89   Back   Wth       0.000        0.000
## 90   Back   Prs       0.000        0.000
## 91   Back   Esc       0.000        0.000
## 92   Back   Pbl       0.000        0.000
## 93   Back   Dec       0.000        0.000
## 94   Back   Dis       0.000        0.000
## 95   Back   Cfl       0.000        0.000
## 96    Low   Pre       0.000        0.000
## 97    Low   Tol       0.000        0.000
## 98    Low   Wth       0.000        0.000
## 99    Low   Prs       0.000        0.000
## 100   Low   Esc       0.000        0.000
## 101   Low   Pbl       0.000        0.000
## 102   Low   Dec       0.000        0.000
## 103   Low   Dis       0.000        0.000
## 104   Low   Cfl       0.000        0.000
## 105  Irri Sleep       0.000        0.000
## 106  Irri Dizzy       0.000        0.000
## 107  Irri  Lone       0.000        0.000
## 108  Irri    WB       0.000        0.000
## 109  Irri   Pre       0.000        0.000
## 110  Irri   Tol       0.000        0.000
## 111  Irri   Wth       0.000        0.000
## 112  Irri   Prs       0.000        0.000
## 113  Irri   Esc       0.000        0.000
## 114  Irri   Pbl       0.000        0.000
## 115  Irri   Dec       0.000        0.000
## 116  Irri   Dis       0.000        0.000
## 117  Irri   Cfl       0.000        0.000
## 118  Nerv Sleep       0.000        0.000
## 119  Nerv  Lone       0.000        0.000
## 120  Nerv    WB       0.000        0.000
## 121  Nerv   Pre       0.000        0.000
## 122  Nerv   Tol       0.000        0.000
## 123  Nerv   Wth       0.000        0.000
## 124  Nerv   Prs       0.000        0.000
## 125  Nerv   Esc       0.000        0.000
## 126  Nerv   Pbl       0.000        0.000
## 127  Nerv   Dec       0.000        0.000
## 128  Nerv   Dis       0.000        0.000
## 129  Nerv   Cfl       0.000        0.000
## 130 Sleep  Lone       0.000        0.000
## 131 Sleep   Pre       0.000        0.000
## 132 Sleep   Tol       0.000        0.000
## 133 Sleep   Wth       0.000        0.000
## 134 Sleep   Prs       0.000        0.000
## 135 Sleep   Esc       0.000        0.000
## 136 Sleep   Pbl       0.000        0.000
## 137 Sleep   Dec       0.000        0.000
## 138 Sleep   Dis       0.000        0.000
## 139 Sleep   Cfl       0.000        0.000
## 140 Dizzy  Lone       0.000        0.000
## 141 Dizzy    WB       0.000        0.000
## 142 Dizzy   Pre       0.000        0.000
## 143 Dizzy   Tol       0.000        0.000
## 144 Dizzy   Wth       0.000        0.000
## 145 Dizzy   Prs       0.000        0.000
## 146 Dizzy   Esc       0.000        0.000
## 147 Dizzy   Pbl       0.000        0.000
## 148 Dizzy   Dec       0.000        0.000
## 149 Dizzy   Dis       0.000        0.000
## 150 Dizzy   Cfl       0.000        0.000
## 151  Lone   Pre       0.000        0.000
## 152  Lone   Tol       0.000        0.000
## 153  Lone   Wth       0.000        0.000
## 154  Lone   Prs       0.000        0.000
## 155  Lone   Pbl       0.000        0.000
## 156  Lone   Dec       0.000        0.000
## 157  Lone   Dis       0.000        0.000
## 158  Lone   Cfl       0.000        0.000
## 159    WB   Pre       0.000        0.000
## 160    WB   Tol       0.000        0.000
## 161    WB   Wth       0.000        0.000
## 162    WB   Prs       0.000        0.000
## 163    WB   Pbl       0.000        0.000
## 164    WB   Dec       0.000        0.000
## 165    WB   Dis       0.000        0.000
## 166    WB   Cfl       0.000        0.000
## 167   Wth   Dis       0.000        0.000
## 168   Prs   Dis       0.000        0.000
## 169   Prs   Cfl       0.000        0.000
## 170   Esc   Dis       0.000        0.000
## 171   Esc   Cfl       0.000        0.000
```

#### Centrality

##### IGDS

**The centrality estimates are relatively stable when the
correlation stability coefficient (CS coefficient) is ≥ 0.5**
while set at correlation stability = 0.7 (Epskamp et al.,
2018). Centrality indices with CS coefficient of 0.25-0.5 should be
interpreted with caution; CS coefficient < 0.25 indicated large
instability of the centrality index.

```
##   region scale group        centrality     CS_r07
## 1     IS  IGDS  Boys          strength 0.67268863
## 2     IS  IGDS  Boys         closeness 0.28374070
## 3     IS  IGDS  Boys       betweenness 0.04994687
## 4     IS  IGDS  Boys expectedInfluence 0.67268863
## 5     IS  IGDS Girls          strength 0.36149584
## 6     IS  IGDS Girls         closeness 0.00000000
## 7     IS  IGDS Girls       betweenness 0.00000000
## 8     IS  IGDS Girls expectedInfluence 0.43905817
```

##### SMDS

**The centrality estimates are relatively stable when the
correlation stability coefficient (CS coefficient) is ≥ 0.5**
while set at correlation stability = 0.7 (Epskamp et al.,
2018). Centrality indices with CS coefficient of 0.25-0.5 should be
interpreted with caution; CS coefficient < 0.25 indicated large
instability of the centrality index.

```
##    region scale group        centrality    CS_r07
## 9      IS  SMDS  Boys          strength 0.6726886
## 10     IS  SMDS  Boys         closeness 0.0000000
## 11     IS  SMDS  Boys       betweenness 0.0000000
## 12     IS  SMDS  Boys expectedInfluence 0.7502657
## 13     IS  SMDS Girls          strength 0.4390582
## 14     IS  SMDS Girls         closeness 0.0000000
## 15     IS  SMDS Girls       betweenness 0.0000000
## 16     IS  SMDS Girls expectedInfluence 0.4390582
```

#### Bootstrapped Edges

##### IGDS

###### Boys

###### Girls

##### SMDS

###### Boys

###### Girls

### MK

#### Figure

#### Edges

##### IGDS

```
##      From    To Weight_Boys Weight_Girls
## 1     Pbl   Cfl       0.363        0.846
## 2     Pre   Tol       0.350        0.608
## 3     Dec   Cfl       0.605        0.361
## 4     Dis   Cfl       0.206        0.564
## 5     Tol   Wth       0.422        0.550
## 6     Pbl   Dis       0.495        0.526
## 7     Dec   Dis       0.364        0.489
## 8     Wth   Dis       0.403        0.290
## 9     Pre   Wth       0.401        0.253
## 10    Pbl   Dec       0.000        0.396
## 11    Tol   Esc       0.000        0.392
## 12    Prs   Pbl       0.198        0.386
## 13    Prs   Dis       0.318        0.385
## 14    Pre   Esc       0.311        0.380
## 15    Tol   Prs       0.333        0.377
## 16    Low  Nerv       0.245        0.364
## 17    Wth   Dec       0.246        0.329
## 18    Pre   Dis       0.000        0.319
## 19    Wth   Cfl       0.307        0.000
## 20   Irri  Nerv       0.296        0.206
## 21    Prs   Dec       0.270        0.000
## 22   Lone    WB      -0.228       -0.266
## 23    Pre   Prs       0.178        0.229
## 24    Wth   Esc       0.000        0.229
## 25    Tol   Dis       0.000        0.224
## 26    Low  Lone       0.217        0.165
## 27    Low    WB      -0.176       -0.209
## 28    Low  Irri       0.142        0.205
## 29   Head Dizzy       0.203        0.187
## 30    Tol   Pbl       0.192        0.139
## 31   Stom Dizzy       0.190        0.080
## 32    Esc   Pbl       0.000        0.188
## 33    Esc   Dec       0.152        0.176
## 34    Tol   Dec       0.172        0.163
## 35  Sleep Dizzy       0.124        0.166
## 36   Head  Stom       0.000        0.165
## 37    Esc   Dis       0.000        0.160
## 38   Lone   Esc       0.000        0.153
## 39   Stom  Back       0.147        0.000
## 40   Head   Low       0.111        0.146
## 41   Back   Low       0.138        0.000
## 42   Nerv Sleep       0.133        0.135
## 43    Prs   Cfl       0.133        0.000
## 44  Sleep    WB      -0.123        0.000
## 45   Irri    WB       0.000       -0.107
## 46    Tol   Cfl       0.106        0.000
## 47     WB   Pre      -0.102        0.000
## 48   Back Dizzy       0.096        0.000
## 49   Head  Nerv       0.092        0.000
## 50   Head Sleep       0.090        0.000
## 51   Back  Irri       0.088        0.000
## 52  Sleep  Lone       0.000        0.083
## 53   Stom Sleep       0.000        0.076
## 54   Head  Back       0.071        0.000
## 55   Head  Irri       0.000        0.000
## 56   Head  Lone       0.000        0.000
## 57   Head    WB       0.000        0.000
## 58   Head   Pre       0.000        0.000
## 59   Head   Tol       0.000        0.000
## 60   Head   Wth       0.000        0.000
## 61   Head   Prs       0.000        0.000
## 62   Head   Esc       0.000        0.000
## 63   Head   Pbl       0.000        0.000
## 64   Head   Dec       0.000        0.000
## 65   Head   Dis       0.000        0.000
## 66   Head   Cfl       0.000        0.000
## 67   Stom   Low       0.000        0.000
## 68   Stom  Irri       0.000        0.000
## 69   Stom  Nerv       0.000        0.000
## 70   Stom  Lone       0.000        0.000
## 71   Stom    WB       0.000        0.000
## 72   Stom   Pre       0.000        0.000
## 73   Stom   Tol       0.000        0.000
## 74   Stom   Wth       0.000        0.000
## 75   Stom   Prs       0.000        0.000
## 76   Stom   Esc       0.000        0.000
## 77   Stom   Pbl       0.000        0.000
## 78   Stom   Dec       0.000        0.000
## 79   Stom   Dis       0.000        0.000
## 80   Stom   Cfl       0.000        0.000
## 81   Back  Nerv       0.000        0.000
## 82   Back Sleep       0.000        0.000
## 83   Back  Lone       0.000        0.000
## 84   Back    WB       0.000        0.000
## 85   Back   Pre       0.000        0.000
## 86   Back   Tol       0.000        0.000
## 87   Back   Wth       0.000        0.000
## 88   Back   Prs       0.000        0.000
## 89   Back   Esc       0.000        0.000
## 90   Back   Pbl       0.000        0.000
## 91   Back   Dec       0.000        0.000
## 92   Back   Dis       0.000        0.000
## 93   Back   Cfl       0.000        0.000
## 94    Low Sleep       0.000        0.000
## 95    Low Dizzy       0.000        0.000
## 96    Low   Pre       0.000        0.000
## 97    Low   Tol       0.000        0.000
## 98    Low   Wth       0.000        0.000
## 99    Low   Prs       0.000        0.000
## 100   Low   Esc       0.000        0.000
## 101   Low   Pbl       0.000        0.000
## 102   Low   Dec       0.000        0.000
## 103   Low   Dis       0.000        0.000
## 104   Low   Cfl       0.000        0.000
## 105  Irri Sleep       0.000        0.000
## 106  Irri Dizzy       0.000        0.000
## 107  Irri  Lone       0.000        0.000
## 108  Irri   Pre       0.000        0.000
## 109  Irri   Tol       0.000        0.000
## 110  Irri   Wth       0.000        0.000
## 111  Irri   Prs       0.000        0.000
## 112  Irri   Esc       0.000        0.000
## 113  Irri   Pbl       0.000        0.000
## 114  Irri   Dec       0.000        0.000
## 115  Irri   Dis       0.000        0.000
## 116  Irri   Cfl       0.000        0.000
## 117  Nerv Dizzy       0.000        0.000
## 118  Nerv  Lone       0.000        0.000
## 119  Nerv    WB       0.000        0.000
## 120  Nerv   Pre       0.000        0.000
## 121  Nerv   Tol       0.000        0.000
## 122  Nerv   Wth       0.000        0.000
## 123  Nerv   Prs       0.000        0.000
## 124  Nerv   Esc       0.000        0.000
## 125  Nerv   Pbl       0.000        0.000
## 126  Nerv   Dec       0.000        0.000
## 127  Nerv   Dis       0.000        0.000
## 128  Nerv   Cfl       0.000        0.000
## 129 Sleep   Pre       0.000        0.000
## 130 Sleep   Tol       0.000        0.000
## 131 Sleep   Wth       0.000        0.000
## 132 Sleep   Prs       0.000        0.000
## 133 Sleep   Esc       0.000        0.000
## 134 Sleep   Pbl       0.000        0.000
## 135 Sleep   Dec       0.000        0.000
## 136 Sleep   Dis       0.000        0.000
## 137 Sleep   Cfl       0.000        0.000
## 138 Dizzy  Lone       0.000        0.000
## 139 Dizzy    WB       0.000        0.000
## 140 Dizzy   Pre       0.000        0.000
## 141 Dizzy   Tol       0.000        0.000
## 142 Dizzy   Wth       0.000        0.000
## 143 Dizzy   Prs       0.000        0.000
## 144 Dizzy   Esc       0.000        0.000
## 145 Dizzy   Pbl       0.000        0.000
## 146 Dizzy   Dec       0.000        0.000
## 147 Dizzy   Dis       0.000        0.000
## 148 Dizzy   Cfl       0.000        0.000
## 149  Lone   Pre       0.000        0.000
## 150  Lone   Tol       0.000        0.000
## 151  Lone   Wth       0.000        0.000
## 152  Lone   Prs       0.000        0.000
## 153  Lone   Pbl       0.000        0.000
## 154  Lone   Dec       0.000        0.000
## 155  Lone   Dis       0.000        0.000
## 156  Lone   Cfl       0.000        0.000
## 157    WB   Tol       0.000        0.000
## 158    WB   Wth       0.000        0.000
## 159    WB   Prs       0.000        0.000
## 160    WB   Esc       0.000        0.000
## 161    WB   Pbl       0.000        0.000
## 162    WB   Dec       0.000        0.000
## 163    WB   Dis       0.000        0.000
## 164    WB   Cfl       0.000        0.000
## 165   Pre   Pbl       0.000        0.000
## 166   Pre   Dec       0.000        0.000
## 167   Pre   Cfl       0.000        0.000
## 168   Wth   Prs       0.000        0.000
## 169   Wth   Pbl       0.000        0.000
## 170   Prs   Esc       0.000        0.000
## 171   Esc   Cfl       0.000        0.000
```

##### SMDS

```
##      From    To Weight_Boys Weight_Girls
## 1     Dec   Cfl       0.583        0.495
## 2     Tol   Wth       0.246        0.550
## 3     Pbl   Cfl       0.506        0.503
## 4     Pre   Wth       0.449        0.206
## 5     Pre   Tol       0.385        0.439
## 6     Pbl   Dec       0.000        0.428
## 7     Tol   Pbl       0.396        0.105
## 8     Wth   Dis       0.384        0.136
## 9     Dec   Dis       0.371        0.149
## 10    Low  Nerv       0.242        0.357
## 11    Wth   Esc       0.186        0.336
## 12    Pbl   Dis       0.141        0.301
## 13   Irri  Nerv       0.292        0.182
## 14    Prs   Esc       0.247        0.277
## 15    Pre   Esc       0.000        0.270
## 16    Wth   Cfl       0.265        0.000
## 17   Lone    WB      -0.211       -0.255
## 18    Prs   Dec       0.243        0.000
## 19    Pre   Dis       0.000        0.233
## 20    Tol   Cfl       0.224        0.000
## 21    Low  Lone       0.219        0.161
## 22    Pre   Cfl       0.214        0.134
## 23    Pre   Prs       0.117        0.207
## 24   Head Dizzy       0.203        0.182
## 25    Low  Irri       0.150        0.198
## 26    Low    WB      -0.180       -0.196
## 27   Stom Dizzy       0.189        0.000
## 28    Wth   Dec       0.184        0.082
## 29     WB   Esc       0.000       -0.181
## 30   Head  Stom       0.000        0.166
## 31    Pre   Dec       0.163        0.000
## 32    Tol   Prs       0.159        0.000
## 33  Sleep Dizzy       0.121        0.155
## 34   Back   Low       0.147        0.000
## 35   Head   Low       0.118        0.146
## 36   Stom  Back       0.146        0.000
## 37    Prs   Dis       0.000        0.145
## 38    Dis   Cfl       0.138        0.094
## 39    Esc   Pbl       0.000        0.137
## 40   Nerv Sleep       0.132        0.123
## 41    Low   Esc       0.092        0.110
## 42  Sleep    WB      -0.107        0.000
## 43   Lone   Cfl       0.000        0.102
## 44    Low Sleep       0.101        0.000
## 45   Back Dizzy       0.097        0.000
## 46   Head  Nerv       0.091        0.000
## 47   Head Sleep       0.087        0.000
## 48    Tol   Esc       0.086        0.000
## 49   Back  Irri       0.085        0.000
## 50   Irri    WB       0.000       -0.085
## 51     WB   Prs       0.000       -0.083
## 52    Esc   Dis       0.000        0.080
## 53   Nerv   Esc       0.000        0.079
## 54   Head  Back       0.072        0.000
## 55   Stom Sleep       0.000        0.072
## 56   Head  Irri       0.000        0.000
## 57   Head  Lone       0.000        0.000
## 58   Head    WB       0.000        0.000
## 59   Head   Pre       0.000        0.000
## 60   Head   Tol       0.000        0.000
## 61   Head   Wth       0.000        0.000
## 62   Head   Prs       0.000        0.000
## 63   Head   Esc       0.000        0.000
## 64   Head   Pbl       0.000        0.000
## 65   Head   Dec       0.000        0.000
## 66   Head   Dis       0.000        0.000
## 67   Head   Cfl       0.000        0.000
## 68   Stom   Low       0.000        0.000
## 69   Stom  Irri       0.000        0.000
## 70   Stom  Nerv       0.000        0.000
## 71   Stom  Lone       0.000        0.000
## 72   Stom    WB       0.000        0.000
## 73   Stom   Pre       0.000        0.000
## 74   Stom   Tol       0.000        0.000
## 75   Stom   Wth       0.000        0.000
## 76   Stom   Prs       0.000        0.000
## 77   Stom   Esc       0.000        0.000
## 78   Stom   Pbl       0.000        0.000
## 79   Stom   Dec       0.000        0.000
## 80   Stom   Dis       0.000        0.000
## 81   Stom   Cfl       0.000        0.000
## 82   Back  Nerv       0.000        0.000
## 83   Back Sleep       0.000        0.000
## 84   Back  Lone       0.000        0.000
## 85   Back    WB       0.000        0.000
## 86   Back   Pre       0.000        0.000
## 87   Back   Tol       0.000        0.000
## 88   Back   Wth       0.000        0.000
## 89   Back   Prs       0.000        0.000
## 90   Back   Esc       0.000        0.000
## 91   Back   Pbl       0.000        0.000
## 92   Back   Dec       0.000        0.000
## 93   Back   Dis       0.000        0.000
## 94   Back   Cfl       0.000        0.000
## 95    Low Dizzy       0.000        0.000
## 96    Low   Pre       0.000        0.000
## 97    Low   Tol       0.000        0.000
## 98    Low   Wth       0.000        0.000
## 99    Low   Prs       0.000        0.000
## 100   Low   Pbl       0.000        0.000
## 101   Low   Dec       0.000        0.000
## 102   Low   Dis       0.000        0.000
## 103   Low   Cfl       0.000        0.000
## 104  Irri Sleep       0.000        0.000
## 105  Irri Dizzy       0.000        0.000
## 106  Irri  Lone       0.000        0.000
## 107  Irri   Pre       0.000        0.000
## 108  Irri   Tol       0.000        0.000
## 109  Irri   Wth       0.000        0.000
## 110  Irri   Prs       0.000        0.000
## 111  Irri   Esc       0.000        0.000
## 112  Irri   Pbl       0.000        0.000
## 113  Irri   Dec       0.000        0.000
## 114  Irri   Dis       0.000        0.000
## 115  Irri   Cfl       0.000        0.000
## 116  Nerv Dizzy       0.000        0.000
## 117  Nerv  Lone       0.000        0.000
## 118  Nerv    WB       0.000        0.000
## 119  Nerv   Pre       0.000        0.000
## 120  Nerv   Tol       0.000        0.000
## 121  Nerv   Wth       0.000        0.000
## 122  Nerv   Prs       0.000        0.000
## 123  Nerv   Pbl       0.000        0.000
## 124  Nerv   Dec       0.000        0.000
## 125  Nerv   Dis       0.000        0.000
## 126  Nerv   Cfl       0.000        0.000
## 127 Sleep  Lone       0.000        0.000
## 128 Sleep   Pre       0.000        0.000
## 129 Sleep   Tol       0.000        0.000
## 130 Sleep   Wth       0.000        0.000
## 131 Sleep   Prs       0.000        0.000
## 132 Sleep   Esc       0.000        0.000
## 133 Sleep   Pbl       0.000        0.000
## 134 Sleep   Dec       0.000        0.000
## 135 Sleep   Dis       0.000        0.000
## 136 Sleep   Cfl       0.000        0.000
## 137 Dizzy  Lone       0.000        0.000
## 138 Dizzy    WB       0.000        0.000
## 139 Dizzy   Pre       0.000        0.000
## 140 Dizzy   Tol       0.000        0.000
## 141 Dizzy   Wth       0.000        0.000
## 142 Dizzy   Prs       0.000        0.000
## 143 Dizzy   Esc       0.000        0.000
## 144 Dizzy   Pbl       0.000        0.000
## 145 Dizzy   Dec       0.000        0.000
## 146 Dizzy   Dis       0.000        0.000
## 147 Dizzy   Cfl       0.000        0.000
## 148  Lone   Pre       0.000        0.000
## 149  Lone   Tol       0.000        0.000
## 150  Lone   Wth       0.000        0.000
## 151  Lone   Prs       0.000        0.000
## 152  Lone   Esc       0.000        0.000
## 153  Lone   Pbl       0.000        0.000
## 154  Lone   Dec       0.000        0.000
## 155  Lone   Dis       0.000        0.000
## 156    WB   Pre       0.000        0.000
## 157    WB   Tol       0.000        0.000
## 158    WB   Wth       0.000        0.000
## 159    WB   Pbl       0.000        0.000
## 160    WB   Dec       0.000        0.000
## 161    WB   Dis       0.000        0.000
## 162    WB   Cfl       0.000        0.000
## 163   Pre   Pbl       0.000        0.000
## 164   Tol   Dec       0.000        0.000
## 165   Tol   Dis       0.000        0.000
## 166   Wth   Prs       0.000        0.000
## 167   Wth   Pbl       0.000        0.000
## 168   Prs   Pbl       0.000        0.000
## 169   Prs   Cfl       0.000        0.000
## 170   Esc   Dec       0.000        0.000
## 171   Esc   Cfl       0.000        0.000
```

#### Centrality

##### IGDS

**The centrality estimates are relatively stable when the
correlation stability coefficient (CS coefficient) is ≥ 0.5**
while set at correlation stability = 0.7 (Epskamp et al.,
2018). Centrality indices with CS coefficient of 0.25-0.5 should be
interpreted with caution; CS coefficient < 0.25 indicated large
instability of the centrality index.

```
##   region scale group        centrality     CS_r07
## 1     MK  IGDS  Boys          strength 0.51719577
## 2     MK  IGDS  Boys         closeness 0.00000000
## 3     MK  IGDS  Boys       betweenness 0.00000000
## 4     MK  IGDS  Boys expectedInfluence 0.59391534
## 5     MK  IGDS Girls          strength 0.59483871
## 6     MK  IGDS Girls         closeness 0.00000000
## 7     MK  IGDS Girls       betweenness 0.05032258
## 8     MK  IGDS Girls expectedInfluence 0.67225806
```

##### SMDS

**The centrality estimates are relatively stable when the
correlation stability coefficient (CS coefficient) is ≥ 0.5**
while set at correlation stability = 0.7 (Epskamp et al.,
2018). Centrality indices with CS coefficient of 0.25-0.5 should be
interpreted with caution; CS coefficient < 0.25 indicated large
instability of the centrality index.

```
##    region scale group        centrality     CS_r07
## 9      MK  SMDS  Boys          strength 0.43915344
## 10     MK  SMDS  Boys         closeness 0.05026455
## 11     MK  SMDS  Boys       betweenness 0.05026455
## 12     MK  SMDS  Boys expectedInfluence 0.51719577
## 13     MK  SMDS Girls          strength 0.36129032
## 14     MK  SMDS Girls         closeness 0.00000000
## 15     MK  SMDS Girls       betweenness 0.05032258
## 16     MK  SMDS Girls expectedInfluence 0.51612903
```

#### Bootstrapped Edges

##### IGDS

###### Boys

###### Girls

##### SMDS

###### Boys

###### Girls

### MT

#### Figure

#### Edges

##### IGDS

```
##      From    To Weight_Boys Weight_Girls
## 1     Pbl   Cfl       0.750        0.955
## 2     Pbl   Dec       0.149        0.651
## 3     Dis   Cfl       0.500        0.259
## 4     Pre   Tol       0.358        0.478
## 5     Pre   Esc       0.426        0.360
## 6     Tol   Esc       0.062        0.410
## 7     Pbl   Dis       0.175        0.400
## 8     Pre   Wth       0.273        0.389
## 9     Tol   Wth       0.388        0.367
## 10    Tol   Pbl       0.368        0.290
## 11    Pre   Prs       0.085        0.361
## 12    Wth   Cfl       0.147        0.350
## 13    Wth   Dec       0.233        0.343
## 14    Wth   Prs       0.098        0.308
## 15    Dec   Cfl       0.300        0.263
## 16    Dec   Dis       0.277        0.160
## 17    Prs   Dec       0.273        0.193
## 18    Low  Irri       0.267        0.240
## 19    Prs   Dis       0.224        0.255
## 20   Head  Stom       0.214        0.247
## 21   Irri  Nerv       0.238        0.210
## 22   Lone    WB      -0.238       -0.229
## 23    Prs   Esc       0.238        0.000
## 24    Prs   Pbl       0.190        0.238
## 25    Wth   Dis       0.230        0.192
## 26    Low  Lone       0.203        0.227
## 27    Low  Nerv       0.184        0.219
## 28   Head Dizzy       0.163        0.210
## 29    Wth   Pbl       0.202        0.105
## 30    Esc   Dec       0.196        0.000
## 31    Tol   Dis       0.169        0.186
## 32    Pre   Pbl       0.185        0.176
## 33    Wth   Esc       0.182        0.114
## 34    Low    WB      -0.085       -0.179
## 35   Nerv Sleep       0.171        0.083
## 36    Esc   Cfl       0.168        0.000
## 37    Low Dizzy       0.097        0.164
## 38    Pre   Dis       0.000        0.164
## 39  Sleep Dizzy       0.149        0.156
## 40   Head  Back       0.149        0.147
## 41   Back   Low       0.149        0.134
## 42   Stom  Back       0.148        0.145
## 43   Stom Dizzy       0.091        0.146
## 44    Tol   Dec       0.142        0.111
## 45    Prs   Cfl       0.125        0.000
## 46    Esc   Pbl       0.098        0.119
## 47    Tol   Prs       0.114        0.000
## 48    Pre   Dec       0.112        0.000
## 49    Tol   Cfl       0.109        0.000
## 50   Nerv  Lone       0.105        0.091
## 51   Irri Sleep       0.000        0.089
## 52  Sleep   Dec       0.000        0.084
## 53   Lone   Esc       0.000        0.082
## 54   Head   Low       0.080        0.067
## 55  Sleep    WB       0.000       -0.078
## 56   Head Sleep       0.069        0.073
## 57  Sleep  Lone       0.070        0.063
## 58   Stom  Nerv       0.062        0.057
## 59   Nerv   Esc       0.000        0.061
## 60   Head  Nerv       0.000        0.058
## 61   Irri Dizzy       0.000        0.057
## 62     WB   Tol      -0.056        0.000
## 63   Nerv Dizzy       0.000        0.053
## 64   Irri   Wth       0.049        0.000
## 65   Head  Irri       0.000        0.000
## 66   Head  Lone       0.000        0.000
## 67   Head    WB       0.000        0.000
## 68   Head   Pre       0.000        0.000
## 69   Head   Tol       0.000        0.000
## 70   Head   Wth       0.000        0.000
## 71   Head   Prs       0.000        0.000
## 72   Head   Esc       0.000        0.000
## 73   Head   Pbl       0.000        0.000
## 74   Head   Dec       0.000        0.000
## 75   Head   Dis       0.000        0.000
## 76   Head   Cfl       0.000        0.000
## 77   Stom   Low       0.000        0.000
## 78   Stom  Irri       0.000        0.000
## 79   Stom Sleep       0.000        0.000
## 80   Stom  Lone       0.000        0.000
## 81   Stom    WB       0.000        0.000
## 82   Stom   Pre       0.000        0.000
## 83   Stom   Tol       0.000        0.000
## 84   Stom   Wth       0.000        0.000
## 85   Stom   Prs       0.000        0.000
## 86   Stom   Esc       0.000        0.000
## 87   Stom   Pbl       0.000        0.000
## 88   Stom   Dec       0.000        0.000
## 89   Stom   Dis       0.000        0.000
## 90   Stom   Cfl       0.000        0.000
## 91   Back  Irri       0.000        0.000
## 92   Back  Nerv       0.000        0.000
## 93   Back Sleep       0.000        0.000
## 94   Back Dizzy       0.000        0.000
## 95   Back  Lone       0.000        0.000
## 96   Back    WB       0.000        0.000
## 97   Back   Pre       0.000        0.000
## 98   Back   Tol       0.000        0.000
## 99   Back   Wth       0.000        0.000
## 100  Back   Prs       0.000        0.000
## 101  Back   Esc       0.000        0.000
## 102  Back   Pbl       0.000        0.000
## 103  Back   Dec       0.000        0.000
## 104  Back   Dis       0.000        0.000
## 105  Back   Cfl       0.000        0.000
## 106   Low Sleep       0.000        0.000
## 107   Low   Pre       0.000        0.000
## 108   Low   Tol       0.000        0.000
## 109   Low   Wth       0.000        0.000
## 110   Low   Prs       0.000        0.000
## 111   Low   Esc       0.000        0.000
## 112   Low   Pbl       0.000        0.000
## 113   Low   Dec       0.000        0.000
## 114   Low   Dis       0.000        0.000
## 115   Low   Cfl       0.000        0.000
## 116  Irri  Lone       0.000        0.000
## 117  Irri    WB       0.000        0.000
## 118  Irri   Pre       0.000        0.000
## 119  Irri   Tol       0.000        0.000
## 120  Irri   Prs       0.000        0.000
## 121  Irri   Esc       0.000        0.000
## 122  Irri   Pbl       0.000        0.000
## 123  Irri   Dec       0.000        0.000
## 124  Irri   Dis       0.000        0.000
## 125  Irri   Cfl       0.000        0.000
## 126  Nerv    WB       0.000        0.000
## 127  Nerv   Pre       0.000        0.000
## 128  Nerv   Tol       0.000        0.000
## 129  Nerv   Wth       0.000        0.000
## 130  Nerv   Prs       0.000        0.000
## 131  Nerv   Pbl       0.000        0.000
## 132  Nerv   Dec       0.000        0.000
## 133  Nerv   Dis       0.000        0.000
## 134  Nerv   Cfl       0.000        0.000
## 135 Sleep   Pre       0.000        0.000
## 136 Sleep   Tol       0.000        0.000
## 137 Sleep   Wth       0.000        0.000
## 138 Sleep   Prs       0.000        0.000
## 139 Sleep   Esc       0.000        0.000
## 140 Sleep   Pbl       0.000        0.000
## 141 Sleep   Dis       0.000        0.000
## 142 Sleep   Cfl       0.000        0.000
## 143 Dizzy  Lone       0.000        0.000
## 144 Dizzy    WB       0.000        0.000
## 145 Dizzy   Pre       0.000        0.000
## 146 Dizzy   Tol       0.000        0.000
## 147 Dizzy   Wth       0.000        0.000
## 148 Dizzy   Prs       0.000        0.000
## 149 Dizzy   Esc       0.000        0.000
## 150 Dizzy   Pbl       0.000        0.000
## 151 Dizzy   Dec       0.000        0.000
## 152 Dizzy   Dis       0.000        0.000
## 153 Dizzy   Cfl       0.000        0.000
## 154  Lone   Pre       0.000        0.000
## 155  Lone   Tol       0.000        0.000
## 156  Lone   Wth       0.000        0.000
## 157  Lone   Prs       0.000        0.000
## 158  Lone   Pbl       0.000        0.000
## 159  Lone   Dec       0.000        0.000
## 160  Lone   Dis       0.000        0.000
## 161  Lone   Cfl       0.000        0.000
## 162    WB   Pre       0.000        0.000
## 163    WB   Wth       0.000        0.000
## 164    WB   Prs       0.000        0.000
## 165    WB   Esc       0.000        0.000
## 166    WB   Pbl       0.000        0.000
## 167    WB   Dec       0.000        0.000
## 168    WB   Dis       0.000        0.000
## 169    WB   Cfl       0.000        0.000
## 170   Pre   Cfl       0.000        0.000
## 171   Esc   Dis       0.000        0.000
```

##### SMDS

```
##      From    To Weight_Boys Weight_Girls
## 1     Dec   Cfl       0.693        0.496
## 2     Pbl   Cfl       0.499        0.672
## 3     Tol   Wth       0.605        0.595
## 4     Pre   Tol       0.356        0.580
## 5     Pbl   Dec       0.267        0.569
## 6     Wth   Cfl       0.324        0.173
## 7     Dec   Dis       0.321        0.147
## 8     Pre   Prs       0.296        0.294
## 9     Pre   Wth       0.293        0.274
## 10    Dis   Cfl       0.290        0.133
## 11    Prs   Pbl       0.251        0.277
## 12    Low  Irri       0.272        0.243
## 13    Pre   Dis       0.266        0.106
## 14    Wth   Esc       0.264        0.261
## 15    Prs   Dis       0.000        0.264
## 16    Prs   Esc       0.249        0.201
## 17   Lone    WB      -0.241       -0.214
## 18   Head  Stom       0.230        0.240
## 19   Irri  Nerv       0.240        0.193
## 20    Low  Nerv       0.182        0.223
## 21    Low  Lone       0.208        0.219
## 22    Pre   Esc       0.216        0.183
## 23   Head Dizzy       0.167        0.211
## 24    Tol   Cfl       0.196        0.000
## 25    Wth   Pbl       0.194        0.069
## 26    Esc   Pbl       0.170        0.184
## 27    Pre   Cfl       0.085        0.183
## 28    Pre   Dec       0.119        0.182
## 29   Nerv Sleep       0.178        0.072
## 30    Prs   Dec       0.173        0.000
## 31    Low    WB      -0.093       -0.170
## 32   Stom  Back       0.166        0.137
## 33    Tol   Prs       0.166        0.000
## 34    Tol   Pbl       0.165        0.156
## 35    Low Dizzy       0.101        0.162
## 36    Pbl   Dis       0.138        0.161
## 37    Wth   Dis       0.000        0.160
## 38   Head  Back       0.158        0.144
## 39   Back   Low       0.157        0.129
## 40  Sleep Dizzy       0.150        0.156
## 41   Stom Dizzy       0.102        0.146
## 42     WB   Esc      -0.064       -0.130
## 43   Lone   Esc       0.000        0.129
## 44    Esc   Cfl       0.070        0.114
## 45   Nerv  Lone       0.111        0.078
## 46   Nerv   Esc       0.111        0.000
## 47    Tol   Dec       0.108        0.000
## 48    Prs   Cfl       0.108        0.000
## 49   Head   Low       0.082        0.000
## 50   Stom  Nerv       0.081        0.000
## 51   Irri Sleep       0.000        0.076
## 52   Head Sleep       0.074        0.063
## 53  Sleep  Lone       0.073        0.057
## 54  Sleep    WB       0.000       -0.064
## 55   Head  Nerv       0.062        0.000
## 56   Irri Dizzy       0.000        0.055
## 57   Head  Irri       0.000        0.000
## 58   Head  Lone       0.000        0.000
## 59   Head    WB       0.000        0.000
## 60   Head   Pre       0.000        0.000
## 61   Head   Tol       0.000        0.000
## 62   Head   Wth       0.000        0.000
## 63   Head   Prs       0.000        0.000
## 64   Head   Esc       0.000        0.000
## 65   Head   Pbl       0.000        0.000
## 66   Head   Dec       0.000        0.000
## 67   Head   Dis       0.000        0.000
## 68   Head   Cfl       0.000        0.000
## 69   Stom   Low       0.000        0.000
## 70   Stom  Irri       0.000        0.000
## 71   Stom Sleep       0.000        0.000
## 72   Stom  Lone       0.000        0.000
## 73   Stom    WB       0.000        0.000
## 74   Stom   Pre       0.000        0.000
## 75   Stom   Tol       0.000        0.000
## 76   Stom   Wth       0.000        0.000
## 77   Stom   Prs       0.000        0.000
## 78   Stom   Esc       0.000        0.000
## 79   Stom   Pbl       0.000        0.000
## 80   Stom   Dec       0.000        0.000
## 81   Stom   Dis       0.000        0.000
## 82   Stom   Cfl       0.000        0.000
## 83   Back  Irri       0.000        0.000
## 84   Back  Nerv       0.000        0.000
## 85   Back Sleep       0.000        0.000
## 86   Back Dizzy       0.000        0.000
## 87   Back  Lone       0.000        0.000
## 88   Back    WB       0.000        0.000
## 89   Back   Pre       0.000        0.000
## 90   Back   Tol       0.000        0.000
## 91   Back   Wth       0.000        0.000
## 92   Back   Prs       0.000        0.000
## 93   Back   Esc       0.000        0.000
## 94   Back   Pbl       0.000        0.000
## 95   Back   Dec       0.000        0.000
## 96   Back   Dis       0.000        0.000
## 97   Back   Cfl       0.000        0.000
## 98    Low Sleep       0.000        0.000
## 99    Low   Pre       0.000        0.000
## 100   Low   Tol       0.000        0.000
## 101   Low   Wth       0.000        0.000
## 102   Low   Prs       0.000        0.000
## 103   Low   Esc       0.000        0.000
## 104   Low   Pbl       0.000        0.000
## 105   Low   Dec       0.000        0.000
## 106   Low   Dis       0.000        0.000
## 107   Low   Cfl       0.000        0.000
## 108  Irri  Lone       0.000        0.000
## 109  Irri    WB       0.000        0.000
## 110  Irri   Pre       0.000        0.000
## 111  Irri   Tol       0.000        0.000
## 112  Irri   Wth       0.000        0.000
## 113  Irri   Prs       0.000        0.000
## 114  Irri   Esc       0.000        0.000
## 115  Irri   Pbl       0.000        0.000
## 116  Irri   Dec       0.000        0.000
## 117  Irri   Dis       0.000        0.000
## 118  Irri   Cfl       0.000        0.000
## 119  Nerv Dizzy       0.000        0.000
## 120  Nerv    WB       0.000        0.000
## 121  Nerv   Pre       0.000        0.000
## 122  Nerv   Tol       0.000        0.000
## 123  Nerv   Wth       0.000        0.000
## 124  Nerv   Prs       0.000        0.000
## 125  Nerv   Pbl       0.000        0.000
## 126  Nerv   Dec       0.000        0.000
## 127  Nerv   Dis       0.000        0.000
## 128  Nerv   Cfl       0.000        0.000
## 129 Sleep   Pre       0.000        0.000
## 130 Sleep   Tol       0.000        0.000
## 131 Sleep   Wth       0.000        0.000
## 132 Sleep   Prs       0.000        0.000
## 133 Sleep   Esc       0.000        0.000
## 134 Sleep   Pbl       0.000        0.000
## 135 Sleep   Dec       0.000        0.000
## 136 Sleep   Dis       0.000        0.000
## 137 Sleep   Cfl       0.000        0.000
## 138 Dizzy  Lone       0.000        0.000
## 139 Dizzy    WB       0.000        0.000
## 140 Dizzy   Pre       0.000        0.000
## 141 Dizzy   Tol       0.000        0.000
## 142 Dizzy   Wth       0.000        0.000
## 143 Dizzy   Prs       0.000        0.000
## 144 Dizzy   Esc       0.000        0.000
## 145 Dizzy   Pbl       0.000        0.000
## 146 Dizzy   Dec       0.000        0.000
## 147 Dizzy   Dis       0.000        0.000
## 148 Dizzy   Cfl       0.000        0.000
## 149  Lone   Pre       0.000        0.000
## 150  Lone   Tol       0.000        0.000
## 151  Lone   Wth       0.000        0.000
## 152  Lone   Prs       0.000        0.000
## 153  Lone   Pbl       0.000        0.000
## 154  Lone   Dec       0.000        0.000
## 155  Lone   Dis       0.000        0.000
## 156  Lone   Cfl       0.000        0.000
## 157    WB   Pre       0.000        0.000
## 158    WB   Tol       0.000        0.000
## 159    WB   Wth       0.000        0.000
## 160    WB   Prs       0.000        0.000
## 161    WB   Pbl       0.000        0.000
## 162    WB   Dec       0.000        0.000
## 163    WB   Dis       0.000        0.000
## 164    WB   Cfl       0.000        0.000
## 165   Pre   Pbl       0.000        0.000
## 166   Tol   Esc       0.000        0.000
## 167   Tol   Dis       0.000        0.000
## 168   Wth   Prs       0.000        0.000
## 169   Wth   Dec       0.000        0.000
## 170   Esc   Dec       0.000        0.000
## 171   Esc   Dis       0.000        0.000
```

#### Centrality

##### IGDS

**The centrality estimates are relatively stable when the
correlation stability coefficient (CS coefficient) is ≥ 0.5**
while set at correlation stability = 0.7 (Epskamp et al.,
2018). Centrality indices with CS coefficient of 0.25-0.5 should be
interpreted with caution; CS coefficient < 0.25 indicated large
instability of the centrality index.

```
##   region scale group        centrality    CS_r07
## 1     MT  IGDS  Boys          strength 0.5169972
## 2     MT  IGDS  Boys         closeness 0.0000000
## 3     MT  IGDS  Boys       betweenness 0.0000000
## 4     MT  IGDS  Boys expectedInfluence 0.5941926
## 5     MT  IGDS Girls          strength 0.6724003
## 6     MT  IGDS Girls         closeness 0.0000000
## 7     MT  IGDS Girls       betweenness 0.0000000
## 8     MT  IGDS Girls expectedInfluence 0.7498045
```

##### SMDS

**The centrality estimates are relatively stable when the
correlation stability coefficient (CS coefficient) is ≥ 0.5**
while set at correlation stability = 0.7 (Epskamp et al.,
2018). Centrality indices with CS coefficient of 0.25-0.5 should be
interpreted with caution; CS coefficient < 0.25 indicated large
instability of the centrality index.

```
##    region scale group        centrality     CS_r07
## 9      MT  SMDS  Boys          strength 0.59419263
## 10     MT  SMDS  Boys         closeness 0.05028329
## 11     MT  SMDS  Boys       betweenness 0.05028329
## 12     MT  SMDS  Boys expectedInfluence 0.67209632
## 13     MT  SMDS Girls          strength 0.59421423
## 14     MT  SMDS Girls         closeness 0.36121970
## 15     MT  SMDS Girls       betweenness 0.00000000
## 16     MT  SMDS Girls expectedInfluence 0.67240031
```

#### Bootstrapped Edges

##### IGDS

###### Boys

###### Girls

##### SMDS

###### Boys

###### Girls

### NL

#### Figure

#### Edges

##### IGDS

```
##      From    To Weight_Boys Weight_Girls
## 1     Tol   Wth       0.577        0.764
## 2     Dec   Cfl       0.596        0.714
## 3     Pbl   Cfl       0.677        0.581
## 4     Pre   Tol       0.359        0.558
## 5     Pbl   Dec       0.193        0.553
## 6     Pre   Wth       0.353        0.530
## 7     Prs   Dis       0.286        0.526
## 8     Prs   Dec       0.434        0.179
## 9     Dis   Cfl       0.407        0.186
## 10    Prs   Esc       0.089        0.405
## 11    Prs   Cfl       0.346        0.404
## 12    Pre   Prs       0.383        0.250
## 13    Esc   Dec       0.245        0.379
## 14    Pre   Dis       0.376        0.195
## 15    Wth   Pbl       0.358        0.210
## 16    Tol   Esc       0.095        0.340
## 17    Low  Lone       0.309        0.333
## 18    Dec   Dis       0.306        0.183
## 19    Prs   Pbl       0.103        0.290
## 20    Wth   Cfl       0.287        0.000
## 21    Tol   Dis       0.286        0.198
## 22    Wth   Prs       0.274        0.237
## 23    Pre   Pbl       0.253        0.106
## 24    Low  Irri       0.225        0.248
## 25    Wth   Esc       0.203        0.246
## 26    Wth   Dis       0.147        0.244
## 27   Head Dizzy       0.216        0.242
## 28    Pre   Esc       0.191        0.239
## 29    Low    WB      -0.170       -0.233
## 30    Tol   Pbl       0.181        0.229
## 31    Tol   Dec       0.132        0.220
## 32   Lone    WB      -0.164       -0.214
## 33   Head  Stom       0.212        0.170
## 34   Irri  Nerv       0.165        0.199
## 35    Tol   Cfl       0.000        0.194
## 36    Tol   Prs       0.193        0.138
## 37   Lone   Esc       0.179        0.162
## 38   Stom Dizzy       0.172        0.153
## 39    Low   Cfl       0.000        0.172
## 40    Pbl   Dis       0.161        0.147
## 41    Wth   Dec       0.149        0.126
## 42    Pre   Dec       0.089        0.145
## 43  Dizzy   Cfl       0.141        0.000
## 44    Low Dizzy       0.097        0.135
## 45   Head  Back       0.084        0.131
## 46   Irri    WB      -0.129       -0.126
## 47   Nerv Sleep       0.091        0.114
## 48    Low  Nerv       0.111        0.079
## 49   Back Dizzy       0.108        0.087
## 50   Nerv Dizzy       0.061        0.108
## 51   Irri   Pbl       0.103        0.000
## 52   Stom  Nerv       0.048        0.100
## 53   Lone   Dec       0.099        0.000
## 54    Esc   Pbl       0.000        0.099
## 55   Irri Sleep       0.098        0.091
## 56   Stom  Back       0.097        0.088
## 57   Head  Irri       0.095        0.083
## 58    Low   Esc       0.081        0.093
## 59  Sleep Dizzy       0.091        0.061
## 60  Dizzy   Pbl       0.088        0.000
## 61    Low Sleep       0.000        0.085
## 62  Sleep    WB      -0.082       -0.074
## 63   Back   Low       0.079        0.000
## 64   Head Sleep       0.075        0.065
## 65   Back   Wth       0.000        0.068
## 66   Irri  Lone       0.065        0.000
## 67   Stom Sleep       0.063        0.000
## 68  Sleep   Esc       0.062        0.000
## 69   Head   Low       0.061        0.057
## 70   Irri   Esc       0.061        0.000
## 71   Back Sleep       0.060        0.000
## 72   Nerv  Lone       0.060        0.057
## 73   Nerv   Esc       0.056        0.000
## 74     WB   Tol      -0.052        0.000
## 75   Head  Nerv       0.000        0.000
## 76   Head  Lone       0.000        0.000
## 77   Head    WB       0.000        0.000
## 78   Head   Pre       0.000        0.000
## 79   Head   Tol       0.000        0.000
## 80   Head   Wth       0.000        0.000
## 81   Head   Prs       0.000        0.000
## 82   Head   Esc       0.000        0.000
## 83   Head   Pbl       0.000        0.000
## 84   Head   Dec       0.000        0.000
## 85   Head   Dis       0.000        0.000
## 86   Head   Cfl       0.000        0.000
## 87   Stom   Low       0.000        0.000
## 88   Stom  Irri       0.000        0.000
## 89   Stom  Lone       0.000        0.000
## 90   Stom    WB       0.000        0.000
## 91   Stom   Pre       0.000        0.000
## 92   Stom   Tol       0.000        0.000
## 93   Stom   Wth       0.000        0.000
## 94   Stom   Prs       0.000        0.000
## 95   Stom   Esc       0.000        0.000
## 96   Stom   Pbl       0.000        0.000
## 97   Stom   Dec       0.000        0.000
## 98   Stom   Dis       0.000        0.000
## 99   Stom   Cfl       0.000        0.000
## 100  Back  Irri       0.000        0.000
## 101  Back  Nerv       0.000        0.000
## 102  Back  Lone       0.000        0.000
## 103  Back    WB       0.000        0.000
## 104  Back   Pre       0.000        0.000
## 105  Back   Tol       0.000        0.000
## 106  Back   Prs       0.000        0.000
## 107  Back   Esc       0.000        0.000
## 108  Back   Pbl       0.000        0.000
## 109  Back   Dec       0.000        0.000
## 110  Back   Dis       0.000        0.000
## 111  Back   Cfl       0.000        0.000
## 112   Low   Pre       0.000        0.000
## 113   Low   Tol       0.000        0.000
## 114   Low   Wth       0.000        0.000
## 115   Low   Prs       0.000        0.000
## 116   Low   Pbl       0.000        0.000
## 117   Low   Dec       0.000        0.000
## 118   Low   Dis       0.000        0.000
## 119  Irri Dizzy       0.000        0.000
## 120  Irri   Pre       0.000        0.000
## 121  Irri   Tol       0.000        0.000
## 122  Irri   Wth       0.000        0.000
## 123  Irri   Prs       0.000        0.000
## 124  Irri   Dec       0.000        0.000
## 125  Irri   Dis       0.000        0.000
## 126  Irri   Cfl       0.000        0.000
## 127  Nerv    WB       0.000        0.000
## 128  Nerv   Pre       0.000        0.000
## 129  Nerv   Tol       0.000        0.000
## 130  Nerv   Wth       0.000        0.000
## 131  Nerv   Prs       0.000        0.000
## 132  Nerv   Pbl       0.000        0.000
## 133  Nerv   Dec       0.000        0.000
## 134  Nerv   Dis       0.000        0.000
## 135  Nerv   Cfl       0.000        0.000
## 136 Sleep  Lone       0.000        0.000
## 137 Sleep   Pre       0.000        0.000
## 138 Sleep   Tol       0.000        0.000
## 139 Sleep   Wth       0.000        0.000
## 140 Sleep   Prs       0.000        0.000
## 141 Sleep   Pbl       0.000        0.000
## 142 Sleep   Dec       0.000        0.000
## 143 Sleep   Dis       0.000        0.000
## 144 Sleep   Cfl       0.000        0.000
## 145 Dizzy  Lone       0.000        0.000
## 146 Dizzy    WB       0.000        0.000
## 147 Dizzy   Pre       0.000        0.000
## 148 Dizzy   Tol       0.000        0.000
## 149 Dizzy   Wth       0.000        0.000
## 150 Dizzy   Prs       0.000        0.000
## 151 Dizzy   Esc       0.000        0.000
## 152 Dizzy   Dec       0.000        0.000
## 153 Dizzy   Dis       0.000        0.000
## 154  Lone   Pre       0.000        0.000
## 155  Lone   Tol       0.000        0.000
## 156  Lone   Wth       0.000        0.000
## 157  Lone   Prs       0.000        0.000
## 158  Lone   Pbl       0.000        0.000
## 159  Lone   Dis       0.000        0.000
## 160  Lone   Cfl       0.000        0.000
## 161    WB   Pre       0.000        0.000
## 162    WB   Wth       0.000        0.000
## 163    WB   Prs       0.000        0.000
## 164    WB   Esc       0.000        0.000
## 165    WB   Pbl       0.000        0.000
## 166    WB   Dec       0.000        0.000
## 167    WB   Dis       0.000        0.000
## 168    WB   Cfl       0.000        0.000
## 169   Pre   Cfl       0.000        0.000
## 170   Esc   Dis       0.000        0.000
## 171   Esc   Cfl       0.000        0.000
```

##### SMDS

```
##      From    To Weight_Boys Weight_Girls
## 1     Tol   Wth       0.683        0.576
## 2     Pbl   Cfl       0.457        0.666
## 3     Dec   Cfl       0.578        0.186
## 4     Tol   Cfl       0.493        0.217
## 5     Prs   Dis       0.183        0.491
## 6     Pbl   Dec       0.446        0.360
## 7     Prs   Dec       0.418        0.097
## 8     Prs   Esc       0.376        0.208
## 9     Pre   Wth       0.299        0.360
## 10    Tol   Esc       0.329        0.226
## 11    Low  Lone       0.309        0.328
## 12    Tol   Dis       0.318        0.119
## 13    Pbl   Dis       0.318        0.234
## 14    Pre   Tol       0.317        0.167
## 15    Dec   Dis       0.297        0.167
## 16    Wth   Esc       0.158        0.275
## 17    Esc   Dec       0.159        0.253
## 18    Esc   Pbl       0.250        0.081
## 19    Low  Irri       0.225        0.243
## 20    Wth   Pbl       0.243        0.099
## 21   Head Dizzy       0.213        0.242
## 22    Tol   Dec       0.186        0.238
## 23    Low    WB      -0.169       -0.228
## 24    Wth   Cfl       0.226        0.000
## 25    Tol   Pbl       0.221        0.103
## 26   Lone    WB      -0.166       -0.212
## 27   Head  Stom       0.211        0.164
## 28    Wth   Dis       0.207        0.086
## 29    Wth   Dec       0.158        0.202
## 30   Irri  Nerv       0.174        0.199
## 31   Stom Dizzy       0.168        0.150
## 32    Dis   Cfl       0.168        0.000
## 33   Lone   Esc       0.164        0.144
## 34    Pre   Esc       0.162        0.122
## 35    Pre   Dis       0.154        0.000
## 36    Low   Cfl       0.000        0.144
## 37    Prs   Pbl       0.091        0.139
## 38    Pre   Prs       0.000        0.136
## 39   Irri    WB      -0.132       -0.123
## 40   Head  Back       0.081        0.130
## 41    Low Dizzy       0.095        0.130
## 42    Wth   Prs       0.128        0.084
## 43   Irri   Wth       0.000        0.121
## 44   Nerv Sleep       0.100        0.118
## 45    Esc   Dis       0.052        0.115
## 46    Low   Esc       0.094        0.114
## 47    Low  Nerv       0.109        0.000
## 48   Nerv Dizzy       0.059        0.108
## 49   Irri Sleep       0.105        0.093
## 50   Back Dizzy       0.103        0.088
## 51   Nerv   Esc       0.081        0.099
## 52   Head  Irri       0.097        0.086
## 53   Stom  Nerv       0.053        0.097
## 54   Irri   Dec       0.097        0.000
## 55   Stom  Back       0.094        0.078
## 56  Sleep Dizzy       0.087        0.064
## 57  Sleep    WB      -0.086       -0.068
## 58    Tol   Prs       0.085        0.000
## 59  Sleep   Esc       0.080        0.000
## 60   Back   Low       0.076        0.000
## 61   Head Sleep       0.074        0.066
## 62   Irri  Lone       0.074        0.000
## 63   Nerv   Prs       0.073        0.000
## 64  Dizzy   Pbl       0.071        0.000
## 65   Nerv  Lone       0.068        0.062
## 66   Stom Sleep       0.067        0.000
## 67    Pre   Dec       0.062        0.000
## 68   Head   Low       0.059        0.000
## 69   Back Sleep       0.059        0.000
## 70   Irri   Pbl       0.058        0.000
## 71  Dizzy   Esc       0.000        0.052
## 72   Irri   Dis       0.046        0.000
## 73   Irri Dizzy       0.042        0.000
## 74   Head  Nerv       0.000        0.000
## 75   Head  Lone       0.000        0.000
## 76   Head    WB       0.000        0.000
## 77   Head   Pre       0.000        0.000
## 78   Head   Tol       0.000        0.000
## 79   Head   Wth       0.000        0.000
## 80   Head   Prs       0.000        0.000
## 81   Head   Esc       0.000        0.000
## 82   Head   Pbl       0.000        0.000
## 83   Head   Dec       0.000        0.000
## 84   Head   Dis       0.000        0.000
## 85   Head   Cfl       0.000        0.000
## 86   Stom   Low       0.000        0.000
## 87   Stom  Irri       0.000        0.000
## 88   Stom  Lone       0.000        0.000
## 89   Stom    WB       0.000        0.000
## 90   Stom   Pre       0.000        0.000
## 91   Stom   Tol       0.000        0.000
## 92   Stom   Wth       0.000        0.000
## 93   Stom   Prs       0.000        0.000
## 94   Stom   Esc       0.000        0.000
## 95   Stom   Pbl       0.000        0.000
## 96   Stom   Dec       0.000        0.000
## 97   Stom   Dis       0.000        0.000
## 98   Stom   Cfl       0.000        0.000
## 99   Back  Irri       0.000        0.000
## 100  Back  Nerv       0.000        0.000
## 101  Back  Lone       0.000        0.000
## 102  Back    WB       0.000        0.000
## 103  Back   Pre       0.000        0.000
## 104  Back   Tol       0.000        0.000
## 105  Back   Wth       0.000        0.000
## 106  Back   Prs       0.000        0.000
## 107  Back   Esc       0.000        0.000
## 108  Back   Pbl       0.000        0.000
## 109  Back   Dec       0.000        0.000
## 110  Back   Dis       0.000        0.000
## 111  Back   Cfl       0.000        0.000
## 112   Low Sleep       0.000        0.000
## 113   Low   Pre       0.000        0.000
## 114   Low   Tol       0.000        0.000
## 115   Low   Wth       0.000        0.000
## 116   Low   Prs       0.000        0.000
## 117   Low   Pbl       0.000        0.000
## 118   Low   Dec       0.000        0.000
## 119   Low   Dis       0.000        0.000
## 120  Irri   Pre       0.000        0.000
## 121  Irri   Tol       0.000        0.000
## 122  Irri   Prs       0.000        0.000
## 123  Irri   Esc       0.000        0.000
## 124  Irri   Cfl       0.000        0.000
## 125  Nerv    WB       0.000        0.000
## 126  Nerv   Pre       0.000        0.000
## 127  Nerv   Tol       0.000        0.000
## 128  Nerv   Wth       0.000        0.000
## 129  Nerv   Pbl       0.000        0.000
## 130  Nerv   Dec       0.000        0.000
## 131  Nerv   Dis       0.000        0.000
## 132  Nerv   Cfl       0.000        0.000
## 133 Sleep  Lone       0.000        0.000
## 134 Sleep   Pre       0.000        0.000
## 135 Sleep   Tol       0.000        0.000
## 136 Sleep   Wth       0.000        0.000
## 137 Sleep   Prs       0.000        0.000
## 138 Sleep   Pbl       0.000        0.000
## 139 Sleep   Dec       0.000        0.000
## 140 Sleep   Dis       0.000        0.000
## 141 Sleep   Cfl       0.000        0.000
## 142 Dizzy  Lone       0.000        0.000
## 143 Dizzy    WB       0.000        0.000
## 144 Dizzy   Pre       0.000        0.000
## 145 Dizzy   Tol       0.000        0.000
## 146 Dizzy   Wth       0.000        0.000
## 147 Dizzy   Prs       0.000        0.000
## 148 Dizzy   Dec       0.000        0.000
## 149 Dizzy   Dis       0.000        0.000
## 150 Dizzy   Cfl       0.000        0.000
## 151  Lone   Pre       0.000        0.000
## 152  Lone   Tol       0.000        0.000
## 153  Lone   Wth       0.000        0.000
## 154  Lone   Prs       0.000        0.000
## 155  Lone   Pbl       0.000        0.000
## 156  Lone   Dec       0.000        0.000
## 157  Lone   Dis       0.000        0.000
## 158  Lone   Cfl       0.000        0.000
## 159    WB   Pre       0.000        0.000
## 160    WB   Tol       0.000        0.000
## 161    WB   Wth       0.000        0.000
## 162    WB   Prs       0.000        0.000
## 163    WB   Esc       0.000        0.000
## 164    WB   Pbl       0.000        0.000
## 165    WB   Dec       0.000        0.000
## 166    WB   Dis       0.000        0.000
## 167    WB   Cfl       0.000        0.000
## 168   Pre   Pbl       0.000        0.000
## 169   Pre   Cfl       0.000        0.000
## 170   Prs   Cfl       0.000        0.000
## 171   Esc   Cfl       0.000        0.000
```

#### Centrality

##### IGDS

**The centrality estimates are relatively stable when the
correlation stability coefficient (CS coefficient) is ≥ 0.5**
while set at correlation stability = 0.7 (Epskamp et al.,
2018). Centrality indices with CS coefficient of 0.25-0.5 should be
interpreted with caution; CS coefficient < 0.25 indicated large
instability of the centrality index.

```
##   region scale group        centrality     CS_r07
## 1     NL  IGDS  Boys          strength 0.59447005
## 2     NL  IGDS  Boys         closeness 0.20532514
## 3     NL  IGDS  Boys       betweenness 0.05017921
## 4     NL  IGDS  Boys expectedInfluence 0.67229903
## 5     NL  IGDS Girls          strength 0.36078717
## 6     NL  IGDS Girls         closeness 0.20553936
## 7     NL  IGDS Girls       betweenness 0.00000000
## 8     NL  IGDS Girls expectedInfluence 0.43877551
```

##### SMDS

**The centrality estimates are relatively stable when the
correlation stability coefficient (CS coefficient) is ≥ 0.5**
while set at correlation stability = 0.7 (Epskamp et al.,
2018). Centrality indices with CS coefficient of 0.25-0.5 should be
interpreted with caution; CS coefficient < 0.25 indicated large
instability of the centrality index.

```
##    region scale group        centrality    CS_r07
## 9      NL  SMDS  Boys          strength 0.5166411
## 10     NL  SMDS  Boys         closeness 0.3609831
## 11     NL  SMDS  Boys       betweenness 0.0000000
## 12     NL  SMDS  Boys expectedInfluence 0.5944700
## 13     NL  SMDS Girls          strength 0.2835277
## 14     NL  SMDS Girls         closeness 0.3607872
## 15     NL  SMDS Girls       betweenness 0.0000000
## 16     NL  SMDS Girls expectedInfluence 0.4387755
```

#### Bootstrapped Edges

##### IGDS

###### Boys

###### Girls

##### SMDS

###### Boys

###### Girls

### GB-SCT

#### Figure

#### Edges

##### IGDS

```
##      From    To Weight_Boys Weight_Girls
## 1     Pbl   Cfl       0.763        0.902
## 2     Tol   Wth       0.671        0.810
## 3     Dec   Cfl       0.376        0.740
## 4     Dis   Cfl       0.699        0.443
## 5     Dec   Dis       0.304        0.662
## 6     Pre   Wth       0.285        0.570
## 7     Pre   Tol       0.529        0.560
## 8     Wth   Dis       0.538        0.242
## 9     Wth   Prs       0.505        0.353
## 10    Esc   Dec       0.194        0.478
## 11    Pre   Esc       0.100        0.438
## 12    Prs   Dis       0.000        0.358
## 13    Low  Nerv       0.355        0.202
## 14    Pre   Prs       0.208        0.321
## 15    Wth   Cfl       0.000        0.310
## 16    Low  Irri       0.172        0.295
## 17    Wth   Dec       0.294        0.180
## 18   Head Dizzy       0.174        0.292
## 19    Tol   Dec       0.151        0.280
## 20    Tol   Prs       0.147        0.276
## 21    Tol   Pbl       0.000        0.274
## 22    Low  Lone       0.268        0.252
## 23    Pbl   Dis       0.000        0.258
## 24    Tol   Esc       0.072        0.257
## 25    Pbl   Dec       0.257        0.203
## 26    Esc   Pbl       0.224        0.177
## 27    Wth   Pbl       0.079        0.223
## 28    Esc   Dis       0.000        0.223
## 29    Low    WB      -0.191       -0.219
## 30    Prs   Cfl       0.216        0.000
## 31   Head  Stom       0.209        0.115
## 32   Lone    WB      -0.205       -0.195
## 33    Prs   Pbl       0.153        0.202
## 34    Prs   Dec       0.000        0.196
## 35   Stom Dizzy       0.154        0.192
## 36  Sleep    WB      -0.148       -0.126
## 37    Pre   Dis       0.000        0.147
## 38   Stom  Back       0.107        0.142
## 39     WB   Esc      -0.142       -0.122
## 40    Tol   Dis       0.000        0.140
## 41   Stom   Low       0.000        0.131
## 42   Nerv Dizzy       0.000        0.121
## 43    Wth   Esc       0.119        0.000
## 44  Sleep Dizzy       0.066        0.116
## 45   Irri   Pbl       0.112        0.000
## 46   Head   Low       0.000        0.111
## 47   Head Sleep       0.107        0.000
## 48   Irri Dizzy       0.000        0.107
## 49    Low   Esc       0.000        0.106
## 50   Head  Back       0.102        0.000
## 51   Stom   Pbl       0.099        0.000
## 52  Sleep  Lone       0.099        0.000
## 53   Nerv  Lone       0.093        0.091
## 54    Prs   Esc       0.000        0.092
## 55    Pre   Pbl       0.086        0.000
## 56     WB   Tol      -0.083        0.000
## 57   Nerv Sleep       0.000        0.081
## 58   Back Dizzy       0.077        0.000
## 59   Irri Sleep       0.073        0.000
## 60   Stom Sleep       0.055        0.000
## 61   Head  Irri       0.000        0.000
## 62   Head  Nerv       0.000        0.000
## 63   Head  Lone       0.000        0.000
## 64   Head    WB       0.000        0.000
## 65   Head   Pre       0.000        0.000
## 66   Head   Tol       0.000        0.000
## 67   Head   Wth       0.000        0.000
## 68   Head   Prs       0.000        0.000
## 69   Head   Esc       0.000        0.000
## 70   Head   Pbl       0.000        0.000
## 71   Head   Dec       0.000        0.000
## 72   Head   Dis       0.000        0.000
## 73   Head   Cfl       0.000        0.000
## 74   Stom  Irri       0.000        0.000
## 75   Stom  Nerv       0.000        0.000
## 76   Stom  Lone       0.000        0.000
## 77   Stom    WB       0.000        0.000
## 78   Stom   Pre       0.000        0.000
## 79   Stom   Tol       0.000        0.000
## 80   Stom   Wth       0.000        0.000
## 81   Stom   Prs       0.000        0.000
## 82   Stom   Esc       0.000        0.000
## 83   Stom   Dec       0.000        0.000
## 84   Stom   Dis       0.000        0.000
## 85   Stom   Cfl       0.000        0.000
## 86   Back   Low       0.000        0.000
## 87   Back  Irri       0.000        0.000
## 88   Back  Nerv       0.000        0.000
## 89   Back Sleep       0.000        0.000
## 90   Back  Lone       0.000        0.000
## 91   Back    WB       0.000        0.000
## 92   Back   Pre       0.000        0.000
## 93   Back   Tol       0.000        0.000
## 94   Back   Wth       0.000        0.000
## 95   Back   Prs       0.000        0.000
## 96   Back   Esc       0.000        0.000
## 97   Back   Pbl       0.000        0.000
## 98   Back   Dec       0.000        0.000
## 99   Back   Dis       0.000        0.000
## 100  Back   Cfl       0.000        0.000
## 101   Low Sleep       0.000        0.000
## 102   Low Dizzy       0.000        0.000
## 103   Low   Pre       0.000        0.000
## 104   Low   Tol       0.000        0.000
## 105   Low   Wth       0.000        0.000
## 106   Low   Prs       0.000        0.000
## 107   Low   Pbl       0.000        0.000
## 108   Low   Dec       0.000        0.000
## 109   Low   Dis       0.000        0.000
## 110   Low   Cfl       0.000        0.000
## 111  Irri  Nerv       0.000        0.000
## 112  Irri  Lone       0.000        0.000
## 113  Irri    WB       0.000        0.000
## 114  Irri   Pre       0.000        0.000
## 115  Irri   Tol       0.000        0.000
## 116  Irri   Wth       0.000        0.000
## 117  Irri   Prs       0.000        0.000
## 118  Irri   Esc       0.000        0.000
## 119  Irri   Dec       0.000        0.000
## 120  Irri   Dis       0.000        0.000
## 121  Irri   Cfl       0.000        0.000
## 122  Nerv    WB       0.000        0.000
## 123  Nerv   Pre       0.000        0.000
## 124  Nerv   Tol       0.000        0.000
## 125  Nerv   Wth       0.000        0.000
## 126  Nerv   Prs       0.000        0.000
## 127  Nerv   Esc       0.000        0.000
## 128  Nerv   Pbl       0.000        0.000
## 129  Nerv   Dec       0.000        0.000
## 130  Nerv   Dis       0.000        0.000
## 131  Nerv   Cfl       0.000        0.000
## 132 Sleep   Pre       0.000        0.000
## 133 Sleep   Tol       0.000        0.000
## 134 Sleep   Wth       0.000        0.000
## 135 Sleep   Prs       0.000        0.000
## 136 Sleep   Esc       0.000        0.000
## 137 Sleep   Pbl       0.000        0.000
## 138 Sleep   Dec       0.000        0.000
## 139 Sleep   Dis       0.000        0.000
## 140 Sleep   Cfl       0.000        0.000
## 141 Dizzy  Lone       0.000        0.000
## 142 Dizzy    WB       0.000        0.000
## 143 Dizzy   Pre       0.000        0.000
## 144 Dizzy   Tol       0.000        0.000
## 145 Dizzy   Wth       0.000        0.000
## 146 Dizzy   Prs       0.000        0.000
## 147 Dizzy   Esc       0.000        0.000
## 148 Dizzy   Pbl       0.000        0.000
## 149 Dizzy   Dec       0.000        0.000
## 150 Dizzy   Dis       0.000        0.000
## 151 Dizzy   Cfl       0.000        0.000
## 152  Lone   Pre       0.000        0.000
## 153  Lone   Tol       0.000        0.000
## 154  Lone   Wth       0.000        0.000
## 155  Lone   Prs       0.000        0.000
## 156  Lone   Esc       0.000        0.000
## 157  Lone   Pbl       0.000        0.000
## 158  Lone   Dec       0.000        0.000
## 159  Lone   Dis       0.000        0.000
## 160  Lone   Cfl       0.000        0.000
## 161    WB   Pre       0.000        0.000
## 162    WB   Wth       0.000        0.000
## 163    WB   Prs       0.000        0.000
## 164    WB   Pbl       0.000        0.000
## 165    WB   Dec       0.000        0.000
## 166    WB   Dis       0.000        0.000
## 167    WB   Cfl       0.000        0.000
## 168   Pre   Dec       0.000        0.000
## 169   Pre   Cfl       0.000        0.000
## 170   Tol   Cfl       0.000        0.000
## 171   Esc   Cfl       0.000        0.000
```

##### SMDS

```
##      From    To Weight_Boys Weight_Girls
## 1     Pre   Tol       0.744        0.743
## 2     Pbl   Cfl       0.712        0.571
## 3     Dec   Cfl       0.668        0.438
## 4     Tol   Wth       0.631        0.612
## 5     Prs   Dec       0.563        0.292
## 6     Pre   Wth       0.483        0.316
## 7     Pbl   Dec       0.377        0.457
## 8     Dec   Dis       0.372        0.084
## 9     Prs   Esc       0.356        0.254
## 10    Low  Nerv       0.348        0.200
## 11    Pbl   Dis       0.265        0.292
## 12    Low  Irri       0.171        0.290
## 13   Head Dizzy       0.180        0.285
## 14    Pre   Pbl       0.276        0.000
## 15    Wth   Pbl       0.271        0.153
## 16    Low  Lone       0.270        0.240
## 17    Esc   Pbl       0.239        0.261
## 18    Dis   Cfl       0.256        0.131
## 19    Wth   Dec       0.108        0.249
## 20    Esc   Dis       0.248        0.100
## 21    Wth   Dis       0.000        0.234
## 22    Tol   Dis       0.229        0.113
## 23    Low    WB      -0.195       -0.225
## 24    Tol   Pbl       0.219        0.146
## 25   Head  Stom       0.205        0.115
## 26   Lone    WB      -0.200       -0.174
## 27   Nerv   Esc       0.199        0.000
## 28   Stom Dizzy       0.161        0.182
## 29    Prs   Pbl       0.177        0.104
## 30    Tol   Esc       0.173        0.127
## 31    Wth   Esc       0.170        0.099
## 32    Tol   Cfl       0.129        0.164
## 33    Esc   Dec       0.000        0.164
## 34    Pre   Dis       0.157        0.000
## 35    Pre   Prs       0.153        0.000
## 36    Tol   Prs       0.000        0.143
## 37  Sleep    WB      -0.136       -0.112
## 38   Stom  Back       0.100        0.132
## 39    Prs   Dis       0.000        0.125
## 40   Stom   Low       0.000        0.124
## 41     WB   Dis      -0.116        0.000
## 42    Pre   Cfl       0.116        0.000
## 43   Irri   Pbl       0.115        0.000
## 44   Lone   Esc       0.059        0.114
## 45   Nerv Dizzy       0.059        0.113
## 46   Head   Low       0.000        0.107
## 47  Sleep Dizzy       0.070        0.106
## 48   Head Sleep       0.103        0.000
## 49   Nerv  Lone       0.103        0.077
## 50  Sleep  Lone       0.101        0.000
## 51   Head  Back       0.100        0.000
## 52   Irri Dizzy       0.058        0.098
## 53   Back Dizzy       0.091        0.000
## 54    Pre   Esc       0.076        0.000
## 55   Nerv Sleep       0.000        0.074
## 56   Irri  Nerv       0.067        0.000
## 57   Irri Sleep       0.067        0.000
## 58    Pre   Dec       0.064        0.000
## 59   Lone   Prs       0.063        0.000
## 60   Head  Irri       0.000        0.000
## 61   Head  Nerv       0.000        0.000
## 62   Head  Lone       0.000        0.000
## 63   Head    WB       0.000        0.000
## 64   Head   Pre       0.000        0.000
## 65   Head   Tol       0.000        0.000
## 66   Head   Wth       0.000        0.000
## 67   Head   Prs       0.000        0.000
## 68   Head   Esc       0.000        0.000
## 69   Head   Pbl       0.000        0.000
## 70   Head   Dec       0.000        0.000
## 71   Head   Dis       0.000        0.000
## 72   Head   Cfl       0.000        0.000
## 73   Stom  Irri       0.000        0.000
## 74   Stom  Nerv       0.000        0.000
## 75   Stom Sleep       0.000        0.000
## 76   Stom  Lone       0.000        0.000
## 77   Stom    WB       0.000        0.000
## 78   Stom   Pre       0.000        0.000
## 79   Stom   Tol       0.000        0.000
## 80   Stom   Wth       0.000        0.000
## 81   Stom   Prs       0.000        0.000
## 82   Stom   Esc       0.000        0.000
## 83   Stom   Pbl       0.000        0.000
## 84   Stom   Dec       0.000        0.000
## 85   Stom   Dis       0.000        0.000
## 86   Stom   Cfl       0.000        0.000
## 87   Back   Low       0.000        0.000
## 88   Back  Irri       0.000        0.000
## 89   Back  Nerv       0.000        0.000
## 90   Back Sleep       0.000        0.000
## 91   Back  Lone       0.000        0.000
## 92   Back    WB       0.000        0.000
## 93   Back   Pre       0.000        0.000
## 94   Back   Tol       0.000        0.000
## 95   Back   Wth       0.000        0.000
## 96   Back   Prs       0.000        0.000
## 97   Back   Esc       0.000        0.000
## 98   Back   Pbl       0.000        0.000
## 99   Back   Dec       0.000        0.000
## 100  Back   Dis       0.000        0.000
## 101  Back   Cfl       0.000        0.000
## 102   Low Sleep       0.000        0.000
## 103   Low Dizzy       0.000        0.000
## 104   Low   Pre       0.000        0.000
## 105   Low   Tol       0.000        0.000
## 106   Low   Wth       0.000        0.000
## 107   Low   Prs       0.000        0.000
## 108   Low   Esc       0.000        0.000
## 109   Low   Pbl       0.000        0.000
## 110   Low   Dec       0.000        0.000
## 111   Low   Dis       0.000        0.000
## 112   Low   Cfl       0.000        0.000
## 113  Irri  Lone       0.000        0.000
## 114  Irri    WB       0.000        0.000
## 115  Irri   Pre       0.000        0.000
## 116  Irri   Tol       0.000        0.000
## 117  Irri   Wth       0.000        0.000
## 118  Irri   Prs       0.000        0.000
## 119  Irri   Esc       0.000        0.000
## 120  Irri   Dec       0.000        0.000
## 121  Irri   Dis       0.000        0.000
## 122  Irri   Cfl       0.000        0.000
## 123  Nerv    WB       0.000        0.000
## 124  Nerv   Pre       0.000        0.000
## 125  Nerv   Tol       0.000        0.000
## 126  Nerv   Wth       0.000        0.000
## 127  Nerv   Prs       0.000        0.000
## 128  Nerv   Pbl       0.000        0.000
## 129  Nerv   Dec       0.000        0.000
## 130  Nerv   Dis       0.000        0.000
## 131  Nerv   Cfl       0.000        0.000
## 132 Sleep   Pre       0.000        0.000
## 133 Sleep   Tol       0.000        0.000
## 134 Sleep   Wth       0.000        0.000
## 135 Sleep   Prs       0.000        0.000
## 136 Sleep   Esc       0.000        0.000
## 137 Sleep   Pbl       0.000        0.000
## 138 Sleep   Dec       0.000        0.000
## 139 Sleep   Dis       0.000        0.000
## 140 Sleep   Cfl       0.000        0.000
## 141 Dizzy  Lone       0.000        0.000
## 142 Dizzy    WB       0.000        0.000
## 143 Dizzy   Pre       0.000        0.000
## 144 Dizzy   Tol       0.000        0.000
## 145 Dizzy   Wth       0.000        0.000
## 146 Dizzy   Prs       0.000        0.000
## 147 Dizzy   Esc       0.000        0.000
## 148 Dizzy   Pbl       0.000        0.000
## 149 Dizzy   Dec       0.000        0.000
## 150 Dizzy   Dis       0.000        0.000
## 151 Dizzy   Cfl       0.000        0.000
## 152  Lone   Pre       0.000        0.000
## 153  Lone   Tol       0.000        0.000
## 154  Lone   Wth       0.000        0.000
## 155  Lone   Pbl       0.000        0.000
## 156  Lone   Dec       0.000        0.000
## 157  Lone   Dis       0.000        0.000
## 158  Lone   Cfl       0.000        0.000
## 159    WB   Pre       0.000        0.000
## 160    WB   Tol       0.000        0.000
## 161    WB   Wth       0.000        0.000
## 162    WB   Prs       0.000        0.000
## 163    WB   Esc       0.000        0.000
## 164    WB   Pbl       0.000        0.000
## 165    WB   Dec       0.000        0.000
## 166    WB   Cfl       0.000        0.000
## 167   Tol   Dec       0.000        0.000
## 168   Wth   Prs       0.000        0.000
## 169   Wth   Cfl       0.000        0.000
## 170   Prs   Cfl       0.000        0.000
## 171   Esc   Cfl       0.000        0.000
```

#### Centrality

##### IGDS

**The centrality estimates are relatively stable when the
correlation stability coefficient (CS coefficient) is ≥ 0.5**
while set at correlation stability = 0.7 (Epskamp et al.,
2018). Centrality indices with CS coefficient of 0.25-0.5 should be
interpreted with caution; CS coefficient < 0.25 indicated large
instability of the centrality index.

```
##   region scale group        centrality     CS_r07
## 1 GB-SCT  IGDS  Boys          strength 0.67229130
## 2 GB-SCT  IGDS  Boys         closeness 0.04973357
## 3 GB-SCT  IGDS  Boys       betweenness 0.00000000
## 4 GB-SCT  IGDS  Boys expectedInfluence 0.74955595
## 5 GB-SCT  IGDS Girls          strength 0.59444444
## 6 GB-SCT  IGDS Girls         closeness 0.05000000
## 7 GB-SCT  IGDS Girls       betweenness 0.05000000
## 8 GB-SCT  IGDS Girls expectedInfluence 0.67222222
```

##### SMDS

**The centrality estimates are relatively stable when the
correlation stability coefficient (CS coefficient) is ≥ 0.5**
while set at correlation stability = 0.7 (Epskamp et al.,
2018). Centrality indices with CS coefficient of 0.25-0.5 should be
interpreted with caution; CS coefficient < 0.25 indicated large
instability of the centrality index.

```
##    region scale group        centrality    CS_r07
## 9  GB-SCT  SMDS  Boys          strength 0.5941385
## 10 GB-SCT  SMDS  Boys         closeness 0.2051510
## 11 GB-SCT  SMDS  Boys       betweenness 0.0000000
## 12 GB-SCT  SMDS  Boys expectedInfluence 0.6722913
## 13 GB-SCT  SMDS Girls          strength 0.5166667
## 14 GB-SCT  SMDS Girls         closeness 0.0500000
## 15 GB-SCT  SMDS Girls       betweenness 0.0500000
## 16 GB-SCT  SMDS Girls expectedInfluence 0.6722222
```

#### Bootstrapped Edges

##### IGDS

###### Boys

###### Girls

##### SMDS

###### Boys

###### Girls

### SI

#### Figure

#### Edges

##### IGDS

```
##      From    To Weight_Boys Weight_Girls
## 1     Pbl   Cfl       0.486        0.819
## 2     Pre   Wth       0.411        0.719
## 3     Tol   Wth       0.714        0.655
## 4     Pbl   Dis       0.599        0.549
## 5     Prs   Dis       0.550        0.495
## 6     Tol   Dis       0.286        0.531
## 7     Pre   Tol       0.509        0.479
## 8     Esc   Cfl       0.199        0.505
## 9     Esc   Dec       0.198        0.499
## 10    Prs   Pbl       0.319        0.484
## 11    Dis   Cfl       0.472        0.234
## 12    Dec   Cfl       0.459        0.361
## 13    Low  Irri       0.410        0.442
## 14    Tol   Pbl       0.431        0.423
## 15    Dec   Dis       0.430        0.392
## 16    Wth   Prs       0.337        0.381
## 17    Pre   Esc       0.379        0.352
## 18    Prs   Dec       0.311        0.374
## 19    Pre   Cfl       0.372        0.151
## 20    Pre   Prs       0.145        0.348
## 21    Wth   Dec       0.298        0.343
## 22    Pre   Dec       0.123        0.338
## 23    Pbl   Dec       0.323        0.331
## 24    Pre   Pbl       0.255        0.330
## 25    Low  Lone       0.258        0.294
## 26   Head Dizzy       0.228        0.277
## 27   Irri  Nerv       0.270        0.246
## 28    Prs   Esc       0.265        0.123
## 29    Prs   Cfl       0.264        0.255
## 30    Wth   Esc       0.209        0.262
## 31    Wth   Cfl       0.257        0.172
## 32    Pre   Dis       0.132        0.215
## 33   Lone    WB      -0.204       -0.212
## 34    Esc   Dis       0.000        0.211
## 35    Esc   Pbl       0.191        0.206
## 36    Wth   Pbl       0.204        0.000
## 37    Wth   Dis       0.000        0.197
## 38    Tol   Prs       0.178        0.108
## 39   Head  Stom       0.174        0.160
## 40    Low    WB      -0.117       -0.132
## 41   Stom Dizzy       0.068        0.125
## 42   Back Dizzy       0.122        0.097
## 43   Head  Back       0.102        0.121
## 44  Sleep    WB      -0.070       -0.117
## 45    Low Dizzy       0.097        0.116
## 46   Irri    WB      -0.057       -0.113
## 47   Nerv Sleep       0.109        0.112
## 48    Tol   Esc       0.098        0.108
## 49    Low  Nerv       0.073        0.107
## 50   Nerv    WB      -0.105       -0.089
## 51   Lone   Esc       0.078        0.102
## 52   Irri Sleep       0.095        0.097
## 53  Sleep Dizzy       0.097        0.052
## 54   Lone   Dis       0.000        0.092
## 55   Stom  Back       0.042        0.086
## 56   Lone   Cfl       0.000        0.085
## 57   Stom  Nerv       0.080        0.064
## 58   Back  Nerv       0.080        0.072
## 59   Nerv Dizzy       0.000        0.072
## 60   Back Sleep       0.069        0.000
## 61   Head Sleep       0.063        0.046
## 62   Head    WB      -0.063       -0.051
## 63   Head   Low       0.062        0.000
## 64   Irri   Tol       0.053        0.000
## 65   Irri  Lone       0.043        0.000
## 66   Head  Irri       0.000        0.000
## 67   Head  Nerv       0.000        0.000
## 68   Head  Lone       0.000        0.000
## 69   Head   Pre       0.000        0.000
## 70   Head   Tol       0.000        0.000
## 71   Head   Wth       0.000        0.000
## 72   Head   Prs       0.000        0.000
## 73   Head   Esc       0.000        0.000
## 74   Head   Pbl       0.000        0.000
## 75   Head   Dec       0.000        0.000
## 76   Head   Dis       0.000        0.000
## 77   Head   Cfl       0.000        0.000
## 78   Stom   Low       0.000        0.000
## 79   Stom  Irri       0.000        0.000
## 80   Stom Sleep       0.000        0.000
## 81   Stom  Lone       0.000        0.000
## 82   Stom    WB       0.000        0.000
## 83   Stom   Pre       0.000        0.000
## 84   Stom   Tol       0.000        0.000
## 85   Stom   Wth       0.000        0.000
## 86   Stom   Prs       0.000        0.000
## 87   Stom   Esc       0.000        0.000
## 88   Stom   Pbl       0.000        0.000
## 89   Stom   Dec       0.000        0.000
## 90   Stom   Dis       0.000        0.000
## 91   Stom   Cfl       0.000        0.000
## 92   Back   Low       0.000        0.000
## 93   Back  Irri       0.000        0.000
## 94   Back  Lone       0.000        0.000
## 95   Back    WB       0.000        0.000
## 96   Back   Pre       0.000        0.000
## 97   Back   Tol       0.000        0.000
## 98   Back   Wth       0.000        0.000
## 99   Back   Prs       0.000        0.000
## 100  Back   Esc       0.000        0.000
## 101  Back   Pbl       0.000        0.000
## 102  Back   Dec       0.000        0.000
## 103  Back   Dis       0.000        0.000
## 104  Back   Cfl       0.000        0.000
## 105   Low Sleep       0.000        0.000
## 106   Low   Pre       0.000        0.000
## 107   Low   Tol       0.000        0.000
## 108   Low   Wth       0.000        0.000
## 109   Low   Prs       0.000        0.000
## 110   Low   Esc       0.000        0.000
## 111   Low   Pbl       0.000        0.000
## 112   Low   Dec       0.000        0.000
## 113   Low   Dis       0.000        0.000
## 114   Low   Cfl       0.000        0.000
## 115  Irri Dizzy       0.000        0.000
## 116  Irri   Pre       0.000        0.000
## 117  Irri   Wth       0.000        0.000
## 118  Irri   Prs       0.000        0.000
## 119  Irri   Esc       0.000        0.000
## 120  Irri   Pbl       0.000        0.000
## 121  Irri   Dec       0.000        0.000
## 122  Irri   Dis       0.000        0.000
## 123  Irri   Cfl       0.000        0.000
## 124  Nerv  Lone       0.000        0.000
## 125  Nerv   Pre       0.000        0.000
## 126  Nerv   Tol       0.000        0.000
## 127  Nerv   Wth       0.000        0.000
## 128  Nerv   Prs       0.000        0.000
## 129  Nerv   Esc       0.000        0.000
## 130  Nerv   Pbl       0.000        0.000
## 131  Nerv   Dec       0.000        0.000
## 132  Nerv   Dis       0.000        0.000
## 133  Nerv   Cfl       0.000        0.000
## 134 Sleep  Lone       0.000        0.000
## 135 Sleep   Pre       0.000        0.000
## 136 Sleep   Tol       0.000        0.000
## 137 Sleep   Wth       0.000        0.000
## 138 Sleep   Prs       0.000        0.000
## 139 Sleep   Esc       0.000        0.000
## 140 Sleep   Pbl       0.000        0.000
## 141 Sleep   Dec       0.000        0.000
## 142 Sleep   Dis       0.000        0.000
## 143 Sleep   Cfl       0.000        0.000
## 144 Dizzy  Lone       0.000        0.000
## 145 Dizzy    WB       0.000        0.000
## 146 Dizzy   Pre       0.000        0.000
## 147 Dizzy   Tol       0.000        0.000
## 148 Dizzy   Wth       0.000        0.000
## 149 Dizzy   Prs       0.000        0.000
## 150 Dizzy   Esc       0.000        0.000
## 151 Dizzy   Pbl       0.000        0.000
## 152 Dizzy   Dec       0.000        0.000
## 153 Dizzy   Dis       0.000        0.000
## 154 Dizzy   Cfl       0.000        0.000
## 155  Lone   Pre       0.000        0.000
## 156  Lone   Tol       0.000        0.000
## 157  Lone   Wth       0.000        0.000
## 158  Lone   Prs       0.000        0.000
## 159  Lone   Pbl       0.000        0.000
## 160  Lone   Dec       0.000        0.000
## 161    WB   Pre       0.000        0.000
## 162    WB   Tol       0.000        0.000
## 163    WB   Wth       0.000        0.000
## 164    WB   Prs       0.000        0.000
## 165    WB   Esc       0.000        0.000
## 166    WB   Pbl       0.000        0.000
## 167    WB   Dec       0.000        0.000
## 168    WB   Dis       0.000        0.000
## 169    WB   Cfl       0.000        0.000
## 170   Tol   Dec       0.000        0.000
## 171   Tol   Cfl       0.000        0.000
```

##### SMDS

```
##      From    To Weight_Boys Weight_Girls
## 1     Pbl   Cfl       0.628        0.687
## 2     Tol   Wth       0.682        0.549
## 3     Pre   Tol       0.437        0.612
## 4     Wth   Dec       0.594        0.283
## 5     Pre   Wth       0.584        0.413
## 6     Dec   Cfl       0.522        0.568
## 7     Low  Irri       0.409        0.441
## 8     Pbl   Dis       0.416        0.269
## 9     Tol   Esc       0.383        0.276
## 10    Dec   Dis       0.380        0.304
## 11    Dis   Cfl       0.374        0.179
## 12    Prs   Esc       0.368        0.371
## 13    Tol   Pbl       0.327        0.327
## 14    Wth   Dis       0.325        0.273
## 15    Low  Lone       0.256        0.289
## 16    Esc   Pbl       0.196        0.288
## 17    Pre   Dis       0.238        0.285
## 18   Head Dizzy       0.223        0.278
## 19   Irri  Nerv       0.275        0.244
## 20    Pre   Cfl       0.272        0.056
## 21    Wth   Cfl       0.266        0.220
## 22    Esc   Dec       0.253        0.156
## 23    Pre   Esc       0.182        0.240
## 24    Prs   Pbl       0.202        0.233
## 25    Esc   Dis       0.085        0.233
## 26    Pre   Prs       0.108        0.232
## 27    Tol   Dis       0.228        0.105
## 28    Wth   Pbl       0.228        0.106
## 29    Prs   Dis       0.179        0.228
## 30    Tol   Prs       0.225        0.095
## 31    Pre   Dec       0.217        0.224
## 32    Pbl   Dec       0.197        0.216
## 33   Lone    WB      -0.209       -0.198
## 34    Pre   Pbl       0.000        0.175
## 35    Wth   Esc       0.114        0.172
## 36   Head  Stom       0.167        0.165
## 37    Esc   Cfl       0.154        0.079
## 38   Lone   Esc       0.099        0.138
## 39     WB   Esc       0.000       -0.132
## 40    Low    WB      -0.116       -0.128
## 41   Stom Dizzy       0.066        0.126
## 42   Head  Back       0.094        0.123
## 43    Tol   Cfl       0.113        0.121
## 44   Nerv Sleep       0.120        0.107
## 45   Back Dizzy       0.119        0.096
## 46    Wth   Prs       0.118        0.000
## 47    Low Dizzy       0.095        0.116
## 48   Irri    WB      -0.057       -0.111
## 49   Nerv    WB      -0.111       -0.081
## 50  Sleep    WB      -0.079       -0.110
## 51    Low  Nerv       0.072        0.105
## 52  Sleep Dizzy       0.103        0.051
## 53   Irri Sleep       0.101        0.096
## 54    Tol   Dec       0.000        0.092
## 55    Prs   Cfl       0.091        0.091
## 56   Stom  Nerv       0.088        0.063
## 57   Stom  Back       0.038        0.087
## 58   Lone   Cfl       0.000        0.087
## 59   Back  Nerv       0.086        0.069
## 60    Prs   Dec       0.000        0.085
## 61   Back Sleep       0.075        0.000
## 62   Nerv   Esc       0.050        0.073
## 63   Nerv Dizzy       0.000        0.071
## 64   Head Sleep       0.064        0.046
## 65   Head   Low       0.059        0.000
## 66   Head    WB      -0.058       -0.051
## 67   Lone   Pre       0.055        0.000
## 68   Irri  Lone       0.049        0.000
## 69  Sleep   Dec       0.000        0.048
## 70   Back   Cfl       0.000        0.042
## 71   Stom Sleep       0.039        0.000
## 72   Head  Irri       0.000        0.000
## 73   Head  Nerv       0.000        0.000
## 74   Head  Lone       0.000        0.000
## 75   Head   Pre       0.000        0.000
## 76   Head   Tol       0.000        0.000
## 77   Head   Wth       0.000        0.000
## 78   Head   Prs       0.000        0.000
## 79   Head   Esc       0.000        0.000
## 80   Head   Pbl       0.000        0.000
## 81   Head   Dec       0.000        0.000
## 82   Head   Dis       0.000        0.000
## 83   Head   Cfl       0.000        0.000
## 84   Stom   Low       0.000        0.000
## 85   Stom  Irri       0.000        0.000
## 86   Stom  Lone       0.000        0.000
## 87   Stom    WB       0.000        0.000
## 88   Stom   Pre       0.000        0.000
## 89   Stom   Tol       0.000        0.000
## 90   Stom   Wth       0.000        0.000
## 91   Stom   Prs       0.000        0.000
## 92   Stom   Esc       0.000        0.000
## 93   Stom   Pbl       0.000        0.000
## 94   Stom   Dec       0.000        0.000
## 95   Stom   Dis       0.000        0.000
## 96   Stom   Cfl       0.000        0.000
## 97   Back   Low       0.000        0.000
## 98   Back  Irri       0.000        0.000
## 99   Back  Lone       0.000        0.000
## 100  Back    WB       0.000        0.000
## 101  Back   Pre       0.000        0.000
## 102  Back   Tol       0.000        0.000
## 103  Back   Wth       0.000        0.000
## 104  Back   Prs       0.000        0.000
## 105  Back   Esc       0.000        0.000
## 106  Back   Pbl       0.000        0.000
## 107  Back   Dec       0.000        0.000
## 108  Back   Dis       0.000        0.000
## 109   Low Sleep       0.000        0.000
## 110   Low   Pre       0.000        0.000
## 111   Low   Tol       0.000        0.000
## 112   Low   Wth       0.000        0.000
## 113   Low   Prs       0.000        0.000
## 114   Low   Esc       0.000        0.000
## 115   Low   Pbl       0.000        0.000
## 116   Low   Dec       0.000        0.000
## 117   Low   Dis       0.000        0.000
## 118   Low   Cfl       0.000        0.000
## 119  Irri Dizzy       0.000        0.000
## 120  Irri   Pre       0.000        0.000
## 121  Irri   Tol       0.000        0.000
## 122  Irri   Wth       0.000        0.000
## 123  Irri   Prs       0.000        0.000
## 124  Irri   Esc       0.000        0.000
## 125  Irri   Pbl       0.000        0.000
## 126  Irri   Dec       0.000        0.000
## 127  Irri   Dis       0.000        0.000
## 128  Irri   Cfl       0.000        0.000
## 129  Nerv  Lone       0.000        0.000
## 130  Nerv   Pre       0.000        0.000
## 131  Nerv   Tol       0.000        0.000
## 132  Nerv   Wth       0.000        0.000
## 133  Nerv   Prs       0.000        0.000
## 134  Nerv   Pbl       0.000        0.000
## 135  Nerv   Dec       0.000        0.000
## 136  Nerv   Dis       0.000        0.000
## 137  Nerv   Cfl       0.000        0.000
## 138 Sleep  Lone       0.000        0.000
## 139 Sleep   Pre       0.000        0.000
## 140 Sleep   Tol       0.000        0.000
## 141 Sleep   Wth       0.000        0.000
## 142 Sleep   Prs       0.000        0.000
## 143 Sleep   Esc       0.000        0.000
## 144 Sleep   Pbl       0.000        0.000
## 145 Sleep   Dis       0.000        0.000
## 146 Sleep   Cfl       0.000        0.000
## 147 Dizzy  Lone       0.000        0.000
## 148 Dizzy    WB       0.000        0.000
## 149 Dizzy   Pre       0.000        0.000
## 150 Dizzy   Tol       0.000        0.000
## 151 Dizzy   Wth       0.000        0.000
## 152 Dizzy   Prs       0.000        0.000
## 153 Dizzy   Esc       0.000        0.000
## 154 Dizzy   Pbl       0.000        0.000
## 155 Dizzy   Dec       0.000        0.000
## 156 Dizzy   Dis       0.000        0.000
## 157 Dizzy   Cfl       0.000        0.000
## 158  Lone   Tol       0.000        0.000
## 159  Lone   Wth       0.000        0.000
## 160  Lone   Prs       0.000        0.000
## 161  Lone   Pbl       0.000        0.000
## 162  Lone   Dec       0.000        0.000
## 163  Lone   Dis       0.000        0.000
## 164    WB   Pre       0.000        0.000
## 165    WB   Tol       0.000        0.000
## 166    WB   Wth       0.000        0.000
## 167    WB   Prs       0.000        0.000
## 168    WB   Pbl       0.000        0.000
## 169    WB   Dec       0.000        0.000
## 170    WB   Dis       0.000        0.000
## 171    WB   Cfl       0.000        0.000
```

#### Centrality

##### IGDS

**The centrality estimates are relatively stable when the
correlation stability coefficient (CS coefficient) is ≥ 0.5**
while set at correlation stability = 0.7 (Epskamp et al.,
2018). Centrality indices with CS coefficient of 0.25-0.5 should be
interpreted with caution; CS coefficient < 0.25 indicated large
instability of the centrality index.

```
##   region scale group        centrality     CS_r07
## 1     SI  IGDS  Boys          strength 0.75011116
## 2     SI  IGDS  Boys         closeness 0.04979991
## 3     SI  IGDS  Boys       betweenness 0.00000000
## 4     SI  IGDS  Boys expectedInfluence 0.75011116
## 5     SI  IGDS Girls          strength 0.75000000
## 6     SI  IGDS Girls         closeness 0.00000000
## 7     SI  IGDS Girls       betweenness 0.00000000
## 8     SI  IGDS Girls expectedInfluence 0.75000000
```

##### SMDS

**The centrality estimates are relatively stable when the
correlation stability coefficient (CS coefficient) is ≥ 0.5**
while set at correlation stability = 0.7 (Epskamp et al.,
2018). Centrality indices with CS coefficient of 0.25-0.5 should be
interpreted with caution; CS coefficient < 0.25 indicated large
instability of the centrality index.

```
##    region scale group        centrality     CS_r07
## 9      SI  SMDS  Boys          strength 0.75011116
## 10     SI  SMDS  Boys         closeness 0.20542463
## 11     SI  SMDS  Boys       betweenness 0.04979991
## 12     SI  SMDS  Boys expectedInfluence 0.75011116
## 13     SI  SMDS Girls          strength 0.75000000
## 14     SI  SMDS Girls         closeness 0.51681128
## 15     SI  SMDS Girls       betweenness 0.12798265
## 16     SI  SMDS Girls expectedInfluence 0.75000000
```

#### Bootstrapped Edges

##### IGDS

###### Boys

###### Girls

##### SMDS

###### Boys

###### Girls

## Figure 4

### Overall

#### Figure

#### Edges

```
##       From     To Weight_Boys Weight_Girls
## 1     gPbl   gCfl       0.513        0.743
## 2     gEsc   sEsc       0.699        0.661
## 3     gCfl   sCfl       0.648        0.648
## 4     gTol   gWth       0.569        0.639
## 5     sTol   sWth       0.535        0.613
## 6     gDis   sDis       0.535        0.612
## 7     gDec   sDec       0.605        0.561
## 8     sDec   sCfl       0.605        0.472
## 9     gPre   gTol       0.479        0.597
## 10    sPre   sTol       0.474        0.587
## 11    sPbl   sCfl       0.493        0.574
## 12    gPre   gWth       0.403        0.509
## 13    sPre   sWth       0.452        0.419
## 14    gPre   gEsc       0.394        0.434
## 15    gPbl   gDis       0.346        0.434
## 16    gDec   gCfl       0.420        0.343
## 17    gPbl   sPbl       0.390        0.326
## 18    gDis   gCfl       0.389        0.284
## 19    gPbl   gDec       0.211        0.373
## 20    gPrs   sPrs       0.322        0.367
## 21    gTol   sTol       0.265        0.363
## 22    gWth   sWth       0.360        0.342
## 23    gDec   gDis       0.342        0.332
## 24    gTol   gEsc       0.137        0.341
## 25    gPrs   gDec       0.252        0.340
## 26    gPrs   gDis       0.330        0.340
## 27    sPrs   sEsc       0.330        0.318
## 28    gPre   gPrs       0.173        0.323
## 29    gEsc   gDec       0.223        0.323
## 30    sPbl   sDec       0.187        0.323
## 31    gTol   gDis       0.247        0.319
## 32    gPrs   gPbl       0.243        0.319
## 33    sEsc   sPbl       0.316        0.222
## 34    sDec   sDis       0.315        0.190
## 35    sDis   sCfl       0.299        0.137
## 36    sTol   sPbl       0.288        0.224
## 37    gTol   gPbl       0.287        0.234
## 38    gWth   gCfl       0.285        0.269
## 39    sTol   sEsc       0.283        0.174
## 40    sPrs   sDis       0.177        0.278
## 41    gWth   gDec       0.258        0.273
## 42    sWth   sDec       0.269        0.172
## 43      WB   lone      -0.238       -0.265
## 44   pIrri  pNerv       0.258        0.260
## 45    gWth   gEsc       0.185        0.253
## 46    sPrs   sDec       0.253        0.174
## 47    gWth   gDis       0.234        0.251
## 48    pLow   lone       0.251        0.237
## 49    gTol   gPrs       0.248        0.220
## 50    gPrs   gEsc       0.147        0.243
## 51    gPre   gDis       0.129        0.241
## 52   pHead pDizzy       0.198        0.241
## 53    pLow  pIrri       0.234        0.241
## 54    sPre   sDis       0.239        0.163
## 55    sWth   sCfl       0.238        0.171
## 56    gEsc   gPbl       0.166        0.228
## 57    sWth   sEsc       0.228        0.209
## 58    sPbl   sDis       0.227        0.194
## 59    gPre   sPre       0.179        0.225
## 60   pHead  pStom       0.225        0.193
## 61    sPre   sEsc       0.161        0.224
## 62    sPrs   sPbl       0.216        0.180
## 63    gPre   gDec       0.168        0.214
## 64    gPre   gPbl       0.210        0.208
## 65    gEsc   gCfl       0.180        0.209
## 66    gPrs   gCfl       0.206        0.190
## 67    sPre   sPrs       0.186        0.206
## 68    sWth   sPbl       0.206        0.169
## 69    gPre   gCfl       0.126        0.205
## 70    sWth   sDis       0.205        0.203
## 71    sPre   sCfl       0.199        0.102
## 72    sTol   sPrs       0.198        0.136
## 73   pStom pDizzy       0.146        0.184
## 74    sPre   sDec       0.178        0.152
## 75    sEsc   sDec       0.104        0.173
## 76    sEsc   sDis       0.098        0.172
## 77    pLow  pNerv       0.151        0.170
## 78    gCfl   sPbl       0.121        0.165
## 79    gWth   gPrs       0.147        0.159
## 80    sTol   sCfl       0.157        0.142
## 81    gPbl   sCfl       0.150        0.156
## 82    pLow     WB      -0.119       -0.156
## 83  pSleep pDizzy       0.132        0.152
## 84    sEsc   sCfl       0.149        0.112
## 85   pHead  pBack       0.122        0.146
## 86    gTol   sWth       0.141        0.122
## 87    gTol   gDec       0.136        0.134
## 88  pSleep     WB      -0.123       -0.120
## 89    sEsc   lone       0.121        0.110
## 90    gWth   sTol       0.114        0.039
## 91    sEsc     WB       0.000       -0.114
## 92   pIrri     WB      -0.114       -0.108
## 93    gWth   gPbl       0.110        0.113
## 94    gEsc   gDis       0.000        0.112
## 95    pLow pDizzy       0.084        0.112
## 96    gPbl   sDec       0.111        0.084
## 97    sPre   sPbl       0.086        0.111
## 98    sTol   sDis       0.111        0.073
## 99    sPrs   sCfl       0.105        0.102
## 100   sEsc  pNerv       0.074        0.104
## 101   gEsc   lone       0.075        0.097
## 102   sEsc   pLow       0.088        0.097
## 103  pStom  pBack       0.088        0.094
## 104   gDec   sPbl       0.091        0.000
## 105   sTol   sDec       0.063        0.091
## 106  pHead pSleep       0.088        0.063
## 107  pHead   pLow       0.077        0.087
## 108   gDec pSleep       0.000        0.086
## 109   gTol   gCfl       0.085        0.000
## 110  pNerv pSleep       0.066        0.084
## 111  pBack pDizzy       0.083        0.083
## 112   gEsc   sCfl      -0.079        0.000
## 113   gDis     WB       0.000       -0.079
## 114   gPbl  pIrri       0.077        0.000
## 115   gPrs   sDis       0.075        0.031
## 116   gEsc   sPrs       0.000        0.072
## 117  pNerv   lone       0.064        0.071
## 118   gDis   sPre       0.053        0.070
## 119  pIrri pSleep       0.067        0.068
## 120  pNerv     WB      -0.066       -0.060
## 121  pStom   pLow       0.048        0.065
## 122  pBack  pIrri       0.063        0.045
## 123  pHead  pNerv       0.062        0.055
## 124  pBack   pLow       0.062        0.044
## 125   gEsc pSleep       0.060        0.054
## 126  pBack pSleep       0.060        0.034
## 127  pStom  pNerv       0.051        0.059
## 128  pStom pSleep       0.059        0.039
## 129   gPre   sTol       0.057        0.000
## 130   gWth   sPre       0.056        0.000
## 131   gPre   sPrs       0.000       -0.055
## 132   gPrs   sPbl       0.053        0.000
## 133   gEsc     WB      -0.050        0.000
## 134   sPbl  pIrri       0.029        0.050
## 135 pSleep   lone       0.050        0.047
## 136   sWth   sPrs       0.044        0.049
## 137  pBack  pNerv       0.045        0.049
## 138   pLow pSleep       0.047        0.049
## 139   gCfl pDizzy       0.048        0.000
## 140   sCfl  pIrri       0.000        0.048
## 141  pNerv pDizzy       0.037        0.048
## 142   gDis   sPbl       0.000        0.046
## 143   gDis   sTol       0.038        0.045
## 144  pHead  pIrri       0.042        0.042
## 145  pIrri   lone       0.040        0.041
## 146   gTol     WB      -0.040        0.000
## 147   gPrs   sTol       0.039        0.000
## 148  pStom  pIrri       0.038        0.000
## 149   gEsc   pLow       0.037        0.000
## 150   sCfl     WB       0.000       -0.037
## 151   sPbl  pNerv       0.000        0.036
## 152   sDis     WB      -0.035        0.000
## 153   gWth   sDec       0.034        0.000
## 154   gEsc  pNerv       0.034        0.000
## 155   sCfl   lone       0.000        0.034
## 156   sPre     WB       0.033        0.000
## 157   sTol pDizzy       0.000        0.033
## 158   sDec pDizzy       0.000        0.033
## 159   sDec  pBack       0.032        0.000
## 160   sWth  pIrri       0.000        0.030
## 161   sDec  pIrri       0.000        0.030
## 162   sEsc pDizzy       0.000        0.029
## 163   sDec     WB       0.000       -0.029
## 164   gPre  pNerv       0.028        0.000
## 165   gWth   sDis       0.028        0.000
## 166   gEsc pDizzy       0.000       -0.028
## 167   sDec pSleep       0.000        0.028
## 168   sPre  pIrri       0.000        0.027
## 169   gTol pDizzy       0.000        0.026
## 170   gWth  pIrri       0.025        0.000
## 171  pIrri pDizzy       0.025        0.000
## 172   gDis   sDec       0.024        0.000
## 173   sPbl pDizzy       0.000        0.024
## 174   gPre     WB      -0.023        0.000
## 175 pDizzy   lone       0.023        0.000
## 176   sWth  pStom       0.000        0.022
## 177   sPre  pBack       0.000        0.018
## 178   sEsc  pIrri       0.000        0.018
## 179  pBack     WB      -0.018        0.000
## 180  pHead     WB       0.000       -0.017
## 181   gPre   sWth       0.000        0.000
## 182   gPre   sEsc       0.000        0.000
## 183   gPre   sPbl       0.000        0.000
## 184   gPre   sDec       0.000        0.000
## 185   gPre   sDis       0.000        0.000
## 186   gPre   sCfl       0.000        0.000
## 187   gPre  pHead       0.000        0.000
## 188   gPre  pStom       0.000        0.000
## 189   gPre  pBack       0.000        0.000
## 190   gPre   pLow       0.000        0.000
## 191   gPre  pIrri       0.000        0.000
## 192   gPre pSleep       0.000        0.000
## 193   gPre pDizzy       0.000        0.000
## 194   gPre   lone       0.000        0.000
## 195   gTol   sPre       0.000        0.000
## 196   gTol   sPrs       0.000        0.000
## 197   gTol   sEsc       0.000        0.000
## 198   gTol   sPbl       0.000        0.000
## 199   gTol   sDec       0.000        0.000
## 200   gTol   sDis       0.000        0.000
## 201   gTol   sCfl       0.000        0.000
## 202   gTol  pHead       0.000        0.000
## 203   gTol  pStom       0.000        0.000
## 204   gTol  pBack       0.000        0.000
## 205   gTol   pLow       0.000        0.000
## 206   gTol  pIrri       0.000        0.000
## 207   gTol  pNerv       0.000        0.000
## 208   gTol pSleep       0.000        0.000
## 209   gTol   lone       0.000        0.000
## 210   gWth   sPrs       0.000        0.000
## 211   gWth   sEsc       0.000        0.000
## 212   gWth   sPbl       0.000        0.000
## 213   gWth   sCfl       0.000        0.000
## 214   gWth  pHead       0.000        0.000
## 215   gWth  pStom       0.000        0.000
## 216   gWth  pBack       0.000        0.000
## 217   gWth   pLow       0.000        0.000
## 218   gWth  pNerv       0.000        0.000
## 219   gWth pSleep       0.000        0.000
## 220   gWth pDizzy       0.000        0.000
## 221   gWth     WB       0.000        0.000
## 222   gWth   lone       0.000        0.000
## 223   gPrs   sPre       0.000        0.000
## 224   gPrs   sWth       0.000        0.000
## 225   gPrs   sEsc       0.000        0.000
## 226   gPrs   sDec       0.000        0.000
## 227   gPrs   sCfl       0.000        0.000
## 228   gPrs  pHead       0.000        0.000
## 229   gPrs  pStom       0.000        0.000
## 230   gPrs  pBack       0.000        0.000
## 231   gPrs   pLow       0.000        0.000
## 232   gPrs  pIrri       0.000        0.000
## 233   gPrs  pNerv       0.000        0.000
## 234   gPrs pSleep       0.000        0.000
## 235   gPrs pDizzy       0.000        0.000
## 236   gPrs     WB       0.000        0.000
## 237   gPrs   lone       0.000        0.000
## 238   gEsc   sPre       0.000        0.000
## 239   gEsc   sTol       0.000        0.000
## 240   gEsc   sWth       0.000        0.000
## 241   gEsc   sPbl       0.000        0.000
## 242   gEsc   sDec       0.000        0.000
## 243   gEsc   sDis       0.000        0.000
## 244   gEsc  pHead       0.000        0.000
## 245   gEsc  pStom       0.000        0.000
## 246   gEsc  pBack       0.000        0.000
## 247   gEsc  pIrri       0.000        0.000
## 248   gPbl   sPre       0.000        0.000
## 249   gPbl   sTol       0.000        0.000
## 250   gPbl   sWth       0.000        0.000
## 251   gPbl   sPrs       0.000        0.000
## 252   gPbl   sEsc       0.000        0.000
## 253   gPbl   sDis       0.000        0.000
## 254   gPbl  pHead       0.000        0.000
## 255   gPbl  pStom       0.000        0.000
## 256   gPbl  pBack       0.000        0.000
## 257   gPbl   pLow       0.000        0.000
## 258   gPbl  pNerv       0.000        0.000
## 259   gPbl pSleep       0.000        0.000
## 260   gPbl pDizzy       0.000        0.000
## 261   gPbl     WB       0.000        0.000
## 262   gPbl   lone       0.000        0.000
## 263   gDec   sPre       0.000        0.000
## 264   gDec   sTol       0.000        0.000
## 265   gDec   sWth       0.000        0.000
## 266   gDec   sPrs       0.000        0.000
## 267   gDec   sEsc       0.000        0.000
## 268   gDec   sDis       0.000        0.000
## 269   gDec   sCfl       0.000        0.000
## 270   gDec  pHead       0.000        0.000
## 271   gDec  pStom       0.000        0.000
## 272   gDec  pBack       0.000        0.000
## 273   gDec   pLow       0.000        0.000
## 274   gDec  pIrri       0.000        0.000
## 275   gDec  pNerv       0.000        0.000
## 276   gDec pDizzy       0.000        0.000
## 277   gDec     WB       0.000        0.000
## 278   gDec   lone       0.000        0.000
## 279   gDis   sWth       0.000        0.000
## 280   gDis   sPrs       0.000        0.000
## 281   gDis   sEsc       0.000        0.000
## 282   gDis   sCfl       0.000        0.000
## 283   gDis  pHead       0.000        0.000
## 284   gDis  pStom       0.000        0.000
## 285   gDis  pBack       0.000        0.000
## 286   gDis   pLow       0.000        0.000
## 287   gDis  pIrri       0.000        0.000
## 288   gDis  pNerv       0.000        0.000
## 289   gDis pSleep       0.000        0.000
## 290   gDis pDizzy       0.000        0.000
## 291   gDis   lone       0.000        0.000
## 292   gCfl   sPre       0.000        0.000
## 293   gCfl   sTol       0.000        0.000
## 294   gCfl   sWth       0.000        0.000
## 295   gCfl   sPrs       0.000        0.000
## 296   gCfl   sEsc       0.000        0.000
## 297   gCfl   sDec       0.000        0.000
## 298   gCfl   sDis       0.000        0.000
## 299   gCfl  pHead       0.000        0.000
## 300   gCfl  pStom       0.000        0.000
## 301   gCfl  pBack       0.000        0.000
## 302   gCfl   pLow       0.000        0.000
## 303   gCfl  pIrri       0.000        0.000
## 304   gCfl  pNerv       0.000        0.000
## 305   gCfl pSleep       0.000        0.000
## 306   gCfl     WB       0.000        0.000
## 307   gCfl   lone       0.000        0.000
## 308   sPre  pHead       0.000        0.000
## 309   sPre  pStom       0.000        0.000
## 310   sPre   pLow       0.000        0.000
## 311   sPre  pNerv       0.000        0.000
## 312   sPre pSleep       0.000        0.000
## 313   sPre pDizzy       0.000        0.000
## 314   sPre   lone       0.000        0.000
## 315   sTol  pHead       0.000        0.000
## 316   sTol  pStom       0.000        0.000
## 317   sTol  pBack       0.000        0.000
## 318   sTol   pLow       0.000        0.000
## 319   sTol  pIrri       0.000        0.000
## 320   sTol  pNerv       0.000        0.000
## 321   sTol pSleep       0.000        0.000
## 322   sTol     WB       0.000        0.000
## 323   sTol   lone       0.000        0.000
## 324   sWth  pHead       0.000        0.000
## 325   sWth  pBack       0.000        0.000
## 326   sWth   pLow       0.000        0.000
## 327   sWth  pNerv       0.000        0.000
## 328   sWth pSleep       0.000        0.000
## 329   sWth pDizzy       0.000        0.000
## 330   sWth     WB       0.000        0.000
## 331   sWth   lone       0.000        0.000
## 332   sPrs  pHead       0.000        0.000
## 333   sPrs  pStom       0.000        0.000
## 334   sPrs  pBack       0.000        0.000
## 335   sPrs   pLow       0.000        0.000
## 336   sPrs  pIrri       0.000        0.000
## 337   sPrs  pNerv       0.000        0.000
## 338   sPrs pSleep       0.000        0.000
## 339   sPrs pDizzy       0.000        0.000
## 340   sPrs     WB       0.000        0.000
## 341   sPrs   lone       0.000        0.000
## 342   sEsc  pHead       0.000        0.000
## 343   sEsc  pStom       0.000        0.000
## 344   sEsc  pBack       0.000        0.000
## 345   sEsc pSleep       0.000        0.000
## 346   sPbl  pHead       0.000        0.000
## 347   sPbl  pStom       0.000        0.000
## 348   sPbl  pBack       0.000        0.000
## 349   sPbl   pLow       0.000        0.000
## 350   sPbl pSleep       0.000        0.000
## 351   sPbl     WB       0.000        0.000
## 352   sPbl   lone       0.000        0.000
## 353   sDec  pHead       0.000        0.000
## 354   sDec  pStom       0.000        0.000
## 355   sDec   pLow       0.000        0.000
## 356   sDec  pNerv       0.000        0.000
## 357   sDec   lone       0.000        0.000
## 358   sDis  pHead       0.000        0.000
## 359   sDis  pStom       0.000        0.000
## 360   sDis  pBack       0.000        0.000
## 361   sDis   pLow       0.000        0.000
## 362   sDis  pIrri       0.000        0.000
## 363   sDis  pNerv       0.000        0.000
## 364   sDis pSleep       0.000        0.000
## 365   sDis pDizzy       0.000        0.000
## 366   sDis   lone       0.000        0.000
## 367   sCfl  pHead       0.000        0.000
## 368   sCfl  pStom       0.000        0.000
## 369   sCfl  pBack       0.000        0.000
## 370   sCfl   pLow       0.000        0.000
## 371   sCfl  pNerv       0.000        0.000
## 372   sCfl pSleep       0.000        0.000
## 373   sCfl pDizzy       0.000        0.000
## 374  pHead   lone       0.000        0.000
## 375  pStom     WB       0.000        0.000
## 376  pStom   lone       0.000        0.000
## 377  pBack   lone       0.000        0.000
## 378 pDizzy     WB       0.000        0.000
```

#### Centrality

**The centrality estimates are relatively stable when the
correlation stability coefficient (CS coefficient) is ≥ 0.5**
while set at correlation stability = 0.7 (Epskamp et al.,
2018). Centrality indices with CS coefficient of 0.25-0.5 should be
interpreted with caution; CS coefficient < 0.25 indicated large
instability of the centrality index.

```
##   group        centrality     CS_r07
## 1  Boys          strength 0.67217788
## 2  Boys         closeness 0.28335234
## 3  Boys       betweenness 0.05017104
## 4  Boys expectedInfluence 0.75028506
## 5 Girls          strength 0.75000000
## 6 Girls         closeness 0.59451220
## 7 Girls       betweenness 0.51676829
## 8 Girls expectedInfluence 0.75000000
```

#### Bootstrapped Edges

### CY

#### Figure

#### Edges

```
##       From     To Weight_Boys Weight_Girls
## 1     sDec   sCfl       0.713        0.460
## 2     gTol   gWth       0.675        0.635
## 3     gEsc   sEsc       0.561        0.513
## 4     gCfl   sCfl       0.251        0.531
## 5    pIrri  pNerv       0.513        0.493
## 6     gPre   gWth       0.300        0.504
## 7     gDec   gCfl       0.471        0.112
## 8     gPbl   gDis       0.076        0.468
## 9     gDec   sDec       0.240        0.467
## 10    sPbl   sCfl       0.358        0.454
## 11    sTol   sWth       0.452        0.426
## 12    gDis   gCfl       0.447        0.336
## 13    sPre   sWth       0.246        0.431
## 14    gPbl   gCfl       0.392        0.430
## 15    gPre   gEsc       0.422        0.319
## 16    gTol   sTol       0.119        0.417
## 17    gDec   gDis       0.291        0.409
## 18    gPrs   gDis       0.406        0.331
## 19    sPre   sTol       0.356        0.391
## 20    gPre   gTol       0.385        0.358
## 21    gPrs   sPrs       0.220        0.384
## 22    gTol   gEsc       0.151        0.379
## 23    gPrs   gDec       0.153        0.367
## 24    gPrs   gPbl       0.348        0.359
## 25    sTol   sEsc       0.356        0.164
## 26    gTol   gPbl       0.355        0.320
## 27    gDis   sDis       0.349        0.291
## 28      WB   lone      -0.239       -0.325
## 29    gPre   gPrs       0.120        0.314
## 30   pHead pDizzy       0.310        0.303
## 31    gPbl   gDec       0.289        0.307
## 32    sWth   sPbl       0.295        0.255
## 33    gWth   sWth       0.167        0.287
## 34    sWth   sDec       0.283        0.183
## 35    gPrs   gEsc       0.164        0.282
## 36    sPre   sEsc       0.237        0.282
## 37    gWth   gCfl       0.262        0.280
## 38    gWth   gDec       0.274        0.149
## 39    gTol   gDis       0.273        0.144
## 40    gPre   gPbl       0.259        0.250
## 41    gWth   gPrs       0.256        0.236
## 42    sPrs   sEsc       0.254        0.202
## 43    gEsc   gCfl       0.198        0.252
## 44    gPbl   sCfl       0.184        0.243
## 45    gEsc   gDec       0.232        0.195
## 46    sPrs   sCfl       0.000        0.218
## 47    sPbl   sDis       0.218        0.189
## 48    gTol   gDec       0.087        0.215
## 49    pLow     WB      -0.201       -0.212
## 50    gWth   gEsc       0.089        0.210
## 51    sEsc   sPbl       0.210        0.156
## 52    sEsc     WB       0.000       -0.208
## 53    gPre   gCfl       0.123        0.206
## 54    sPre   sDis       0.205        0.192
## 55   pHead  pStom       0.201        0.178
## 56    gWth   sTol       0.200        0.000
## 57    sDec   sDis       0.101        0.200
## 58    gPre   gDec       0.087        0.198
## 59    gPrs   gCfl       0.141        0.197
## 60    sPre   sPrs       0.193        0.132
## 61    sWth   sEsc       0.191        0.110
## 62    gTol   gPrs       0.119        0.188
## 63    sTol   sCfl       0.186        0.160
## 64    gPre   sPre       0.184        0.000
## 65    sPrs   sDec       0.184        0.055
## 66    sEsc   sCfl       0.181        0.137
## 67    gPbl   sPbl       0.174        0.149
## 68    sTol   sPbl       0.174        0.162
## 69    sWth   sDis       0.173        0.151
## 70  pSleep pDizzy       0.113        0.166
## 71    sPrs   sPbl       0.144        0.162
## 72    sPre   sCfl       0.148        0.079
## 73    sPre   sDec       0.147        0.106
## 74    sDis   sCfl       0.145        0.098
## 75    sWth   sPrs       0.000        0.144
## 76    sPbl   sDec       0.000        0.143
## 77    gCfl   sPbl       0.000        0.142
## 78    sWth   sCfl       0.000        0.142
## 79    sPrs   sDis       0.118        0.142
## 80    gCfl   sDec       0.000        0.134
## 81    gTol   sWth       0.133        0.000
## 82    pLow  pIrri       0.133        0.122
## 83   pStom pDizzy       0.130        0.105
## 84    gEsc   gPbl       0.000        0.129
## 85    pLow  pNerv       0.000        0.125
## 86    sPre   sPbl       0.124        0.101
## 87    sEsc   sDec       0.000        0.123
## 88    gPrs   sCfl       0.121        0.000
## 89   pHead  pBack       0.097        0.118
## 90    sEsc   lone       0.115        0.000
## 91   pStom  pBack       0.000        0.115
## 92    pLow   lone       0.063        0.109
## 93    pLow pSleep       0.000        0.103
## 94    gPbl   sDec       0.100        0.096
## 95   pNerv     WB      -0.082       -0.098
## 96  pSleep   lone       0.096        0.083
## 97    pLow pDizzy       0.000        0.094
## 98    sTol   sDis       0.093        0.000
## 99    gEsc   sWth       0.091        0.000
## 100  pHead   pLow       0.091        0.086
## 101   gTol   gCfl       0.089        0.000
## 102   gWth   gPbl       0.000        0.086
## 103  pBack   pLow       0.086        0.000
## 104 pSleep     WB      -0.086        0.000
## 105   gEsc   lone       0.084        0.000
## 106   gDec   sPbl       0.082        0.000
## 107   sTol   sDec       0.082        0.066
## 108   sCfl  pIrri       0.000        0.079
## 109  pBack pDizzy       0.000        0.076
## 110   gEsc   sDis       0.070        0.000
## 111  pHead     WB       0.000       -0.070
## 112  pNerv pSleep       0.000        0.070
## 113  pStom   pLow       0.067        0.057
## 114   gWth   gDis       0.000        0.066
## 115  pBack pSleep       0.049        0.056
## 116   gPre   gDis       0.000        0.000
## 117   gPre   sTol       0.000        0.000
## 118   gPre   sWth       0.000        0.000
## 119   gPre   sPrs       0.000        0.000
## 120   gPre   sEsc       0.000        0.000
## 121   gPre   sPbl       0.000        0.000
## 122   gPre   sDec       0.000        0.000
## 123   gPre   sDis       0.000        0.000
## 124   gPre   sCfl       0.000        0.000
## 125   gPre  pHead       0.000        0.000
## 126   gPre  pStom       0.000        0.000
## 127   gPre  pBack       0.000        0.000
## 128   gPre   pLow       0.000        0.000
## 129   gPre  pIrri       0.000        0.000
## 130   gPre  pNerv       0.000        0.000
## 131   gPre pSleep       0.000        0.000
## 132   gPre pDizzy       0.000        0.000
## 133   gPre     WB       0.000        0.000
## 134   gPre   lone       0.000        0.000
## 135   gTol   sPre       0.000        0.000
## 136   gTol   sPrs       0.000        0.000
## 137   gTol   sEsc       0.000        0.000
## 138   gTol   sPbl       0.000        0.000
## 139   gTol   sDec       0.000        0.000
## 140   gTol   sDis       0.000        0.000
## 141   gTol   sCfl       0.000        0.000
## 142   gTol  pHead       0.000        0.000
## 143   gTol  pStom       0.000        0.000
## 144   gTol  pBack       0.000        0.000
## 145   gTol   pLow       0.000        0.000
## 146   gTol  pIrri       0.000        0.000
## 147   gTol  pNerv       0.000        0.000
## 148   gTol pSleep       0.000        0.000
## 149   gTol pDizzy       0.000        0.000
## 150   gTol     WB       0.000        0.000
## 151   gTol   lone       0.000        0.000
## 152   gWth   sPre       0.000        0.000
## 153   gWth   sPrs       0.000        0.000
## 154   gWth   sEsc       0.000        0.000
## 155   gWth   sPbl       0.000        0.000
## 156   gWth   sDec       0.000        0.000
## 157   gWth   sDis       0.000        0.000
## 158   gWth   sCfl       0.000        0.000
## 159   gWth  pHead       0.000        0.000
## 160   gWth  pStom       0.000        0.000
## 161   gWth  pBack       0.000        0.000
## 162   gWth   pLow       0.000        0.000
## 163   gWth  pIrri       0.000        0.000
## 164   gWth  pNerv       0.000        0.000
## 165   gWth pSleep       0.000        0.000
## 166   gWth pDizzy       0.000        0.000
## 167   gWth     WB       0.000        0.000
## 168   gWth   lone       0.000        0.000
## 169   gPrs   sPre       0.000        0.000
## 170   gPrs   sTol       0.000        0.000
## 171   gPrs   sWth       0.000        0.000
## 172   gPrs   sEsc       0.000        0.000
## 173   gPrs   sPbl       0.000        0.000
## 174   gPrs   sDec       0.000        0.000
## 175   gPrs   sDis       0.000        0.000
## 176   gPrs  pHead       0.000        0.000
## 177   gPrs  pStom       0.000        0.000
## 178   gPrs  pBack       0.000        0.000
## 179   gPrs   pLow       0.000        0.000
## 180   gPrs  pIrri       0.000        0.000
## 181   gPrs  pNerv       0.000        0.000
## 182   gPrs pSleep       0.000        0.000
## 183   gPrs pDizzy       0.000        0.000
## 184   gPrs     WB       0.000        0.000
## 185   gPrs   lone       0.000        0.000
## 186   gEsc   gDis       0.000        0.000
## 187   gEsc   sPre       0.000        0.000
## 188   gEsc   sTol       0.000        0.000
## 189   gEsc   sPrs       0.000        0.000
## 190   gEsc   sPbl       0.000        0.000
## 191   gEsc   sDec       0.000        0.000
## 192   gEsc   sCfl       0.000        0.000
## 193   gEsc  pHead       0.000        0.000
## 194   gEsc  pStom       0.000        0.000
## 195   gEsc  pBack       0.000        0.000
## 196   gEsc   pLow       0.000        0.000
## 197   gEsc  pIrri       0.000        0.000
## 198   gEsc  pNerv       0.000        0.000
## 199   gEsc pSleep       0.000        0.000
## 200   gEsc pDizzy       0.000        0.000
## 201   gEsc     WB       0.000        0.000
## 202   gPbl   sPre       0.000        0.000
## 203   gPbl   sTol       0.000        0.000
## 204   gPbl   sWth       0.000        0.000
## 205   gPbl   sPrs       0.000        0.000
## 206   gPbl   sEsc       0.000        0.000
## 207   gPbl   sDis       0.000        0.000
## 208   gPbl  pHead       0.000        0.000
## 209   gPbl  pStom       0.000        0.000
## 210   gPbl  pBack       0.000        0.000
## 211   gPbl   pLow       0.000        0.000
## 212   gPbl  pIrri       0.000        0.000
## 213   gPbl  pNerv       0.000        0.000
## 214   gPbl pSleep       0.000        0.000
## 215   gPbl pDizzy       0.000        0.000
## 216   gPbl     WB       0.000        0.000
## 217   gPbl   lone       0.000        0.000
## 218   gDec   sPre       0.000        0.000
## 219   gDec   sTol       0.000        0.000
## 220   gDec   sWth       0.000        0.000
## 221   gDec   sPrs       0.000        0.000
## 222   gDec   sEsc       0.000        0.000
## 223   gDec   sDis       0.000        0.000
## 224   gDec   sCfl       0.000        0.000
## 225   gDec  pHead       0.000        0.000
## 226   gDec  pStom       0.000        0.000
## 227   gDec  pBack       0.000        0.000
## 228   gDec   pLow       0.000        0.000
## 229   gDec  pIrri       0.000        0.000
## 230   gDec  pNerv       0.000        0.000
## 231   gDec pSleep       0.000        0.000
## 232   gDec pDizzy       0.000        0.000
## 233   gDec     WB       0.000        0.000
## 234   gDec   lone       0.000        0.000
## 235   gDis   sPre       0.000        0.000
## 236   gDis   sTol       0.000        0.000
## 237   gDis   sWth       0.000        0.000
## 238   gDis   sPrs       0.000        0.000
## 239   gDis   sEsc       0.000        0.000
## 240   gDis   sPbl       0.000        0.000
## 241   gDis   sDec       0.000        0.000
## 242   gDis   sCfl       0.000        0.000
## 243   gDis  pHead       0.000        0.000
## 244   gDis  pStom       0.000        0.000
## 245   gDis  pBack       0.000        0.000
## 246   gDis   pLow       0.000        0.000
## 247   gDis  pIrri       0.000        0.000
## 248   gDis  pNerv       0.000        0.000
## 249   gDis pSleep       0.000        0.000
## 250   gDis pDizzy       0.000        0.000
## 251   gDis     WB       0.000        0.000
## 252   gDis   lone       0.000        0.000
## 253   gCfl   sPre       0.000        0.000
## 254   gCfl   sTol       0.000        0.000
## 255   gCfl   sWth       0.000        0.000
## 256   gCfl   sPrs       0.000        0.000
## 257   gCfl   sEsc       0.000        0.000
## 258   gCfl   sDis       0.000        0.000
## 259   gCfl  pHead       0.000        0.000
## 260   gCfl  pStom       0.000        0.000
## 261   gCfl  pBack       0.000        0.000
## 262   gCfl   pLow       0.000        0.000
## 263   gCfl  pIrri       0.000        0.000
## 264   gCfl  pNerv       0.000        0.000
## 265   gCfl pSleep       0.000        0.000
## 266   gCfl pDizzy       0.000        0.000
## 267   gCfl     WB       0.000        0.000
## 268   gCfl   lone       0.000        0.000
## 269   sPre  pHead       0.000        0.000
## 270   sPre  pStom       0.000        0.000
## 271   sPre  pBack       0.000        0.000
## 272   sPre   pLow       0.000        0.000
## 273   sPre  pIrri       0.000        0.000
## 274   sPre  pNerv       0.000        0.000
## 275   sPre pSleep       0.000        0.000
## 276   sPre pDizzy       0.000        0.000
## 277   sPre     WB       0.000        0.000
## 278   sPre   lone       0.000        0.000
## 279   sTol   sPrs       0.000        0.000
## 280   sTol  pHead       0.000        0.000
## 281   sTol  pStom       0.000        0.000
## 282   sTol  pBack       0.000        0.000
## 283   sTol   pLow       0.000        0.000
## 284   sTol  pIrri       0.000        0.000
## 285   sTol  pNerv       0.000        0.000
## 286   sTol pSleep       0.000        0.000
## 287   sTol pDizzy       0.000        0.000
## 288   sTol     WB       0.000        0.000
## 289   sTol   lone       0.000        0.000
## 290   sWth  pHead       0.000        0.000
## 291   sWth  pStom       0.000        0.000
## 292   sWth  pBack       0.000        0.000
## 293   sWth   pLow       0.000        0.000
## 294   sWth  pIrri       0.000        0.000
## 295   sWth  pNerv       0.000        0.000
## 296   sWth pSleep       0.000        0.000
## 297   sWth pDizzy       0.000        0.000
## 298   sWth     WB       0.000        0.000
## 299   sWth   lone       0.000        0.000
## 300   sPrs  pHead       0.000        0.000
## 301   sPrs  pStom       0.000        0.000
## 302   sPrs  pBack       0.000        0.000
## 303   sPrs   pLow       0.000        0.000
## 304   sPrs  pIrri       0.000        0.000
## 305   sPrs  pNerv       0.000        0.000
## 306   sPrs pSleep       0.000        0.000
## 307   sPrs pDizzy       0.000        0.000
## 308   sPrs     WB       0.000        0.000
## 309   sPrs   lone       0.000        0.000
## 310   sEsc   sDis       0.000        0.000
## 311   sEsc  pHead       0.000        0.000
## 312   sEsc  pStom       0.000        0.000
## 313   sEsc  pBack       0.000        0.000
## 314   sEsc   pLow       0.000        0.000
## 315   sEsc  pIrri       0.000        0.000
## 316   sEsc  pNerv       0.000        0.000
## 317   sEsc pSleep       0.000        0.000
## 318   sEsc pDizzy       0.000        0.000
## 319   sPbl  pHead       0.000        0.000
## 320   sPbl  pStom       0.000        0.000
## 321   sPbl  pBack       0.000        0.000
## 322   sPbl   pLow       0.000        0.000
## 323   sPbl  pIrri       0.000        0.000
## 324   sPbl  pNerv       0.000        0.000
## 325   sPbl pSleep       0.000        0.000
## 326   sPbl pDizzy       0.000        0.000
## 327   sPbl     WB       0.000        0.000
## 328   sPbl   lone       0.000        0.000
## 329   sDec  pHead       0.000        0.000
## 330   sDec  pStom       0.000        0.000
## 331   sDec  pBack       0.000        0.000
## 332   sDec   pLow       0.000        0.000
## 333   sDec  pIrri       0.000        0.000
## 334   sDec  pNerv       0.000        0.000
## 335   sDec pSleep       0.000        0.000
## 336   sDec pDizzy       0.000        0.000
## 337   sDec     WB       0.000        0.000
## 338   sDec   lone       0.000        0.000
## 339   sDis  pHead       0.000        0.000
## 340   sDis  pStom       0.000        0.000
## 341   sDis  pBack       0.000        0.000
## 342   sDis   pLow       0.000        0.000
## 343   sDis  pIrri       0.000        0.000
## 344   sDis  pNerv       0.000        0.000
## 345   sDis pSleep       0.000        0.000
## 346   sDis pDizzy       0.000        0.000
## 347   sDis     WB       0.000        0.000
## 348   sDis   lone       0.000        0.000
## 349   sCfl  pHead       0.000        0.000
## 350   sCfl  pStom       0.000        0.000
## 351   sCfl  pBack       0.000        0.000
## 352   sCfl   pLow       0.000        0.000
## 353   sCfl  pNerv       0.000        0.000
## 354   sCfl pSleep       0.000        0.000
## 355   sCfl pDizzy       0.000        0.000
## 356   sCfl     WB       0.000        0.000
## 357   sCfl   lone       0.000        0.000
## 358  pHead  pIrri       0.000        0.000
## 359  pHead  pNerv       0.000        0.000
## 360  pHead pSleep       0.000        0.000
## 361  pHead   lone       0.000        0.000
## 362  pStom  pIrri       0.000        0.000
## 363  pStom  pNerv       0.000        0.000
## 364  pStom pSleep       0.000        0.000
## 365  pStom     WB       0.000        0.000
## 366  pStom   lone       0.000        0.000
## 367  pBack  pIrri       0.000        0.000
## 368  pBack  pNerv       0.000        0.000
## 369  pBack     WB       0.000        0.000
## 370  pBack   lone       0.000        0.000
## 371  pIrri pSleep       0.000        0.000
## 372  pIrri pDizzy       0.000        0.000
## 373  pIrri     WB       0.000        0.000
## 374  pIrri   lone       0.000        0.000
## 375  pNerv pDizzy       0.000        0.000
## 376  pNerv   lone       0.000        0.000
## 377 pDizzy     WB       0.000        0.000
## 378 pDizzy   lone       0.000        0.000
```

#### Centrality

**The centrality estimates are relatively stable when the
correlation stability coefficient (CS coefficient) is ≥ 0.5**
while set at correlation stability = 0.7 (Epskamp et al.,
2018). Centrality indices with CS coefficient of 0.25-0.5 should be
interpreted with caution; CS coefficient < 0.25 indicated large
instability of the centrality index.

```
##   group        centrality     CS_r07
## 1  Boys          strength 0.67217788
## 2  Boys         closeness 0.28335234
## 3  Boys       betweenness 0.05017104
## 4  Boys expectedInfluence 0.75028506
## 5 Girls          strength 0.75000000
## 6 Girls         closeness 0.59451220
## 7 Girls       betweenness 0.51676829
## 8 Girls expectedInfluence 0.75000000
```

#### Bootstrapped Edges

### CZ

#### Figure

#### Edges

```
##       From     To Weight_Boys Weight_Girls
## 1     gPbl   gCfl       0.448        0.840
## 2     gPre   gTol       0.492        0.801
## 3     gEsc   sEsc       0.782        0.625
## 4     gDec   sDec       0.737        0.526
## 5     gCfl   sCfl       0.634        0.656
## 6     gDis   sDis       0.410        0.621
## 7     sPre   sTol       0.357        0.612
## 8     gTol   gWth       0.591        0.608
## 9     sPre   sWth       0.604        0.487
## 10    gPre   gWth       0.487        0.585
## 11    sDec   sCfl       0.578        0.428
## 12    gPrs   sPrs       0.576        0.496
## 13    sTol   sWth       0.503        0.576
## 14    sPbl   sCfl       0.416        0.560
## 15    gPre   gEsc       0.489        0.487
## 16    gWth   gDis       0.286        0.477
## 17    gPbl   gDis       0.458        0.291
## 18    gDec   gCfl       0.395        0.448
## 19    gPrs   gDec       0.276        0.445
## 20    gPbl   sPbl       0.383        0.436
## 21    gDis   gCfl       0.289        0.403
## 22    gWth   sWth       0.382        0.238
## 23    gTol   gDis       0.255        0.364
## 24    sDec   sDis       0.357        0.171
## 25    gWth   gCfl       0.347        0.000
## 26    sEsc   sPbl       0.338        0.210
## 27    gWth   gDec       0.303        0.336
## 28    gPbl   gDec       0.197        0.326
## 29    gPrs   gDis       0.317        0.187
## 30    sWth   sDec       0.315        0.263
## 31    gTol   sTol       0.300        0.284
## 32    sDis   sCfl       0.297        0.000
## 33    gTol   gPbl       0.294        0.098
## 34    sEsc   sDec       0.000        0.292
## 35    sTol   sPrs       0.289        0.132
## 36    pLow  pIrri       0.287        0.256
## 37    sTol   sPbl       0.283        0.269
## 38    gWth   gPbl       0.000        0.279
## 39    gEsc   gDec       0.236        0.278
## 40    sPre   sDis       0.277        0.063
## 41    gEsc   gCfl       0.274        0.106
## 42    sPrs   sEsc       0.267        0.264
## 43    gDec   gDis       0.261        0.118
## 44      WB   lone      -0.221       -0.259
## 45   pIrri  pNerv       0.246        0.217
## 46    gPre   gDis       0.000        0.240
## 47    sTol   sEsc       0.236        0.125
## 48    sPrs   sDis       0.168        0.232
## 49    gPrs   gCfl       0.229        0.200
## 50    sWth   sDis       0.229        0.087
## 51    gPrs   gPbl       0.139        0.228
## 52    pLow   lone       0.227        0.222
## 53    gTol   gPrs       0.225        0.198
## 54    sPre   sDec       0.223        0.204
## 55    sPrs   sPbl       0.219        0.109
## 56    gPre   gPbl       0.216        0.116
## 57    gPre   gDec       0.150        0.215
## 58   pIrri     WB      -0.207       -0.174
## 59    gTol   gEsc       0.042        0.204
## 60    gPrs   gEsc       0.073        0.203
## 61    gPre   gCfl       0.000        0.200
## 62    gPre   gPrs       0.052        0.191
## 63    gPre   sPre       0.185        0.139
## 64   pStom pDizzy       0.180        0.172
## 65    sPre   sPrs       0.179        0.139
## 66    sWth   sCfl       0.179        0.179
## 67    sPbl   sDec       0.136        0.178
## 68   pHead  pStom       0.178        0.150
## 69    sPre   sEsc       0.104        0.174
## 70    gTol   gDec       0.172        0.000
## 71    gWth   gEsc       0.172        0.154
## 72    sWth   sEsc       0.167        0.040
## 73    gTol   sDis       0.000        0.162
## 74    sEsc   sCfl       0.161        0.101
## 75   pHead pDizzy       0.120        0.152
## 76    sWth   sPbl       0.097        0.151
## 77    gEsc   gPbl       0.100        0.150
## 78    gDis   sDec       0.000        0.144
## 79    sEsc   lone       0.115        0.143
## 80   pHead  pBack       0.133        0.143
## 81    gWth   gPrs       0.052        0.142
## 82    gCfl   sPbl       0.068        0.142
## 83    sPrs   sDec       0.118        0.142
## 84    gPbl   sCfl       0.140        0.000
## 85    pLow  pNerv       0.072        0.138
## 86    sPre   sPbl       0.124        0.134
## 87    sEsc   sDis       0.000        0.134
## 88    sEsc   pLow       0.067        0.129
## 89    gTol   sWth       0.123        0.078
## 90  pSleep pDizzy       0.051        0.120
## 91    gTol   gCfl       0.115        0.000
## 92    pLow pDizzy       0.083        0.115
## 93    sEsc  pNerv       0.069        0.112
## 94   pIrri pSleep       0.070        0.112
## 95    gWth   sPbl       0.110        0.000
## 96   pIrri   lone       0.110        0.105
## 97   pHead  pNerv       0.107        0.063
## 98   pHead pSleep       0.101        0.080
## 99    sTol   sDis       0.059        0.099
## 100   gDis   sPbl       0.056        0.098
## 101   gWth   sPre       0.097        0.000
## 102   sTol   sCfl       0.064        0.092
## 103   pLow     WB      -0.058       -0.089
## 104  pNerv pSleep       0.061        0.087
## 105  pHead   pLow       0.055        0.085
## 106   gDec   sPbl       0.084        0.000
## 107   sPre   sCfl       0.078        0.084
## 108   sEsc     WB       0.000       -0.084
## 109  pBack pDizzy       0.084        0.074
## 110  pBack   pLow       0.054        0.082
## 111 pSleep     WB      -0.079       -0.048
## 112   sPbl  pIrri       0.000        0.077
## 113  pStom pSleep       0.076        0.000
## 114  pBack  pIrri       0.076        0.000
## 115   gPrs   sDis       0.074        0.000
## 116   gPrs   sPbl       0.073        0.000
## 117   sTol   sDec       0.073        0.064
## 118  pHead  pIrri       0.000        0.073
## 119   sCfl  pStom       0.000        0.071
## 120  pStom  pNerv       0.046        0.071
## 121   gEsc   sPrs       0.000        0.069
## 122  pBack  pNerv       0.000        0.067
## 123 pSleep   lone       0.055        0.065
## 124   gDis     WB       0.000       -0.064
## 125   gWth   sTol       0.062        0.000
## 126   gEsc   lone       0.000        0.059
## 127  pNerv   lone       0.049        0.059
## 128   sPbl   sDis       0.057        0.052
## 129   sTol     WB       0.000       -0.055
## 130  pBack pSleep       0.053        0.000
## 131   gPrs  pNerv       0.052        0.000
## 132  pStom  pIrri       0.049        0.000
## 133   gCfl pDizzy       0.048        0.000
## 134   sPrs   sCfl       0.000        0.047
## 135  pNerv pDizzy       0.047        0.043
## 136   sEsc  pIrri       0.046        0.000
## 137   gTol   sPbl       0.045        0.000
## 138   gEsc     WB      -0.045        0.000
## 139   pLow pSleep       0.045        0.000
## 140   gPbl   sDis       0.044        0.000
## 141   gDec   sPre       0.044        0.000
## 142   gEsc pSleep       0.000        0.043
## 143   gDis   sTol       0.042        0.000
## 144   gCfl  pNerv       0.042        0.000
## 145   gPrs   pLow      -0.041        0.000
## 146  pNerv     WB      -0.037       -0.039
## 147   sWth  pIrri       0.000        0.038
## 148  pStom  pBack       0.038        0.038
## 149   gPre  pIrri       0.000        0.035
## 150   gDis   sWth       0.035        0.000
## 151   sEsc  pHead       0.000        0.035
## 152   gTol   sPre       0.034        0.000
## 153   gTol     WB      -0.032        0.000
## 154   gPre   sTol       0.000        0.000
## 155   gPre   sWth       0.000        0.000
## 156   gPre   sPrs       0.000        0.000
## 157   gPre   sEsc       0.000        0.000
## 158   gPre   sPbl       0.000        0.000
## 159   gPre   sDec       0.000        0.000
## 160   gPre   sDis       0.000        0.000
## 161   gPre   sCfl       0.000        0.000
## 162   gPre  pHead       0.000        0.000
## 163   gPre  pStom       0.000        0.000
## 164   gPre  pBack       0.000        0.000
## 165   gPre   pLow       0.000        0.000
## 166   gPre  pNerv       0.000        0.000
## 167   gPre pSleep       0.000        0.000
## 168   gPre pDizzy       0.000        0.000
## 169   gPre     WB       0.000        0.000
## 170   gPre   lone       0.000        0.000
## 171   gTol   sPrs       0.000        0.000
## 172   gTol   sEsc       0.000        0.000
## 173   gTol   sDec       0.000        0.000
## 174   gTol   sCfl       0.000        0.000
## 175   gTol  pHead       0.000        0.000
## 176   gTol  pStom       0.000        0.000
## 177   gTol  pBack       0.000        0.000
## 178   gTol   pLow       0.000        0.000
## 179   gTol  pIrri       0.000        0.000
## 180   gTol  pNerv       0.000        0.000
## 181   gTol pSleep       0.000        0.000
## 182   gTol pDizzy       0.000        0.000
## 183   gTol   lone       0.000        0.000
## 184   gWth   sPrs       0.000        0.000
## 185   gWth   sEsc       0.000        0.000
## 186   gWth   sDec       0.000        0.000
## 187   gWth   sDis       0.000        0.000
## 188   gWth   sCfl       0.000        0.000
## 189   gWth  pHead       0.000        0.000
## 190   gWth  pStom       0.000        0.000
## 191   gWth  pBack       0.000        0.000
## 192   gWth   pLow       0.000        0.000
## 193   gWth  pIrri       0.000        0.000
## 194   gWth  pNerv       0.000        0.000
## 195   gWth pSleep       0.000        0.000
## 196   gWth pDizzy       0.000        0.000
## 197   gWth     WB       0.000        0.000
## 198   gWth   lone       0.000        0.000
## 199   gPrs   sPre       0.000        0.000
## 200   gPrs   sTol       0.000        0.000
## 201   gPrs   sWth       0.000        0.000
## 202   gPrs   sEsc       0.000        0.000
## 203   gPrs   sDec       0.000        0.000
## 204   gPrs   sCfl       0.000        0.000
## 205   gPrs  pHead       0.000        0.000
## 206   gPrs  pStom       0.000        0.000
## 207   gPrs  pBack       0.000        0.000
## 208   gPrs  pIrri       0.000        0.000
## 209   gPrs pSleep       0.000        0.000
## 210   gPrs pDizzy       0.000        0.000
## 211   gPrs     WB       0.000        0.000
## 212   gPrs   lone       0.000        0.000
## 213   gEsc   gDis       0.000        0.000
## 214   gEsc   sPre       0.000        0.000
## 215   gEsc   sTol       0.000        0.000
## 216   gEsc   sWth       0.000        0.000
## 217   gEsc   sPbl       0.000        0.000
## 218   gEsc   sDec       0.000        0.000
## 219   gEsc   sDis       0.000        0.000
## 220   gEsc   sCfl       0.000        0.000
## 221   gEsc  pHead       0.000        0.000
## 222   gEsc  pStom       0.000        0.000
## 223   gEsc  pBack       0.000        0.000
## 224   gEsc   pLow       0.000        0.000
## 225   gEsc  pIrri       0.000        0.000
## 226   gEsc  pNerv       0.000        0.000
## 227   gEsc pDizzy       0.000        0.000
## 228   gPbl   sPre       0.000        0.000
## 229   gPbl   sTol       0.000        0.000
## 230   gPbl   sWth       0.000        0.000
## 231   gPbl   sPrs       0.000        0.000
## 232   gPbl   sEsc       0.000        0.000
## 233   gPbl   sDec       0.000        0.000
## 234   gPbl  pHead       0.000        0.000
## 235   gPbl  pStom       0.000        0.000
## 236   gPbl  pBack       0.000        0.000
## 237   gPbl   pLow       0.000        0.000
## 238   gPbl  pIrri       0.000        0.000
## 239   gPbl  pNerv       0.000        0.000
## 240   gPbl pSleep       0.000        0.000
## 241   gPbl pDizzy       0.000        0.000
## 242   gPbl     WB       0.000        0.000
## 243   gPbl   lone       0.000        0.000
## 244   gDec   sTol       0.000        0.000
## 245   gDec   sWth       0.000        0.000
## 246   gDec   sPrs       0.000        0.000
## 247   gDec   sEsc       0.000        0.000
## 248   gDec   sDis       0.000        0.000
## 249   gDec   sCfl       0.000        0.000
## 250   gDec  pHead       0.000        0.000
## 251   gDec  pStom       0.000        0.000
## 252   gDec  pBack       0.000        0.000
## 253   gDec   pLow       0.000        0.000
## 254   gDec  pIrri       0.000        0.000
## 255   gDec  pNerv       0.000        0.000
## 256   gDec pSleep       0.000        0.000
## 257   gDec pDizzy       0.000        0.000
## 258   gDec     WB       0.000        0.000
## 259   gDec   lone       0.000        0.000
## 260   gDis   sPre       0.000        0.000
## 261   gDis   sPrs       0.000        0.000
## 262   gDis   sEsc       0.000        0.000
## 263   gDis   sCfl       0.000        0.000
## 264   gDis  pHead       0.000        0.000
## 265   gDis  pStom       0.000        0.000
## 266   gDis  pBack       0.000        0.000
## 267   gDis   pLow       0.000        0.000
## 268   gDis  pIrri       0.000        0.000
## 269   gDis  pNerv       0.000        0.000
## 270   gDis pSleep       0.000        0.000
## 271   gDis pDizzy       0.000        0.000
## 272   gDis   lone       0.000        0.000
## 273   gCfl   sPre       0.000        0.000
## 274   gCfl   sTol       0.000        0.000
## 275   gCfl   sWth       0.000        0.000
## 276   gCfl   sPrs       0.000        0.000
## 277   gCfl   sEsc       0.000        0.000
## 278   gCfl   sDec       0.000        0.000
## 279   gCfl   sDis       0.000        0.000
## 280   gCfl  pHead       0.000        0.000
## 281   gCfl  pStom       0.000        0.000
## 282   gCfl  pBack       0.000        0.000
## 283   gCfl   pLow       0.000        0.000
## 284   gCfl  pIrri       0.000        0.000
## 285   gCfl pSleep       0.000        0.000
## 286   gCfl     WB       0.000        0.000
## 287   gCfl   lone       0.000        0.000
## 288   sPre  pHead       0.000        0.000
## 289   sPre  pStom       0.000        0.000
## 290   sPre  pBack       0.000        0.000
## 291   sPre   pLow       0.000        0.000
## 292   sPre  pIrri       0.000        0.000
## 293   sPre  pNerv       0.000        0.000
## 294   sPre pSleep       0.000        0.000
## 295   sPre pDizzy       0.000        0.000
## 296   sPre     WB       0.000        0.000
## 297   sPre   lone       0.000        0.000
## 298   sTol  pHead       0.000        0.000
## 299   sTol  pStom       0.000        0.000
## 300   sTol  pBack       0.000        0.000
## 301   sTol   pLow       0.000        0.000
## 302   sTol  pIrri       0.000        0.000
## 303   sTol  pNerv       0.000        0.000
## 304   sTol pSleep       0.000        0.000
## 305   sTol pDizzy       0.000        0.000
## 306   sTol   lone       0.000        0.000
## 307   sWth   sPrs       0.000        0.000
## 308   sWth  pHead       0.000        0.000
## 309   sWth  pStom       0.000        0.000
## 310   sWth  pBack       0.000        0.000
## 311   sWth   pLow       0.000        0.000
## 312   sWth  pNerv       0.000        0.000
## 313   sWth pSleep       0.000        0.000
## 314   sWth pDizzy       0.000        0.000
## 315   sWth     WB       0.000        0.000
## 316   sWth   lone       0.000        0.000
## 317   sPrs  pHead       0.000        0.000
## 318   sPrs  pStom       0.000        0.000
## 319   sPrs  pBack       0.000        0.000
## 320   sPrs   pLow       0.000        0.000
## 321   sPrs  pIrri       0.000        0.000
## 322   sPrs  pNerv       0.000        0.000
## 323   sPrs pSleep       0.000        0.000
## 324   sPrs pDizzy       0.000        0.000
## 325   sPrs     WB       0.000        0.000
## 326   sPrs   lone       0.000        0.000
## 327   sEsc  pStom       0.000        0.000
## 328   sEsc  pBack       0.000        0.000
## 329   sEsc pSleep       0.000        0.000
## 330   sEsc pDizzy       0.000        0.000
## 331   sPbl  pHead       0.000        0.000
## 332   sPbl  pStom       0.000        0.000
## 333   sPbl  pBack       0.000        0.000
## 334   sPbl   pLow       0.000        0.000
## 335   sPbl  pNerv       0.000        0.000
## 336   sPbl pSleep       0.000        0.000
## 337   sPbl pDizzy       0.000        0.000
## 338   sPbl     WB       0.000        0.000
## 339   sPbl   lone       0.000        0.000
## 340   sDec  pHead       0.000        0.000
## 341   sDec  pStom       0.000        0.000
## 342   sDec  pBack       0.000        0.000
## 343   sDec   pLow       0.000        0.000
## 344   sDec  pIrri       0.000        0.000
## 345   sDec  pNerv       0.000        0.000
## 346   sDec pSleep       0.000        0.000
## 347   sDec pDizzy       0.000        0.000
## 348   sDec     WB       0.000        0.000
## 349   sDec   lone       0.000        0.000
## 350   sDis  pHead       0.000        0.000
## 351   sDis  pStom       0.000        0.000
## 352   sDis  pBack       0.000        0.000
## 353   sDis   pLow       0.000        0.000
## 354   sDis  pIrri       0.000        0.000
## 355   sDis  pNerv       0.000        0.000
## 356   sDis pSleep       0.000        0.000
## 357   sDis pDizzy       0.000        0.000
## 358   sDis     WB       0.000        0.000
## 359   sDis   lone       0.000        0.000
## 360   sCfl  pHead       0.000        0.000
## 361   sCfl  pBack       0.000        0.000
## 362   sCfl   pLow       0.000        0.000
## 363   sCfl  pIrri       0.000        0.000
## 364   sCfl  pNerv       0.000        0.000
## 365   sCfl pSleep       0.000        0.000
## 366   sCfl pDizzy       0.000        0.000
## 367   sCfl     WB       0.000        0.000
## 368   sCfl   lone       0.000        0.000
## 369  pHead     WB       0.000        0.000
## 370  pHead   lone       0.000        0.000
## 371  pStom   pLow       0.000        0.000
## 372  pStom     WB       0.000        0.000
## 373  pStom   lone       0.000        0.000
## 374  pBack     WB       0.000        0.000
## 375  pBack   lone       0.000        0.000
## 376  pIrri pDizzy       0.000        0.000
## 377 pDizzy     WB       0.000        0.000
## 378 pDizzy   lone       0.000        0.000
```

#### Centrality

**The centrality estimates are relatively stable when the
correlation stability coefficient (CS coefficient) is ≥ 0.5**
while set at correlation stability = 0.7 (Epskamp et al.,
2018). Centrality indices with CS coefficient of 0.25-0.5 should be
interpreted with caution; CS coefficient < 0.25 indicated large
instability of the centrality index.

```
##   group        centrality    CS_r07
## 1  Boys          strength 0.7498924
## 2  Boys         closeness 0.6721911
## 3  Boys       betweenness 0.1278519
## 4  Boys expectedInfluence 0.7498924
## 5 Girls          strength 0.7498623
## 6 Girls         closeness 0.7498623
## 7 Girls       betweenness 0.4388430
## 8 Girls expectedInfluence 0.7498623
```

#### Bootstrapped Edges

### EE

#### Figure

#### Edges

```
##       From     To Weight_Boys Weight_Girls
## 1     sPre   sTol       0.708        0.644
## 2     gTol   gWth       0.575        0.698
## 3     sPbl   sCfl       0.628        0.465
## 4     gDec   sDec       0.584        0.460
## 5     gPbl   gDis       0.196        0.568
## 6     gCfl   sCfl       0.481        0.562
## 7     gPbl   gCfl       0.533        0.546
## 8     gDec   gDis       0.512        0.234
## 9     sDec   sCfl       0.410        0.504
## 10    sTol   sWth       0.435        0.501
## 11    gPre   gTol       0.375        0.488
## 12    gEsc   sEsc       0.471        0.455
## 13    sWth   sDec       0.423        0.167
## 14    gDis   gCfl       0.410        0.184
## 15    gPrs   gPbl       0.397        0.324
## 16    gPbl   sPbl       0.382        0.103
## 17    sPbl   sDec       0.380        0.377
## 18    gDis   sDis       0.354        0.371
## 19    gPre   gCfl       0.095        0.370
## 20    sDis   sCfl       0.260        0.362
## 21   pIrri  pNerv       0.323        0.357
## 22    gPrs   gDec       0.216        0.354
## 23    pLow  pIrri       0.313        0.354
## 24    gPrs   gDis       0.346        0.227
## 25    sPbl   sDis       0.342        0.000
## 26    sTol   sPbl       0.339        0.205
## 27    sPre   sWth       0.262        0.337
## 28    sTol   sEsc       0.332        0.144
## 29    gPre   gEsc       0.327        0.241
## 30    gTol   sTol       0.321        0.197
## 31    sDec   sDis       0.309        0.000
## 32    gTol   gDis       0.119        0.305
## 33    gDec   gCfl       0.304        0.097
## 34    gPre   gDis       0.302        0.235
## 35    gTol   gPrs       0.301        0.293
## 36    gPre   gWth       0.300        0.279
## 37   pHead  pStom       0.297        0.233
## 38    sPrs   sEsc       0.244        0.291
## 39    sTol   sCfl       0.000        0.279
## 40    gWth   sWth       0.214        0.275
## 41    sWth   sCfl       0.273        0.087
## 42    gPre   gPrs       0.139        0.271
## 43    pLow   lone       0.266        0.257
## 44      WB   lone      -0.213       -0.266
## 45    gPbl   gDec       0.170        0.265
## 46    gWth   gEsc       0.070        0.264
## 47    gTol   gPbl       0.261        0.131
## 48    sPre   sCfl       0.252        0.000
## 49    sPrs   sDec       0.247        0.175
## 50    gPrs   gEsc       0.052        0.244
## 51    gWth   gCfl       0.194        0.236
## 52    gWth   gDec       0.160        0.222
## 53    gEsc   gPbl       0.168        0.216
## 54    gPre   gDec       0.215        0.159
## 55    gWth   gDis       0.214        0.000
## 56    gPrs   sPrs       0.213        0.179
## 57   pHead pDizzy       0.104        0.205
## 58  pSleep pDizzy       0.205        0.176
## 59    sPrs   sDis       0.142        0.191
## 60    sEsc   sDis       0.109        0.187
## 61    sEsc   sPbl       0.182        0.000
## 62    sPre   sDec       0.180        0.162
## 63    gDis   sTol       0.000        0.178
## 64    gCfl   sPbl       0.000        0.176
## 65    sTol   sDec       0.174        0.000
## 66    gTol   gEsc       0.173        0.072
## 67    gPbl   sCfl       0.000        0.173
## 68    gPrs   gCfl       0.170        0.096
## 69    sPre   sPrs       0.168        0.000
## 70    gPre   gPbl       0.141        0.167
## 71    sTol   sPrs       0.167        0.147
## 72    gEsc   gDec       0.137        0.161
## 73    gPre   sPre       0.106        0.159
## 74    gPre   sTol       0.133        0.148
## 75    sPrs   sPbl       0.147        0.000
## 76   pBack pDizzy       0.120        0.143
## 77    gTol   gDec       0.086        0.138
## 78    sEsc   sCfl       0.136        0.076
## 79  pSleep     WB      -0.128       -0.106
## 80   pIrri     WB      -0.120       -0.127
## 81    sWth   sEsc       0.126        0.095
## 82    pLow     WB      -0.078       -0.123
## 83    pLow  pNerv       0.117        0.122
## 84    gTol   sWth       0.000        0.113
## 85    gDis   sPbl       0.110        0.000
## 86    sEsc   pLow       0.068        0.110
## 87    gTol   gCfl       0.000        0.108
## 88    gWth   sDec       0.107        0.000
## 89   pHead  pBack       0.107        0.100
## 90    sEsc   lone       0.104        0.067
## 91    sWth   sDis       0.103        0.000
## 92    sPre   sEsc       0.000        0.101
## 93    sEsc pDizzy       0.000        0.099
## 94    gDec   sTol       0.000        0.098
## 95   pStom  pBack       0.098        0.000
## 96    sPre   sPbl       0.000        0.096
## 97   pStom pDizzy       0.000        0.096
## 98    gPbl   sDec       0.095        0.000
## 99   pBack  pNerv       0.093        0.000
## 100   sWth   sPbl       0.090        0.000
## 101   sEsc  pNerv       0.000        0.088
## 102   gWth   sTol       0.000        0.087
## 103   gWth   gPbl       0.074        0.086
## 104  pNerv     WB      -0.083       -0.079
## 105   gPrs   sDec       0.081        0.000
## 106  pNerv pSleep       0.079        0.000
## 107   sWth   lone       0.000        0.077
## 108  pStom   pLow       0.000        0.075
## 109   gTol   sPrs       0.000        0.074
## 110   gDis   sPre       0.074        0.000
## 111  pHead  pNerv       0.052        0.073
## 112   gPbl   sDis       0.000        0.072
## 113   gEsc   lone       0.071        0.000
## 114   gWth   gPrs       0.069        0.000
## 115   gEsc pSleep       0.069        0.000
## 116   gEsc     WB      -0.064        0.000
## 117   sPre   sDis       0.062        0.000
## 118  pIrri   lone       0.062        0.000
## 119  pBack pSleep       0.060        0.000
## 120  pNerv pDizzy       0.000        0.060
## 121   gEsc  pNerv       0.049        0.000
## 122   gDec   sPrs       0.046        0.000
## 123   gPre   sWth       0.000        0.000
## 124   gPre   sPrs       0.000        0.000
## 125   gPre   sEsc       0.000        0.000
## 126   gPre   sPbl       0.000        0.000
## 127   gPre   sDec       0.000        0.000
## 128   gPre   sDis       0.000        0.000
## 129   gPre   sCfl       0.000        0.000
## 130   gPre  pHead       0.000        0.000
## 131   gPre  pStom       0.000        0.000
## 132   gPre  pBack       0.000        0.000
## 133   gPre   pLow       0.000        0.000
## 134   gPre  pIrri       0.000        0.000
## 135   gPre  pNerv       0.000        0.000
## 136   gPre pSleep       0.000        0.000
## 137   gPre pDizzy       0.000        0.000
## 138   gPre     WB       0.000        0.000
## 139   gPre   lone       0.000        0.000
## 140   gTol   sPre       0.000        0.000
## 141   gTol   sEsc       0.000        0.000
## 142   gTol   sPbl       0.000        0.000
## 143   gTol   sDec       0.000        0.000
## 144   gTol   sDis       0.000        0.000
## 145   gTol   sCfl       0.000        0.000
## 146   gTol  pHead       0.000        0.000
## 147   gTol  pStom       0.000        0.000
## 148   gTol  pBack       0.000        0.000
## 149   gTol   pLow       0.000        0.000
## 150   gTol  pIrri       0.000        0.000
## 151   gTol  pNerv       0.000        0.000
## 152   gTol pSleep       0.000        0.000
## 153   gTol pDizzy       0.000        0.000
## 154   gTol     WB       0.000        0.000
## 155   gTol   lone       0.000        0.000
## 156   gWth   sPre       0.000        0.000
## 157   gWth   sPrs       0.000        0.000
## 158   gWth   sEsc       0.000        0.000
## 159   gWth   sPbl       0.000        0.000
## 160   gWth   sDis       0.000        0.000
## 161   gWth   sCfl       0.000        0.000
## 162   gWth  pHead       0.000        0.000
## 163   gWth  pStom       0.000        0.000
## 164   gWth  pBack       0.000        0.000
## 165   gWth   pLow       0.000        0.000
## 166   gWth  pIrri       0.000        0.000
## 167   gWth  pNerv       0.000        0.000
## 168   gWth pSleep       0.000        0.000
## 169   gWth pDizzy       0.000        0.000
## 170   gWth     WB       0.000        0.000
## 171   gWth   lone       0.000        0.000
## 172   gPrs   sPre       0.000        0.000
## 173   gPrs   sTol       0.000        0.000
## 174   gPrs   sWth       0.000        0.000
## 175   gPrs   sEsc       0.000        0.000
## 176   gPrs   sPbl       0.000        0.000
## 177   gPrs   sDis       0.000        0.000
## 178   gPrs   sCfl       0.000        0.000
## 179   gPrs  pHead       0.000        0.000
## 180   gPrs  pStom       0.000        0.000
## 181   gPrs  pBack       0.000        0.000
## 182   gPrs   pLow       0.000        0.000
## 183   gPrs  pIrri       0.000        0.000
## 184   gPrs  pNerv       0.000        0.000
## 185   gPrs pSleep       0.000        0.000
## 186   gPrs pDizzy       0.000        0.000
## 187   gPrs     WB       0.000        0.000
## 188   gPrs   lone       0.000        0.000
## 189   gEsc   gDis       0.000        0.000
## 190   gEsc   gCfl       0.000        0.000
## 191   gEsc   sPre       0.000        0.000
## 192   gEsc   sTol       0.000        0.000
## 193   gEsc   sWth       0.000        0.000
## 194   gEsc   sPrs       0.000        0.000
## 195   gEsc   sPbl       0.000        0.000
## 196   gEsc   sDec       0.000        0.000
## 197   gEsc   sDis       0.000        0.000
## 198   gEsc   sCfl       0.000        0.000
## 199   gEsc  pHead       0.000        0.000
## 200   gEsc  pStom       0.000        0.000
## 201   gEsc  pBack       0.000        0.000
## 202   gEsc   pLow       0.000        0.000
## 203   gEsc  pIrri       0.000        0.000
## 204   gEsc pDizzy       0.000        0.000
## 205   gPbl   sPre       0.000        0.000
## 206   gPbl   sTol       0.000        0.000
## 207   gPbl   sWth       0.000        0.000
## 208   gPbl   sPrs       0.000        0.000
## 209   gPbl   sEsc       0.000        0.000
## 210   gPbl  pHead       0.000        0.000
## 211   gPbl  pStom       0.000        0.000
## 212   gPbl  pBack       0.000        0.000
## 213   gPbl   pLow       0.000        0.000
## 214   gPbl  pIrri       0.000        0.000
## 215   gPbl  pNerv       0.000        0.000
## 216   gPbl pSleep       0.000        0.000
## 217   gPbl pDizzy       0.000        0.000
## 218   gPbl     WB       0.000        0.000
## 219   gPbl   lone       0.000        0.000
## 220   gDec   sPre       0.000        0.000
## 221   gDec   sWth       0.000        0.000
## 222   gDec   sEsc       0.000        0.000
## 223   gDec   sPbl       0.000        0.000
## 224   gDec   sDis       0.000        0.000
## 225   gDec   sCfl       0.000        0.000
## 226   gDec  pHead       0.000        0.000
## 227   gDec  pStom       0.000        0.000
## 228   gDec  pBack       0.000        0.000
## 229   gDec   pLow       0.000        0.000
## 230   gDec  pIrri       0.000        0.000
## 231   gDec  pNerv       0.000        0.000
## 232   gDec pSleep       0.000        0.000
## 233   gDec pDizzy       0.000        0.000
## 234   gDec     WB       0.000        0.000
## 235   gDec   lone       0.000        0.000
## 236   gDis   sWth       0.000        0.000
## 237   gDis   sPrs       0.000        0.000
## 238   gDis   sEsc       0.000        0.000
## 239   gDis   sDec       0.000        0.000
## 240   gDis   sCfl       0.000        0.000
## 241   gDis  pHead       0.000        0.000
## 242   gDis  pStom       0.000        0.000
## 243   gDis  pBack       0.000        0.000
## 244   gDis   pLow       0.000        0.000
## 245   gDis  pIrri       0.000        0.000
## 246   gDis  pNerv       0.000        0.000
## 247   gDis pSleep       0.000        0.000
## 248   gDis pDizzy       0.000        0.000
## 249   gDis     WB       0.000        0.000
## 250   gDis   lone       0.000        0.000
## 251   gCfl   sPre       0.000        0.000
## 252   gCfl   sTol       0.000        0.000
## 253   gCfl   sWth       0.000        0.000
## 254   gCfl   sPrs       0.000        0.000
## 255   gCfl   sEsc       0.000        0.000
## 256   gCfl   sDec       0.000        0.000
## 257   gCfl   sDis       0.000        0.000
## 258   gCfl  pHead       0.000        0.000
## 259   gCfl  pStom       0.000        0.000
## 260   gCfl  pBack       0.000        0.000
## 261   gCfl   pLow       0.000        0.000
## 262   gCfl  pIrri       0.000        0.000
## 263   gCfl  pNerv       0.000        0.000
## 264   gCfl pSleep       0.000        0.000
## 265   gCfl pDizzy       0.000        0.000
## 266   gCfl     WB       0.000        0.000
## 267   gCfl   lone       0.000        0.000
## 268   sPre  pHead       0.000        0.000
## 269   sPre  pStom       0.000        0.000
## 270   sPre  pBack       0.000        0.000
## 271   sPre   pLow       0.000        0.000
## 272   sPre  pIrri       0.000        0.000
## 273   sPre  pNerv       0.000        0.000
## 274   sPre pSleep       0.000        0.000
## 275   sPre pDizzy       0.000        0.000
## 276   sPre     WB       0.000        0.000
## 277   sPre   lone       0.000        0.000
## 278   sTol   sDis       0.000        0.000
## 279   sTol  pHead       0.000        0.000
## 280   sTol  pStom       0.000        0.000
## 281   sTol  pBack       0.000        0.000
## 282   sTol   pLow       0.000        0.000
## 283   sTol  pIrri       0.000        0.000
## 284   sTol  pNerv       0.000        0.000
## 285   sTol pSleep       0.000        0.000
## 286   sTol pDizzy       0.000        0.000
## 287   sTol     WB       0.000        0.000
## 288   sTol   lone       0.000        0.000
## 289   sWth   sPrs       0.000        0.000
## 290   sWth  pHead       0.000        0.000
## 291   sWth  pStom       0.000        0.000
## 292   sWth  pBack       0.000        0.000
## 293   sWth   pLow       0.000        0.000
## 294   sWth  pIrri       0.000        0.000
## 295   sWth  pNerv       0.000        0.000
## 296   sWth pSleep       0.000        0.000
## 297   sWth pDizzy       0.000        0.000
## 298   sWth     WB       0.000        0.000
## 299   sPrs   sCfl       0.000        0.000
## 300   sPrs  pHead       0.000        0.000
## 301   sPrs  pStom       0.000        0.000
## 302   sPrs  pBack       0.000        0.000
## 303   sPrs   pLow       0.000        0.000
## 304   sPrs  pIrri       0.000        0.000
## 305   sPrs  pNerv       0.000        0.000
## 306   sPrs pSleep       0.000        0.000
## 307   sPrs pDizzy       0.000        0.000
## 308   sPrs     WB       0.000        0.000
## 309   sPrs   lone       0.000        0.000
## 310   sEsc   sDec       0.000        0.000
## 311   sEsc  pHead       0.000        0.000
## 312   sEsc  pStom       0.000        0.000
## 313   sEsc  pBack       0.000        0.000
## 314   sEsc  pIrri       0.000        0.000
## 315   sEsc pSleep       0.000        0.000
## 316   sEsc     WB       0.000        0.000
## 317   sPbl  pHead       0.000        0.000
## 318   sPbl  pStom       0.000        0.000
## 319   sPbl  pBack       0.000        0.000
## 320   sPbl   pLow       0.000        0.000
## 321   sPbl  pIrri       0.000        0.000
## 322   sPbl  pNerv       0.000        0.000
## 323   sPbl pSleep       0.000        0.000
## 324   sPbl pDizzy       0.000        0.000
## 325   sPbl     WB       0.000        0.000
## 326   sPbl   lone       0.000        0.000
## 327   sDec  pHead       0.000        0.000
## 328   sDec  pStom       0.000        0.000
## 329   sDec  pBack       0.000        0.000
## 330   sDec   pLow       0.000        0.000
## 331   sDec  pIrri       0.000        0.000
## 332   sDec  pNerv       0.000        0.000
## 333   sDec pSleep       0.000        0.000
## 334   sDec pDizzy       0.000        0.000
## 335   sDec     WB       0.000        0.000
## 336   sDec   lone       0.000        0.000
## 337   sDis  pHead       0.000        0.000
## 338   sDis  pStom       0.000        0.000
## 339   sDis  pBack       0.000        0.000
## 340   sDis   pLow       0.000        0.000
## 341   sDis  pIrri       0.000        0.000
## 342   sDis  pNerv       0.000        0.000
## 343   sDis pSleep       0.000        0.000
## 344   sDis pDizzy       0.000        0.000
## 345   sDis     WB       0.000        0.000
## 346   sDis   lone       0.000        0.000
## 347   sCfl  pHead       0.000        0.000
## 348   sCfl  pStom       0.000        0.000
## 349   sCfl  pBack       0.000        0.000
## 350   sCfl   pLow       0.000        0.000
## 351   sCfl  pIrri       0.000        0.000
## 352   sCfl  pNerv       0.000        0.000
## 353   sCfl pSleep       0.000        0.000
## 354   sCfl pDizzy       0.000        0.000
## 355   sCfl     WB       0.000        0.000
## 356   sCfl   lone       0.000        0.000
## 357  pHead   pLow       0.000        0.000
## 358  pHead  pIrri       0.000        0.000
## 359  pHead pSleep       0.000        0.000
## 360  pHead     WB       0.000        0.000
## 361  pHead   lone       0.000        0.000
## 362  pStom  pIrri       0.000        0.000
## 363  pStom  pNerv       0.000        0.000
## 364  pStom pSleep       0.000        0.000
## 365  pStom     WB       0.000        0.000
## 366  pStom   lone       0.000        0.000
## 367  pBack   pLow       0.000        0.000
## 368  pBack  pIrri       0.000        0.000
## 369  pBack     WB       0.000        0.000
## 370  pBack   lone       0.000        0.000
## 371   pLow pSleep       0.000        0.000
## 372   pLow pDizzy       0.000        0.000
## 373  pIrri pSleep       0.000        0.000
## 374  pIrri pDizzy       0.000        0.000
## 375  pNerv   lone       0.000        0.000
## 376 pSleep   lone       0.000        0.000
## 377 pDizzy     WB       0.000        0.000
## 378 pDizzy   lone       0.000        0.000
```

#### Centrality

**The centrality estimates are relatively stable when the
correlation stability coefficient (CS coefficient) is ≥ 0.5**
while set at correlation stability = 0.7 (Epskamp et al.,
2018). Centrality indices with CS coefficient of 0.25-0.5 should be
interpreted with caution; CS coefficient < 0.25 indicated large
instability of the centrality index.

```
##   group        centrality    CS_r07
## 1  Boys          strength 0.7500149
## 2  Boys         closeness 0.7500149
## 3  Boys       betweenness 0.7500149
## 4  Boys expectedInfluence 0.7500149
## 5 Girls          strength 0.7500149
## 6 Girls         closeness 0.7500149
## 7 Girls       betweenness 0.7500149
## 8 Girls expectedInfluence 0.7500149
```

#### Bootstrapped Edges

### GB-ENG

#### Figure

#### Edges

```
##       From     To Weight_Boys Weight_Girls
## 1     gPbl   gCfl       0.569        0.957
## 2     sPre   sTol       0.688        0.787
## 3     sTol   sWth       0.536        0.711
## 4     sPbl   sCfl       0.584        0.680
## 5     gCfl   sCfl       0.660        0.369
## 6     gDis   sDis       0.650        0.550
## 7     gEsc   sEsc       0.590        0.514
## 8     sDec   sCfl       0.575        0.520
## 9     gPre   gTol       0.396        0.530
## 10    gPre   gWth       0.509        0.326
## 11    gTol   gWth       0.449        0.507
## 12    gDec   sDec       0.500        0.435
## 13    sPre   sWth       0.498        0.298
## 14    gDec   gDis       0.239        0.481
## 15    gPre   gEsc       0.306        0.461
## 16    gWth   gCfl       0.342        0.444
## 17    gWth   gDis       0.000        0.435
## 18    sPrs   sEsc       0.317        0.435
## 19    gTol   gDis       0.398        0.336
## 20    gPbl   gDec       0.395        0.281
## 21    gTol   gEsc       0.335        0.372
## 22    gPbl   sPbl       0.273        0.355
## 23    gPbl   gDis       0.311        0.349
## 24    sPrs   sDis       0.339        0.094
## 25    gPre   gPrs       0.137        0.324
## 26    gWth   sWth       0.322        0.214
## 27    gPre   gPbl       0.000        0.301
## 28    gDec   gCfl       0.300        0.212
## 29    gPrs   gDis       0.183        0.299
## 30    gPre   gDec       0.169        0.290
## 31    sWth   sCfl       0.290        0.115
## 32    sDis   sCfl       0.286        0.000
## 33    gPrs   gDec       0.126        0.285
## 34    sEsc   sDec       0.280        0.111
## 35    sPbl   sDec       0.125        0.265
## 36    pLow  pIrri       0.168        0.261
## 37    gDis   gCfl       0.258        0.078
## 38    sEsc   sPbl       0.256        0.114
## 39    pLow   lone       0.253        0.207
## 40    gEsc   gPbl       0.245        0.000
## 41    sTol   sPbl       0.245        0.244
## 42      WB   lone      -0.233       -0.241
## 43    gPrs   gCfl       0.146        0.238
## 44    pLow     WB      -0.223       -0.236
## 45    sPrs   sDec       0.234        0.199
## 46   pHead pDizzy       0.227        0.230
## 47    pLow  pNerv       0.230        0.175
## 48   pHead  pStom       0.203        0.222
## 49    sEsc   sDis       0.154        0.221
## 50    gWth   gEsc       0.189        0.219
## 51    sPre   sEsc       0.000        0.217
## 52    gTol   gPrs       0.216        0.000
## 53    gPre   sPre       0.159        0.213
## 54    gPrs   gPbl       0.202        0.000
## 55    gPre   gDis       0.000        0.199
## 56    sPre   sDis       0.166        0.196
## 57    sTol   sEsc       0.193        0.109
## 58    sTol   sPrs       0.191        0.000
## 59   pHead  pBack       0.000        0.191
## 60    gPrs   sPrs       0.160        0.187
## 61    sPre   sCfl       0.184        0.122
## 62    sTol   sDec       0.000        0.184
## 63    gDec   sPbl       0.000        0.181
## 64    sWth   sDis       0.173        0.180
## 65    gTol   sTol       0.089        0.177
## 66    gWth   gDec       0.177        0.000
## 67    gWth   gPbl       0.171        0.000
## 68    gCfl   sWth       0.000        0.171
## 69    gPrs   gEsc       0.168        0.000
## 70    gCfl   sPbl       0.166        0.134
## 71    sPrs   sPbl       0.165        0.065
## 72   pStom pDizzy       0.101        0.163
## 73    sWth   sEsc       0.162        0.076
## 74    gPbl   sCfl       0.160        0.000
## 75    gPrs   sPbl       0.156        0.000
## 76    sDec   sDis       0.082        0.154
## 77    sPre   sPbl       0.147        0.000
## 78    sEsc     WB       0.000       -0.139
## 79  pSleep     WB      -0.139       -0.104
## 80    gPrs   sDis       0.137        0.000
## 81    gDis     WB       0.000       -0.135
## 82   pNerv   lone       0.092        0.132
## 83   pIrri pSleep       0.127        0.000
## 84    gPbl   sDec       0.088        0.125
## 85    gEsc   gDec       0.124        0.000
## 86    sWth   sPbl       0.124        0.000
## 87    gTol   gDec       0.000        0.122
## 88    gWth   sTol       0.120        0.000
## 89    sPbl   sDis       0.113        0.120
## 90   pNerv pDizzy       0.119        0.063
## 91    gDis   sTol       0.000        0.117
## 92    sWth   sPrs       0.000        0.117
## 93   pNerv pSleep       0.000        0.117
## 94  pSleep pDizzy       0.109        0.092
## 95   pStom  pBack       0.101        0.103
## 96    gTol   gPbl       0.098        0.082
## 97    sEsc   lone       0.097        0.000
## 98   pIrri  pNerv       0.050        0.096
## 99    sEsc   pLow       0.095        0.000
## 100   sEsc  pNerv       0.095        0.000
## 101   gTol   sWth       0.090        0.069
## 102  pStom  pIrri       0.090        0.000
## 103  pHead   pLow       0.088        0.074
## 104   gTol   sDec       0.000        0.085
## 105   gPbl  pIrri       0.081        0.000
## 106   pLow pDizzy       0.000        0.080
## 107  pStom  pNerv       0.077        0.000
## 108   gEsc   gDis       0.075        0.000
## 109   gDis   sPre       0.073        0.000
## 110   sPre   sDec       0.071        0.000
## 111   sTol   sDis       0.000        0.068
## 112  pHead pSleep       0.064        0.067
## 113   sTol  pStom       0.000        0.066
## 114   gPre   sEsc       0.057        0.000
## 115   sPre   sPrs       0.057        0.000
## 116  pStom     WB       0.000       -0.057
## 117  pHead  pIrri       0.056        0.000
## 118   gPre   gCfl       0.000        0.000
## 119   gPre   sTol       0.000        0.000
## 120   gPre   sWth       0.000        0.000
## 121   gPre   sPrs       0.000        0.000
## 122   gPre   sPbl       0.000        0.000
## 123   gPre   sDec       0.000        0.000
## 124   gPre   sDis       0.000        0.000
## 125   gPre   sCfl       0.000        0.000
## 126   gPre  pHead       0.000        0.000
## 127   gPre  pStom       0.000        0.000
## 128   gPre  pBack       0.000        0.000
## 129   gPre   pLow       0.000        0.000
## 130   gPre  pIrri       0.000        0.000
## 131   gPre  pNerv       0.000        0.000
## 132   gPre pSleep       0.000        0.000
## 133   gPre pDizzy       0.000        0.000
## 134   gPre     WB       0.000        0.000
## 135   gPre   lone       0.000        0.000
## 136   gTol   gCfl       0.000        0.000
## 137   gTol   sPre       0.000        0.000
## 138   gTol   sPrs       0.000        0.000
## 139   gTol   sEsc       0.000        0.000
## 140   gTol   sPbl       0.000        0.000
## 141   gTol   sDis       0.000        0.000
## 142   gTol   sCfl       0.000        0.000
## 143   gTol  pHead       0.000        0.000
## 144   gTol  pStom       0.000        0.000
## 145   gTol  pBack       0.000        0.000
## 146   gTol   pLow       0.000        0.000
## 147   gTol  pIrri       0.000        0.000
## 148   gTol  pNerv       0.000        0.000
## 149   gTol pSleep       0.000        0.000
## 150   gTol pDizzy       0.000        0.000
## 151   gTol     WB       0.000        0.000
## 152   gTol   lone       0.000        0.000
## 153   gWth   gPrs       0.000        0.000
## 154   gWth   sPre       0.000        0.000
## 155   gWth   sPrs       0.000        0.000
## 156   gWth   sEsc       0.000        0.000
## 157   gWth   sPbl       0.000        0.000
## 158   gWth   sDec       0.000        0.000
## 159   gWth   sDis       0.000        0.000
## 160   gWth   sCfl       0.000        0.000
## 161   gWth  pHead       0.000        0.000
## 162   gWth  pStom       0.000        0.000
## 163   gWth  pBack       0.000        0.000
## 164   gWth   pLow       0.000        0.000
## 165   gWth  pIrri       0.000        0.000
## 166   gWth  pNerv       0.000        0.000
## 167   gWth pSleep       0.000        0.000
## 168   gWth pDizzy       0.000        0.000
## 169   gWth     WB       0.000        0.000
## 170   gWth   lone       0.000        0.000
## 171   gPrs   sPre       0.000        0.000
## 172   gPrs   sTol       0.000        0.000
## 173   gPrs   sWth       0.000        0.000
## 174   gPrs   sEsc       0.000        0.000
## 175   gPrs   sDec       0.000        0.000
## 176   gPrs   sCfl       0.000        0.000
## 177   gPrs  pHead       0.000        0.000
## 178   gPrs  pStom       0.000        0.000
## 179   gPrs  pBack       0.000        0.000
## 180   gPrs   pLow       0.000        0.000
## 181   gPrs  pIrri       0.000        0.000
## 182   gPrs  pNerv       0.000        0.000
## 183   gPrs pSleep       0.000        0.000
## 184   gPrs pDizzy       0.000        0.000
## 185   gPrs     WB       0.000        0.000
## 186   gPrs   lone       0.000        0.000
## 187   gEsc   gCfl       0.000        0.000
## 188   gEsc   sPre       0.000        0.000
## 189   gEsc   sTol       0.000        0.000
## 190   gEsc   sWth       0.000        0.000
## 191   gEsc   sPrs       0.000        0.000
## 192   gEsc   sPbl       0.000        0.000
## 193   gEsc   sDec       0.000        0.000
## 194   gEsc   sDis       0.000        0.000
## 195   gEsc   sCfl       0.000        0.000
## 196   gEsc  pHead       0.000        0.000
## 197   gEsc  pStom       0.000        0.000
## 198   gEsc  pBack       0.000        0.000
## 199   gEsc   pLow       0.000        0.000
## 200   gEsc  pIrri       0.000        0.000
## 201   gEsc  pNerv       0.000        0.000
## 202   gEsc pSleep       0.000        0.000
## 203   gEsc pDizzy       0.000        0.000
## 204   gEsc     WB       0.000        0.000
## 205   gEsc   lone       0.000        0.000
## 206   gPbl   sPre       0.000        0.000
## 207   gPbl   sTol       0.000        0.000
## 208   gPbl   sWth       0.000        0.000
## 209   gPbl   sPrs       0.000        0.000
## 210   gPbl   sEsc       0.000        0.000
## 211   gPbl   sDis       0.000        0.000
## 212   gPbl  pHead       0.000        0.000
## 213   gPbl  pStom       0.000        0.000
## 214   gPbl  pBack       0.000        0.000
## 215   gPbl   pLow       0.000        0.000
## 216   gPbl  pNerv       0.000        0.000
## 217   gPbl pSleep       0.000        0.000
## 218   gPbl pDizzy       0.000        0.000
## 219   gPbl     WB       0.000        0.000
## 220   gPbl   lone       0.000        0.000
## 221   gDec   sPre       0.000        0.000
## 222   gDec   sTol       0.000        0.000
## 223   gDec   sWth       0.000        0.000
## 224   gDec   sPrs       0.000        0.000
## 225   gDec   sEsc       0.000        0.000
## 226   gDec   sDis       0.000        0.000
## 227   gDec   sCfl       0.000        0.000
## 228   gDec  pHead       0.000        0.000
## 229   gDec  pStom       0.000        0.000
## 230   gDec  pBack       0.000        0.000
## 231   gDec   pLow       0.000        0.000
## 232   gDec  pIrri       0.000        0.000
## 233   gDec  pNerv       0.000        0.000
## 234   gDec pSleep       0.000        0.000
## 235   gDec pDizzy       0.000        0.000
## 236   gDec     WB       0.000        0.000
## 237   gDec   lone       0.000        0.000
## 238   gDis   sWth       0.000        0.000
## 239   gDis   sPrs       0.000        0.000
## 240   gDis   sEsc       0.000        0.000
## 241   gDis   sPbl       0.000        0.000
## 242   gDis   sDec       0.000        0.000
## 243   gDis   sCfl       0.000        0.000
## 244   gDis  pHead       0.000        0.000
## 245   gDis  pStom       0.000        0.000
## 246   gDis  pBack       0.000        0.000
## 247   gDis   pLow       0.000        0.000
## 248   gDis  pIrri       0.000        0.000
## 249   gDis  pNerv       0.000        0.000
## 250   gDis pSleep       0.000        0.000
## 251   gDis pDizzy       0.000        0.000
## 252   gDis   lone       0.000        0.000
## 253   gCfl   sPre       0.000        0.000
## 254   gCfl   sTol       0.000        0.000
## 255   gCfl   sPrs       0.000        0.000
## 256   gCfl   sEsc       0.000        0.000
## 257   gCfl   sDec       0.000        0.000
## 258   gCfl   sDis       0.000        0.000
## 259   gCfl  pHead       0.000        0.000
## 260   gCfl  pStom       0.000        0.000
## 261   gCfl  pBack       0.000        0.000
## 262   gCfl   pLow       0.000        0.000
## 263   gCfl  pIrri       0.000        0.000
## 264   gCfl  pNerv       0.000        0.000
## 265   gCfl pSleep       0.000        0.000
## 266   gCfl pDizzy       0.000        0.000
## 267   gCfl     WB       0.000        0.000
## 268   gCfl   lone       0.000        0.000
## 269   sPre  pHead       0.000        0.000
## 270   sPre  pStom       0.000        0.000
## 271   sPre  pBack       0.000        0.000
## 272   sPre   pLow       0.000        0.000
## 273   sPre  pIrri       0.000        0.000
## 274   sPre  pNerv       0.000        0.000
## 275   sPre pSleep       0.000        0.000
## 276   sPre pDizzy       0.000        0.000
## 277   sPre     WB       0.000        0.000
## 278   sPre   lone       0.000        0.000
## 279   sTol   sCfl       0.000        0.000
## 280   sTol  pHead       0.000        0.000
## 281   sTol  pBack       0.000        0.000
## 282   sTol   pLow       0.000        0.000
## 283   sTol  pIrri       0.000        0.000
## 284   sTol  pNerv       0.000        0.000
## 285   sTol pSleep       0.000        0.000
## 286   sTol pDizzy       0.000        0.000
## 287   sTol     WB       0.000        0.000
## 288   sTol   lone       0.000        0.000
## 289   sWth   sDec       0.000        0.000
## 290   sWth  pHead       0.000        0.000
## 291   sWth  pStom       0.000        0.000
## 292   sWth  pBack       0.000        0.000
## 293   sWth   pLow       0.000        0.000
## 294   sWth  pIrri       0.000        0.000
## 295   sWth  pNerv       0.000        0.000
## 296   sWth pSleep       0.000        0.000
## 297   sWth pDizzy       0.000        0.000
## 298   sWth     WB       0.000        0.000
## 299   sWth   lone       0.000        0.000
## 300   sPrs   sCfl       0.000        0.000
## 301   sPrs  pHead       0.000        0.000
## 302   sPrs  pStom       0.000        0.000
## 303   sPrs  pBack       0.000        0.000
## 304   sPrs   pLow       0.000        0.000
## 305   sPrs  pIrri       0.000        0.000
## 306   sPrs  pNerv       0.000        0.000
## 307   sPrs pSleep       0.000        0.000
## 308   sPrs pDizzy       0.000        0.000
## 309   sPrs     WB       0.000        0.000
## 310   sPrs   lone       0.000        0.000
## 311   sEsc   sCfl       0.000        0.000
## 312   sEsc  pHead       0.000        0.000
## 313   sEsc  pStom       0.000        0.000
## 314   sEsc  pBack       0.000        0.000
## 315   sEsc  pIrri       0.000        0.000
## 316   sEsc pSleep       0.000        0.000
## 317   sEsc pDizzy       0.000        0.000
## 318   sPbl  pHead       0.000        0.000
## 319   sPbl  pStom       0.000        0.000
## 320   sPbl  pBack       0.000        0.000
## 321   sPbl   pLow       0.000        0.000
## 322   sPbl  pIrri       0.000        0.000
## 323   sPbl  pNerv       0.000        0.000
## 324   sPbl pSleep       0.000        0.000
## 325   sPbl pDizzy       0.000        0.000
## 326   sPbl     WB       0.000        0.000
## 327   sPbl   lone       0.000        0.000
## 328   sDec  pHead       0.000        0.000
## 329   sDec  pStom       0.000        0.000
## 330   sDec  pBack       0.000        0.000
## 331   sDec   pLow       0.000        0.000
## 332   sDec  pIrri       0.000        0.000
## 333   sDec  pNerv       0.000        0.000
## 334   sDec pSleep       0.000        0.000
## 335   sDec pDizzy       0.000        0.000
## 336   sDec     WB       0.000        0.000
## 337   sDec   lone       0.000        0.000
## 338   sDis  pHead       0.000        0.000
## 339   sDis  pStom       0.000        0.000
## 340   sDis  pBack       0.000        0.000
## 341   sDis   pLow       0.000        0.000
## 342   sDis  pIrri       0.000        0.000
## 343   sDis  pNerv       0.000        0.000
## 344   sDis pSleep       0.000        0.000
## 345   sDis pDizzy       0.000        0.000
## 346   sDis     WB       0.000        0.000
## 347   sDis   lone       0.000        0.000
## 348   sCfl  pHead       0.000        0.000
## 349   sCfl  pStom       0.000        0.000
## 350   sCfl  pBack       0.000        0.000
## 351   sCfl   pLow       0.000        0.000
## 352   sCfl  pIrri       0.000        0.000
## 353   sCfl  pNerv       0.000        0.000
## 354   sCfl pSleep       0.000        0.000
## 355   sCfl pDizzy       0.000        0.000
## 356   sCfl     WB       0.000        0.000
## 357   sCfl   lone       0.000        0.000
## 358  pHead  pNerv       0.000        0.000
## 359  pHead     WB       0.000        0.000
## 360  pHead   lone       0.000        0.000
## 361  pStom   pLow       0.000        0.000
## 362  pStom pSleep       0.000        0.000
## 363  pStom   lone       0.000        0.000
## 364  pBack   pLow       0.000        0.000
## 365  pBack  pIrri       0.000        0.000
## 366  pBack  pNerv       0.000        0.000
## 367  pBack pSleep       0.000        0.000
## 368  pBack pDizzy       0.000        0.000
## 369  pBack     WB       0.000        0.000
## 370  pBack   lone       0.000        0.000
## 371   pLow pSleep       0.000        0.000
## 372  pIrri pDizzy       0.000        0.000
## 373  pIrri     WB       0.000        0.000
## 374  pIrri   lone       0.000        0.000
## 375  pNerv     WB       0.000        0.000
## 376 pSleep   lone       0.000        0.000
## 377 pDizzy     WB       0.000        0.000
## 378 pDizzy   lone       0.000        0.000
```

#### Centrality

**The centrality estimates are relatively stable when the
correlation stability coefficient (CS coefficient) is ≥ 0.5**
while set at correlation stability = 0.7 (Epskamp et al.,
2018). Centrality indices with CS coefficient of 0.25-0.5 should be
interpreted with caution; CS coefficient < 0.25 indicated large
instability of the centrality index.

```
##   group        centrality    CS_r07
## 1  Boys          strength 0.7500149
## 2  Boys         closeness 0.7500149
## 3  Boys       betweenness 0.7500149
## 4  Boys expectedInfluence 0.7500149
## 5 Girls          strength 0.7500149
## 6 Girls         closeness 0.7500149
## 7 Girls       betweenness 0.7500149
## 8 Girls expectedInfluence 0.7500149
```

#### Bootstrapped Edges

### IS

#### Figure

#### Edges

```
##       From     To Weight_Boys Weight_Girls
## 1     gPre   gWth       0.289        0.857
## 2     gPbl   gDis       0.341        0.833
## 3     gWth   gCfl       0.527        0.780
## 4     sPbl   sCfl       0.731        0.473
## 5     sTol   sWth       0.555        0.684
## 6     sPre   sTol       0.649        0.421
## 7     sDec   sCfl       0.583        0.428
## 8     sEsc   sPbl       0.571        0.239
## 9     gTol   gDis       0.000        0.562
## 10    gTol   gEsc       0.000        0.539
## 11    gWth   gDec       0.130        0.526
## 12    sPre   sDis       0.512        0.244
## 13    gCfl   sCfl       0.497        0.000
## 14    gPbl   gCfl       0.134        0.489
## 15    gPrs   gDis       0.000        0.487
## 16    gPre   gEsc       0.357        0.481
## 17    sPre   sWth       0.381        0.468
## 18    gTol   gWth       0.450        0.425
## 19    gPre   gTol       0.449        0.334
## 20    gEsc   sEsc       0.439        0.405
## 21    gTol   gPbl       0.436        0.000
## 22    gDec   gDis       0.434        0.132
## 23    gWth   gEsc       0.428        0.000
## 24    gDec   gCfl       0.423        0.000
## 25    gWth   sWth       0.410        0.000
## 26    sDis   sCfl       0.387        0.404
## 27    gPbl   gDec       0.210        0.403
## 28    gDis   sTol       0.000        0.396
## 29    gPrs   gPbl       0.266        0.393
## 30    gWth   gDis       0.389        0.000
## 31    sPbl   sDec       0.212        0.377
## 32    gTol   gCfl       0.373        0.000
## 33    sPre   sCfl       0.355        0.000
## 34    sPrs   sEsc       0.300        0.354
## 35    gTol   sTol       0.348        0.209
## 36    sDec   sDis       0.339        0.250
## 37    gPre   gDec       0.185        0.338
## 38    sWth   sPbl       0.332        0.185
## 39    sTol   sPbl       0.330        0.000
## 40    sPre   sPrs       0.323        0.274
## 41    sTol   sDis       0.179        0.306
## 42    sPre   sEsc       0.295        0.214
## 43    gPre   gPbl       0.292        0.000
## 44    gDis   sPbl       0.000        0.287
## 45    gDis   gCfl       0.282        0.000
## 46    gDec   sDec       0.275        0.000
## 47    pLow  pIrri       0.193        0.269
## 48   pHead pDizzy       0.215        0.263
## 49    pLow  pNerv       0.254        0.214
## 50    pLow   lone       0.247        0.199
## 51    gDec   sPre       0.238        0.000
## 52    sPbl   sDis       0.237        0.235
## 53   pHead  pStom       0.237        0.181
## 54   pIrri  pNerv       0.103        0.236
## 55    gDec   sPbl       0.231        0.000
## 56    gEsc   gPbl       0.216        0.000
## 57    sPre   sDec       0.000        0.216
## 58    sPrs   sDec       0.206        0.159
## 59    sWth   sPrs       0.202        0.000
## 60    sEsc   sDec       0.202        0.000
## 61    pLow     WB      -0.197       -0.163
## 62    gPrs   gDec       0.191        0.000
## 63    sWth   sDec       0.183        0.000
## 64      WB   lone      -0.158       -0.180
## 65    gTol   gDec       0.177        0.000
## 66    gTol   gPrs       0.171        0.000
## 67    sWth   sCfl       0.000        0.168
## 68    gPre   gDis       0.166        0.000
## 69    sPrs   sPbl       0.166        0.000
## 70    gDis   sDis       0.000        0.164
## 71    sWth   sEsc       0.158        0.000
## 72    gPbl   sEsc       0.152        0.000
## 73    pLow pSleep       0.152        0.000
## 74    gEsc   gDec       0.149        0.000
## 75  pSleep pDizzy       0.000        0.149
## 76    sTol   sEsc       0.101        0.147
## 77    gWth   gPrs       0.143        0.000
## 78    gEsc     WB      -0.139        0.000
## 79    gPrs   gEsc       0.110        0.138
## 80    sTol   sDec       0.115        0.135
## 81   pNerv pDizzy       0.132        0.000
## 82  pSleep     WB      -0.127       -0.097
## 83    sTol   sCfl       0.000        0.123
## 84   pStom  pBack       0.000        0.121
## 85    gEsc   sCfl       0.000        0.116
## 86    pLow pDizzy       0.000        0.116
## 87    gTol   sPbl       0.108        0.000
## 88   pBack pDizzy       0.068        0.104
## 89    sPre   sPbl       0.000        0.102
## 90   pHead  pBack       0.101        0.095
## 91    sEsc     WB       0.000       -0.097
## 92   pBack pSleep       0.096        0.000
## 93   pStom  pNerv       0.087        0.000
## 94    gPre   gPrs       0.085        0.000
## 95   pStom pDizzy       0.083        0.084
## 96   pStom   pLow       0.083        0.000
## 97    gPre     WB      -0.066        0.000
## 98    gPre   gCfl       0.000        0.000
## 99    gPre   sPre       0.000        0.000
## 100   gPre   sTol       0.000        0.000
## 101   gPre   sWth       0.000        0.000
## 102   gPre   sPrs       0.000        0.000
## 103   gPre   sEsc       0.000        0.000
## 104   gPre   sPbl       0.000        0.000
## 105   gPre   sDec       0.000        0.000
## 106   gPre   sDis       0.000        0.000
## 107   gPre   sCfl       0.000        0.000
## 108   gPre  pHead       0.000        0.000
## 109   gPre  pStom       0.000        0.000
## 110   gPre  pBack       0.000        0.000
## 111   gPre   pLow       0.000        0.000
## 112   gPre  pIrri       0.000        0.000
## 113   gPre  pNerv       0.000        0.000
## 114   gPre pSleep       0.000        0.000
## 115   gPre pDizzy       0.000        0.000
## 116   gPre   lone       0.000        0.000
## 117   gTol   sPre       0.000        0.000
## 118   gTol   sWth       0.000        0.000
## 119   gTol   sPrs       0.000        0.000
## 120   gTol   sEsc       0.000        0.000
## 121   gTol   sDec       0.000        0.000
## 122   gTol   sDis       0.000        0.000
## 123   gTol   sCfl       0.000        0.000
## 124   gTol  pHead       0.000        0.000
## 125   gTol  pStom       0.000        0.000
## 126   gTol  pBack       0.000        0.000
## 127   gTol   pLow       0.000        0.000
## 128   gTol  pIrri       0.000        0.000
## 129   gTol  pNerv       0.000        0.000
## 130   gTol pSleep       0.000        0.000
## 131   gTol pDizzy       0.000        0.000
## 132   gTol     WB       0.000        0.000
## 133   gTol   lone       0.000        0.000
## 134   gWth   gPbl       0.000        0.000
## 135   gWth   sPre       0.000        0.000
## 136   gWth   sTol       0.000        0.000
## 137   gWth   sPrs       0.000        0.000
## 138   gWth   sEsc       0.000        0.000
## 139   gWth   sPbl       0.000        0.000
## 140   gWth   sDec       0.000        0.000
## 141   gWth   sDis       0.000        0.000
## 142   gWth   sCfl       0.000        0.000
## 143   gWth  pHead       0.000        0.000
## 144   gWth  pStom       0.000        0.000
## 145   gWth  pBack       0.000        0.000
## 146   gWth   pLow       0.000        0.000
## 147   gWth  pIrri       0.000        0.000
## 148   gWth  pNerv       0.000        0.000
## 149   gWth pSleep       0.000        0.000
## 150   gWth pDizzy       0.000        0.000
## 151   gWth     WB       0.000        0.000
## 152   gWth   lone       0.000        0.000
## 153   gPrs   gCfl       0.000        0.000
## 154   gPrs   sPre       0.000        0.000
## 155   gPrs   sTol       0.000        0.000
## 156   gPrs   sWth       0.000        0.000
## 157   gPrs   sPrs       0.000        0.000
## 158   gPrs   sEsc       0.000        0.000
## 159   gPrs   sPbl       0.000        0.000
## 160   gPrs   sDec       0.000        0.000
## 161   gPrs   sDis       0.000        0.000
## 162   gPrs   sCfl       0.000        0.000
## 163   gPrs  pHead       0.000        0.000
## 164   gPrs  pStom       0.000        0.000
## 165   gPrs  pBack       0.000        0.000
## 166   gPrs   pLow       0.000        0.000
## 167   gPrs  pIrri       0.000        0.000
## 168   gPrs  pNerv       0.000        0.000
## 169   gPrs pSleep       0.000        0.000
## 170   gPrs pDizzy       0.000        0.000
## 171   gPrs     WB       0.000        0.000
## 172   gPrs   lone       0.000        0.000
## 173   gEsc   gDis       0.000        0.000
## 174   gEsc   gCfl       0.000        0.000
## 175   gEsc   sPre       0.000        0.000
## 176   gEsc   sTol       0.000        0.000
## 177   gEsc   sWth       0.000        0.000
## 178   gEsc   sPrs       0.000        0.000
## 179   gEsc   sPbl       0.000        0.000
## 180   gEsc   sDec       0.000        0.000
## 181   gEsc   sDis       0.000        0.000
## 182   gEsc  pHead       0.000        0.000
## 183   gEsc  pStom       0.000        0.000
## 184   gEsc  pBack       0.000        0.000
## 185   gEsc   pLow       0.000        0.000
## 186   gEsc  pIrri       0.000        0.000
## 187   gEsc  pNerv       0.000        0.000
## 188   gEsc pSleep       0.000        0.000
## 189   gEsc pDizzy       0.000        0.000
## 190   gEsc   lone       0.000        0.000
## 191   gPbl   sPre       0.000        0.000
## 192   gPbl   sTol       0.000        0.000
## 193   gPbl   sWth       0.000        0.000
## 194   gPbl   sPrs       0.000        0.000
## 195   gPbl   sPbl       0.000        0.000
## 196   gPbl   sDec       0.000        0.000
## 197   gPbl   sDis       0.000        0.000
## 198   gPbl   sCfl       0.000        0.000
## 199   gPbl  pHead       0.000        0.000
## 200   gPbl  pStom       0.000        0.000
## 201   gPbl  pBack       0.000        0.000
## 202   gPbl   pLow       0.000        0.000
## 203   gPbl  pIrri       0.000        0.000
## 204   gPbl  pNerv       0.000        0.000
## 205   gPbl pSleep       0.000        0.000
## 206   gPbl pDizzy       0.000        0.000
## 207   gPbl     WB       0.000        0.000
## 208   gPbl   lone       0.000        0.000
## 209   gDec   sTol       0.000        0.000
## 210   gDec   sWth       0.000        0.000
## 211   gDec   sPrs       0.000        0.000
## 212   gDec   sEsc       0.000        0.000
## 213   gDec   sDis       0.000        0.000
## 214   gDec   sCfl       0.000        0.000
## 215   gDec  pHead       0.000        0.000
## 216   gDec  pStom       0.000        0.000
## 217   gDec  pBack       0.000        0.000
## 218   gDec   pLow       0.000        0.000
## 219   gDec  pIrri       0.000        0.000
## 220   gDec  pNerv       0.000        0.000
## 221   gDec pSleep       0.000        0.000
## 222   gDec pDizzy       0.000        0.000
## 223   gDec     WB       0.000        0.000
## 224   gDec   lone       0.000        0.000
## 225   gDis   sPre       0.000        0.000
## 226   gDis   sWth       0.000        0.000
## 227   gDis   sPrs       0.000        0.000
## 228   gDis   sEsc       0.000        0.000
## 229   gDis   sDec       0.000        0.000
## 230   gDis   sCfl       0.000        0.000
## 231   gDis  pHead       0.000        0.000
## 232   gDis  pStom       0.000        0.000
## 233   gDis  pBack       0.000        0.000
## 234   gDis   pLow       0.000        0.000
## 235   gDis  pIrri       0.000        0.000
## 236   gDis  pNerv       0.000        0.000
## 237   gDis pSleep       0.000        0.000
## 238   gDis pDizzy       0.000        0.000
## 239   gDis     WB       0.000        0.000
## 240   gDis   lone       0.000        0.000
## 241   gCfl   sPre       0.000        0.000
## 242   gCfl   sTol       0.000        0.000
## 243   gCfl   sWth       0.000        0.000
## 244   gCfl   sPrs       0.000        0.000
## 245   gCfl   sEsc       0.000        0.000
## 246   gCfl   sPbl       0.000        0.000
## 247   gCfl   sDec       0.000        0.000
## 248   gCfl   sDis       0.000        0.000
## 249   gCfl  pHead       0.000        0.000
## 250   gCfl  pStom       0.000        0.000
## 251   gCfl  pBack       0.000        0.000
## 252   gCfl   pLow       0.000        0.000
## 253   gCfl  pIrri       0.000        0.000
## 254   gCfl  pNerv       0.000        0.000
## 255   gCfl pSleep       0.000        0.000
## 256   gCfl pDizzy       0.000        0.000
## 257   gCfl     WB       0.000        0.000
## 258   gCfl   lone       0.000        0.000
## 259   sPre  pHead       0.000        0.000
## 260   sPre  pStom       0.000        0.000
## 261   sPre  pBack       0.000        0.000
## 262   sPre   pLow       0.000        0.000
## 263   sPre  pIrri       0.000        0.000
## 264   sPre  pNerv       0.000        0.000
## 265   sPre pSleep       0.000        0.000
## 266   sPre pDizzy       0.000        0.000
## 267   sPre     WB       0.000        0.000
## 268   sPre   lone       0.000        0.000
## 269   sTol   sPrs       0.000        0.000
## 270   sTol  pHead       0.000        0.000
## 271   sTol  pStom       0.000        0.000
## 272   sTol  pBack       0.000        0.000
## 273   sTol   pLow       0.000        0.000
## 274   sTol  pIrri       0.000        0.000
## 275   sTol  pNerv       0.000        0.000
## 276   sTol pSleep       0.000        0.000
## 277   sTol pDizzy       0.000        0.000
## 278   sTol     WB       0.000        0.000
## 279   sTol   lone       0.000        0.000
## 280   sWth   sDis       0.000        0.000
## 281   sWth  pHead       0.000        0.000
## 282   sWth  pStom       0.000        0.000
## 283   sWth  pBack       0.000        0.000
## 284   sWth   pLow       0.000        0.000
## 285   sWth  pIrri       0.000        0.000
## 286   sWth  pNerv       0.000        0.000
## 287   sWth pSleep       0.000        0.000
## 288   sWth pDizzy       0.000        0.000
## 289   sWth     WB       0.000        0.000
## 290   sWth   lone       0.000        0.000
## 291   sPrs   sDis       0.000        0.000
## 292   sPrs   sCfl       0.000        0.000
## 293   sPrs  pHead       0.000        0.000
## 294   sPrs  pStom       0.000        0.000
## 295   sPrs  pBack       0.000        0.000
## 296   sPrs   pLow       0.000        0.000
## 297   sPrs  pIrri       0.000        0.000
## 298   sPrs  pNerv       0.000        0.000
## 299   sPrs pSleep       0.000        0.000
## 300   sPrs pDizzy       0.000        0.000
## 301   sPrs     WB       0.000        0.000
## 302   sPrs   lone       0.000        0.000
## 303   sEsc   sDis       0.000        0.000
## 304   sEsc   sCfl       0.000        0.000
## 305   sEsc  pHead       0.000        0.000
## 306   sEsc  pStom       0.000        0.000
## 307   sEsc  pBack       0.000        0.000
## 308   sEsc   pLow       0.000        0.000
## 309   sEsc  pIrri       0.000        0.000
## 310   sEsc  pNerv       0.000        0.000
## 311   sEsc pSleep       0.000        0.000
## 312   sEsc pDizzy       0.000        0.000
## 313   sEsc   lone       0.000        0.000
## 314   sPbl  pHead       0.000        0.000
## 315   sPbl  pStom       0.000        0.000
## 316   sPbl  pBack       0.000        0.000
## 317   sPbl   pLow       0.000        0.000
## 318   sPbl  pIrri       0.000        0.000
## 319   sPbl  pNerv       0.000        0.000
## 320   sPbl pSleep       0.000        0.000
## 321   sPbl pDizzy       0.000        0.000
## 322   sPbl     WB       0.000        0.000
## 323   sPbl   lone       0.000        0.000
## 324   sDec  pHead       0.000        0.000
## 325   sDec  pStom       0.000        0.000
## 326   sDec  pBack       0.000        0.000
## 327   sDec   pLow       0.000        0.000
## 328   sDec  pIrri       0.000        0.000
## 329   sDec  pNerv       0.000        0.000
## 330   sDec pSleep       0.000        0.000
## 331   sDec pDizzy       0.000        0.000
## 332   sDec     WB       0.000        0.000
## 333   sDec   lone       0.000        0.000
## 334   sDis  pHead       0.000        0.000
## 335   sDis  pStom       0.000        0.000
## 336   sDis  pBack       0.000        0.000
## 337   sDis   pLow       0.000        0.000
## 338   sDis  pIrri       0.000        0.000
## 339   sDis  pNerv       0.000        0.000
## 340   sDis pSleep       0.000        0.000
## 341   sDis pDizzy       0.000        0.000
## 342   sDis     WB       0.000        0.000
## 343   sDis   lone       0.000        0.000
## 344   sCfl  pHead       0.000        0.000
## 345   sCfl  pStom       0.000        0.000
## 346   sCfl  pBack       0.000        0.000
## 347   sCfl   pLow       0.000        0.000
## 348   sCfl  pIrri       0.000        0.000
## 349   sCfl  pNerv       0.000        0.000
## 350   sCfl pSleep       0.000        0.000
## 351   sCfl pDizzy       0.000        0.000
## 352   sCfl     WB       0.000        0.000
## 353   sCfl   lone       0.000        0.000
## 354  pHead   pLow       0.000        0.000
## 355  pHead  pIrri       0.000        0.000
## 356  pHead  pNerv       0.000        0.000
## 357  pHead pSleep       0.000        0.000
## 358  pHead     WB       0.000        0.000
## 359  pHead   lone       0.000        0.000
## 360  pStom  pIrri       0.000        0.000
## 361  pStom pSleep       0.000        0.000
## 362  pStom     WB       0.000        0.000
## 363  pStom   lone       0.000        0.000
## 364  pBack   pLow       0.000        0.000
## 365  pBack  pIrri       0.000        0.000
## 366  pBack  pNerv       0.000        0.000
## 367  pBack     WB       0.000        0.000
## 368  pBack   lone       0.000        0.000
## 369  pIrri pSleep       0.000        0.000
## 370  pIrri pDizzy       0.000        0.000
## 371  pIrri     WB       0.000        0.000
## 372  pIrri   lone       0.000        0.000
## 373  pNerv pSleep       0.000        0.000
## 374  pNerv     WB       0.000        0.000
## 375  pNerv   lone       0.000        0.000
## 376 pSleep   lone       0.000        0.000
## 377 pDizzy     WB       0.000        0.000
## 378 pDizzy   lone       0.000        0.000
```

#### Centrality

**The centrality estimates are relatively stable when the
correlation stability coefficient (CS coefficient) is ≥ 0.5**
while set at correlation stability = 0.7 (Epskamp et al.,
2018). Centrality indices with CS coefficient of 0.25-0.5 should be
interpreted with caution; CS coefficient < 0.25 indicated large
instability of the centrality index.

```
##   group        centrality    CS_r07
## 1  Boys          strength 0.7500149
## 2  Boys         closeness 0.7500149
## 3  Boys       betweenness 0.7500149
## 4  Boys expectedInfluence 0.7500149
## 5 Girls          strength 0.7500149
## 6 Girls         closeness 0.7500149
## 7 Girls       betweenness 0.7500149
## 8 Girls expectedInfluence 0.7500149
```

#### Bootstrapped Edges

### MK

#### Figure

#### Edges

```
##       From     To Weight_Boys Weight_Girls
## 1     gPbl   gCfl       0.256        0.818
## 2     gPre   gTol       0.293        0.573
## 3     sTol   sWth       0.201        0.541
## 4     gDis   gCfl       0.127        0.533
## 5     sDec   sCfl       0.527        0.469
## 6     gTol   gWth       0.401        0.490
## 7     gPbl   gDis       0.417        0.486
## 8     gDec   gCfl       0.481        0.316
## 9     gDec   gDis       0.300        0.477
## 10    sPbl   sCfl       0.391        0.449
## 11    sPre   sWth       0.423        0.172
## 12    sTol   sPbl       0.405        0.000
## 13    sPre   sTol       0.380        0.394
## 14    gPre   gWth       0.389        0.183
## 15    gPbl   gDec       0.000        0.382
## 16    gDec   sPbl       0.364        0.000
## 17    gCfl   sCfl       0.360        0.261
## 18    gPrs   gPbl       0.136        0.357
## 19    pLow  pNerv       0.242        0.357
## 20    gTol   gEsc       0.000        0.352
## 21    gWth   gDis       0.345        0.235
## 22    sPbl   sDec       0.000        0.344
## 23    gDec   sDec       0.340        0.223
## 24    sWth   sDis       0.337        0.123
## 25    gEsc   sEsc       0.327        0.246
## 26    gTol   gPrs       0.272        0.325
## 27    gPrs   gDis       0.231        0.322
## 28    sDec   sDis       0.319        0.131
## 29    gPre   gEsc       0.290        0.313
## 30    sWth   sEsc       0.172        0.296
## 31   pIrri  pNerv       0.292        0.182
## 32    gWth   gDec       0.235        0.286
## 33    gWth   gCfl       0.269        0.000
## 34    gPre   gDis       0.000        0.260
## 35      WB   lone      -0.211       -0.256
## 36    gCfl   sPbl       0.250        0.000
## 37    gPbl   sPbl       0.243        0.000
## 38    sWth   sCfl       0.239        0.000
## 39    gWth   sTol       0.228        0.000
## 40    sPbl   sDis       0.000        0.225
## 41    sTol   sCfl       0.224        0.000
## 42    sPre   sEsc       0.000        0.219
## 43    pLow   lone       0.217        0.159
## 44    gPrs   sDec       0.213        0.000
## 45    gDis   sDis       0.211        0.185
## 46    sPre   sCfl       0.209        0.102
## 47    gPbl   sDec       0.207        0.000
## 48    sPrs   sEsc       0.198        0.205
## 49   pHead pDizzy       0.203        0.179
## 50    pLow  pIrri       0.148        0.198
## 51    pLow     WB      -0.177       -0.196
## 52    sPrs   sDec       0.192        0.000
## 53   pStom pDizzy       0.189        0.000
## 54    sPre   sDis       0.000        0.179
## 55    gTol   gDis       0.000        0.178
## 56    gPrs   gDec       0.171        0.000
## 57    sEsc     WB       0.000       -0.168
## 58   pHead  pStom       0.000        0.165
## 59    gPre   gPrs       0.122        0.164
## 60    gWth   gEsc       0.000        0.164
## 61    sWth   sDec       0.164        0.077
## 62    gEsc   gPbl       0.000        0.160
## 63    sPre   sDec       0.160        0.000
## 64    gTol   gPbl       0.158        0.103
## 65    gEsc   gDec       0.125        0.158
## 66  pSleep pDizzy       0.121        0.150
## 67    gTol   sPbl       0.000        0.149
## 68   pHead   pLow       0.116        0.146
## 69   pStom  pBack       0.145        0.000
## 70   pBack   pLow       0.144        0.000
## 71    gTol   gDec       0.107        0.143
## 72    gWth   sDec       0.000        0.140
## 73    gWth   sWth       0.135        0.000
## 74   pNerv pSleep       0.132        0.122
## 75    gDis   sWth       0.123        0.000
## 76    gEsc   lone       0.000        0.122
## 77    sTol   sPrs       0.119        0.000
## 78    sPre   sPrs       0.000        0.111
## 79    sTol   sEsc       0.109        0.000
## 80    sEsc   pLow       0.072        0.107
## 81  pSleep     WB      -0.106        0.000
## 82    sCfl   lone       0.000        0.105
## 83    gDec   sCfl       0.000        0.100
## 84    sDis   sCfl       0.083        0.098
## 85    pLow pSleep       0.098        0.000
## 86   pBack pDizzy       0.096        0.000
## 87    gEsc   gDis       0.000        0.091
## 88   pHead  pNerv       0.091        0.000
## 89    gPbl   sDis       0.087        0.000
## 90   pHead pSleep       0.087        0.000
## 91   pIrri     WB       0.000       -0.085
## 92   pBack  pIrri       0.083        0.000
## 93    sEsc  pNerv       0.000        0.071
## 94    gPre   gPbl       0.000        0.000
## 95    gPre   gDec       0.000        0.000
## 96    gPre   gCfl       0.000        0.000
## 97    gPre   sPre       0.000        0.000
## 98    gPre   sTol       0.000        0.000
## 99    gPre   sWth       0.000        0.000
## 100   gPre   sPrs       0.000        0.000
## 101   gPre   sEsc       0.000        0.000
## 102   gPre   sPbl       0.000        0.000
## 103   gPre   sDec       0.000        0.000
## 104   gPre   sDis       0.000        0.000
## 105   gPre   sCfl       0.000        0.000
## 106   gPre  pHead       0.000        0.000
## 107   gPre  pStom       0.000        0.000
## 108   gPre  pBack       0.000        0.000
## 109   gPre   pLow       0.000        0.000
## 110   gPre  pIrri       0.000        0.000
## 111   gPre  pNerv       0.000        0.000
## 112   gPre pSleep       0.000        0.000
## 113   gPre pDizzy       0.000        0.000
## 114   gPre     WB       0.000        0.000
## 115   gPre   lone       0.000        0.000
## 116   gTol   gCfl       0.000        0.000
## 117   gTol   sPre       0.000        0.000
## 118   gTol   sTol       0.000        0.000
## 119   gTol   sWth       0.000        0.000
## 120   gTol   sPrs       0.000        0.000
## 121   gTol   sEsc       0.000        0.000
## 122   gTol   sDec       0.000        0.000
## 123   gTol   sDis       0.000        0.000
## 124   gTol   sCfl       0.000        0.000
## 125   gTol  pHead       0.000        0.000
## 126   gTol  pStom       0.000        0.000
## 127   gTol  pBack       0.000        0.000
## 128   gTol   pLow       0.000        0.000
## 129   gTol  pIrri       0.000        0.000
## 130   gTol  pNerv       0.000        0.000
## 131   gTol pSleep       0.000        0.000
## 132   gTol pDizzy       0.000        0.000
## 133   gTol     WB       0.000        0.000
## 134   gTol   lone       0.000        0.000
## 135   gWth   gPrs       0.000        0.000
## 136   gWth   gPbl       0.000        0.000
## 137   gWth   sPre       0.000        0.000
## 138   gWth   sPrs       0.000        0.000
## 139   gWth   sEsc       0.000        0.000
## 140   gWth   sPbl       0.000        0.000
## 141   gWth   sDis       0.000        0.000
## 142   gWth   sCfl       0.000        0.000
## 143   gWth  pHead       0.000        0.000
## 144   gWth  pStom       0.000        0.000
## 145   gWth  pBack       0.000        0.000
## 146   gWth   pLow       0.000        0.000
## 147   gWth  pIrri       0.000        0.000
## 148   gWth  pNerv       0.000        0.000
## 149   gWth pSleep       0.000        0.000
## 150   gWth pDizzy       0.000        0.000
## 151   gWth     WB       0.000        0.000
## 152   gWth   lone       0.000        0.000
## 153   gPrs   gEsc       0.000        0.000
## 154   gPrs   gCfl       0.000        0.000
## 155   gPrs   sPre       0.000        0.000
## 156   gPrs   sTol       0.000        0.000
## 157   gPrs   sWth       0.000        0.000
## 158   gPrs   sPrs       0.000        0.000
## 159   gPrs   sEsc       0.000        0.000
## 160   gPrs   sPbl       0.000        0.000
## 161   gPrs   sDis       0.000        0.000
## 162   gPrs   sCfl       0.000        0.000
## 163   gPrs  pHead       0.000        0.000
## 164   gPrs  pStom       0.000        0.000
## 165   gPrs  pBack       0.000        0.000
## 166   gPrs   pLow       0.000        0.000
## 167   gPrs  pIrri       0.000        0.000
## 168   gPrs  pNerv       0.000        0.000
## 169   gPrs pSleep       0.000        0.000
## 170   gPrs pDizzy       0.000        0.000
## 171   gPrs     WB       0.000        0.000
## 172   gPrs   lone       0.000        0.000
## 173   gEsc   gCfl       0.000        0.000
## 174   gEsc   sPre       0.000        0.000
## 175   gEsc   sTol       0.000        0.000
## 176   gEsc   sWth       0.000        0.000
## 177   gEsc   sPrs       0.000        0.000
## 178   gEsc   sPbl       0.000        0.000
## 179   gEsc   sDec       0.000        0.000
## 180   gEsc   sDis       0.000        0.000
## 181   gEsc   sCfl       0.000        0.000
## 182   gEsc  pHead       0.000        0.000
## 183   gEsc  pStom       0.000        0.000
## 184   gEsc  pBack       0.000        0.000
## 185   gEsc   pLow       0.000        0.000
## 186   gEsc  pIrri       0.000        0.000
## 187   gEsc  pNerv       0.000        0.000
## 188   gEsc pSleep       0.000        0.000
## 189   gEsc pDizzy       0.000        0.000
## 190   gEsc     WB       0.000        0.000
## 191   gPbl   sPre       0.000        0.000
## 192   gPbl   sTol       0.000        0.000
## 193   gPbl   sWth       0.000        0.000
## 194   gPbl   sPrs       0.000        0.000
## 195   gPbl   sEsc       0.000        0.000
## 196   gPbl   sCfl       0.000        0.000
## 197   gPbl  pHead       0.000        0.000
## 198   gPbl  pStom       0.000        0.000
## 199   gPbl  pBack       0.000        0.000
## 200   gPbl   pLow       0.000        0.000
## 201   gPbl  pIrri       0.000        0.000
## 202   gPbl  pNerv       0.000        0.000
## 203   gPbl pSleep       0.000        0.000
## 204   gPbl pDizzy       0.000        0.000
## 205   gPbl     WB       0.000        0.000
## 206   gPbl   lone       0.000        0.000
## 207   gDec   sPre       0.000        0.000
## 208   gDec   sTol       0.000        0.000
## 209   gDec   sWth       0.000        0.000
## 210   gDec   sPrs       0.000        0.000
## 211   gDec   sEsc       0.000        0.000
## 212   gDec   sDis       0.000        0.000
## 213   gDec  pHead       0.000        0.000
## 214   gDec  pStom       0.000        0.000
## 215   gDec  pBack       0.000        0.000
## 216   gDec   pLow       0.000        0.000
## 217   gDec  pIrri       0.000        0.000
## 218   gDec  pNerv       0.000        0.000
## 219   gDec pSleep       0.000        0.000
## 220   gDec pDizzy       0.000        0.000
## 221   gDec     WB       0.000        0.000
## 222   gDec   lone       0.000        0.000
## 223   gDis   sPre       0.000        0.000
## 224   gDis   sTol       0.000        0.000
## 225   gDis   sPrs       0.000        0.000
## 226   gDis   sEsc       0.000        0.000
## 227   gDis   sPbl       0.000        0.000
## 228   gDis   sDec       0.000        0.000
## 229   gDis   sCfl       0.000        0.000
## 230   gDis  pHead       0.000        0.000
## 231   gDis  pStom       0.000        0.000
## 232   gDis  pBack       0.000        0.000
## 233   gDis   pLow       0.000        0.000
## 234   gDis  pIrri       0.000        0.000
## 235   gDis  pNerv       0.000        0.000
## 236   gDis pSleep       0.000        0.000
## 237   gDis pDizzy       0.000        0.000
## 238   gDis     WB       0.000        0.000
## 239   gDis   lone       0.000        0.000
## 240   gCfl   sPre       0.000        0.000
## 241   gCfl   sTol       0.000        0.000
## 242   gCfl   sWth       0.000        0.000
## 243   gCfl   sPrs       0.000        0.000
## 244   gCfl   sEsc       0.000        0.000
## 245   gCfl   sDec       0.000        0.000
## 246   gCfl   sDis       0.000        0.000
## 247   gCfl  pHead       0.000        0.000
## 248   gCfl  pStom       0.000        0.000
## 249   gCfl  pBack       0.000        0.000
## 250   gCfl   pLow       0.000        0.000
## 251   gCfl  pIrri       0.000        0.000
## 252   gCfl  pNerv       0.000        0.000
## 253   gCfl pSleep       0.000        0.000
## 254   gCfl pDizzy       0.000        0.000
## 255   gCfl     WB       0.000        0.000
## 256   gCfl   lone       0.000        0.000
## 257   sPre   sPbl       0.000        0.000
## 258   sPre  pHead       0.000        0.000
## 259   sPre  pStom       0.000        0.000
## 260   sPre  pBack       0.000        0.000
## 261   sPre   pLow       0.000        0.000
## 262   sPre  pIrri       0.000        0.000
## 263   sPre  pNerv       0.000        0.000
## 264   sPre pSleep       0.000        0.000
## 265   sPre pDizzy       0.000        0.000
## 266   sPre     WB       0.000        0.000
## 267   sPre   lone       0.000        0.000
## 268   sTol   sDec       0.000        0.000
## 269   sTol   sDis       0.000        0.000
## 270   sTol  pHead       0.000        0.000
## 271   sTol  pStom       0.000        0.000
## 272   sTol  pBack       0.000        0.000
## 273   sTol   pLow       0.000        0.000
## 274   sTol  pIrri       0.000        0.000
## 275   sTol  pNerv       0.000        0.000
## 276   sTol pSleep       0.000        0.000
## 277   sTol pDizzy       0.000        0.000
## 278   sTol     WB       0.000        0.000
## 279   sTol   lone       0.000        0.000
## 280   sWth   sPrs       0.000        0.000
## 281   sWth   sPbl       0.000        0.000
## 282   sWth  pHead       0.000        0.000
## 283   sWth  pStom       0.000        0.000
## 284   sWth  pBack       0.000        0.000
## 285   sWth   pLow       0.000        0.000
## 286   sWth  pIrri       0.000        0.000
## 287   sWth  pNerv       0.000        0.000
## 288   sWth pSleep       0.000        0.000
## 289   sWth pDizzy       0.000        0.000
## 290   sWth     WB       0.000        0.000
## 291   sWth   lone       0.000        0.000
## 292   sPrs   sPbl       0.000        0.000
## 293   sPrs   sDis       0.000        0.000
## 294   sPrs   sCfl       0.000        0.000
## 295   sPrs  pHead       0.000        0.000
## 296   sPrs  pStom       0.000        0.000
## 297   sPrs  pBack       0.000        0.000
## 298   sPrs   pLow       0.000        0.000
## 299   sPrs  pIrri       0.000        0.000
## 300   sPrs  pNerv       0.000        0.000
## 301   sPrs pSleep       0.000        0.000
## 302   sPrs pDizzy       0.000        0.000
## 303   sPrs     WB       0.000        0.000
## 304   sPrs   lone       0.000        0.000
## 305   sEsc   sPbl       0.000        0.000
## 306   sEsc   sDec       0.000        0.000
## 307   sEsc   sDis       0.000        0.000
## 308   sEsc   sCfl       0.000        0.000
## 309   sEsc  pHead       0.000        0.000
## 310   sEsc  pStom       0.000        0.000
## 311   sEsc  pBack       0.000        0.000
## 312   sEsc  pIrri       0.000        0.000
## 313   sEsc pSleep       0.000        0.000
## 314   sEsc pDizzy       0.000        0.000
## 315   sEsc   lone       0.000        0.000
## 316   sPbl  pHead       0.000        0.000
## 317   sPbl  pStom       0.000        0.000
## 318   sPbl  pBack       0.000        0.000
## 319   sPbl   pLow       0.000        0.000
## 320   sPbl  pIrri       0.000        0.000
## 321   sPbl  pNerv       0.000        0.000
## 322   sPbl pSleep       0.000        0.000
## 323   sPbl pDizzy       0.000        0.000
## 324   sPbl     WB       0.000        0.000
## 325   sPbl   lone       0.000        0.000
## 326   sDec  pHead       0.000        0.000
## 327   sDec  pStom       0.000        0.000
## 328   sDec  pBack       0.000        0.000
## 329   sDec   pLow       0.000        0.000
## 330   sDec  pIrri       0.000        0.000
## 331   sDec  pNerv       0.000        0.000
## 332   sDec pSleep       0.000        0.000
## 333   sDec pDizzy       0.000        0.000
## 334   sDec     WB       0.000        0.000
## 335   sDec   lone       0.000        0.000
## 336   sDis  pHead       0.000        0.000
## 337   sDis  pStom       0.000        0.000
## 338   sDis  pBack       0.000        0.000
## 339   sDis   pLow       0.000        0.000
## 340   sDis  pIrri       0.000        0.000
## 341   sDis  pNerv       0.000        0.000
## 342   sDis pSleep       0.000        0.000
## 343   sDis pDizzy       0.000        0.000
## 344   sDis     WB       0.000        0.000
## 345   sDis   lone       0.000        0.000
## 346   sCfl  pHead       0.000        0.000
## 347   sCfl  pStom       0.000        0.000
## 348   sCfl  pBack       0.000        0.000
## 349   sCfl   pLow       0.000        0.000
## 350   sCfl  pIrri       0.000        0.000
## 351   sCfl  pNerv       0.000        0.000
## 352   sCfl pSleep       0.000        0.000
## 353   sCfl pDizzy       0.000        0.000
## 354   sCfl     WB       0.000        0.000
## 355  pHead  pBack       0.000        0.000
## 356  pHead  pIrri       0.000        0.000
## 357  pHead     WB       0.000        0.000
## 358  pHead   lone       0.000        0.000
## 359  pStom   pLow       0.000        0.000
## 360  pStom  pIrri       0.000        0.000
## 361  pStom  pNerv       0.000        0.000
## 362  pStom pSleep       0.000        0.000
## 363  pStom     WB       0.000        0.000
## 364  pStom   lone       0.000        0.000
## 365  pBack  pNerv       0.000        0.000
## 366  pBack pSleep       0.000        0.000
## 367  pBack     WB       0.000        0.000
## 368  pBack   lone       0.000        0.000
## 369   pLow pDizzy       0.000        0.000
## 370  pIrri pSleep       0.000        0.000
## 371  pIrri pDizzy       0.000        0.000
## 372  pIrri   lone       0.000        0.000
## 373  pNerv pDizzy       0.000        0.000
## 374  pNerv     WB       0.000        0.000
## 375  pNerv   lone       0.000        0.000
## 376 pSleep   lone       0.000        0.000
## 377 pDizzy     WB       0.000        0.000
## 378 pDizzy   lone       0.000        0.000
```

#### Centrality

**The centrality estimates are relatively stable when the
correlation stability coefficient (CS coefficient) is ≥ 0.5**
while set at correlation stability = 0.7 (Epskamp et al.,
2018). Centrality indices with CS coefficient of 0.25-0.5 should be
interpreted with caution; CS coefficient < 0.25 indicated large
instability of the centrality index.

```
##   group        centrality    CS_r07
## 1  Boys          strength 0.7500149
## 2  Boys         closeness 0.7500149
## 3  Boys       betweenness 0.7500149
## 4  Boys expectedInfluence 0.7500149
## 5 Girls          strength 0.7500149
## 6 Girls         closeness 0.7500149
## 7 Girls       betweenness 0.7500149
## 8 Girls expectedInfluence 0.7500149
```

#### Bootstrapped Edges

### MT

#### Figure

#### Edges

```
##       From     To Weight_Boys Weight_Girls
## 1     gPbl   gCfl       0.686        0.826
## 2     gCfl   sCfl       0.569        0.663
## 3     gDec   sDec       0.626        0.482
## 4     sDec   sCfl       0.616        0.396
## 5     gDis   sDis       0.551        0.592
## 6     sPbl   sCfl       0.405        0.556
## 7     gPbl   gDec       0.094        0.549
## 8     sTol   sWth       0.536        0.545
## 9     sPre   sTol       0.312        0.538
## 10    gEsc   sEsc       0.529        0.447
## 11    sPbl   sDec       0.157        0.507
## 12    gPre   gTol       0.329        0.471
## 13    gPre   gEsc       0.416        0.365
## 14    gPbl   sPbl       0.229        0.407
## 15    gTol   gEsc       0.000        0.404
## 16    gDis   gCfl       0.402        0.194
## 17    gPre   gWth       0.232        0.381
## 18    gPbl   gDis       0.130        0.375
## 19    gTol   gPbl       0.360        0.249
## 20    gPre   gPrs       0.081        0.349
## 21    gWth   gCfl       0.089        0.342
## 22    gWth   sWth       0.326        0.258
## 23    gTol   sTol       0.197        0.320
## 24    gWth   gDec       0.195        0.317
## 25    gTol   gWth       0.314        0.306
## 26    sWth   sCfl       0.293        0.000
## 27    gWth   gPrs       0.071        0.292
## 28    sPrs   sPbl       0.247        0.287
## 29    sPre   sPrs       0.272        0.259
## 30    sPrs   sDis       0.000        0.272
## 31    pLow  pIrri       0.267        0.243
## 32    sPre   sWth       0.259        0.237
## 33    gPrs   gDec       0.248        0.134
## 34    gDec   gCfl       0.225        0.247
## 35    sWth   sEsc       0.233        0.241
## 36    sPrs   sEsc       0.240        0.190
## 37   pHead  pStom       0.214        0.240
## 38   pIrri  pNerv       0.238        0.193
## 39    gPrs   gDis       0.000        0.230
## 40      WB   lone      -0.228       -0.208
## 41    sPre   sDis       0.223        0.000
## 42    pLow  pNerv       0.181        0.223
## 43    gPrs   gEsc       0.222        0.000
## 44    sDis   sCfl       0.219        0.000
## 45    pLow   lone       0.203        0.219
## 46   pHead pDizzy       0.163        0.208
## 47    gPrs   gPbl       0.192        0.205
## 48    gPrs   sPrs       0.199        0.000
## 49    gPre   gPbl       0.190        0.166
## 50    sEsc   sPbl       0.184        0.161
## 51    gWth   gPbl       0.182        0.071
## 52    sDec   sDis       0.179        0.101
## 53    sWth   sPbl       0.178        0.000
## 54    sPre   sEsc       0.177        0.116
## 55    gDec   gDis       0.175        0.111
## 56    gWth   gDis       0.153        0.174
## 57    sTol   sCfl       0.173        0.000
## 58   pNerv pSleep       0.173        0.069
## 59    gTol   gDis       0.000        0.170
## 60    pLow     WB       0.000       -0.170
## 61    gPre   sTol       0.167        0.000
## 62    pLow pDizzy       0.097        0.162
## 63    gCfl   sPbl       0.159        0.000
## 64    gPre   gDis       0.000        0.155
## 65   pHead  pBack       0.149        0.144
## 66   pBack   pLow       0.149        0.129
## 67   pStom  pBack       0.148        0.137
## 68  pSleep pDizzy       0.148        0.148
## 69    gWth   gEsc       0.147        0.122
## 70    gWth   sTol       0.145        0.000
## 71    gEsc   gDec       0.143        0.000
## 72   pStom pDizzy       0.090        0.143
## 73    gEsc   gCfl       0.140        0.000
## 74    sTol   sPrs       0.140        0.000
## 75    sWth   sDis       0.000        0.139
## 76    sTol   sPbl       0.137        0.122
## 77    sPbl   sDis       0.116        0.137
## 78    sEsc     WB       0.000       -0.126
## 79    sPre   sDec       0.000        0.123
## 80    gPre   sPre       0.088        0.112
## 81    gPrs   gCfl       0.112        0.000
## 82    gTol   gDec       0.109        0.071
## 83   pNerv   lone       0.105        0.074
## 84    gTol   sWth       0.099        0.000
## 85    gEsc   gPbl       0.096        0.086
## 86    gTol   gPrs       0.093        0.000
## 87    sPrs   sCfl       0.093        0.000
## 88    sEsc   lone       0.000        0.093
## 89    gPre   gDec       0.092        0.000
## 90    sEsc  pNerv       0.090        0.000
## 91    gWth   sPre       0.082        0.000
## 92   pHead   pLow       0.080        0.000
## 93    gTol   gCfl       0.078        0.000
## 94    sPre   sCfl       0.076        0.000
## 95   pIrri pSleep       0.000        0.071
## 96  pSleep   lone       0.070        0.000
## 97   pHead pSleep       0.069        0.057
## 98   pStom  pNerv       0.067        0.000
## 99   pIrri pDizzy       0.000        0.052
## 100   gPre   gCfl       0.000        0.000
## 101   gPre   sWth       0.000        0.000
## 102   gPre   sPrs       0.000        0.000
## 103   gPre   sEsc       0.000        0.000
## 104   gPre   sPbl       0.000        0.000
## 105   gPre   sDec       0.000        0.000
## 106   gPre   sDis       0.000        0.000
## 107   gPre   sCfl       0.000        0.000
## 108   gPre  pHead       0.000        0.000
## 109   gPre  pStom       0.000        0.000
## 110   gPre  pBack       0.000        0.000
## 111   gPre   pLow       0.000        0.000
## 112   gPre  pIrri       0.000        0.000
## 113   gPre  pNerv       0.000        0.000
## 114   gPre pSleep       0.000        0.000
## 115   gPre pDizzy       0.000        0.000
## 116   gPre     WB       0.000        0.000
## 117   gPre   lone       0.000        0.000
## 118   gTol   sPre       0.000        0.000
## 119   gTol   sPrs       0.000        0.000
## 120   gTol   sEsc       0.000        0.000
## 121   gTol   sPbl       0.000        0.000
## 122   gTol   sDec       0.000        0.000
## 123   gTol   sDis       0.000        0.000
## 124   gTol   sCfl       0.000        0.000
## 125   gTol  pHead       0.000        0.000
## 126   gTol  pStom       0.000        0.000
## 127   gTol  pBack       0.000        0.000
## 128   gTol   pLow       0.000        0.000
## 129   gTol  pIrri       0.000        0.000
## 130   gTol  pNerv       0.000        0.000
## 131   gTol pSleep       0.000        0.000
## 132   gTol pDizzy       0.000        0.000
## 133   gTol     WB       0.000        0.000
## 134   gTol   lone       0.000        0.000
## 135   gWth   sPrs       0.000        0.000
## 136   gWth   sEsc       0.000        0.000
## 137   gWth   sPbl       0.000        0.000
## 138   gWth   sDec       0.000        0.000
## 139   gWth   sDis       0.000        0.000
## 140   gWth   sCfl       0.000        0.000
## 141   gWth  pHead       0.000        0.000
## 142   gWth  pStom       0.000        0.000
## 143   gWth  pBack       0.000        0.000
## 144   gWth   pLow       0.000        0.000
## 145   gWth  pIrri       0.000        0.000
## 146   gWth  pNerv       0.000        0.000
## 147   gWth pSleep       0.000        0.000
## 148   gWth pDizzy       0.000        0.000
## 149   gWth     WB       0.000        0.000
## 150   gWth   lone       0.000        0.000
## 151   gPrs   sPre       0.000        0.000
## 152   gPrs   sTol       0.000        0.000
## 153   gPrs   sWth       0.000        0.000
## 154   gPrs   sEsc       0.000        0.000
## 155   gPrs   sPbl       0.000        0.000
## 156   gPrs   sDec       0.000        0.000
## 157   gPrs   sDis       0.000        0.000
## 158   gPrs   sCfl       0.000        0.000
## 159   gPrs  pHead       0.000        0.000
## 160   gPrs  pStom       0.000        0.000
## 161   gPrs  pBack       0.000        0.000
## 162   gPrs   pLow       0.000        0.000
## 163   gPrs  pIrri       0.000        0.000
## 164   gPrs  pNerv       0.000        0.000
## 165   gPrs pSleep       0.000        0.000
## 166   gPrs pDizzy       0.000        0.000
## 167   gPrs     WB       0.000        0.000
## 168   gPrs   lone       0.000        0.000
## 169   gEsc   gDis       0.000        0.000
## 170   gEsc   sPre       0.000        0.000
## 171   gEsc   sTol       0.000        0.000
## 172   gEsc   sWth       0.000        0.000
## 173   gEsc   sPrs       0.000        0.000
## 174   gEsc   sPbl       0.000        0.000
## 175   gEsc   sDec       0.000        0.000
## 176   gEsc   sDis       0.000        0.000
## 177   gEsc   sCfl       0.000        0.000
## 178   gEsc  pHead       0.000        0.000
## 179   gEsc  pStom       0.000        0.000
## 180   gEsc  pBack       0.000        0.000
## 181   gEsc   pLow       0.000        0.000
## 182   gEsc  pIrri       0.000        0.000
## 183   gEsc  pNerv       0.000        0.000
## 184   gEsc pSleep       0.000        0.000
## 185   gEsc pDizzy       0.000        0.000
## 186   gEsc     WB       0.000        0.000
## 187   gEsc   lone       0.000        0.000
## 188   gPbl   sPre       0.000        0.000
## 189   gPbl   sTol       0.000        0.000
## 190   gPbl   sWth       0.000        0.000
## 191   gPbl   sPrs       0.000        0.000
## 192   gPbl   sEsc       0.000        0.000
## 193   gPbl   sDec       0.000        0.000
## 194   gPbl   sDis       0.000        0.000
## 195   gPbl   sCfl       0.000        0.000
## 196   gPbl  pHead       0.000        0.000
## 197   gPbl  pStom       0.000        0.000
## 198   gPbl  pBack       0.000        0.000
## 199   gPbl   pLow       0.000        0.000
## 200   gPbl  pIrri       0.000        0.000
## 201   gPbl  pNerv       0.000        0.000
## 202   gPbl pSleep       0.000        0.000
## 203   gPbl pDizzy       0.000        0.000
## 204   gPbl     WB       0.000        0.000
## 205   gPbl   lone       0.000        0.000
## 206   gDec   sPre       0.000        0.000
## 207   gDec   sTol       0.000        0.000
## 208   gDec   sWth       0.000        0.000
## 209   gDec   sPrs       0.000        0.000
## 210   gDec   sEsc       0.000        0.000
## 211   gDec   sPbl       0.000        0.000
## 212   gDec   sDis       0.000        0.000
## 213   gDec   sCfl       0.000        0.000
## 214   gDec  pHead       0.000        0.000
## 215   gDec  pStom       0.000        0.000
## 216   gDec  pBack       0.000        0.000
## 217   gDec   pLow       0.000        0.000
## 218   gDec  pIrri       0.000        0.000
## 219   gDec  pNerv       0.000        0.000
## 220   gDec pSleep       0.000        0.000
## 221   gDec pDizzy       0.000        0.000
## 222   gDec     WB       0.000        0.000
## 223   gDec   lone       0.000        0.000
## 224   gDis   sPre       0.000        0.000
## 225   gDis   sTol       0.000        0.000
## 226   gDis   sWth       0.000        0.000
## 227   gDis   sPrs       0.000        0.000
## 228   gDis   sEsc       0.000        0.000
## 229   gDis   sPbl       0.000        0.000
## 230   gDis   sDec       0.000        0.000
## 231   gDis   sCfl       0.000        0.000
## 232   gDis  pHead       0.000        0.000
## 233   gDis  pStom       0.000        0.000
## 234   gDis  pBack       0.000        0.000
## 235   gDis   pLow       0.000        0.000
## 236   gDis  pIrri       0.000        0.000
## 237   gDis  pNerv       0.000        0.000
## 238   gDis pSleep       0.000        0.000
## 239   gDis pDizzy       0.000        0.000
## 240   gDis     WB       0.000        0.000
## 241   gDis   lone       0.000        0.000
## 242   gCfl   sPre       0.000        0.000
## 243   gCfl   sTol       0.000        0.000
## 244   gCfl   sWth       0.000        0.000
## 245   gCfl   sPrs       0.000        0.000
## 246   gCfl   sEsc       0.000        0.000
## 247   gCfl   sDec       0.000        0.000
## 248   gCfl   sDis       0.000        0.000
## 249   gCfl  pHead       0.000        0.000
## 250   gCfl  pStom       0.000        0.000
## 251   gCfl  pBack       0.000        0.000
## 252   gCfl   pLow       0.000        0.000
## 253   gCfl  pIrri       0.000        0.000
## 254   gCfl  pNerv       0.000        0.000
## 255   gCfl pSleep       0.000        0.000
## 256   gCfl pDizzy       0.000        0.000
## 257   gCfl     WB       0.000        0.000
## 258   gCfl   lone       0.000        0.000
## 259   sPre   sPbl       0.000        0.000
## 260   sPre  pHead       0.000        0.000
## 261   sPre  pStom       0.000        0.000
## 262   sPre  pBack       0.000        0.000
## 263   sPre   pLow       0.000        0.000
## 264   sPre  pIrri       0.000        0.000
## 265   sPre  pNerv       0.000        0.000
## 266   sPre pSleep       0.000        0.000
## 267   sPre pDizzy       0.000        0.000
## 268   sPre     WB       0.000        0.000
## 269   sPre   lone       0.000        0.000
## 270   sTol   sEsc       0.000        0.000
## 271   sTol   sDec       0.000        0.000
## 272   sTol   sDis       0.000        0.000
## 273   sTol  pHead       0.000        0.000
## 274   sTol  pStom       0.000        0.000
## 275   sTol  pBack       0.000        0.000
## 276   sTol   pLow       0.000        0.000
## 277   sTol  pIrri       0.000        0.000
## 278   sTol  pNerv       0.000        0.000
## 279   sTol pSleep       0.000        0.000
## 280   sTol pDizzy       0.000        0.000
## 281   sTol     WB       0.000        0.000
## 282   sTol   lone       0.000        0.000
## 283   sWth   sPrs       0.000        0.000
## 284   sWth   sDec       0.000        0.000
## 285   sWth  pHead       0.000        0.000
## 286   sWth  pStom       0.000        0.000
## 287   sWth  pBack       0.000        0.000
## 288   sWth   pLow       0.000        0.000
## 289   sWth  pIrri       0.000        0.000
## 290   sWth  pNerv       0.000        0.000
## 291   sWth pSleep       0.000        0.000
## 292   sWth pDizzy       0.000        0.000
## 293   sWth     WB       0.000        0.000
## 294   sWth   lone       0.000        0.000
## 295   sPrs   sDec       0.000        0.000
## 296   sPrs  pHead       0.000        0.000
## 297   sPrs  pStom       0.000        0.000
## 298   sPrs  pBack       0.000        0.000
## 299   sPrs   pLow       0.000        0.000
## 300   sPrs  pIrri       0.000        0.000
## 301   sPrs  pNerv       0.000        0.000
## 302   sPrs pSleep       0.000        0.000
## 303   sPrs pDizzy       0.000        0.000
## 304   sPrs     WB       0.000        0.000
## 305   sPrs   lone       0.000        0.000
## 306   sEsc   sDec       0.000        0.000
## 307   sEsc   sDis       0.000        0.000
## 308   sEsc   sCfl       0.000        0.000
## 309   sEsc  pHead       0.000        0.000
## 310   sEsc  pStom       0.000        0.000
## 311   sEsc  pBack       0.000        0.000
## 312   sEsc   pLow       0.000        0.000
## 313   sEsc  pIrri       0.000        0.000
## 314   sEsc pSleep       0.000        0.000
## 315   sEsc pDizzy       0.000        0.000
## 316   sPbl  pHead       0.000        0.000
## 317   sPbl  pStom       0.000        0.000
## 318   sPbl  pBack       0.000        0.000
## 319   sPbl   pLow       0.000        0.000
## 320   sPbl  pIrri       0.000        0.000
## 321   sPbl  pNerv       0.000        0.000
## 322   sPbl pSleep       0.000        0.000
## 323   sPbl pDizzy       0.000        0.000
## 324   sPbl     WB       0.000        0.000
## 325   sPbl   lone       0.000        0.000
## 326   sDec  pHead       0.000        0.000
## 327   sDec  pStom       0.000        0.000
## 328   sDec  pBack       0.000        0.000
## 329   sDec   pLow       0.000        0.000
## 330   sDec  pIrri       0.000        0.000
## 331   sDec  pNerv       0.000        0.000
## 332   sDec pSleep       0.000        0.000
## 333   sDec pDizzy       0.000        0.000
## 334   sDec     WB       0.000        0.000
## 335   sDec   lone       0.000        0.000
## 336   sDis  pHead       0.000        0.000
## 337   sDis  pStom       0.000        0.000
## 338   sDis  pBack       0.000        0.000
## 339   sDis   pLow       0.000        0.000
## 340   sDis  pIrri       0.000        0.000
## 341   sDis  pNerv       0.000        0.000
## 342   sDis pSleep       0.000        0.000
## 343   sDis pDizzy       0.000        0.000
## 344   sDis     WB       0.000        0.000
## 345   sDis   lone       0.000        0.000
## 346   sCfl  pHead       0.000        0.000
## 347   sCfl  pStom       0.000        0.000
## 348   sCfl  pBack       0.000        0.000
## 349   sCfl   pLow       0.000        0.000
## 350   sCfl  pIrri       0.000        0.000
## 351   sCfl  pNerv       0.000        0.000
## 352   sCfl pSleep       0.000        0.000
## 353   sCfl pDizzy       0.000        0.000
## 354   sCfl     WB       0.000        0.000
## 355   sCfl   lone       0.000        0.000
## 356  pHead  pIrri       0.000        0.000
## 357  pHead  pNerv       0.000        0.000
## 358  pHead     WB       0.000        0.000
## 359  pHead   lone       0.000        0.000
## 360  pStom   pLow       0.000        0.000
## 361  pStom  pIrri       0.000        0.000
## 362  pStom pSleep       0.000        0.000
## 363  pStom     WB       0.000        0.000
## 364  pStom   lone       0.000        0.000
## 365  pBack  pIrri       0.000        0.000
## 366  pBack  pNerv       0.000        0.000
## 367  pBack pSleep       0.000        0.000
## 368  pBack pDizzy       0.000        0.000
## 369  pBack     WB       0.000        0.000
## 370  pBack   lone       0.000        0.000
## 371   pLow pSleep       0.000        0.000
## 372  pIrri     WB       0.000        0.000
## 373  pIrri   lone       0.000        0.000
## 374  pNerv pDizzy       0.000        0.000
## 375  pNerv     WB       0.000        0.000
## 376 pSleep     WB       0.000        0.000
## 377 pDizzy     WB       0.000        0.000
## 378 pDizzy   lone       0.000        0.000
```

#### Centrality

**The centrality estimates are relatively stable when the
correlation stability coefficient (CS coefficient) is ≥ 0.5**
while set at correlation stability = 0.7 (Epskamp et al.,
2018). Centrality indices with CS coefficient of 0.25-0.5 should be
interpreted with caution; CS coefficient < 0.25 indicated large
instability of the centrality index.

```
##   group        centrality    CS_r07
## 1  Boys          strength 0.7500149
## 2  Boys         closeness 0.7500149
## 3  Boys       betweenness 0.7500149
## 4  Boys expectedInfluence 0.7500149
## 5 Girls          strength 0.7500149
## 6 Girls         closeness 0.7500149
## 7 Girls       betweenness 0.7500149
## 8 Girls expectedInfluence 0.7500149
```

#### Bootstrapped Edges

### NL

#### Figure

#### Edges

```
##       From     To Weight_Boys Weight_Girls
## 1     gCfl   sCfl       0.915        1.347
## 2     gEsc   sEsc       1.086        0.985
## 3     gTol   gWth       0.519        0.753
## 4     gDis   sDis       0.569        0.744
## 5     gDec   sDec       0.736        0.498
## 6     gWth   sWth       0.552        0.640
## 7     gPbl   sPbl       0.628        0.485
## 8     gTol   sTol       0.414        0.601
## 9     sTol   sWth       0.558        0.546
## 10    gPre   gTol       0.354        0.526
## 11    gPre   gWth       0.307        0.515
## 12    sPbl   sCfl       0.281        0.512
## 13    gDec   gCfl       0.464        0.501
## 14    sPrs   sDis       0.083        0.488
## 15    gPbl   gCfl       0.486        0.267
## 16    sDec   sCfl       0.475        0.000
## 17    gPrs   gDis       0.226        0.474
## 18    gPbl   gDec       0.125        0.473
## 19    sTol   sCfl       0.447        0.108
## 20    sPrs   sDec       0.415        0.000
## 21    gPrs   gEsc       0.000        0.411
## 22    gPre   sPre       0.176        0.403
## 23    sPbl   sDec       0.395        0.298
## 24    gPrs   gDec       0.391        0.103
## 25    sPre   sWth       0.305        0.356
## 26    gPrs   gCfl       0.254        0.341
## 27    gPre   gPrs       0.337        0.215
## 28    gPrs   sPrs       0.284        0.336
## 29    gPre   gDis       0.325        0.071
## 30    pLow   lone       0.304        0.324
## 31    gDis   gCfl       0.323        0.179
## 32    gWth   gPbl       0.314        0.098
## 33    gTol   gEsc       0.157        0.295
## 34    sPrs   sEsc       0.295        0.189
## 35    sTol   sEsc       0.293        0.141
## 36    sPre   sTol       0.285        0.122
## 37    gEsc   gDec       0.277        0.269
## 38    gTol   gDis       0.269        0.177
## 39    gPrs   gPbl       0.000        0.266
## 40    pLow  pIrri       0.226        0.243
## 41    sWth   sPbl       0.237        0.000
## 42   pHead pDizzy       0.213        0.237
## 43    gWth   gDis       0.091        0.236
## 44    gWth   gEsc       0.155        0.235
## 45    sTol   sDis       0.231        0.097
## 46    pLow     WB      -0.168       -0.228
## 47    gDec   gDis       0.223        0.118
## 48    sPbl   sDis       0.222        0.221
## 49    gWth   gPrs       0.213        0.210
## 50    sWth   sEsc       0.100        0.213
## 51   pHead  pStom       0.211        0.164
## 52    gCfl   sPbl       0.205        0.000
## 53      WB   lone      -0.165       -0.205
## 54    sEsc   sPbl       0.204        0.000
## 55    sDec   sDis       0.201        0.142
## 56    gPre   gPbl       0.200        0.000
## 57    gTol   gDec       0.100        0.199
## 58   pIrri  pNerv       0.162        0.198
## 59    sTol   sPbl       0.196        0.000
## 60    sWth   sCfl       0.191        0.000
## 61    gPre   gEsc       0.188        0.118
## 62    gTol   gPrs       0.182        0.169
## 63    gWth   gCfl       0.180        0.000
## 64    sEsc   sDec       0.000        0.178
## 65    sTol   sDec       0.102        0.176
## 66   pStom pDizzy       0.168        0.142
## 67    gTol   gPbl       0.157        0.146
## 68    sWth   sDec       0.081        0.151
## 69    gEsc   lone       0.141        0.121
## 70    sWth   sDis       0.137        0.086
## 71    pLow pDizzy       0.094        0.134
## 72   pHead  pBack       0.081        0.125
## 73   pIrri     WB      -0.124       -0.122
## 74    sEsc   sDis       0.000        0.122
## 75    gTol   sDec       0.119        0.000
## 76    sWth  pIrri       0.000        0.114
## 77   pNerv pSleep       0.088        0.114
## 78    gWth   gDec       0.094        0.112
## 79    gPbl   gDis       0.112        0.000
## 80    gDis   sTol       0.112        0.000
## 81    sWth   sPrs       0.111        0.000
## 82    gPrs   sDec       0.000        0.110
## 83    sCfl   pLow       0.000        0.109
## 84    pLow  pNerv       0.108        0.000
## 85    sPre   sPrs       0.000        0.105
## 86   pNerv pDizzy       0.057        0.103
## 87   pBack pDizzy       0.102        0.075
## 88    sEsc   pLow       0.058        0.101
## 89    gPrs   sDis       0.099        0.000
## 90    gDec   lone       0.098        0.000
## 91   pStom  pNerv       0.048        0.095
## 92   pStom  pBack       0.094        0.072
## 93   pIrri pSleep       0.094        0.091
## 94   pHead  pIrri       0.091        0.085
## 95    gTol   sWth       0.090        0.000
## 96    sEsc  pNerv       0.000        0.089
## 97  pSleep pDizzy       0.085        0.000
## 98    gDis   sDec       0.082        0.000
## 99    sPre   sEsc       0.000        0.082
## 100   sPrs   sPbl       0.080        0.000
## 101 pSleep     WB      -0.079       -0.066
## 102  pBack   pLow       0.076        0.000
## 103  pHead pSleep       0.071        0.064
## 104   sPre   sDec       0.068        0.000
## 105   sEsc   lone       0.068        0.061
## 106  pIrri   lone       0.063        0.000
## 107  pStom pSleep       0.061        0.000
## 108  pNerv   lone       0.060        0.000
## 109   gPbl  pIrri       0.059        0.000
## 110  pHead   pLow       0.059        0.000
## 111   gPrs   sWth       0.053        0.000
## 112  pBack pSleep       0.053        0.000
## 113   gPre   gDec       0.000        0.000
## 114   gPre   gCfl       0.000        0.000
## 115   gPre   sTol       0.000        0.000
## 116   gPre   sWth       0.000        0.000
## 117   gPre   sPrs       0.000        0.000
## 118   gPre   sEsc       0.000        0.000
## 119   gPre   sPbl       0.000        0.000
## 120   gPre   sDec       0.000        0.000
## 121   gPre   sDis       0.000        0.000
## 122   gPre   sCfl       0.000        0.000
## 123   gPre  pHead       0.000        0.000
## 124   gPre  pStom       0.000        0.000
## 125   gPre  pBack       0.000        0.000
## 126   gPre   pLow       0.000        0.000
## 127   gPre  pIrri       0.000        0.000
## 128   gPre  pNerv       0.000        0.000
## 129   gPre pSleep       0.000        0.000
## 130   gPre pDizzy       0.000        0.000
## 131   gPre     WB       0.000        0.000
## 132   gPre   lone       0.000        0.000
## 133   gTol   gCfl       0.000        0.000
## 134   gTol   sPre       0.000        0.000
## 135   gTol   sPrs       0.000        0.000
## 136   gTol   sEsc       0.000        0.000
## 137   gTol   sPbl       0.000        0.000
## 138   gTol   sDis       0.000        0.000
## 139   gTol   sCfl       0.000        0.000
## 140   gTol  pHead       0.000        0.000
## 141   gTol  pStom       0.000        0.000
## 142   gTol  pBack       0.000        0.000
## 143   gTol   pLow       0.000        0.000
## 144   gTol  pIrri       0.000        0.000
## 145   gTol  pNerv       0.000        0.000
## 146   gTol pSleep       0.000        0.000
## 147   gTol pDizzy       0.000        0.000
## 148   gTol     WB       0.000        0.000
## 149   gTol   lone       0.000        0.000
## 150   gWth   sPre       0.000        0.000
## 151   gWth   sTol       0.000        0.000
## 152   gWth   sPrs       0.000        0.000
## 153   gWth   sEsc       0.000        0.000
## 154   gWth   sPbl       0.000        0.000
## 155   gWth   sDec       0.000        0.000
## 156   gWth   sDis       0.000        0.000
## 157   gWth   sCfl       0.000        0.000
## 158   gWth  pHead       0.000        0.000
## 159   gWth  pStom       0.000        0.000
## 160   gWth  pBack       0.000        0.000
## 161   gWth   pLow       0.000        0.000
## 162   gWth  pIrri       0.000        0.000
## 163   gWth  pNerv       0.000        0.000
## 164   gWth pSleep       0.000        0.000
## 165   gWth pDizzy       0.000        0.000
## 166   gWth     WB       0.000        0.000
## 167   gWth   lone       0.000        0.000
## 168   gPrs   sPre       0.000        0.000
## 169   gPrs   sTol       0.000        0.000
## 170   gPrs   sEsc       0.000        0.000
## 171   gPrs   sPbl       0.000        0.000
## 172   gPrs   sCfl       0.000        0.000
## 173   gPrs  pHead       0.000        0.000
## 174   gPrs  pStom       0.000        0.000
## 175   gPrs  pBack       0.000        0.000
## 176   gPrs   pLow       0.000        0.000
## 177   gPrs  pIrri       0.000        0.000
## 178   gPrs  pNerv       0.000        0.000
## 179   gPrs pSleep       0.000        0.000
## 180   gPrs pDizzy       0.000        0.000
## 181   gPrs     WB       0.000        0.000
## 182   gPrs   lone       0.000        0.000
## 183   gEsc   gPbl       0.000        0.000
## 184   gEsc   gDis       0.000        0.000
## 185   gEsc   gCfl       0.000        0.000
## 186   gEsc   sPre       0.000        0.000
## 187   gEsc   sTol       0.000        0.000
## 188   gEsc   sWth       0.000        0.000
## 189   gEsc   sPrs       0.000        0.000
## 190   gEsc   sPbl       0.000        0.000
## 191   gEsc   sDec       0.000        0.000
## 192   gEsc   sDis       0.000        0.000
## 193   gEsc   sCfl       0.000        0.000
## 194   gEsc  pHead       0.000        0.000
## 195   gEsc  pStom       0.000        0.000
## 196   gEsc  pBack       0.000        0.000
## 197   gEsc   pLow       0.000        0.000
## 198   gEsc  pIrri       0.000        0.000
## 199   gEsc  pNerv       0.000        0.000
## 200   gEsc pSleep       0.000        0.000
## 201   gEsc pDizzy       0.000        0.000
## 202   gEsc     WB       0.000        0.000
## 203   gPbl   sPre       0.000        0.000
## 204   gPbl   sTol       0.000        0.000
## 205   gPbl   sWth       0.000        0.000
## 206   gPbl   sPrs       0.000        0.000
## 207   gPbl   sEsc       0.000        0.000
## 208   gPbl   sDec       0.000        0.000
## 209   gPbl   sDis       0.000        0.000
## 210   gPbl   sCfl       0.000        0.000
## 211   gPbl  pHead       0.000        0.000
## 212   gPbl  pStom       0.000        0.000
## 213   gPbl  pBack       0.000        0.000
## 214   gPbl   pLow       0.000        0.000
## 215   gPbl  pNerv       0.000        0.000
## 216   gPbl pSleep       0.000        0.000
## 217   gPbl pDizzy       0.000        0.000
## 218   gPbl     WB       0.000        0.000
## 219   gPbl   lone       0.000        0.000
## 220   gDec   sPre       0.000        0.000
## 221   gDec   sTol       0.000        0.000
## 222   gDec   sWth       0.000        0.000
## 223   gDec   sPrs       0.000        0.000
## 224   gDec   sEsc       0.000        0.000
## 225   gDec   sPbl       0.000        0.000
## 226   gDec   sDis       0.000        0.000
## 227   gDec   sCfl       0.000        0.000
## 228   gDec  pHead       0.000        0.000
## 229   gDec  pStom       0.000        0.000
## 230   gDec  pBack       0.000        0.000
## 231   gDec   pLow       0.000        0.000
## 232   gDec  pIrri       0.000        0.000
## 233   gDec  pNerv       0.000        0.000
## 234   gDec pSleep       0.000        0.000
## 235   gDec pDizzy       0.000        0.000
## 236   gDec     WB       0.000        0.000
## 237   gDis   sPre       0.000        0.000
## 238   gDis   sWth       0.000        0.000
## 239   gDis   sPrs       0.000        0.000
## 240   gDis   sEsc       0.000        0.000
## 241   gDis   sPbl       0.000        0.000
## 242   gDis   sCfl       0.000        0.000
## 243   gDis  pHead       0.000        0.000
## 244   gDis  pStom       0.000        0.000
## 245   gDis  pBack       0.000        0.000
## 246   gDis   pLow       0.000        0.000
## 247   gDis  pIrri       0.000        0.000
## 248   gDis  pNerv       0.000        0.000
## 249   gDis pSleep       0.000        0.000
## 250   gDis pDizzy       0.000        0.000
## 251   gDis     WB       0.000        0.000
## 252   gDis   lone       0.000        0.000
## 253   gCfl   sPre       0.000        0.000
## 254   gCfl   sTol       0.000        0.000
## 255   gCfl   sWth       0.000        0.000
## 256   gCfl   sPrs       0.000        0.000
## 257   gCfl   sEsc       0.000        0.000
## 258   gCfl   sDec       0.000        0.000
## 259   gCfl   sDis       0.000        0.000
## 260   gCfl  pHead       0.000        0.000
## 261   gCfl  pStom       0.000        0.000
## 262   gCfl  pBack       0.000        0.000
## 263   gCfl   pLow       0.000        0.000
## 264   gCfl  pIrri       0.000        0.000
## 265   gCfl  pNerv       0.000        0.000
## 266   gCfl pSleep       0.000        0.000
## 267   gCfl pDizzy       0.000        0.000
## 268   gCfl     WB       0.000        0.000
## 269   gCfl   lone       0.000        0.000
## 270   sPre   sPbl       0.000        0.000
## 271   sPre   sDis       0.000        0.000
## 272   sPre   sCfl       0.000        0.000
## 273   sPre  pHead       0.000        0.000
## 274   sPre  pStom       0.000        0.000
## 275   sPre  pBack       0.000        0.000
## 276   sPre   pLow       0.000        0.000
## 277   sPre  pIrri       0.000        0.000
## 278   sPre  pNerv       0.000        0.000
## 279   sPre pSleep       0.000        0.000
## 280   sPre pDizzy       0.000        0.000
## 281   sPre     WB       0.000        0.000
## 282   sPre   lone       0.000        0.000
## 283   sTol   sPrs       0.000        0.000
## 284   sTol  pHead       0.000        0.000
## 285   sTol  pStom       0.000        0.000
## 286   sTol  pBack       0.000        0.000
## 287   sTol   pLow       0.000        0.000
## 288   sTol  pIrri       0.000        0.000
## 289   sTol  pNerv       0.000        0.000
## 290   sTol pSleep       0.000        0.000
## 291   sTol pDizzy       0.000        0.000
## 292   sTol     WB       0.000        0.000
## 293   sTol   lone       0.000        0.000
## 294   sWth  pHead       0.000        0.000
## 295   sWth  pStom       0.000        0.000
## 296   sWth  pBack       0.000        0.000
## 297   sWth   pLow       0.000        0.000
## 298   sWth  pNerv       0.000        0.000
## 299   sWth pSleep       0.000        0.000
## 300   sWth pDizzy       0.000        0.000
## 301   sWth     WB       0.000        0.000
## 302   sWth   lone       0.000        0.000
## 303   sPrs   sCfl       0.000        0.000
## 304   sPrs  pHead       0.000        0.000
## 305   sPrs  pStom       0.000        0.000
## 306   sPrs  pBack       0.000        0.000
## 307   sPrs   pLow       0.000        0.000
## 308   sPrs  pIrri       0.000        0.000
## 309   sPrs  pNerv       0.000        0.000
## 310   sPrs pSleep       0.000        0.000
## 311   sPrs pDizzy       0.000        0.000
## 312   sPrs     WB       0.000        0.000
## 313   sPrs   lone       0.000        0.000
## 314   sEsc   sCfl       0.000        0.000
## 315   sEsc  pHead       0.000        0.000
## 316   sEsc  pStom       0.000        0.000
## 317   sEsc  pBack       0.000        0.000
## 318   sEsc  pIrri       0.000        0.000
## 319   sEsc pSleep       0.000        0.000
## 320   sEsc pDizzy       0.000        0.000
## 321   sEsc     WB       0.000        0.000
## 322   sPbl  pHead       0.000        0.000
## 323   sPbl  pStom       0.000        0.000
## 324   sPbl  pBack       0.000        0.000
## 325   sPbl   pLow       0.000        0.000
## 326   sPbl  pIrri       0.000        0.000
## 327   sPbl  pNerv       0.000        0.000
## 328   sPbl pSleep       0.000        0.000
## 329   sPbl pDizzy       0.000        0.000
## 330   sPbl     WB       0.000        0.000
## 331   sPbl   lone       0.000        0.000
## 332   sDec  pHead       0.000        0.000
## 333   sDec  pStom       0.000        0.000
## 334   sDec  pBack       0.000        0.000
## 335   sDec   pLow       0.000        0.000
## 336   sDec  pIrri       0.000        0.000
## 337   sDec  pNerv       0.000        0.000
## 338   sDec pSleep       0.000        0.000
## 339   sDec pDizzy       0.000        0.000
## 340   sDec     WB       0.000        0.000
## 341   sDec   lone       0.000        0.000
## 342   sDis   sCfl       0.000        0.000
## 343   sDis  pHead       0.000        0.000
## 344   sDis  pStom       0.000        0.000
## 345   sDis  pBack       0.000        0.000
## 346   sDis   pLow       0.000        0.000
## 347   sDis  pIrri       0.000        0.000
## 348   sDis  pNerv       0.000        0.000
## 349   sDis pSleep       0.000        0.000
## 350   sDis pDizzy       0.000        0.000
## 351   sDis     WB       0.000        0.000
## 352   sDis   lone       0.000        0.000
## 353   sCfl  pHead       0.000        0.000
## 354   sCfl  pStom       0.000        0.000
## 355   sCfl  pBack       0.000        0.000
## 356   sCfl  pIrri       0.000        0.000
## 357   sCfl  pNerv       0.000        0.000
## 358   sCfl pSleep       0.000        0.000
## 359   sCfl pDizzy       0.000        0.000
## 360   sCfl     WB       0.000        0.000
## 361   sCfl   lone       0.000        0.000
## 362  pHead  pNerv       0.000        0.000
## 363  pHead     WB       0.000        0.000
## 364  pHead   lone       0.000        0.000
## 365  pStom   pLow       0.000        0.000
## 366  pStom  pIrri       0.000        0.000
## 367  pStom     WB       0.000        0.000
## 368  pStom   lone       0.000        0.000
## 369  pBack  pIrri       0.000        0.000
## 370  pBack  pNerv       0.000        0.000
## 371  pBack     WB       0.000        0.000
## 372  pBack   lone       0.000        0.000
## 373   pLow pSleep       0.000        0.000
## 374  pIrri pDizzy       0.000        0.000
## 375  pNerv     WB       0.000        0.000
## 376 pSleep   lone       0.000        0.000
## 377 pDizzy     WB       0.000        0.000
## 378 pDizzy   lone       0.000        0.000
```

#### Centrality

**The centrality estimates are relatively stable when the
correlation stability coefficient (CS coefficient) is ≥ 0.5**
while set at correlation stability = 0.7 (Epskamp et al.,
2018). Centrality indices with CS coefficient of 0.25-0.5 should be
interpreted with caution; CS coefficient < 0.25 indicated large
instability of the centrality index.

```
##   group        centrality    CS_r07
## 1  Boys          strength 0.7500149
## 2  Boys         closeness 0.7500149
## 3  Boys       betweenness 0.7500149
## 4  Boys expectedInfluence 0.7500149
## 5 Girls          strength 0.7500149
## 6 Girls         closeness 0.7500149
## 7 Girls       betweenness 0.7500149
## 8 Girls expectedInfluence 0.7500149
```

#### Bootstrapped Edges

### GB-SCT

#### Figure

#### Edges

```
##       From     To Weight_Boys Weight_Girls
## 1     gPbl   gCfl       0.658        0.817
## 2     gTol   gWth       0.659        0.754
## 3     sPre   sTol       0.701        0.741
## 4     gDec   gCfl       0.345        0.684
## 5     gDec   sDec       0.661        0.144
## 6     gDis   gCfl       0.655        0.416
## 7     gDec   gDis       0.268        0.643
## 8     sTol   sWth       0.592        0.572
## 9     sPbl   sCfl       0.558        0.507
## 10    gPre   gWth       0.310        0.552
## 11    sDec   sCfl       0.535        0.388
## 12    gWth   gDis       0.532        0.225
## 13    gPre   gTol       0.516        0.528
## 14    gWth   gPrs       0.502        0.325
## 15    gEsc   sEsc       0.480        0.353
## 16    gPbl   sPbl       0.463        0.000
## 17    sPrs   sDec       0.440        0.222
## 18    gDis   sDis       0.364        0.431
## 19    gCfl   sCfl       0.430        0.000
## 20    gPre   gEsc       0.078        0.423
## 21    sPre   sWth       0.420        0.265
## 22    sPbl   sDec       0.257        0.401
## 23    gEsc   gDec       0.228        0.392
## 24    pLow  pNerv       0.351        0.200
## 25    sPrs   sEsc       0.314        0.225
## 26    sDec   sDis       0.291        0.000
## 27    gPre   gPrs       0.150        0.290
## 28    pLow  pIrri       0.171        0.289
## 29   pHead pDizzy       0.173        0.285
## 30    gWth   gDec       0.281        0.147
## 31    gWth   sWth       0.278        0.000
## 32    pLow   lone       0.269        0.240
## 33    gWth   gCfl       0.000        0.265
## 34    gPrs   gDis       0.000        0.249
## 35    sEsc   sDis       0.237        0.000
## 36    sPbl   sDis       0.230        0.235
## 37    sWth   sPbl       0.228        0.100
## 38    gTol   gEsc       0.000        0.225
## 39    gDis   sTol       0.000        0.224
## 40    gTol   gPrs       0.101        0.221
## 41    pLow     WB      -0.194       -0.221
## 42    gTol   gDec       0.122        0.220
## 43   pHead  pStom       0.206        0.115
## 44    sTol   sDis       0.203        0.000
## 45    sEsc   sPbl       0.203        0.182
## 46    sTol   sEsc       0.202        0.106
## 47      WB   lone      -0.201       -0.176
## 48    gPbl   gDis       0.000        0.184
## 49   pStom pDizzy       0.152        0.182
## 50    sPre   sPbl       0.180        0.000
## 51    gEsc   gDis       0.000        0.170
## 52    sTol   sPbl       0.170        0.132
## 53    sWth   sDis       0.000        0.170
## 54    gPbl   gDec       0.169        0.119
## 55    sWth   sDec       0.000        0.163
## 56    gWth   gPbl       0.000        0.161
## 57    sWth   sEsc       0.159        0.000
## 58    gPrs   gCfl       0.156        0.000
## 59    sTol   sCfl       0.000        0.155
## 60    sDis   sCfl       0.152        0.109
## 61    gWth   gEsc       0.146        0.000
## 62    gEsc   gPbl       0.140        0.000
## 63    sEsc  pNerv       0.140        0.000
## 64    sTol   sPrs       0.000        0.138
## 65    gPre   gDis       0.000        0.137
## 66  pSleep     WB      -0.137       -0.114
## 67   pStom  pBack       0.104        0.132
## 68    sDis     WB      -0.124        0.000
## 69   pStom   pLow       0.000        0.124
## 70    gPrs   gDec       0.000        0.123
## 71    gTol   gPbl       0.000        0.120
## 72    gEsc     WB      -0.119        0.000
## 73   pNerv pDizzy       0.000        0.113
## 74    sPrs   sDis       0.000        0.109
## 75   pHead   pLow       0.000        0.107
## 76  pSleep pDizzy       0.063        0.106
## 77    gTol   sWth       0.073        0.105
## 78   pHead pSleep       0.103        0.000
## 79    sEsc   lone       0.000        0.102
## 80   pHead  pBack       0.100        0.000
## 81    gWth   sTol       0.099        0.000
## 82  pSleep   lone       0.099        0.000
## 83   pIrri pDizzy       0.000        0.098
## 84   pNerv   lone       0.090        0.077
## 85    sPrs   sPbl       0.084        0.000
## 86   pBack pDizzy       0.077        0.000
## 87   pNerv pSleep       0.000        0.074
## 88    gPrs   sPbl       0.073        0.000
## 89   pIrri pSleep       0.068        0.000
## 90    gCfl   sTol       0.060        0.000
## 91    gPre   gPbl       0.000        0.000
## 92    gPre   gDec       0.000        0.000
## 93    gPre   gCfl       0.000        0.000
## 94    gPre   sPre       0.000        0.000
## 95    gPre   sTol       0.000        0.000
## 96    gPre   sWth       0.000        0.000
## 97    gPre   sPrs       0.000        0.000
## 98    gPre   sEsc       0.000        0.000
## 99    gPre   sPbl       0.000        0.000
## 100   gPre   sDec       0.000        0.000
## 101   gPre   sDis       0.000        0.000
## 102   gPre   sCfl       0.000        0.000
## 103   gPre  pHead       0.000        0.000
## 104   gPre  pStom       0.000        0.000
## 105   gPre  pBack       0.000        0.000
## 106   gPre   pLow       0.000        0.000
## 107   gPre  pIrri       0.000        0.000
## 108   gPre  pNerv       0.000        0.000
## 109   gPre pSleep       0.000        0.000
## 110   gPre pDizzy       0.000        0.000
## 111   gPre     WB       0.000        0.000
## 112   gPre   lone       0.000        0.000
## 113   gTol   gDis       0.000        0.000
## 114   gTol   gCfl       0.000        0.000
## 115   gTol   sPre       0.000        0.000
## 116   gTol   sTol       0.000        0.000
## 117   gTol   sPrs       0.000        0.000
## 118   gTol   sEsc       0.000        0.000
## 119   gTol   sPbl       0.000        0.000
## 120   gTol   sDec       0.000        0.000
## 121   gTol   sDis       0.000        0.000
## 122   gTol   sCfl       0.000        0.000
## 123   gTol  pHead       0.000        0.000
## 124   gTol  pStom       0.000        0.000
## 125   gTol  pBack       0.000        0.000
## 126   gTol   pLow       0.000        0.000
## 127   gTol  pIrri       0.000        0.000
## 128   gTol  pNerv       0.000        0.000
## 129   gTol pSleep       0.000        0.000
## 130   gTol pDizzy       0.000        0.000
## 131   gTol     WB       0.000        0.000
## 132   gTol   lone       0.000        0.000
## 133   gWth   sPre       0.000        0.000
## 134   gWth   sPrs       0.000        0.000
## 135   gWth   sEsc       0.000        0.000
## 136   gWth   sPbl       0.000        0.000
## 137   gWth   sDec       0.000        0.000
## 138   gWth   sDis       0.000        0.000
## 139   gWth   sCfl       0.000        0.000
## 140   gWth  pHead       0.000        0.000
## 141   gWth  pStom       0.000        0.000
## 142   gWth  pBack       0.000        0.000
## 143   gWth   pLow       0.000        0.000
## 144   gWth  pIrri       0.000        0.000
## 145   gWth  pNerv       0.000        0.000
## 146   gWth pSleep       0.000        0.000
## 147   gWth pDizzy       0.000        0.000
## 148   gWth     WB       0.000        0.000
## 149   gWth   lone       0.000        0.000
## 150   gPrs   gEsc       0.000        0.000
## 151   gPrs   gPbl       0.000        0.000
## 152   gPrs   sPre       0.000        0.000
## 153   gPrs   sTol       0.000        0.000
## 154   gPrs   sWth       0.000        0.000
## 155   gPrs   sPrs       0.000        0.000
## 156   gPrs   sEsc       0.000        0.000
## 157   gPrs   sDec       0.000        0.000
## 158   gPrs   sDis       0.000        0.000
## 159   gPrs   sCfl       0.000        0.000
## 160   gPrs  pHead       0.000        0.000
## 161   gPrs  pStom       0.000        0.000
## 162   gPrs  pBack       0.000        0.000
## 163   gPrs   pLow       0.000        0.000
## 164   gPrs  pIrri       0.000        0.000
## 165   gPrs  pNerv       0.000        0.000
## 166   gPrs pSleep       0.000        0.000
## 167   gPrs pDizzy       0.000        0.000
## 168   gPrs     WB       0.000        0.000
## 169   gPrs   lone       0.000        0.000
## 170   gEsc   gCfl       0.000        0.000
## 171   gEsc   sPre       0.000        0.000
## 172   gEsc   sTol       0.000        0.000
## 173   gEsc   sWth       0.000        0.000
## 174   gEsc   sPrs       0.000        0.000
## 175   gEsc   sPbl       0.000        0.000
## 176   gEsc   sDec       0.000        0.000
## 177   gEsc   sDis       0.000        0.000
## 178   gEsc   sCfl       0.000        0.000
## 179   gEsc  pHead       0.000        0.000
## 180   gEsc  pStom       0.000        0.000
## 181   gEsc  pBack       0.000        0.000
## 182   gEsc   pLow       0.000        0.000
## 183   gEsc  pIrri       0.000        0.000
## 184   gEsc  pNerv       0.000        0.000
## 185   gEsc pSleep       0.000        0.000
## 186   gEsc pDizzy       0.000        0.000
## 187   gEsc   lone       0.000        0.000
## 188   gPbl   sPre       0.000        0.000
## 189   gPbl   sTol       0.000        0.000
## 190   gPbl   sWth       0.000        0.000
## 191   gPbl   sPrs       0.000        0.000
## 192   gPbl   sEsc       0.000        0.000
## 193   gPbl   sDec       0.000        0.000
## 194   gPbl   sDis       0.000        0.000
## 195   gPbl   sCfl       0.000        0.000
## 196   gPbl  pHead       0.000        0.000
## 197   gPbl  pStom       0.000        0.000
## 198   gPbl  pBack       0.000        0.000
## 199   gPbl   pLow       0.000        0.000
## 200   gPbl  pIrri       0.000        0.000
## 201   gPbl  pNerv       0.000        0.000
## 202   gPbl pSleep       0.000        0.000
## 203   gPbl pDizzy       0.000        0.000
## 204   gPbl     WB       0.000        0.000
## 205   gPbl   lone       0.000        0.000
## 206   gDec   sPre       0.000        0.000
## 207   gDec   sTol       0.000        0.000
## 208   gDec   sWth       0.000        0.000
## 209   gDec   sPrs       0.000        0.000
## 210   gDec   sEsc       0.000        0.000
## 211   gDec   sPbl       0.000        0.000
## 212   gDec   sDis       0.000        0.000
## 213   gDec   sCfl       0.000        0.000
## 214   gDec  pHead       0.000        0.000
## 215   gDec  pStom       0.000        0.000
## 216   gDec  pBack       0.000        0.000
## 217   gDec   pLow       0.000        0.000
## 218   gDec  pIrri       0.000        0.000
## 219   gDec  pNerv       0.000        0.000
## 220   gDec pSleep       0.000        0.000
## 221   gDec pDizzy       0.000        0.000
## 222   gDec     WB       0.000        0.000
## 223   gDec   lone       0.000        0.000
## 224   gDis   sPre       0.000        0.000
## 225   gDis   sWth       0.000        0.000
## 226   gDis   sPrs       0.000        0.000
## 227   gDis   sEsc       0.000        0.000
## 228   gDis   sPbl       0.000        0.000
## 229   gDis   sDec       0.000        0.000
## 230   gDis   sCfl       0.000        0.000
## 231   gDis  pHead       0.000        0.000
## 232   gDis  pStom       0.000        0.000
## 233   gDis  pBack       0.000        0.000
## 234   gDis   pLow       0.000        0.000
## 235   gDis  pIrri       0.000        0.000
## 236   gDis  pNerv       0.000        0.000
## 237   gDis pSleep       0.000        0.000
## 238   gDis pDizzy       0.000        0.000
## 239   gDis     WB       0.000        0.000
## 240   gDis   lone       0.000        0.000
## 241   gCfl   sPre       0.000        0.000
## 242   gCfl   sWth       0.000        0.000
## 243   gCfl   sPrs       0.000        0.000
## 244   gCfl   sEsc       0.000        0.000
## 245   gCfl   sPbl       0.000        0.000
## 246   gCfl   sDec       0.000        0.000
## 247   gCfl   sDis       0.000        0.000
## 248   gCfl  pHead       0.000        0.000
## 249   gCfl  pStom       0.000        0.000
## 250   gCfl  pBack       0.000        0.000
## 251   gCfl   pLow       0.000        0.000
## 252   gCfl  pIrri       0.000        0.000
## 253   gCfl  pNerv       0.000        0.000
## 254   gCfl pSleep       0.000        0.000
## 255   gCfl pDizzy       0.000        0.000
## 256   gCfl     WB       0.000        0.000
## 257   gCfl   lone       0.000        0.000
## 258   sPre   sPrs       0.000        0.000
## 259   sPre   sEsc       0.000        0.000
## 260   sPre   sDec       0.000        0.000
## 261   sPre   sDis       0.000        0.000
## 262   sPre   sCfl       0.000        0.000
## 263   sPre  pHead       0.000        0.000
## 264   sPre  pStom       0.000        0.000
## 265   sPre  pBack       0.000        0.000
## 266   sPre   pLow       0.000        0.000
## 267   sPre  pIrri       0.000        0.000
## 268   sPre  pNerv       0.000        0.000
## 269   sPre pSleep       0.000        0.000
## 270   sPre pDizzy       0.000        0.000
## 271   sPre     WB       0.000        0.000
## 272   sPre   lone       0.000        0.000
## 273   sTol   sDec       0.000        0.000
## 274   sTol  pHead       0.000        0.000
## 275   sTol  pStom       0.000        0.000
## 276   sTol  pBack       0.000        0.000
## 277   sTol   pLow       0.000        0.000
## 278   sTol  pIrri       0.000        0.000
## 279   sTol  pNerv       0.000        0.000
## 280   sTol pSleep       0.000        0.000
## 281   sTol pDizzy       0.000        0.000
## 282   sTol     WB       0.000        0.000
## 283   sTol   lone       0.000        0.000
## 284   sWth   sPrs       0.000        0.000
## 285   sWth   sCfl       0.000        0.000
## 286   sWth  pHead       0.000        0.000
## 287   sWth  pStom       0.000        0.000
## 288   sWth  pBack       0.000        0.000
## 289   sWth   pLow       0.000        0.000
## 290   sWth  pIrri       0.000        0.000
## 291   sWth  pNerv       0.000        0.000
## 292   sWth pSleep       0.000        0.000
## 293   sWth pDizzy       0.000        0.000
## 294   sWth     WB       0.000        0.000
## 295   sWth   lone       0.000        0.000
## 296   sPrs   sCfl       0.000        0.000
## 297   sPrs  pHead       0.000        0.000
## 298   sPrs  pStom       0.000        0.000
## 299   sPrs  pBack       0.000        0.000
## 300   sPrs   pLow       0.000        0.000
## 301   sPrs  pIrri       0.000        0.000
## 302   sPrs  pNerv       0.000        0.000
## 303   sPrs pSleep       0.000        0.000
## 304   sPrs pDizzy       0.000        0.000
## 305   sPrs     WB       0.000        0.000
## 306   sPrs   lone       0.000        0.000
## 307   sEsc   sDec       0.000        0.000
## 308   sEsc   sCfl       0.000        0.000
## 309   sEsc  pHead       0.000        0.000
## 310   sEsc  pStom       0.000        0.000
## 311   sEsc  pBack       0.000        0.000
## 312   sEsc   pLow       0.000        0.000
## 313   sEsc  pIrri       0.000        0.000
## 314   sEsc pSleep       0.000        0.000
## 315   sEsc pDizzy       0.000        0.000
## 316   sEsc     WB       0.000        0.000
## 317   sPbl  pHead       0.000        0.000
## 318   sPbl  pStom       0.000        0.000
## 319   sPbl  pBack       0.000        0.000
## 320   sPbl   pLow       0.000        0.000
## 321   sPbl  pIrri       0.000        0.000
## 322   sPbl  pNerv       0.000        0.000
## 323   sPbl pSleep       0.000        0.000
## 324   sPbl pDizzy       0.000        0.000
## 325   sPbl     WB       0.000        0.000
## 326   sPbl   lone       0.000        0.000
## 327   sDec  pHead       0.000        0.000
## 328   sDec  pStom       0.000        0.000
## 329   sDec  pBack       0.000        0.000
## 330   sDec   pLow       0.000        0.000
## 331   sDec  pIrri       0.000        0.000
## 332   sDec  pNerv       0.000        0.000
## 333   sDec pSleep       0.000        0.000
## 334   sDec pDizzy       0.000        0.000
## 335   sDec     WB       0.000        0.000
## 336   sDec   lone       0.000        0.000
## 337   sDis  pHead       0.000        0.000
## 338   sDis  pStom       0.000        0.000
## 339   sDis  pBack       0.000        0.000
## 340   sDis   pLow       0.000        0.000
## 341   sDis  pIrri       0.000        0.000
## 342   sDis  pNerv       0.000        0.000
## 343   sDis pSleep       0.000        0.000
## 344   sDis pDizzy       0.000        0.000
## 345   sDis   lone       0.000        0.000
## 346   sCfl  pHead       0.000        0.000
## 347   sCfl  pStom       0.000        0.000
## 348   sCfl  pBack       0.000        0.000
## 349   sCfl   pLow       0.000        0.000
## 350   sCfl  pIrri       0.000        0.000
## 351   sCfl  pNerv       0.000        0.000
## 352   sCfl pSleep       0.000        0.000
## 353   sCfl pDizzy       0.000        0.000
## 354   sCfl     WB       0.000        0.000
## 355   sCfl   lone       0.000        0.000
## 356  pHead  pIrri       0.000        0.000
## 357  pHead  pNerv       0.000        0.000
## 358  pHead     WB       0.000        0.000
## 359  pHead   lone       0.000        0.000
## 360  pStom  pIrri       0.000        0.000
## 361  pStom  pNerv       0.000        0.000
## 362  pStom pSleep       0.000        0.000
## 363  pStom     WB       0.000        0.000
## 364  pStom   lone       0.000        0.000
## 365  pBack   pLow       0.000        0.000
## 366  pBack  pIrri       0.000        0.000
## 367  pBack  pNerv       0.000        0.000
## 368  pBack pSleep       0.000        0.000
## 369  pBack     WB       0.000        0.000
## 370  pBack   lone       0.000        0.000
## 371   pLow pSleep       0.000        0.000
## 372   pLow pDizzy       0.000        0.000
## 373  pIrri  pNerv       0.000        0.000
## 374  pIrri     WB       0.000        0.000
## 375  pIrri   lone       0.000        0.000
## 376  pNerv     WB       0.000        0.000
## 377 pDizzy     WB       0.000        0.000
## 378 pDizzy   lone       0.000        0.000
```

#### Centrality

**The centrality estimates are relatively stable when the
correlation stability coefficient (CS coefficient) is ≥ 0.5**
while set at correlation stability = 0.7 (Epskamp et al.,
2018). Centrality indices with CS coefficient of 0.25-0.5 should be
interpreted with caution; CS coefficient < 0.25 indicated large
instability of the centrality index.

```
##   group        centrality    CS_r07
## 1  Boys          strength 0.7500149
## 2  Boys         closeness 0.7500149
## 3  Boys       betweenness 0.7500149
## 4  Boys expectedInfluence 0.7500149
## 5 Girls          strength 0.7500149
## 6 Girls         closeness 0.7500149
## 7 Girls       betweenness 0.7500149
## 8 Girls expectedInfluence 0.7500149
```

#### Bootstrapped Edges

### SI

#### Figure

#### Edges

```
##       From     To Weight_Boys Weight_Girls
## 1     gPbl   gCfl       0.422        0.759
## 2     gTol   gWth       0.705        0.628
## 3     gPre   gWth       0.378        0.693
## 4     gEsc   sEsc       0.515        0.639
## 5     sTol   sWth       0.635        0.524
## 6     gDis   sDis       0.633        0.552
## 7     sPbl   sCfl       0.575        0.623
## 8     sPre   sTol       0.429        0.607
## 9     gDec   sDec       0.379        0.605
## 10    gPbl   gDis       0.548        0.509
## 11    sPre   sWth       0.534        0.382
## 12    sWth   sDec       0.534        0.215
## 13    gTol   gDis       0.297        0.507
## 14    gPrs   gDis       0.493        0.425
## 15    sDec   sCfl       0.473        0.489
## 16    gPre   gTol       0.474        0.462
## 17    gPrs   gPbl       0.268        0.449
## 18    gDec   gCfl       0.442        0.270
## 19    pLow  pIrri       0.407        0.441
## 20    gCfl   sCfl       0.439        0.427
## 21    gTol   gPbl       0.427        0.398
## 22    gEsc   gCfl       0.179        0.408
## 23    gEsc   gDec       0.160        0.396
## 24    sTol   sEsc       0.395        0.268
## 25    gDis   gCfl       0.393        0.180
## 26    sPbl   sDis       0.389        0.221
## 27    gDec   gDis       0.364        0.275
## 28    sPrs   sEsc       0.358        0.347
## 29    gPre   gEsc       0.336        0.317
## 30    gWth   gPrs       0.304        0.330
## 31    gPre   gDec       0.089        0.329
## 32    gWth   sWth       0.262        0.318
## 33    gPrs   gDec       0.292        0.316
## 34    sDec   sDis       0.306        0.239
## 35    gPre   gPbl       0.224        0.303
## 36    sTol   sPbl       0.301        0.297
## 37    gPbl   gDec       0.292        0.264
## 38    gPre   gCfl       0.291        0.000
## 39    gPre   gPrs       0.094        0.288
## 40    sDis   sCfl       0.286        0.144
## 41    pLow   lone       0.247        0.282
## 42    sWth   sDis       0.277        0.241
## 43    sEsc   sPbl       0.170        0.277
## 44   pHead pDizzy       0.223        0.277
## 45   pIrri  pNerv       0.270        0.244
## 46    sPre   sDis       0.207        0.266
## 47    gWth   gDec       0.264        0.265
## 48    gPbl   sPbl       0.251        0.000
## 49    gPrs   gCfl       0.248        0.158
## 50    sPre   sCfl       0.246        0.000
## 51    sEsc   sDec       0.238        0.124
## 52    gWth   gCfl       0.236        0.149
## 53    sPre   sPrs       0.109        0.230
## 54    sTol   sPrs       0.226        0.092
## 55    sPrs   sPbl       0.222        0.172
## 56    gPre   gDis       0.000        0.214
## 57    sPre   sDec       0.198        0.214
## 58    sPre   sEsc       0.159        0.212
## 59    gPrs   gEsc       0.208        0.000
## 60    sPrs   sDis       0.172        0.207
## 61    gWth   gEsc       0.185        0.205
## 62    gPrs   sPre       0.203        0.000
## 63    gPbl   sCfl       0.203        0.000
## 64    sWth   sCfl       0.201        0.172
## 65      WB   lone      -0.199       -0.187
## 66    sEsc   sDis       0.000        0.197
## 67    sWth   sPbl       0.185        0.066
## 68    sPbl   sDec       0.152        0.184
## 69    sTol   sDis       0.182        0.071
## 70    gTol   gPrs       0.168        0.064
## 71   pHead  pStom       0.167        0.160
## 72    gWth   gPbl       0.164        0.000
## 73    gPbl   sTol       0.159        0.000
## 74    gEsc   gPbl       0.152        0.158
## 75    gPre   sPre       0.107        0.156
## 76    sPre   sPbl       0.000        0.153
## 77    gWth   gDis       0.000        0.150
## 78    sEsc   sCfl       0.144        0.000
## 79    gDec   sCfl       0.000        0.139
## 80    sWth   sEsc       0.087        0.131
## 81    pLow     WB      -0.113       -0.128
## 82    gCfl   sPbl       0.000        0.125
## 83    sEsc     WB       0.000       -0.125
## 84   pStom pDizzy       0.066        0.125
## 85    gPrs   sWth       0.123        0.000
## 86    gEsc   gDis       0.000        0.123
## 87   pHead  pBack       0.094        0.120
## 88   pBack pDizzy       0.119        0.096
## 89    pLow pDizzy       0.091        0.116
## 90    gPrs   sPrs       0.114        0.000
## 91    gDis   sDec       0.000        0.114
## 92    gTol   sWth       0.111        0.000
## 93   pIrri     WB      -0.057       -0.111
## 94  pSleep     WB      -0.070       -0.110
## 95    gTol   gEsc       0.098        0.109
## 96   pNerv pSleep       0.109        0.107
## 97    pLow  pNerv       0.070        0.105
## 98   pNerv     WB      -0.105       -0.081
## 99    sWth   sPrs       0.102        0.000
## 100  pIrri pSleep       0.095        0.096
## 101 pSleep pDizzy       0.096        0.051
## 102   gWth   sDis       0.093        0.000
## 103   gEsc   sPbl       0.092        0.000
## 104   gDis   sTol       0.076        0.091
## 105   sTol   sCfl       0.061        0.090
## 106   sEsc   lone       0.063        0.090
## 107   gDis   sCfl       0.086        0.000
## 108  pStom  pBack       0.000        0.083
## 109  pStom  pNerv       0.080        0.062
## 110   gPbl   sWth       0.079        0.000
## 111   sTol   sDec       0.000        0.079
## 112  pBack  pNerv       0.079        0.069
## 113   sPrs   sCfl       0.077        0.000
## 114   gPre   sWth       0.072        0.000
## 115  pNerv pDizzy       0.000        0.071
## 116   sEsc  pNerv       0.000        0.070
## 117   gDis   sPbl       0.000        0.069
## 118   sPrs   sDec       0.000        0.066
## 119  pBack pSleep       0.066        0.000
## 120   gTol   sTol       0.000        0.061
## 121   gEsc   sPrs       0.059        0.000
## 122  pHead pSleep       0.058        0.044
## 123  pHead     WB      -0.058        0.000
## 124   gTol  pIrri       0.054        0.000
## 125   gWth   sDec       0.053        0.000
## 126  pIrri   lone       0.040        0.000
## 127   gPre   sTol       0.000        0.000
## 128   gPre   sPrs       0.000        0.000
## 129   gPre   sEsc       0.000        0.000
## 130   gPre   sPbl       0.000        0.000
## 131   gPre   sDec       0.000        0.000
## 132   gPre   sDis       0.000        0.000
## 133   gPre   sCfl       0.000        0.000
## 134   gPre  pHead       0.000        0.000
## 135   gPre  pStom       0.000        0.000
## 136   gPre  pBack       0.000        0.000
## 137   gPre   pLow       0.000        0.000
## 138   gPre  pIrri       0.000        0.000
## 139   gPre  pNerv       0.000        0.000
## 140   gPre pSleep       0.000        0.000
## 141   gPre pDizzy       0.000        0.000
## 142   gPre     WB       0.000        0.000
## 143   gPre   lone       0.000        0.000
## 144   gTol   gDec       0.000        0.000
## 145   gTol   gCfl       0.000        0.000
## 146   gTol   sPre       0.000        0.000
## 147   gTol   sPrs       0.000        0.000
## 148   gTol   sEsc       0.000        0.000
## 149   gTol   sPbl       0.000        0.000
## 150   gTol   sDec       0.000        0.000
## 151   gTol   sDis       0.000        0.000
## 152   gTol   sCfl       0.000        0.000
## 153   gTol  pHead       0.000        0.000
## 154   gTol  pStom       0.000        0.000
## 155   gTol  pBack       0.000        0.000
## 156   gTol   pLow       0.000        0.000
## 157   gTol  pNerv       0.000        0.000
## 158   gTol pSleep       0.000        0.000
## 159   gTol pDizzy       0.000        0.000
## 160   gTol     WB       0.000        0.000
## 161   gTol   lone       0.000        0.000
## 162   gWth   sPre       0.000        0.000
## 163   gWth   sTol       0.000        0.000
## 164   gWth   sPrs       0.000        0.000
## 165   gWth   sEsc       0.000        0.000
## 166   gWth   sPbl       0.000        0.000
## 167   gWth   sCfl       0.000        0.000
## 168   gWth  pHead       0.000        0.000
## 169   gWth  pStom       0.000        0.000
## 170   gWth  pBack       0.000        0.000
## 171   gWth   pLow       0.000        0.000
## 172   gWth  pIrri       0.000        0.000
## 173   gWth  pNerv       0.000        0.000
## 174   gWth pSleep       0.000        0.000
## 175   gWth pDizzy       0.000        0.000
## 176   gWth     WB       0.000        0.000
## 177   gWth   lone       0.000        0.000
## 178   gPrs   sTol       0.000        0.000
## 179   gPrs   sEsc       0.000        0.000
## 180   gPrs   sPbl       0.000        0.000
## 181   gPrs   sDec       0.000        0.000
## 182   gPrs   sDis       0.000        0.000
## 183   gPrs   sCfl       0.000        0.000
## 184   gPrs  pHead       0.000        0.000
## 185   gPrs  pStom       0.000        0.000
## 186   gPrs  pBack       0.000        0.000
## 187   gPrs   pLow       0.000        0.000
## 188   gPrs  pIrri       0.000        0.000
## 189   gPrs  pNerv       0.000        0.000
## 190   gPrs pSleep       0.000        0.000
## 191   gPrs pDizzy       0.000        0.000
## 192   gPrs     WB       0.000        0.000
## 193   gPrs   lone       0.000        0.000
## 194   gEsc   sPre       0.000        0.000
## 195   gEsc   sTol       0.000        0.000
## 196   gEsc   sWth       0.000        0.000
## 197   gEsc   sDec       0.000        0.000
## 198   gEsc   sDis       0.000        0.000
## 199   gEsc   sCfl       0.000        0.000
## 200   gEsc  pHead       0.000        0.000
## 201   gEsc  pStom       0.000        0.000
## 202   gEsc  pBack       0.000        0.000
## 203   gEsc   pLow       0.000        0.000
## 204   gEsc  pIrri       0.000        0.000
## 205   gEsc  pNerv       0.000        0.000
## 206   gEsc pSleep       0.000        0.000
## 207   gEsc pDizzy       0.000        0.000
## 208   gEsc     WB       0.000        0.000
## 209   gEsc   lone       0.000        0.000
## 210   gPbl   sPre       0.000        0.000
## 211   gPbl   sPrs       0.000        0.000
## 212   gPbl   sEsc       0.000        0.000
## 213   gPbl   sDec       0.000        0.000
## 214   gPbl   sDis       0.000        0.000
## 215   gPbl  pHead       0.000        0.000
## 216   gPbl  pStom       0.000        0.000
## 217   gPbl  pBack       0.000        0.000
## 218   gPbl   pLow       0.000        0.000
## 219   gPbl  pIrri       0.000        0.000
## 220   gPbl  pNerv       0.000        0.000
## 221   gPbl pSleep       0.000        0.000
## 222   gPbl pDizzy       0.000        0.000
## 223   gPbl     WB       0.000        0.000
## 224   gPbl   lone       0.000        0.000
## 225   gDec   sPre       0.000        0.000
## 226   gDec   sTol       0.000        0.000
## 227   gDec   sWth       0.000        0.000
## 228   gDec   sPrs       0.000        0.000
## 229   gDec   sEsc       0.000        0.000
## 230   gDec   sPbl       0.000        0.000
## 231   gDec   sDis       0.000        0.000
## 232   gDec  pHead       0.000        0.000
## 233   gDec  pStom       0.000        0.000
## 234   gDec  pBack       0.000        0.000
## 235   gDec   pLow       0.000        0.000
## 236   gDec  pIrri       0.000        0.000
## 237   gDec  pNerv       0.000        0.000
## 238   gDec pSleep       0.000        0.000
## 239   gDec pDizzy       0.000        0.000
## 240   gDec     WB       0.000        0.000
## 241   gDec   lone       0.000        0.000
## 242   gDis   sPre       0.000        0.000
## 243   gDis   sWth       0.000        0.000
## 244   gDis   sPrs       0.000        0.000
## 245   gDis   sEsc       0.000        0.000
## 246   gDis  pHead       0.000        0.000
## 247   gDis  pStom       0.000        0.000
## 248   gDis  pBack       0.000        0.000
## 249   gDis   pLow       0.000        0.000
## 250   gDis  pIrri       0.000        0.000
## 251   gDis  pNerv       0.000        0.000
## 252   gDis pSleep       0.000        0.000
## 253   gDis pDizzy       0.000        0.000
## 254   gDis     WB       0.000        0.000
## 255   gDis   lone       0.000        0.000
## 256   gCfl   sPre       0.000        0.000
## 257   gCfl   sTol       0.000        0.000
## 258   gCfl   sWth       0.000        0.000
## 259   gCfl   sPrs       0.000        0.000
## 260   gCfl   sEsc       0.000        0.000
## 261   gCfl   sDec       0.000        0.000
## 262   gCfl   sDis       0.000        0.000
## 263   gCfl  pHead       0.000        0.000
## 264   gCfl  pStom       0.000        0.000
## 265   gCfl  pBack       0.000        0.000
## 266   gCfl   pLow       0.000        0.000
## 267   gCfl  pIrri       0.000        0.000
## 268   gCfl  pNerv       0.000        0.000
## 269   gCfl pSleep       0.000        0.000
## 270   gCfl pDizzy       0.000        0.000
## 271   gCfl     WB       0.000        0.000
## 272   gCfl   lone       0.000        0.000
## 273   sPre  pHead       0.000        0.000
## 274   sPre  pStom       0.000        0.000
## 275   sPre  pBack       0.000        0.000
## 276   sPre   pLow       0.000        0.000
## 277   sPre  pIrri       0.000        0.000
## 278   sPre  pNerv       0.000        0.000
## 279   sPre pSleep       0.000        0.000
## 280   sPre pDizzy       0.000        0.000
## 281   sPre     WB       0.000        0.000
## 282   sPre   lone       0.000        0.000
## 283   sTol  pHead       0.000        0.000
## 284   sTol  pStom       0.000        0.000
## 285   sTol  pBack       0.000        0.000
## 286   sTol   pLow       0.000        0.000
## 287   sTol  pIrri       0.000        0.000
## 288   sTol  pNerv       0.000        0.000
## 289   sTol pSleep       0.000        0.000
## 290   sTol pDizzy       0.000        0.000
## 291   sTol     WB       0.000        0.000
## 292   sTol   lone       0.000        0.000
## 293   sWth  pHead       0.000        0.000
## 294   sWth  pStom       0.000        0.000
## 295   sWth  pBack       0.000        0.000
## 296   sWth   pLow       0.000        0.000
## 297   sWth  pIrri       0.000        0.000
## 298   sWth  pNerv       0.000        0.000
## 299   sWth pSleep       0.000        0.000
## 300   sWth pDizzy       0.000        0.000
## 301   sWth     WB       0.000        0.000
## 302   sWth   lone       0.000        0.000
## 303   sPrs  pHead       0.000        0.000
## 304   sPrs  pStom       0.000        0.000
## 305   sPrs  pBack       0.000        0.000
## 306   sPrs   pLow       0.000        0.000
## 307   sPrs  pIrri       0.000        0.000
## 308   sPrs  pNerv       0.000        0.000
## 309   sPrs pSleep       0.000        0.000
## 310   sPrs pDizzy       0.000        0.000
## 311   sPrs     WB       0.000        0.000
## 312   sPrs   lone       0.000        0.000
## 313   sEsc  pHead       0.000        0.000
## 314   sEsc  pStom       0.000        0.000
## 315   sEsc  pBack       0.000        0.000
## 316   sEsc   pLow       0.000        0.000
## 317   sEsc  pIrri       0.000        0.000
## 318   sEsc pSleep       0.000        0.000
## 319   sEsc pDizzy       0.000        0.000
## 320   sPbl  pHead       0.000        0.000
## 321   sPbl  pStom       0.000        0.000
## 322   sPbl  pBack       0.000        0.000
## 323   sPbl   pLow       0.000        0.000
## 324   sPbl  pIrri       0.000        0.000
## 325   sPbl  pNerv       0.000        0.000
## 326   sPbl pSleep       0.000        0.000
## 327   sPbl pDizzy       0.000        0.000
## 328   sPbl     WB       0.000        0.000
## 329   sPbl   lone       0.000        0.000
## 330   sDec  pHead       0.000        0.000
## 331   sDec  pStom       0.000        0.000
## 332   sDec  pBack       0.000        0.000
## 333   sDec   pLow       0.000        0.000
## 334   sDec  pIrri       0.000        0.000
## 335   sDec  pNerv       0.000        0.000
## 336   sDec pSleep       0.000        0.000
## 337   sDec pDizzy       0.000        0.000
## 338   sDec     WB       0.000        0.000
## 339   sDec   lone       0.000        0.000
## 340   sDis  pHead       0.000        0.000
## 341   sDis  pStom       0.000        0.000
## 342   sDis  pBack       0.000        0.000
## 343   sDis   pLow       0.000        0.000
## 344   sDis  pIrri       0.000        0.000
## 345   sDis  pNerv       0.000        0.000
## 346   sDis pSleep       0.000        0.000
## 347   sDis pDizzy       0.000        0.000
## 348   sDis     WB       0.000        0.000
## 349   sDis   lone       0.000        0.000
## 350   sCfl  pHead       0.000        0.000
## 351   sCfl  pStom       0.000        0.000
## 352   sCfl  pBack       0.000        0.000
## 353   sCfl   pLow       0.000        0.000
## 354   sCfl  pIrri       0.000        0.000
## 355   sCfl  pNerv       0.000        0.000
## 356   sCfl pSleep       0.000        0.000
## 357   sCfl pDizzy       0.000        0.000
## 358   sCfl     WB       0.000        0.000
## 359   sCfl   lone       0.000        0.000
## 360  pHead   pLow       0.000        0.000
## 361  pHead  pIrri       0.000        0.000
## 362  pHead  pNerv       0.000        0.000
## 363  pHead   lone       0.000        0.000
## 364  pStom   pLow       0.000        0.000
## 365  pStom  pIrri       0.000        0.000
## 366  pStom pSleep       0.000        0.000
## 367  pStom     WB       0.000        0.000
## 368  pStom   lone       0.000        0.000
## 369  pBack   pLow       0.000        0.000
## 370  pBack  pIrri       0.000        0.000
## 371  pBack     WB       0.000        0.000
## 372  pBack   lone       0.000        0.000
## 373   pLow pSleep       0.000        0.000
## 374  pIrri pDizzy       0.000        0.000
## 375  pNerv   lone       0.000        0.000
## 376 pSleep   lone       0.000        0.000
## 377 pDizzy     WB       0.000        0.000
## 378 pDizzy   lone       0.000        0.000
```

#### Centrality

**The centrality estimates are relatively stable when the
correlation stability coefficient (CS coefficient) is ≥ 0.5**
while set at correlation stability = 0.7 (Epskamp et al.,
2018). Centrality indices with CS coefficient of 0.25-0.5 should be
interpreted with caution; CS coefficient < 0.25 indicated large
instability of the centrality index.

```
##   group        centrality    CS_r07
## 1  Boys          strength 0.7500149
## 2  Boys         closeness 0.7500149
## 3  Boys       betweenness 0.7500149
## 4  Boys expectedInfluence 0.7500149
## 5 Girls          strength 0.7500149
## 6 Girls         closeness 0.7500149
## 7 Girls       betweenness 0.7500149
## 8 Girls expectedInfluence 0.7500149
```

#### Bootstrapped Edges
